# Synthesis of Benzo[4,5]thiazolo[2,3-*c*][1,2,4]triazole Derivatives via C-H Bond Functionalization of Disulfide Intermediates

**DOI:** 10.3390/molecules27051464

**Published:** 2022-02-22

**Authors:** Luis G. Ardón-Muñoz, Jeanne L. Bolliger

**Affiliations:** Department of Chemistry, Oklahoma State University, 107 Physical Sciences, Stillwater, OK 74078-3071, USA; lardonm@okstate.edu

**Keywords:** heteroaromatics, C-H bond functionalization, oxidative cyclization

## Abstract

Many nitrogen- and sulfur-containing heterocyclic compounds exhibit biological activity. Among these heterocycles are benzo[4,5]thiazolo[2,3-*c*][1,2,4]triazoles for which two main synthetic approaches exist. Here we report a new synthetic protocol that allows the preparation of these tricyclic compounds via the oxidation of a mercaptophenyl moiety to its corresponding disulfide. Subsequent C-H bond functionalization is thought to enable an intramolecular ring closure, thus forming the desired benzo[4,5]thiazolo[2,3-*c*][1,2,4]triazole. This method combines a high functional group tolerance with short reaction times and good to excellent yields.

## 1. Introduction

**Figure 1:**
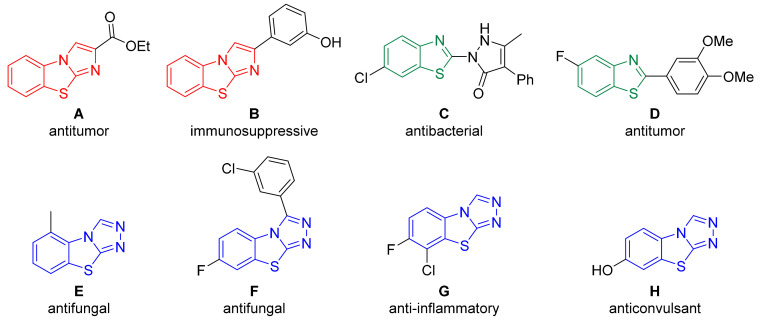
Examples of bioactive benzothiazoles. (**A**) antitumor [24]; (**B**) immunosuppressive [25]; (**C**) antibacterial [26]; (**D**) antitumor [27]; (**E**) antifungal [29,30,31]; (**F**) antifungal [32]; (**G**) anti–inflammatory [33]; (**H**) anticonvulsant [34].

Sulfur- and nitrogen-containing heterocycles are present in many natural products [1,2], agrochemicals [3,4,5], commercially available drugs [6,7], and compounds with the potential to become active pharmaceutical ingredients [8,9,10,11,12,13,14]. As a result, there is continued interest in developing new methods for the synthesis of biologically active fused heterocycles incorporating the benzothiazole fragment [15,16,17,18,19,20,21,22,23]. Some of these fused heterocyclic scaffolds with proven biological activities are shown in Figure 1. While the ester-substituted tricyclic benzo[d]imidazo [2,1-*b*]thiazole, **A,** shows antitumor properties [24], the related phenol derivative, **B,** shows immunosuppressive activity [25]. The bicyclic benzothiazole, **C,** acts as an antibiotic [26], whereas **D** is an antitumor compound with the potential to be applied as a PET imaging agent [27,28]. Among the biologically active benzo[4,5]thiazolo[2,3-*c*][1,2,4]triazole derivatives, **E**–**H** are the commercially available fungicide tricyclazole (compound **E**), which is used to treat rice blast, and **F,** which also exhibits antifungal properties [29,30,31,32]. **G** displays anti-inflammatory activity [33], and **H** shows promising results as an anticonvulsant agent [34].

**Scheme 1:**
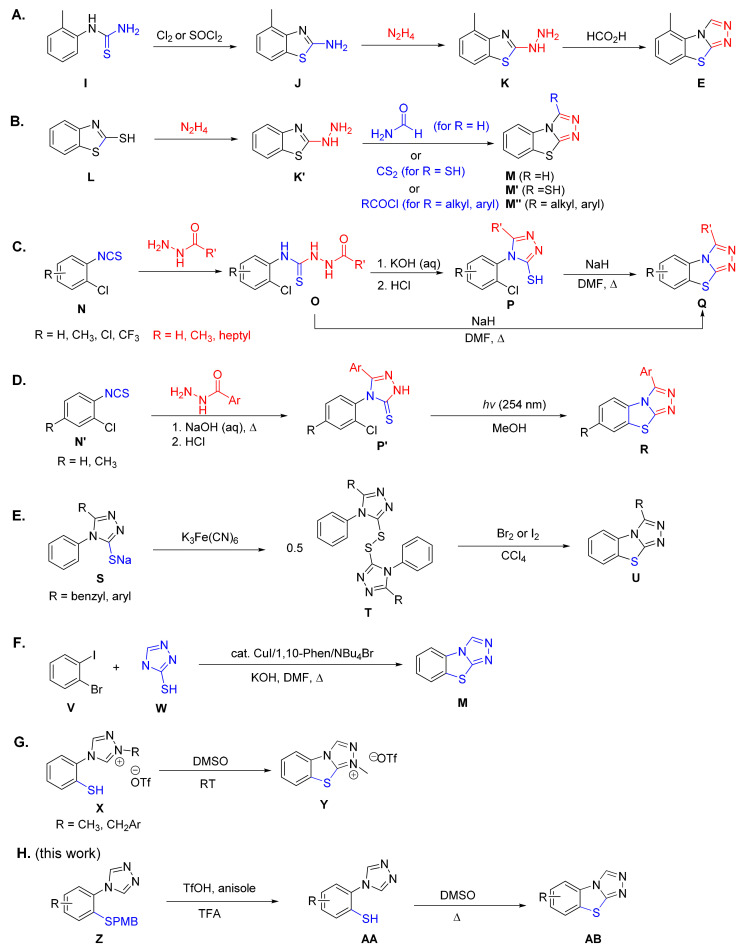
Synthetic routes leading to benzo[4,5]thiazolo[2,3-*c*][1,2,4]triazole derivatives. (**A**) Synthesis of the commercially available fungicide tricyclazole via a 2-aminobenzothiazole intermediate [35]. (**B**) A related synthesis starting from a 2-mercaptobenzothiazole derivative [32,36,37,38,39]. (**C**) Synthesis of benzothiazolotriazole derivatives from a 1-chloro-2-isothiocyanatobenzene species [40]. (**D**) A photochemical approach by 254 nm irradiation [41]. (**E**) I Oxidative cyclization of sodium salts followed by halogenation [42]. (**F**) Copper catalyzed diarylation of 3-mercaptotriazole [45]. (**G**) Oxidation of benzothiazolotriazolium salts to *N*-substituted benzothiazolotriazolium derivatives [46,47]. (**H**) This work: Synthesis of benzothiazolotriazole derivatives via C-H bond functionalization.

Even though benzo[4,5]thiazolo[2,3-*c*][1,2,4]triazoles are known to exhibit a broad range of biological activities, synthetic methods for obtaining this moiety remain limited and often lack functional group tolerance. The majority of these compounds are prepared by first forming the thiazole ring to obtain a benzothiazole derivative, followed by the construction of the triazole unit (Scheme 1A,B) [15,23,32,33,34]. Scheme 1A demonstrates this route using tricyclazole (**E**) as an example [35]. The thiourea, **I**, can be obtained by treatment of the appropriate aniline with potassium thiocyanate and is often not isolated before the subsequent oxidative cyclization to the 2-aminobenzothiazole, **J**. An exchange of the amino substituent with a hydrazine results in the 2-hydrazinylbenzothiazole, **K**, which, in the presence of a one-carbon electrophile, such as formic acid, gives the triazole ring of tricyclazole, **E** [35]. As depicted in Scheme 1B, 2-hydrazinylbenzothiazole, **K**′, can alternatively be prepared from 2-mercaptobenzothiazole, **L** [36]. The reaction of **K**′ with formamide or formic acid leads to the unsubstituted product, **M**, while the reaction with carbon disulfide has been used to obtain the sulfur derivative, **M**′ [37,38]. Substituted triazoles, such as **M**″, can either be obtained directly from **K**′ and the appropriate acid chloride or in two steps by using an aldehyde, followed by the addition of an oxidant [32,37,39].

An alternative approach for the synthesis of benzo[4,5]thiazolo[2,3-*c*][1,2,4]triazoles starts with a 3-mercaptotriazole derivative that is fused with the benzene ring, thus forming the thiazole ring in the last step (Scheme 1C,F). An example of this synthetic route is shown in Scheme 1C, where 1-chloro-2-isothiocyanatobenzene, **N**, is reacted with a hydrazide derivative to give compound **O [40]**. In the presence of a strong base, such as sodium hydride in boiling DMF, the tricyclic product, **Q**, can be obtained directly from **O**. Insight into the mechanism of this reaction was gained by the use of a weaker base, which allowed the isolation of the reaction intermediate, **P**, upon acidification. Subsequent treatment of this intermediate, **P**, with sodium hydride in DMF under reflux affords the tricyclic compound, **Q**. As shown in Scheme 1D, cyclization of this intermediate, **P**′, can also be achieved photochemically by subjecting it to 254 nm irradiation to yield compound **R** in a moderate yield [41]. Scheme 1E highlights a different route to the triazolo species (**U**) via two sequential oxidation steps. In the first step, a sodium salt (**S**) is converted to its disulfide (**T**), which, in the presence of bromine or iodine, undergoes an oxidative cyclization to the target molecule, **U** [42]. Unfortunately, a very narrow substrate scope, in combination with carbon tetrachloride being used as a solvent, restricts the application of this procedure significantly [43,44]. While the copper-catalyzed sequential diarylation of 2-mercaptotriazole, **W**, with 1-bromo-2-iodobenzene, **V**, in Scheme 1F was also shown to be possible, this reaction gave heterocycle, **M**, in only 14% yield [45].

A third approach leading to the benzo[4,5]thiazolo[2,3-*c*][1,2,4]triazole ring system is based on forming the thiazole ring in the last reaction step by a bond formation between the sulfur of the 2-mercaptophenyl substituent of a triazole and the unfunctionalized triazole carbon (Scheme 1G,H). The Straub group has observed two of their thiol-containing triazolium salts (**X**) undergo an oxidative cyclization in DMSO (Scheme 1G), thereby forming charged *N*-substituted heteroaromatic compounds, **Y** [46,47]. As opposed to the acidic triazolium starting materials of the Straub group, we demonstrate in this report that a similar bond formation can be employed to obtain neutral benzo[4,5]thiazolo[2,3-*c*][1,2,4]triazoles containing a wide variety of functional groups on the benzene ring from non-acidic triazoles (Scheme 1H). We were aiming to include both electron-donating and electron-withdrawing groups, halides, amines, alcohols, carboxylic acids, and other synthetically valuable substituents. As shown in Scheme 1H, we developed a two-step process for the conversion of a 4-(2-((4-methoxybenzyl)thio)phenyl)-4*H*-1,2,4-triazole species (**Z**) into the target tricyclic compound (**AB**).After the selective removal of the *p*-methoxybenzyl protecting group in the first step, the resulting free thiol (**AA**) is subsequently oxidized with DMSO to a disulfide intermediate (not shown in this scheme), which, upon deprotonation of the triazole carbon, undergoes an intramolecular ring closure. Herein, we present both the preparation of the starting triazoles (**Z**) from commercially available precursors, as well as their conversion to the benzo[4,5]thiazolo[2,3-*c*][1,2,4]triazole derivative, **AB**.

## 2. Results and Discussion

### 2.1. Preparation of Triazole Precursors

**Scheme 2:**
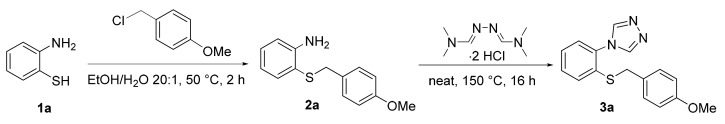
Synthesis of triazole, **3a**.

Triazole, **3a**, was prepared according to the literature procedure shown in Scheme 2 [46,48]. While the *p*-methoxybenzyl-protected aniline, **2a**, could be obtained in this case by protecting 2-mercaptoaniline, **1a**, under argon atmosphere, the lack of commercial availability of substituted 2-mercaptoanilines required the development of a different synthesis route. An obvious method for introducing a sulfur substituent on an aromatic ring would be a classical nucleophilic aromatic substitution using a 2-halonitrobenzene derivative starting material [49,50,51]. Subsequent reduction of the nitro group would afford the corresponding aniline derivative.

**Scheme 3:**
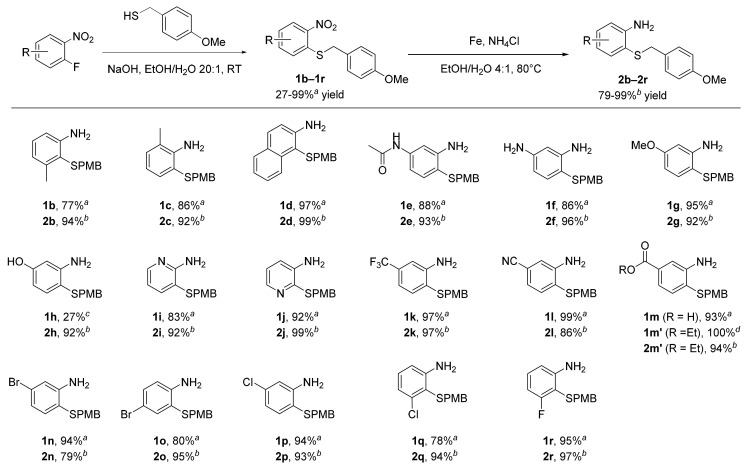
Preparation of protected 2-mercaptoanilines, **2b**–**2r**. *^a^*Isolated yields of *p*-methoxybenzyl-protected 2-mercaptonitrobenzene derivatives, **1b**–**1r**. *^b^*Isolated yields of protected 2-mercaptoanilines, **2b**–**2r**. *^c^*Prepared from *tert*-butyl(4-fluoro-3-nitrophenoxy)dimethylsilane. *^d^*Prepared from **1m**.

This alternative route to the *p*-methoxybenzyl-protected 2-mercaptoanilines, **2b**–**2r**, starting from 1-fluoro-2-nitrobenzene derivatives, is shown in Scheme 3. By using (4-methoxyphenyl)methanethiol as a reagent in the nucleophilic aromatic substitution, we were able to obtain the *p*-methoxybenzyl-protected 2-mercaptonitrobenzene derivatives, **1b**–**1r**, in excellent yields (77–99%) in one step. An exception was **1h**, which was prepared from *tert*-butyl(4-fluoro-3-nitrophenoxy)dimethylsilane in 27% yield. The silyl-protecting group was required to prevent self-condensation of the starting material under basic conditions and is cleaved during the nucleophilic aromatic substitution due to the generation of fluoride, thus forming the free phenol **1h**. The ester species **1m′** was obtained quantitatively from the carboxylic acid **1m**. Reduction of the nitrobenzene derivatives **1b**–**1r** in the presence of excess iron powder and ammonium chloride gave the corresponding amines, **2b**–**2r**, in excellent yields (79–99%).

**Scheme 4:**
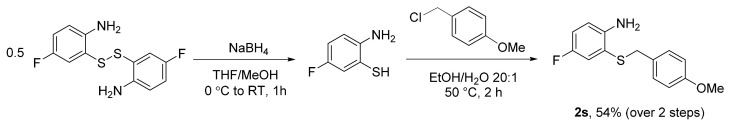
Synthesis of **2s**.

Compound **2s** was not available via a nucleophilic substitution followed by a reduction due to the reactivity of the second fluorine substituent. Instead, we prepared **2s** in two steps from 2,2′-disulfanediylbis(4-fluoroaniline) by first reducing it with NaBH_4_ to free the thiol that was subsequently protected with the *p*-methoxybenzyl group (Scheme 4). The synthesis of 2,2′-disulfanediylbis(4-fluoroaniline) can be found in the experimental procedures (Section 3.6.1).

**Scheme 5:**
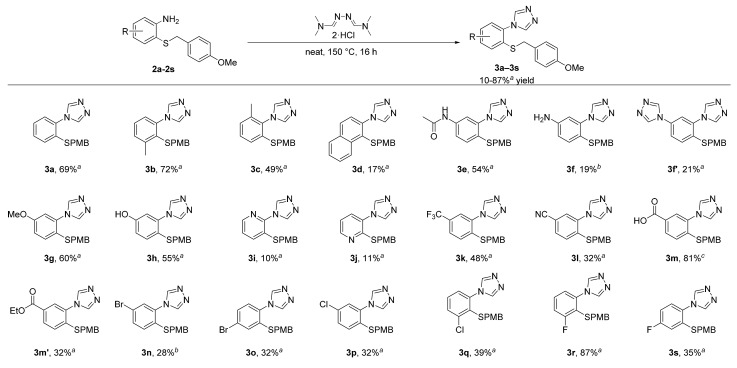
Preparation of triazole precursors **3a**–**3s**. *^a^*All yields shown here are isolated yields. *^b^* Obtained as a by-product from the synthesis of **3f′**. *^c^* Synthesized from **3m′**.

In analogy to **3a**, which was prepared in 69% yield following a literature procedure [46,49], triazoles **3b**–**3s** were obtained by heating the aniline derivatives **2b**–**2s** in the presence of *N,N*-dimethylformamide azine dihydrochloride at 150 °C for 16 h (Scheme 5). While the carboxylic acid **3m** can be formed using the procedure described above, we were unable to separate it from the salts and eventually synthesized it in 81% yield by hydrolysis of the ester **3m′**. The yields of the triazoles obtained using this method were extremely variable (10–72%), as can be seen in Scheme 4, and certainly could use improvement. The low yields depended on the exact structure of the aniline. In many cases, the starting amine could be isolated back. However, leaving the reaction for a longer duration did not increase the amount of product isolated, which suggests that the *N,N*-dimethylformamide azine dihydrochloride may degrade over time. In some cases, the starting amine was not detected at the end of a low-yielding reaction, and we believe that it may undergo decomposition due to the high reaction temperature. Loss of the *p-*methoxybenzyl protecting group was observed in all reactions, generally resulting in the formation of trace amounts of the target benzo[4,5]thiazolo[2,3-*c*][1,2,4]triazoles as byproducts of the triazole formation. However, this reaction was found to be the most dominant transformation in the preparation of **3d**, which was obtained in only 17% yield. In addition, dehalogenation further lowered the yields of the bromo- and chloro-derivatives **3n**–**3q**, whereas deamination was the major cause for the low isolated yields of the pyridine derivatives **3i** and **3j**. While we are currently investigating higher yielding routes to triazoles **3a**–**3s**, we were able to isolate sufficient material to carry out a thorough investigation of the new oxidative cyclization reaction leading to the benzo[4,5]thiazolo[2,3-*c*][1,2,4]triazole derivatives.

### 2.2. Reaction Optimization

**Scheme 6:**
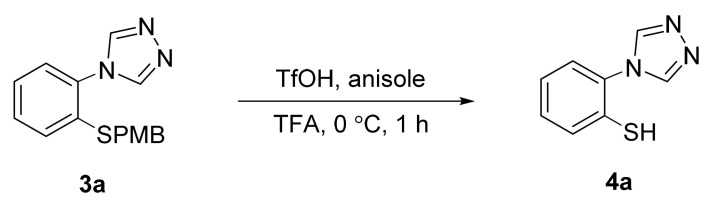
Synthesis of test compound **4a**.

Complete deprotection of the thiol group of triazole **3a** was achieved within 1 h by treatment with triflic acid and anisole in TFA under an argon atmosphere at 0 °C, thus forming the free thiol **4a** (Scheme 6).

Compound **4a** served as our model substrate for optimization of the reaction conditions leading to our tricyclic heteroaromatic molecule, **6a** (Table 1). Based on previous studies in our group, we rationalized that an oxidative cyclization should be possible via a symmetrical disulfide intermediate, **5a**. Therefore, standard oxidation conditions for the formation of a disulfide bond were explored for this transformation, and the reaction was followed using LCMS.

The use of stoichiometric hydrogen peroxide in aqueous ethanol, a widely used oxidant for disulfide coupling, led to the disulfide **5a** at room temperature (Entry 1). Increasing the temperature to 80 °C under otherwise identical conditions resulted in complete conversion to the desired product, **6a,** within 16 h, which could be isolated in 78% yield (Entry 2). In an attempt to reduce the reaction time, we used an excess of hydrogen peroxide in ethanol; however, neither the reaction time nor the isolated yield showed any improvements (Entry 3). In addition, hydrogen peroxide was used in 10% aqueous NaOH. In this case, the desired product was not obtained at all since the reaction stopped at the disulfide stage (Entry 4). We decided to change our solvent to DMSO. Using DMSO as both the oxidant and solvent at room temperature, gave only the disulfide **5a**, even after 16 h (Entry 5), and no cyclized product was observed. After increasing the temperature to 100 °C, we observed full conversion to compound **6a** in 4 h and were able to isolate the heterocycle in 83% yield (Entry 6). In an attempt to reduce the reaction temperature, one equivalent of iodine was added to the DMSO solution, but this did not change the outcome of the reaction; at room temperature, the disulfide **5a** was obtained as the sole product (Entry 7), while, at 100 °C, the reaction time for complete conversion to **6a** was still 4 h, with a comparable isolated yield of 82% (Entry 8). With these results in hand, we decided that carrying out the reaction in DMSO at 100 °C (Entry 6) without an additional oxidant were the best reaction conditions to convert thiol **4a** to the tricyclic heteroaromatic species **6a**.

### 2.3. Oxidative Cyclization Reaction leading to benzo[4,5]thiazolo[2,3-*c*][1,2,4]triazole Derivatives

**Scheme 7:**
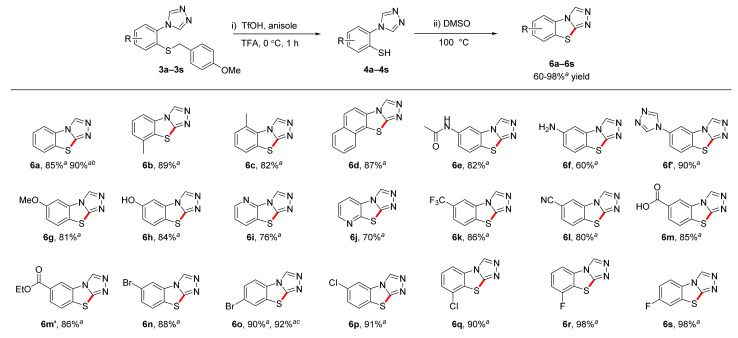
Formation of benzo[4,5]thiazolo[2,3-*c*][1,2,4]triazole derivatives **6a**–**6s** in two steps from **3a**–**3s**. *^a^*Isolated yields for reactions carried out on a 0.25–1 mmol scale, *^b^*5 mmol scale, and *^c^*10 mmol scale.

We found that, by using the optimized conditions developed above, **3a** could be converted to the tricyclic heteroaromatic compound **6a** in one step without isolating the free thiol **4a**. However, we generally carried out a brief aqueous extraction after the deprotection in order to remove residual acid and characterized the free thiol by LCMS. As shown in Scheme 7, triazoles **3a**–**3s** were subjected to the deprotection conditions to give the corresponding free thiols **4a**–**4s**, which were used without purification in the next step after removal of the solvent under high vacuum, followed by an aqueous extraction. During the oxidative cyclization step carried out in DMSO, we observed the free thiols **4a**–**4s** to rapidly undergo oxidative disulfide coupling, leading to disulfides **5a**–**5s**. At elevated temperatures, the disulfide intermediates were found to undergo ring closure to give the desired compounds **6a**–**6s** in 100% conversions by LCMS within less than 4 h. Regardless of whether the substituents on the aryl ring were electron-donating or electron-withdrawing, the tricyclic heteroaromatic compounds **6a**–**6s** were obtained in good to excellent yields. For example, weakly electron-donating groups, such as methyl (compounds **6b** and **6c**) as well as the naphthalene derivative (**6d**) were obtained in 82–89% yields. Various nitrogen substituents were tolerated (compounds **6e**, **6f**, and **6f′**): While the acetyl-protected amine **6e** was isolated in good yield (82%), a lower yield was observed for the unprotected primary amine **6f** (60%). Compound **6f′**, with its triazole substituent, was, on the other hand, isolated in excellent yield (94%). Since complete conversions were observed by LCMS in all cases and no other byproducts were detected, the lower yield of **6f** is thought to be a result of its water solubility. Both **6e** and **6f′** are not only insoluble in water but also showed poor solubility in organic solvents, such as methylene chloride, methanol, and even DMSO. The two examples of oxygen-containing substituents at position 6 (**6g** and **6h**) also gave good yields (81% and 85%, respectively). Pyridine derivatives (**6i** and **6j**) were found to tolerate our reaction conditions; however, the products were obtained in lower yields (75% and 70% respectively). Strongly electron-withdrawing groups, such as a trifluoromethyl and a carbonitrile substituent in the 6-position, gave the tricyclic compounds **6k** and **6l** in good yields (86% and 80%, respectively). Similarly, the carboxylic acid **6m** and ester **6m′** derivatives were synthesized in 85% and 86% yields, respectively. Additionally, all halogen-containing derivatives **6n-6s** were isolated in excellent yields, ranging from 88% to 98%. It is noteworthy that the reaction time for both compounds with halogens at position 7 (**6o** and **6s**) was 30 min shorter than the time required for the cyclization of any of the other compounds under identical conditions.

While most of the reactions in Scheme 7 were carried out on a 0.5 mmol or 1 mmol scale, smaller-scale reactions of 0.25 mmol (**3g**, **3i**, and **3j**) still allowed the isolation of the products **6g**, **6i**, and **6j** in acceptable yields. However, as demonstrated with **3a** and **3o**, increasing the reaction scale to 5 mmol and above generally afforded the products **6a** and **6o** in excellent yields of over 90%. On a 5 mmol scale, heterocycle **6a** was obtained in 90% yield compared to 85% on a 1 mmol scale. Likewise, increasing the scale from 1 mmol to 10 mmol for the preparation of **6o** led to a small increase in yield from 90% to 92%.

### 2.4. Basic Mechanistic Investigations

**Figure 2:**
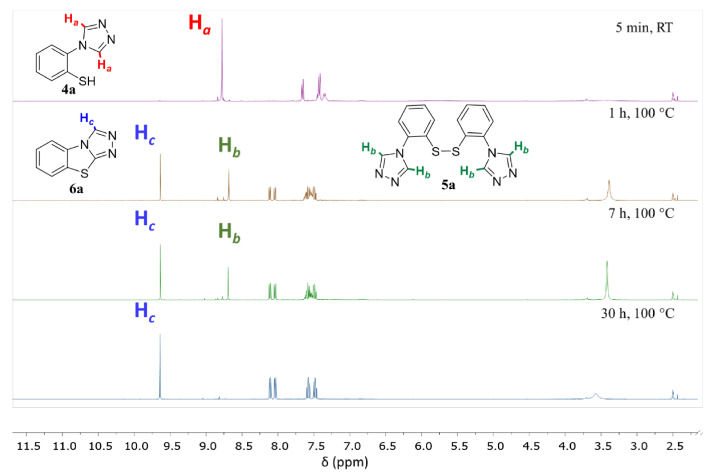
Conversion of thiol **4a** in DMSO-*d_6_* to benzothiazolotriazole **6a** via disulfide **5a**.

We were interested in following the conversion of thiol **4a** to compound **6a** by NMR to confirm the disulfide intermediate **5a** detected previously by LCMS. Therefore, compound **4a** was dissolved in DMSO-*d_6_* and an ^1^H NMR was recorded immediately after the addition of the solvent. This NMR showed the presence of a singlet at 8.78 ppm (**H*_a_***), which was assigned to the triazole C-H of the free thiol **4a** (Figure 2, 5 min RT). The NMR reaction tube was then heated to 100 °C, and a second ^1^H NMR was measured after 1 h, which showed the presence of two new singlets at 9.64 (**H*_c_***) and 8.70 ppm (**H*_b_***). The singlet at 9.64 ppm corresponded to **H*_c_*** of heterocycle **6a**, while the singlet at 8.70 ppm was assigned to **H*_b_*** of the disulfide intermediate **5a**. LCMS of the NMR solution confirmed the presence of these two molecules, while no free thiol was detected. After 30 h, the disulfide **5a** was consumed completely, and only the compound **6a** was observed by both NMR and LCMS.

**Figure 3:**
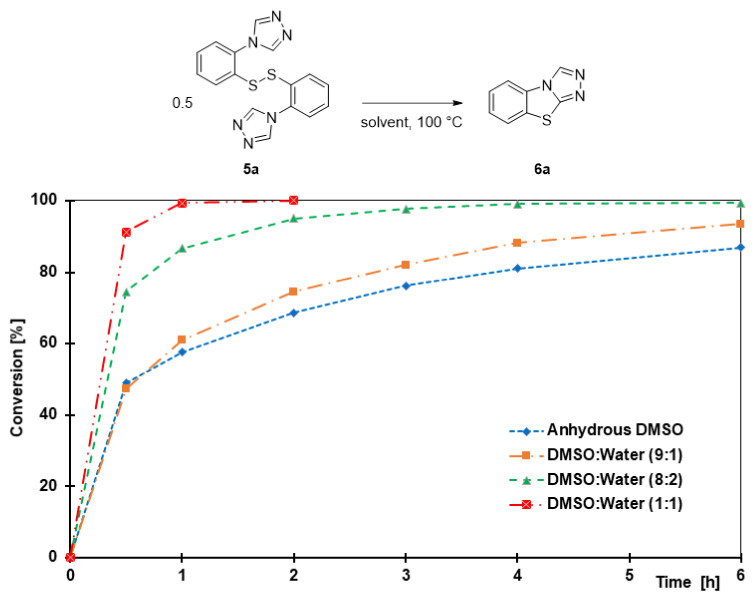
Effect of water on the conversion of disulfide **5a** to heterocycle **6a**.

Interestingly, the reaction carried out in dried deuterated DMSO took longer to reach completion than in reagent grade DMSO, which suggested that water might play a role in the reaction mechanism. While the conversion of each thiol to the heterocycle is expected to generate one equivalent of dimethyl sulfide and one equivalent of water if carried out in DMSO, we were particularly interested to see how the rate of the cyclization step was affected by the amount of water present. To study this effect of water, the disulfide **5a** was isolated and heated at 100 °C in DMSO containing known amounts of water (Figure 3). Samples taken at regular intervals were immediately analyzed by LCMS, which, after calibration, allowed the quantification of the species present. Indeed, as expected, we observed that increases in the water content corresponded to increases in the rate of conversion of the disulfide **5a** to product **6a**. A possible explanation for the rate-enhancing effect of water could be that it is involved in the C-H bond functionalization of the triazole C-H bond. Although it cannot be excluded that some residual acid is present from the deprotection of the thiol despite the aqueous extraction, we hypothesize that the role of water might be twofold. On the one hand, reversible protonation of a triazole nitrogen is expected to significantly increase the acidity of the C-H bond in the resulting triazolium species. However, water is also the strongest base present and is likely to be involved in the deprotonation of this triazolium species, thereby leading to a nucleophilic carbene intermediate, which could attack one of the sulfur atoms in the disulfide bond to afford our heterocyclic product **6a** and a thiolate. This thiolate leaving group would immediately undergo oxidative disulfide coupling, thus generating the next active disulfide intermediate.

**Figure 4:**
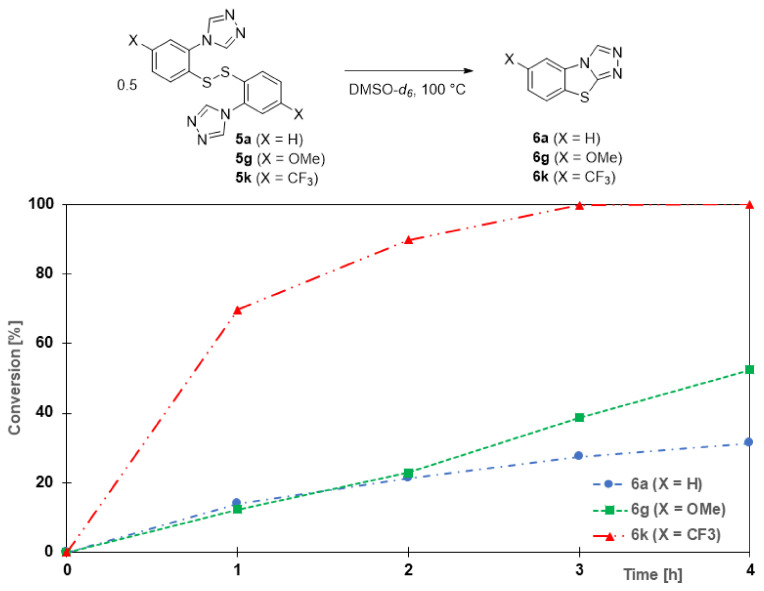
Effect of electron-donating and electron-withdrawing groups on the cyclization step.

Preliminary NMR studies into substituent effects on the reaction rate show that a very electron-withdrawing group, such as trifluoromethyl, in the *para*-position to the disulfide bond significantly increases the rate of conversion of this disulfide (**5k**) to the tricyclic heteroaromatic compound **6k** (Figure 4). This effect is in agreement with the aforementioned mechanism, as the sulfur atom in the disulfide would become significantly more electrophilic while simultaneously the hydrogen of the C-H bond of the triazole would increase in acidity. Meanwhile, disulfide **5g** (containing the electron-donating methoxy group) displayed a similar initial reaction rate as the unsubstituted disulfide **5a**. Full conversion to both the unsubstituted heterocycle **6a** and the methoxy derivative **6g** was only observed after 30 h.

Although our hypothetical mechanism provides both a potential explanation for the role of water and some of the substituent effects observed, we cannot exclude other mechanisms for the formation of the heterocycle in the absence of a more rigorous kinetic investigation.

## 3. Materials and Methods

### 3.1. General Information

Most reagents and solvents were purchased from Fisher Scientific (Waltham, MA, USA), Oakwood Chemical (Estill, SC, USA), TCI America (Portland, OR, USA), and Avantor (Radnor, PA, USA) and were used as supplied unless otherwise noted. Thermo Scientific™ silica gel (for column chromatography, 0.035–0.070 mm, 60Å) from Fisher Scientific (Waltham, MA, USA) was used for chromatographic separations. DMSO-*d_6_* was dried over molecular sieves.

### 3.2. Analyses

^1^H NMR, ^13^C{^1^H} NMR spectra, and ^19^F{^1^H} NMR spectra were all recorded using a 400 MHz Bruker Avance III spectrometer with a 5 mm liquid-state Smart Probe. Chemical shifts (δ_H_ and δ_C_) are expressed in parts per million (ppm) and reported relative to the resonance of the residual protons of the DMSO-*d_6_* (δ_H_ = 2.50 ppm), CD_3_CN (δ_H_ = 1.94 ppm), or CDCl_3_ (δ_H_ = 7.26 ppm) or in ^13^C{^1^H} NMR spectra relative to the resonance of the deuterated solvent DMSO-*d_6_* (δ_C_ = 39.52 ppm), CD_3_CN (δ_C_ = 1.32 ppm), or CDCl_3_ (δ_C_ = 77.16 ppm). Chemical shifts in the ^19^F{^1^H} NMR spectra are reported relative to the internal standard fluorobenzene (δ_F_ = −113.15). The coupling constants (*J*) are given in Hz. All measurements were carried out at 298 K. The abbreviations used in the description of the NMR data are as follows: s, singlet; d, duplet; t, triplet; m, multiplet; and sept., septet. Copies of the ^1^H NMR and ^13^C NMR spectra for compounds 1b–1r, 2b–2s, 3b–3s, and 6a–6s are provided in the Supporting Information. High-resolution mass spectrometry (HRMS) data were obtained on an LTQ Orbitrap Fusion in FT orbitrap mode at a resolution of 240,000.

### 3.3. Preparation of 2-Fluoronitrobenzene Starting Materials for **1e** and **1h**

#### 3.3.1. N-(4-Fluoro-3-nitrophenyl)acetamide

A 500 mL round-bottomed flask was charged with 4-fluoro-3-nitroaniline (30 mmol, 1 equiv.), acetyl chloride (1.1 equiv.), and THF (150 mL) and stirred overnight at room temperature. The solvent was removed under reduced pressure. Water was added to the residue and the crude product was extracted with ethyl acetate, dried over MgSO_4_, filtered, and evaporated. Purification of the crude product by column chromatography (silica gel, ethyl acetate/hexanes, 2:1, R_f_ = 0.50) afforded the product as a beige solid in 80% (4.753 g, 23.98 mmol) yield; m.p. 140–141 °C. ^1^H NMR (400 MHz, DMSO-*d*_6_, 298 K): δ = 10.37 (s, 1H), 8.49 (dd, *J* = 6.7 Hz, *J* = 2.6 Hz, 1H), 7.84–7.80 (m, 1H), 7.51 (dd, *J* = 11.1 Hz, *J* = 9.2 Hz, 1H), and 2.07 (s, 3H); ^13^C{^1^H} NMR (100 MHz, DMSO-*d*_6_, 298 K): δ = 168.9, 150.1 (d, *J*_C–F_ = 256.2 Hz), 136.5 (d, *J*_C–F_ = 8.0 Hz), 136.0 (d, *J*_C–F_ = 3.2 Hz), 126.1 (d, *J*_C–F_ = 8.1 Hz), 118.7 (d, *J*_C–F_ = 21.8 Hz), 115.2, and 23.9; ^19^F{^1^H} NMR (376 MHz, DMSO-*d*_6_, 298 K, referenced to C_6_H_5_F): *δ* = −125.81.

#### 3.3.2. *Tert*-Butyl(4-fluoro-3-Nitrophenoxy)dimethylsilane

A 500 mL round-bottomed flask was charged with 4-fluoro-3-nitrophenol (30 mmol, 1 equiv.), *tert-*butylchlorodimethylsilane (1.2 equiv.), imidazole (3 equiv.), and THF (60 mL) and stirred at room temperature for 2 h. The solvent was removed under reduced pressure. Water was added to the residue and the crude product was extracted with diethyl ether, dried over MgSO_4_, filtered and evaporated. Purification by column chromatography (silica gel, diethyl ether/hexanes 4:1) afforded the product as a red oil in 92% (8.718 g, 28.55 mmol) yield. ^1^H NMR (400 MHz, CDCl_3_, 298 K): δ = 7.45 (dd, *J* = 6.1 Hz, *J* = 3.0 Hz, 1H), 7.16–7.05 (m, 2H), 0.97 (s, 9H), and 0.21 (s, 6H); ^13^C{^1^H} NMR (100 MHz, CDCl_3_, 298 K): δ = 151.8 (d, *J*_C–F_ = 3.0 Hz), 150.4 (d, *J*_C–F_ = 256.7 Hz), 137.3 (d, *J*_C–F_ = 7.8 Hz), 127.2 (d, *J*_C–F_ = 7.6 Hz), 118.9 (d, *J*_C–F_ = 22.2 Hz), 116.6 (d, *J*_C–F_ = 5.5 Hz), 25.6, 18.2, and −4.5; ^19^F{^1^H} NMR (376 MHz, CDCl_3_, 298 K, referenced to C_6_H_5_F): *δ* = −127.55.

### 3.4. General Procedure 1 for the Synthesis of (4-Methoxybenzyl)(2-Nitrophenyl)sulfanes (**1b**–**1r**)

The following description is for a 30 mmol scale reaction. The solvent quantities and flask size were adjusted accordingly for smaller-scale reactions.

A 500 mL round-bottomed flask equipped with a stir bar was loaded with the 1-fluoro-2-nitrobenzene derivative (1 equiv.) and 200 mL of ethanol and placed under an atmosphere of argon. (4-methoxyphenyl)methanethiol (1 equiv.) was added with a syringe, followed by a dropwise addition of NaOH (1 equiv.) dissolved in 10 mL of H_2_O. The reaction mixture was stirred at room temperature until TLC indicated the completion of the reaction (typically within 2 h). After removing the solvent under reduced pressure, the residue was diluted with 150 mL of H_2_O and extracted twice with dichloromethane. The organic phases were combined, dried over MgSO_4_, filtered, and concentrated. The resulting crude product was purified by recrystallization or column chromatography as described below.

#### 3.4.1. (4-Methoxybenzyl)(2-Methyl-6-Nitrophenyl)sulfane (**1b**)

The title compound was prepared according to general procedure 1 on a 30 mmol scale. Recrystallization from diethyl ether/hexanes (1:1) afforded the product as an off-white powder in 77% (6.701 g, 23.18 mmol) yield; m.p. 63–64 °C. ^1^H NMR (400 MHz, DMSO-*d*_6_, 298 K): δ = 7.62 (dd, *J* = 7.4 Hz, *J* = 1.0 Hz, 1H), 7.55 (dd, *J* = 7.6 Hz, *J* = 0.8 Hz, 1H), 7.48 (t, *J* = 7.6 Hz, 1H), 7.02 (dt, *J* = 8.6 Hz, *J* = 2.9 Hz, 2H), 6.80 (dt, *J* = 8.4 Hz, *J* = 3.0 Hz, 1H), 3.95 (s, 2H), 3.71 (s, 3H), and 2.39 (s, 3H); ^13^C{^1^H} NMR (100 MHz, DMSO-*d*_6_, 298 K): δ = 158.6, 156.2, 145.6, 133.0, 130.0, 130.0, 128.8, 124.2, 120.5, 113.8, 55.0, and 20.6.

#### 3.4.2. (4-Methoxybenzyl)(3-Methyl-2-Nitrophenyl)sulfane (**1c**)

The title compound was prepared according to general procedure 1 on a 5 mmol scale. Recrystallization from diethyl ether afforded the product as a bright yellow powder in 86% (1.238 g, 4.28 mmol) yield; m.p. 90–92 °C. ^1^H NMR (400 MHz, DMSO-*d*_6_, 298 K): δ = 7.52 (d, *J* = 7.5 Hz, 1H), 7.44 (t, *J* = 7.7 Hz, 1H), 7.30 (d, *J* = 7.5 Hz, 1H), 7.22 (dt, *J* = 8.6 Hz, *J* = 2.8 Hz, 2H), 6.85 (dt, *J* = 8.7 Hz, *J* = 2.9 Hz, 2H), 4.23 (s, 2H), 3.71 (s, 3H), and 3.32 (s, 3H); ^13^C{^1^H} NMR (100 MHz, DMSO-*d*_6_, 298 K): δ = 158.5, 151.0, 130.7, 130.1 (2 signals), 129.4, 128.8, 128.5, 128.0, 113.9, 55.0, 37.0, and 17.0.

#### 3.4.3. (4-Methoxybenzyl)(2-Nitronaphthalen-1-yl)sulfane (**1d**)

The title compound was prepared according to general procedure 1 on a 5.21 mmol scale. Recrystallization from acetone/hexanes (1:4) afforded the product as a yellow powder in 97% (1.636 g, 5.03 mmol) yield; m.p. 68–70 °C. ^1^H NMR (400 MHz, DMSO-*d*_6_, 298 K): δ = 8.57 (d, *J* = 8.2 Hz, 1H), 8.21 (d, *J* = 8.8 Hz, 1H), 8.13 (dd, *J* = 7.8 Hz, *J* = 1.4 Hz, 1H), 7.85 (d, *J* = 8.8 Hz, 1H), 7.81–7.73 (m, 2H), 6.92 (dt, *J* = 8.6 Hz, *J* = 2.6 Hz, 2H), 6.72 (dt, *J* = 8.6 Hz, *J* = 2.6 Hz, 2H), 4.07 (s, 2H), and 3.67 (s, 3H); ^13^C{^1^H} NMR (100 MHz, DMSO-*d*_6_, 298 K): δ = 158.6, 153.7, 133.8, 133.6, 131.5, 129.9, 129.0, 128.5, 128.5, 127.3, 123.8, 119.4, 113.8, 55.0, and 40.5.

#### 3.4.4. *N*-(4-((4-Ethoxybenzyl)thio)-3-Nitrophenyl)acetamide (**1e**)

The title compound was prepared according to general procedure 1 from *N*-(4-fluoro-3-nitrophenyl)acetamide (see Section 3.3.1) on a 30 mmol scale. Extraction was carried out with ethyl acetate (instead of dichloromethane) and afforded the pure product as a bright yellow powder in 88% (6.150 g, 19.04 mmol) yield; m.p. 140–141 °C. ^1^H NMR (400 MHz, DMSO-*d*_6_, 298 K): δ = 10.35 (s, 1H), 8.55 (d, *J* = 2.2 Hz, 1H), 7.80 (dd, *J* = 8.8 Hz, *J* = 2.3 Hz, 1H), 7.67 (d, *J* = 8.8 Hz, 1H), 7.33 (d, *J* = 8.6 Hz, 2H), 6.89 (d, *J* = 8.6 Hz, 2H), 4.44 (s, 2H), 3.73 (s, 3H), and 2.07 (s, 3H); ^13^C{^1^H} NMR (100 MHz, DMSO-*d*_6_, 298 K): δ = 168.9, 158.6, 145.4, 136.8, 130.4, 129.6, 128.5, 127.4, 124.4, 114.9, 114.0, 55.0, 35.7, and 24.0.

#### 3.4.5. (2,4-. Dinitrophenyl)(4-Methoxybenzyl)sulfane (**1f**)

The title compound was prepared according to general procedure 1 on a 30 mmol scale. Recrystallization from diethyl ether afforded the product as a light brown powder in 86% (8.718 g, 25.84 mmol) yield; m.p. 113–114 °C. ^1^H NMR (400 MHz, DMSO-*d*_6_, 298 K): δ = 8.86 (d, *J* = 2.6 Hz, 2H), 8.44 (dt, *J* = 9.0 Hz, *J* = 2.6 Hz, 1H), 7.96 (d, *J* = 9.1 Hz, 1H), 7.40 (dt, *J* = 8.7 Hz, *J* = 2.9 Hz, 2H), 6.92 (dt, *J* = 8.7 Hz, *J* = 3.0 Hz, 1H), 4.44 (s, 2H), and 3.74 (s, 3H); ^13^C{^1^H} NMR (100 MHz, DMSO-*d*_6_, 298 K): δ = 158.9, 145.7, 144.0, 143.6, 130.6, 128.4, 127.4, 126.1, 121.2, 114.2, 55.1, and 35.8.

#### 3.4.6. (4-. Methoxy-2-Nitrophenyl)(4-Methoxybenzyl)sulfane (**1g**)

The title compound was prepared according to general procedure 1 on a 30 mmol scale. Recrystallization from acetone/hexanes (1:4) afforded the product as a bright orange powder in 95% (8.718 g, 28.55 mmol) yield; m.p. 116–118 °C. ^1^H NMR (400 MHz, DMSO-*d*_6_, 298 K): δ = 7.65 (d, *J* = 2.8 Hz, 1H), 7.62 (d, *J* = 9.0 Hz, 1H), 7.33–7.28 (m, 3 H), 6.87 (dt, *J* = 8.6 Hz, *J* = 2.8 Hz, 2H), 4.22 (s, 2 H), 3.83 (s, 3H), and 3.72 (s, 3H); ^13^C{^1^H} NMR (100 MHz, DMSO-*d*_6_, 298 K): δ = 158.6, 157.0, 147.1, 130.3, 130.0, 127.6, 126.1, 121.2, 113.9, 109.5, 56.0, 55.0, and 36.2.

#### 3.4.7. 4-((4-Methoxybenzyl)thio)-3-Nitrophenol (**1h**)

The title compound was prepared according to general procedure 1 from *tert-*butyl(4-fluoro-3-nitrophenoxy)dimethylsilane (see 3.3.2) on a 26.5 mmol scale. Purification by column chromatography (silica gel, 1. diethyl ether/hexanes (1:3), and 2. diethyl ether/hexanes (1:2), R_f_ = 0.21) afforded the product as a bright yellow powder in 27% (8.718 g, 7.06 mmol) yield; m.p. 115–116 °C. ^1^H NMR (400 MHz, DMSO-*d*_6_, 298 K): δ = 10.35 (s, 1H), 7.53 (d, *J* = 8.8 Hz, 1H), 7.46 (d, *J* = 2.7 Hz, 1H), 7.28 (d, *J* = 8.6 Hz, 2H), 7.13 (dd, *J* = 8.8 Hz, *J* = 2.7 Hz, 1H), 8.87 (d, *J* = 8.6 Hz, 2H), 4.18 (s, 2H), and 3.73 (s, 3H); ^13^C{^1^H} NMR (100 MHz, DMSO-*d*_6_, 298 K): δ = 158.5, 155.5, 147.3, 130.4, 130.3, 127.8, 123.8, 122.0, 113.9, 111.2, 55.0, and 36.4.

#### 3.4.8. 3-((4-Methoxybenzyl)thio)-2-Nitropyridine (**1i**)

The title compound was prepared according to general procedure 1 on a 30 mmol scale. Recrystallization from acetone/hexanes (1:4) afforded the product as a bright powder in 83% (6.889 g, 24.75 mmol) yield; m.p. 140–142 °C. ^1^H NMR (400 MHz, DMSO-*d*_6_, 298 K): δ = 8.39 (d, *J* = 3.6 Hz, 1H), 8.30 (d, *J* = 7.8 Hz, 1H), 7.77 (d, *J* = 4.4 Hz, 1H), 7.34 (d, *J* = 8.5 Hz, 2H), 6.89 (d, *J* = 8.5 Hz, 2H), 4.34 (s, 2H), and 3.72 (s, 3H); ^13^C{^1^H} NMR (100 MHz, DMSO-*d*_6_, 298 K): δ = 158.7, 153.5, 144.4, 138.8, 131.3, 130.4, 128.7, 126.9, 114.1, 55.1, and 35.4.

#### 3.4.9. 2-((4-Methoxybenzyl)thio)-3-Nitropyridine (**1j**)

The title compound was prepared according to general procedure 1 on a 27.9 mmol scale. Trituration with hexanes afforded the product as a yellow powder in 92% (7.629 g, 27.61 mmol) yield; m.p. 88–90 °C. ^1^H NMR (400 MHz, DMSO-*d*_6_, 298 K): δ = 8.86 (dd, *J* = 4.6 Hz, *J* = 1.5 Hz, 2H), 8.60 (dd, *J* = 8.3 Hz, *J* = 1.5 Hz, 1H), 7.46 (dd, *J* = 8.3 Hz, *J* = 4.6 Hz, 1H), 7.35 (d, *J* = 8.6 Hz, 2H), 6.87 (d, *J* = 8.6 Hz, 2H), 4.42 (s, 2H), and 3.72 (s, 3H); ^13^C{^1^H} NMR (100 MHz, DMSO-*d*_6_, 298 K): δ = 158.5, 156.0, 153.8, 141.4, 134.4, 130.5, 128.4, 120.0, 113.9, 55.0, and 33.9.

#### 3.4.10. (4-. Methoxybenzyl)(2-Nitro-4-(Trifluoromethyl)phenyl)sulfane (**1k**)

The title compound was prepared according to general procedure 1 on a 30 mmol scale. Trituration with hexanes afforded the product as a bright yellow powder in 97% (9.967 g, 29.03 mmol) yield; m.p. 111–112 °C. ^1^H NMR (400 MHz, DMSO-*d*_6_, 298 K): δ = 8.45 (d, *J* = 1.1 Hz, 1H), 8.04 (dd, *J* = 8.6 Hz, *J* = 1.7 Hz, 1H), 7.94 (d, *J* = 8.6 Hz, 1H), 7.38 (d, *J* = 8.6 Hz, 2H), 6.91 (d, *J* = 8.6 Hz, 2H), 4.39 (s, 2H), and 3.74 (s, 3H); ^13^C{^1^H} NMR (100 MHz, DMSO-*d*_6_, 298 K): δ = 159.3, 145.2, 142.9, 131.0, 130.4 (q, *J*_C–F_ = 3.3 Hz), 129.2, 126.9, 125.7 (q, *J*_C–F_ = 33.6 Hz), 123.6 (q, *J*_C–F_ = 270.4 Hz), 123.4 (q, *J*_C–F_ = 4.0 Hz), 114.6, 55.5, and 36.1; ^19^F{^1^H} NMR (376 MHz, DMSO-*d*_6_, 298 K, referenced to C_6_H_5_F): *δ* = −61.3.

#### 3.4.11. 4-((4-Methoxybenzyl)thio)-3-Nitrobenzonitrile (**1l**)

The title compound was prepared according to general procedure 1 on a 30 mmol scale. Recrystallization from diethyl ether afforded the product as a bright yellow powder in 99% (8.934 g, 29.74 mmol) yield; m.p. 202–204 °C. ^1^H NMR (400 MHz, DMSO-*d*_6_, 298 K): δ = 8.70 (d, *J* = 1.8 Hz, 2H), 8.12 (dt, *J* = 8.5 Hz, *J* = 1.8 Hz, 1H), 7.89 (d, *J* = 8.6 Hz, 1H), 7.38 (dt, *J* = 8.6 Hz, *J* = 2.9 Hz, 2H), 6.92 (dt, *J* = 8.7 Hz, *J* = 2.9 Hz, 2H), 4.40 (s, 2H), and 3.74 (s, 3H); ^13^C{^1^H} NMR (100 MHz, DMSO-*d*_6_, 298 K): δ = 158.8, 144.7, 143.4, 136.2, 130.6, 130.0, 128.4, 126.3, 117.1, 114.2, 107.4, 55.1, and 35.6.

#### 3.4.12. 4-((4-Methoxybenzyl)thio)-3-Nitrobenzoic acid (**1m**)

The title compound was prepared according to procedure 1 on an 85 mmol scale. Recrystallization from acetone/hexanes (1:4) afforded the product as a bright yellow powder in 93% (25.374 g, 79.46 mmol) yield., m.p. 269–271 °C. ^1^H NMR (400 MHz, DMSO-*d*_6_, 298 K): δ = 13.55 (s, 1H), 8.61 (d, *J* = 1.9 Hz, 1H), 8.15 (dd, *J* = 8.5 Hz, *J* = 1.9 Hz, 1H), 7.86 (d, *J* = 8.6 Hz, 1H), 7.39 (d, *J* = 8.6 Hz, 2H), 6.92 (d, *J* = 8.7 Hz, 2H), 4.37 (s, 2H), and 3.74 (s, 3H); ^13^C{^1^H} NMR (100 MHz, DMSO-*d*_6_, 298 K): δ = 165.4, 158.8, 144.6, 142.5, 133.7, 130.6, 127.8, 127.5, 126.6, 126.4, 114.1, 55.1, and 35.6.

#### 3.4.13. Ethyl 4-((4-Methoxybenzyl)thio)-3-Nitrobenzoate (**1m**′)

A 250 mL round-bottomed flask was charged with compound **1m** (23 mmol, 1 equiv.), oxalyl chloride (3 equiv.), and dichloromethane (50 mL). After adding DMF (3 drops), the reaction mixture was stirred for 1 h at 0 °C in an ice bath. EtOH (1.3 equiv.), triethylamine (1.3 equiv), and dichloromethane (50 mL) were added at 0 °C, the ice bath was removed, and the reaction flask was fitted with a reflux condenser. The reaction mixture was stirred at 40 °C for 2 h, then cooled to room temperature. After washing the dichloromethane solution with water, the organic phase was dried over MgSO_4_, filtered, and evaporated. Recrystallization of the crude product from diethyl ether/hexanes (1:2) afforded the product as a bright yellow solid in 99.99% (7.989 g, 22.998 mmol) yield; m.p. 149–150 °C. ^1^H NMR (400 MHz, DMSO-*d*_6_, 298 K): δ = 8.69 (d, *J* = 1.9 Hz, 1H), 8.16 (dd, *J* = 8.5 Hz, *J* = 1.9 Hz, 1H), 7.89 (d, *J* = 8.6 Hz, 1H), 7.39 (d, *J* = 8.6 Hz, 2H), 6.92 (d, *J* = 8.7 Hz, 2H), 4.39–4.33 (m, 4H), 3.74 (s, 3H), and 1.34 (t, *J* = 7.1 Hz, 3H); ^13^C{^1^H} NMR (100 MHz, DMSO-*d*_6_, 298 K): δ = 164.1, 158.9, 144.7, 143.2, 133.6, 130.8, 128.0, 126.6, 126.5, 126.4, 114.3, 61.7, 55.2, 35.8, and 14.2.

#### 3.4.14. (4-. Bromo-2-Nitrophenyl)(4-Methoxybenzyl)sulfane (**1n**)

The title compound was prepared according to general procedure 1 on a 30 mmol scale. Recrystallization from diethyl ether/hexanes (1:2) afforded the product as a bright yellow powder in 94% (9.968 g, 28.14mmol) yield; m.p. 121–124 °C. ^1^H NMR (400 MHz, DMSO-*d*_6_, 298 K): δ = 8.33 (d, *J* = 2.2 Hz, 1H), 7.89 (dd, *J* = 8.7 Hz, *J* = 2.2 Hz, 1H), 7.67 (d, *J* = 8.8 Hz, 1H), 7.34 (dt, *J* = 8.7 Hz, *J* = 2.8 Hz, 2H), 6.90 (dt, *J* = 8.7 Hz, *J* = 3.0 Hz, 2H), 4.31 (s, 2H), and 3.73 (s, 3H); ^13^C{^1^H} NMR (100 MHz, DMSO-*d*_6_, 298 K): δ = 158.7, 145.9, 136.6, 136.0, 130.4, 129.6, 128.0, 126.8, 117.0, 114.1, 55.1, and 35.6.

#### 3.4.15. (5-. Bromo-2-nitrophenyl)(4-methoxybenzyl)sulfane (**1o**)

The title compound was prepared according to general procedure 1 on a 30 mmol scale. Recrystallization from diethyl ether/hexanes (1:5) afforded the product as a bright yellow powder in 80% (8.501 g, 24.00 mmol) yield; m.p. 139–140 °C. ^1^H NMR (400 MHz, DMSO-*d*_6_, 298 K): δ = 8.10 (d, *J* = 8.8 Hz, 1H), 7.85 (d, *J* = 2.0 Hz, 1H), 7.57 (dd, *J* = 8.8 Hz, *J* = 2.0 Hz, 1H), 7.35 (dt, *J* = 8.7 Hz, *J* = 2.8 Hz, 2H), 7.91 (dt, *J* = 8.7 Hz, *J* = 3.0 Hz, 2H), 4.36 (s, 2H), and 3.74 (s, 3H); ^13^C{^1^H} NMR (100 MHz, DMSO-*d*_6_, 298 K): δ = 158.7, 144.1, 139.2, 130.5, 129.6, 128.7, 128.1, 127.5, 126.7, 114.1, 55.1, and 35.6.

#### 3.4.16. (4-. Chloro-2-Nitrophenyl)(4-Methoxybenzyl)sulfane (**1p**)

The title compound was prepared according to general procedure 1 on a 30 mmol scale. Recrystallization from diethyl ether/hexanes (1:2) afforded the product as an orange powder in 94% (8.258 g, 28.23 mmol) yield; m.p. 113–114 °C. ^1^H NMR (400 MHz, DMSO-*d*_6_, 298 K): δ = 8.23 (d, *J* = 2.2 Hz, 1H), 7.78 (dd, *J* = 8.7 Hz, *J* = 2.2 Hz, 1H), 7.74 (d, *J* = 8.8 Hz, 1H), 7.35 (dt, *J* = 8.6 Hz, *J* = 3.0 Hz, 2H), 6.90 (dt, *J* = 8.7 Hz, *J* = 3.0 Hz, 2H), 4.31 (s, 2H), and 3.73 (s, 3H); ^13^C{^1^H} NMR (100 MHz, DMSO-*d*_6_, 298 K): δ = 158.7, 145.8, 135.6, 133.8, 130.4, 129.4, 126.9, 125.3, 114.1, 55.1, and 35.6.

#### 3.4.17. (2-. Chloro-6-Nitrophenyl)(4-Methoxybenzyl)sulfane (**1q**)

The title compound was prepared according to general procedure 1 on a 30 mmol scale. Recrystallization from diethyl ether/hexanes (1:1) afforded the product as a bright yellow powder in 78% (7.290 g, 23.53 mmol) yield; m.p. 86–88 °C. ^1^H NMR (400 MHz, DMSO-*d*_6_, 298 K): δ = 7.84 (dd, *J* = 8.1 Hz, *J* = 1.2 Hz, 1H), 7.76 (dd, *J* = 8.0 Hz, *J* = 1.1 Hz, 1H), 7.58 (t, *J* = 8.1 Hz, 1H), 7.02 (dt, *J* = 8.6 Hz, *J* = 2.7 Hz, 2H), 6.78 (dt, *J* = 8.6 Hz, *J* = 2.6 Hz, 2H), 4.09 (s, 2H), and 3.69 (s, 3H); ^13^C{^1^H} NMR (100 MHz, DMSO-*d*_6_, 298 K): δ = 158.6, 156.1, 140.5, 132.9, 131.4, 130.0, 128.1, 125.0, 121.9, 113.8, 55.0, and 38.6.

#### 3.4.18. (2-. Fluoro-6-Nitrophenyl)(4-Methoxybenzyl)sulfane (1r)

The title compound was prepared according to general procedure 1 on a 15 mmol scale. Recrystallization from acetone/hexanes (1:4) afforded the product as a bright yellow powder in 95% (4.167 g, 14.20 mmol) yield; m.p. 105–107 °C. ^1^H NMR (400 MHz, DMSO-*d*_6_, 298 K): δ = 7.74–7.71 (m, 1H), 7.63–7.56 (m, 2H), 7.08 (dt, *J* = 8.6 Hz, *J* = 2.0 Hz, 2H), 6.78 (dt, *J* = 8.7 Hz, *J* = 2.0 Hz, 2H), 4.14 (s, 2H), and 3.69 (s, 3H); ^13^C{^1^H} NMR (100 MHz, DMSO-*d*_6_, 298 K): δ = 162.0 (d, *J*_C–F_ = 246.7 Hz), 158.6, 153.5 (d, *J*_C–F_ = 3.0 Hz), 130.7 (d, *J*_C–F_ = 9.4 Hz), 130.0, 128.4, 120.0 (d, *J*_C–F_ = 5.9 Hz), 119.9 (d, *J*_C–F_ = 14.8 Hz), 116.6 (d, *J*_C–F_ = 23.2 Hz), 113.8, 55.0, and 38.0 (d, *J*_C–F_ = 7.0 Hz); ^19^F{^1^H} NMR (376 MHz, DMSO-*d*_6_, 298 K, referenced to C_6_H_5_F): *δ* = −102.14

### 3.5. General Procedure 2 for the Synthesis of Substituted Anilines (**2b**–**2r**)

The following description is for a 20–25 mmol scale reaction. The solvent quantities and flask size were adjusted accordingly for smaller-scale reactions.

A 250 mL round-bottomed flask equipped with a stir bar was loaded with the (4-methoxybenzyl)(2-nitrophenyl)sulfane derivative (**1b**–**1r**, 1 equiv.), iron power (5.0 equiv.), NH_4_Cl (5.0 equiv.), and 150 mL of EtOH/H_2_O (4:1). The reaction flask was placed under inert atmosphere (argon), fitted with a reflux condenser, and stirred at 80 °C until TLC indicated a complete reduction (typically between 1 and 4 h). After cooling to room temperature, the reaction mixture was filtered through celite and concentrated under reduced pressure. The residue was then basified with 1M NaOH and extracted twice with dichloromethane, dried over MgSO_4_, and evaporated. The crystalline solid was washed with hexanes or diethyl ether as described below.

#### 3.5.1. 2-((4-Methoxybenzyl)thio)-3-Methylaniline (**2b**)

The title compound was prepared according to general procedure 2 on a 20.0 mmol scale. The crystalline light brown solid was washed with hexanes, which gave the desired product in 94% (4.897 g, 18.88 mmol) yield; m.p. 74–76 °C. ^1^H NMR (400 MHz, DMSO-*d*_6_, 298 K): δ = 7.06 (d, *J* = 8.3 Hz, 2H), 6.92 (t, *J* = 7.6 Hz, 1H), 7.76 (d, *J* = 8.3 Hz, 2H), 6.60 (d, *J* = 7.9 Hz, 1H), 6.41 (d, *J* = 7.2 Hz, 1H), 5.39 (s, 2H), 3.74 (s, 2H), 3.70 (s, 3H), and 2.13 (s, 3H); ^13^C{^1^H} NMR (100 MHz, DMSO-*d*_6_, 298 K): δ = 158.2, 150.5, 143.0, 130.0, 129.9, 129.2, 117.7, 114.8, 113.5, 112.0, 55.0, 36.4, and 21.4. HRMS (ESI) *m*/*z* calculated for [M + H]^+^ = [C_15_H_18_NOS]^+^ 260.1104; observed, 260.1105.

#### 3.5.2. 2-((4-Methoxybenzyl)thio)-6-Methylaniline (**2c**)

The title compound was prepared according to general procedure 2 on a 2.00 mmol scale. The crystalline white solid was washed with hexanes, which gave the desired product in 92% (0.474 g, 1.83 mmol) yield; m.p. 92–93 °C. ^1^H NMR (400 MHz, DMSO-*d*_6_, 298 K): δ = 7.14 (d, *J* = 8.1 Hz, 2H), 7.03 (d, *J* = 7.4 Hz, 1H), 6.94 (d, *J* = 7.0 Hz, 1H), 8.82 (d, *J* = 7.0 Hz, 2H), 6.44 (t, *J* = 7.4 Hz, 1H), 5.01 (s, 2H), 3.89 (s, 2H), 3.71 (s, 3H), and 2.11 (s, 3H); ^13^C{^1^H} NMR (100 MHz, DMSO-*d*_6_, 298 K): δ = 158.2, 146.9, 132.5, 130.4, 130.0, 129.9, 121.7, 116.2, 116.1, 113.6, 55.0, 37.5, and 18.2. HRMS (ESI) *m*/*z* calculated for [M + H]^+^ = [C_15_H_18_NOS]^+^ 260.1104 observed, 260.1104.

#### 3.5.3. 1-((4-Methoxybenzyl)thio)naphthalen-2-Amine (2d)

The title compound was prepared according to general procedure 2 on a 4.45 mmol scale. The yellow oil was washed with hexanes, which gave the desired product as a light brown solid in 99% (1.300 g, 4.41 mmol) yield; m.p. 58–60 °C. ^1^H NMR (400 MHz, DMSO-*d*_6_, 298 K): δ = 8.20 (d, *J* = 8.5 Hz, 2H), 7.66 (t, *J* = 8.4 Hz, 2H), 7.42–7.38 (m, 1H), 7.17–7.12 (m, 3H), 7.09 (d, *J* = 8.8 Hz, 1H), 6.78 (dt, *J* = 8.7 Hz, *J* = 2.1 Hz, 2H), 5.83 (s, 2H), 3.79 (s, 2H), and 3.69 (s, 3H); ^13^C{^1^H} NMR (100 MHz, DMSO-*d*_6_, 298 K): δ = 158.2, 149.5, 136.3, 130.3, 130.0 129.9, 128.3, 127.1, 126.9, 123.3, 121.1, 117.4, 113.6, 104.9, 55.0, and 37.3. HRMS (ESI) *m*/*z* calculated for [M + H]^+^ = [C_18_H_18_NOS]^+^ 296.1104; observed, 296.1104.

#### 3.5.4. N-(3-Amino-4-((4-Methoxybenzyl)thio)phenyl)acetamide (**2e**)

The title compound was prepared according to general procedure 2 on a 17.05 mmol scale. The crystalline beige powder solid was washed with hexanes, which gave the desired product in 93% (4.807 g, 15.91 mmol) yield; m.p. 111–112 °C. ^1^H NMR (400 MHz, DMSO-*d*_6_, 298 K): δ = 9.71 (s, 1H), 7.11 (t, *J* = 8.3 Hz, 3H), 6.97 (d, *J* = 8.2 Hz, 1H), 6.80 (d, *J* = 8.3 Hz, 2H), 6.62 (d, *J* = 7.8 Hz, 1H), 5.33 (s, 2H), 3.81 (s, 2H), 3.70 (s, 3H), and 2.01 (s, 3H); ^13^C{^1^H} NMR (100 MHz, DMSO-*d*_6_, 298 K): δ = 168.1, 158.1, 149.9, 140.6, 135.9, 130.2, 130.0, 113.6, 109.7, 107.7, 104.3, 55.0, 37.7, and 24.1. HRMS (ESI) *m*/*z* calculated for [M + H]^+^ = [C_16_H_19_N_2_O_2_S]^+^ 303.1162; observed, 303.1162.

#### 3.5.5. 4-((4-Methoxybenzyl)thio)benzene-1,3-Diamine (**2f**)

The title compound was prepared according to general procedure 2 on a 20.0 mmol scale. The crystalline beige solid was washed with hexanes, which gave the desired product in 96% (4.979 g, 19.12 mmol) yield; m.p. 91–92 °C. ^1^H NMR (400 MHz, DMSO-*d*_6_, 298 K): δ = 7.08 (d, *J* = 8.6 Hz, 2H), 6.80 (d, *J* = 8.6 Hz, 2H), 6.72 (d, *J* = 8.2 Hz, 1H), 5.93 (d, *J* = 7.9 Hz, 1H), 5.73 (dd, *J* = 8.2 Hz, *J* = 2.3 Hz, 1H), 5.00 (s, 2H), 4.97 (s, 2H), 3.71 (s, 3H), and 3.67 (s, 3H); ^13^C{^1^H} NMR (100 MHz, DMSO-*d*_6_, 298 K): δ = 158.0, 150.6, 150.5, 137.4, 130.6, 130.0, 113.5, 104.3, 102.0, 99.0, 55.0, and 38.8. HRMS (ESI) *m*/*z* calculated for [M + H]^+^ = [C_14_H_17_N_2_OS]^+^ 261.1056; observed, 261.1057.

#### 3.5.6. 5-Methoxy-2-((4-Methoxybenzyl)thio)aniline (**2g**)

The title compound was prepared according to general procedure 2 on a 27.9 mmol scale. The crystalline dark beige solid was washed with hexanes, which gave the desired product in 92% (7.082 g, 25.72 mmol) yield; m.p. 113–114 °C. ^1^H NMR (400 MHz, DMSO-*d*_6_, 298 K): δ = 7.10 (dt, *J* = 8.6 Hz, *J* = 2.0 Hz, 1H), 6.96 (d, *J* = 8.4 Hz, 2H), 7.76 (dt, *J* = 8.6 Hz, *J* = 2.0 Hz, 2H), 6.30 (d, *J* = 2.7 Hz, 1H), 6.05 (dd, *J* = 8.5 Hz, *J* = 2.7 Hz, 1H), 5.34 (s, 2H), 3.77 (s, 2H), 3.71 (s, 3H), and 3.65 (s, 3H); ^13^C{^1^H} NMR (100 MHz, DMSO-*d*_6_, 298 K): δ = 160.9, 158.1, 151.0, 137.2, 130.2, 130.0, 113.6, 107.1, 102.9, 98.9, 55.0, 54.7, and 38.0. HRMS (ESI) *m*/*z* calculated for [M + H]^+^ = [C_15_H_18_NO_2_S]^+^ 276.1053; observed, 276.1052.

#### 3.5.7. 3-Amino-4-((4-Methoxybenzyl)thio)phenol (**2h**)

The title compound was prepared according to general procedure 2 on a 2.75 mmol scale. The crystalline light brown powder was washed with diethyl ether/hexanes (1:6), which gave the desired product in 92% (546.4 mg, 2.52 mmol) yield; m.p. 116–118 °C. ^1^H NMR (400 MHz, CD_3_CN, 298 K): δ = 7.07 (dt, *J* = 8.7 Hz, *J* = 2.0 Hz, 2H), 6.96 (d, *J* = 8.3 Hz, 1H), 6.86 (s, 2H), 6.79 (dt, *J* = 8.7 Hz, *J* = 2.1 Hz, 2H), 6.20 (d, *J* = 2.6 Hz, 1H), 6.03 (dd, *J* = 8.3 Hz, *J* = 2.6 Hz, 1H), 4.62 (s, 2H), 3.76 (s, 2H), and 3.74 (s, 3H); ^13^C{^1^H} NMR (100 MHz, CD_3_CN, 298 K): δ = 159.8, 159.6, 151.9, 139.0, 131.6, 131.0, 114.5, 108.2, 106.2, 101.7, 55.8, and 39.6. HRMS (ESI) *m*/*z* calculated for [M + H]^+^ = [C_15_H_15_NO_2_S]^+^ 262.0896; observed, 262.0898.

#### 3.5.8. 3-((4-Methoxybenzyl)thio)pyridin-2-Amine (**2i**)

The title compound was prepared according to general procedure 2 on a 20.0 mmol scale. The crystalline beige solid was washed with hexanes, which gave the desired product in 92% (4.553 g, 18.48 mmol) yield; m.p. 76–78 °C. ^1^H NMR (400 MHz, DMSO-*d*_6_, 298 K): δ = 7.87 (dd, *J* = 4.8 Hz, *J* = 1.7 Hz, 1H), 7.66 (dd, *J* = 7.4 Hz, *J* = 1.6 Hz, 1H), 7.15 (d, *J* = 8.6 Hz, 2H), 6.82 (d, *J* = 8.6 Hz, 2H), 6.46 (dd, *J* = 7.4 Hz, *J* = 4.9 Hz, 1H), 6.03 (s, 2H), 3.97 (s, 2H), and 3.70 (s, 3H); ^13^C{^1^H} NMR (100 MHz, DMSO-*d*_6_, 298 K): δ = 159.1, 158.3, 147.4, 141.6, 130.1, 129.4, 113.7, 112.8, 111.9, 55.0, and 36.4. HRMS (ESI) *m*/*z* calculated for [M + H]^+^ = [C_13_H_15_N_2_OS]^+^ 247.0900; observed, 247.0899.

#### 3.5.9. 2-((4-Methoxybenzyl)thio)pyridin-3-Amine (**2j**)

The title compound was prepared according to general procedure 2 on a 20.0 mmol scale. The crystalline brown powder was washed with hexanes, which gave the desired product in 99% (4.898 g, 19.88 mmol) yield. ^1^H NMR (400 MHz, DMSO-*d*_6_, 298 K): δ = 7.79 (dd, *J* = 7.6 Hz, *J* = 1.4 Hz, 1H), 7.29 (d, *J* = 7.9 Hz, 1H), 6.90 (t, *J* = 7.9 Hz, 2H), 6.84 (d, *J* = 7.6 Hz, 2H), 5.01 (s, 2H), 4.34 (s, 2H), and 3.71 (s, 3H); ^13^C{^1^H} NMR (100 MHz, DMSO-*d*_6_, 298 K): δ = 158.2, 141.6, 141.5, 137.2, 130.2, 130.0, 120.5, 119.1, 113.7, 55.0, and 32.7. HRMS (ESI) *m*/*z* calculated for [M + H]^+^ = [C_13_H_15_N_2_OS]^+^ 247.0900; observed, 247.0899.

#### 3.5.10. 2-((4-Methoxybenzyl)thio)-5-(Trifluoromethyl)aniline (**2k**)

The title compound was prepared according to general procedure 2 on a 27.0 mmol scale. The crystalline off-white solid was washed with hexanes, which gave the desired product in 97% (8.167 g, 26.07 mmol) yield; m.p. 79–80 °C. ^1^H NMR (400 MHz, DMSO-*d*_6_, 298 K): δ = 7.28 (d, *J* = 8.0 Hz, 1H), 7.21 (d, *J* = 8.4 Hz, 2H), 7.02 (s, 2H), 6.83 (d, *J* = 8.4 Hz, 2H), 6.75 (d, *J* = 7.8 Hz, 1H), 5.66 (s, 2H), 4.04 (s, 3H), and 3.71 (s, 3H); ^13^C{^1^H} NMR (100 MHz, DMSO-*d*_6_, 298 K): δ = 158.4, 148.6, 133.1, 130.1, 129.2, 128.7 (q, *J*_C–F_ = 31.0 Hz), 124.4 (q, *J*_C–F_ = 270.4 Hz), 121.5, 113.7, 112.0 (q, *J*_C–F_ = 15.4 Hz), 109.8 (q, *J*_C–F_ = 3.9 Hz), 55.0, and 36.2; ^19^F{^1^H} NMR (376 MHz, DMSO-*d*_6_, 298 K, referenced to C_6_H_5_F): *δ* = −61.45. HRMS (ESI) *m*/*z* calculated for [M + H]^+^ = [C_15_H_15_F_3_NOS]^+^ 314.0821; observed, 314.0823.

#### 3.5.11. 3-Amino-4-((4-Methoxybenzyl)thio)benzonitrile (**2l**)

The title compound was prepared according to general procedure 2 on a 10.0 mmol scale. The crystalline yellow solid was washed with hexanes, which gave the desired product in 86% (2.315 g, 8.56 mmol) yield; m.p. 189–190 °C. ^1^H NMR (400 MHz, DMSO-*d*_6_, 298 K): δ = 7.28 (d, *J* = 8.0 Hz, 1H), 7.20 (d, *J* = 8.6 Hz, 2H), 6.98 (d, *J* = 1.6 Hz, 1H), 6.84 (d, *J* = 8.7 Hz, 2H), 6.74 (dd, *J* = 8.0 Hz, *J* = 1.4 Hz, 1H), 5.64 (s, 2H), 4.04 (s, 2H), and 3.71 (s, 3H); ^13^C{^1^H} NMR (100 MHz, DMSO-*d*_6_, 298 K): δ = 158.8, 144.7, 143.4, 136.2, 130.6, 130.0, 128.4, 126.3, 117.1, 114.2, 107.4, 55.1, and 35.6. HRMS (ESI) *m*/*z* calculated for [M + H]^+^ = [C_15_H_15_N2OS]^+^ 271.0900; observed, 271.0900.

#### 3.5.12. Ethyl 3-Amino-4-((4-Methoxybenzyl)thio)benzoate (**2m**′)

The title compound was prepared according to general procedure 2 on a 23.0 mmol scale. The crystalline yellow solid was washed with hexanes, which gave the desired product in 94% (6.894 g, 21.72 mmol) yield; m.p. 89–90 °C. ^1^H NMR (400 MHz, DMSO-*d*_6_, 298 K): δ = 7.33 (s, 1H), 7.23–7.19 (m, 3H), 7.07 (d, *J* = 7.9 Hz, 1H), 6.83 (d, *J* = 8.3, 2H), 5.44 (s, 2H), 6.88 (q, *J* = 7.0 Hz, 2H), 4.05 (s, 2H), 3.71 (s, 3H), and 1.29 (t, *J* = 7.0 Hz, 3H); ^13^C{^1^H} NMR (100 MHz, DMSO-*d*_6_, 298 K): δ = 165.9, 158.3, 147.8, 131.8, 130.1, 129.2, 129.2, 123.0, 116.8, 114.3, 113.7, 60.4, 55.0, 55.0, 36.0, and 14.2. HRMS (ESI) *m*/*z* calculated for [M + H]^+^ = [C_17_H_20_NO_2_S]^+^ 318.1158; observed, 318.1160.

#### 3.5.13. 5-Bromo-2-((4-Methoxybenzyl)thio)aniline (**2n**)

The title compound was prepared according to general procedure 2 on a 2.86 mmol scale. The crystalline light brown solid was washed with hexanes, which gave the desired product in 79% (728.1 mg, 2.25 mmol) yield; m.p. 100–102 °C. ^1^H NMR (400 MHz, DMSO-*d*_6_, 298 K): δ = 7.13 (d, *J* = 8.5 Hz, 2H), 6.98 (d, *J* = 8.2 Hz, 1H), 6.90 (d, *J* = 2.0 Hz, 1H), 6.82 (d, *J* = 8.6 Hz, 2H), 6.58 (dd, *J* = 8.2 Hz, *J* = 2.1 Hz, 1H), 5.56 (s, 2H), 3.89 (s, 2H), and 3.71 (s, 3H); ^13^C{^1^H} NMR (100 MHz, DMSO-*d*_6_, 298 K): δ = 158.2, 150.6, 136.3, 130.0, 129.6, 122.2, 118.5, 116.1, 115.2, 113.6, 55.0, and 36.9. HRMS (ESI) *m*/*z* calculated for [M + H]^+^ = [C_14_H_15_BrNOS]^+^ 324.0052; observed, 324.0052.

#### 3.5.14. 4-Bromo-2-((4-Methoxybenzyl)thio)aniline (**2o**)

The title compound was prepared according to general procedure 2 on a 30 mmol scale. The crystalline beige solid was washed with hexanes, which gave the desired product in 95% (9.287g, 28.64 mmol) yield; m.p. 86–87 °C. ^1^H NMR (400 MHz, DMSO-*d*_6_, 298 K): δ = 7.18–7.11 (m, 4H), 6.83 (dt, *J* = 8.6 Hz, *J* = 1.9 Hz, 1H), 6.66 (d, *J* = 8.6 Hz, 1H), 5.44 (s, 2H), 3.95 (s, 2H), and 3.72 (s, 3H); ^13^C{^1^H} NMR (100 MHz, DMSO-*d*_6_, 298 K): δ = 158.3, 148.3, 135.6, 131.4, 130,1, 130.0, 118.2, 115.9, 113.6, 105.9, 55.0, and 36.9. HRMS (ESI) *m*/*z* calculated for [M + H]^+^ = [C_14_H_15_BrNOS]^+^ 324.0052; observed, 324.0050.

#### 3.5.15. 5-Chloro-2-((4-Methoxybenzyl)thio)aniline (**2p**)

The title compound was prepared according to general procedure 2 on a 2.72 mmol scale. The crystalline pale brown powder was washed with hexanes, which gave the desired product in 93% (7.142 g, 2.55 mmol) yield; m.p. 80–82 °C. ^1^H NMR (400 MHz, DMSO-*d*_6_, 298 K): δ = 7.13 (d, *J* = 8.5 Hz, 2H), 7.05 (d, *J* = 8.2 Hz, 1H), 6.81 (d, *J* = 2.0 Hz, 1H), 6.75 (d, *J* = 8.6 Hz, 2H), 6.45 (dd, *J* = 8.2 Hz, *J* = 2.1 Hz, 1H), 5.58 (s, 2H), 3.89 (s, 2H), and 3.71 (s, 3H); ^13^C{^1^H} NMR (100 MHz, DMSO-*d*_6_, 298 K): δ = 158.2, 150.5, 136.2, 133.6, 130.0, 129.6, 115.6, 114.7, 113.6, 113.2, 55.0, and 37.0. HRMS (ESI) *m*/*z* calculated for [M + H]^+^ = [C_14_H_15_ClNOS]^+^ 280.0557; observed, 280.0558.

#### 3.5.16. 3-Chloro-2-((4-Methoxybenzyl)thio)aniline (**2q**)

The title compound was prepared according to general procedure 2 on a 20.0 mmol scale. The crystalline off-white powder was washed with hexanes, which gave the desired product in 94% (5.282 g, 18.88 mmol) yield; m.p. 71–73 °C. ^1^H NMR (400 MHz, DMSO-*d*_6_, 298 K): δ = 7.14 (d, *J* = 8.5 Hz, 2H), 7.00 (t, *J* = 8.0 Hz, 1H), 6.80 (d, *J* = 8.5 Hz, 2H), 6.67–6.63 (m, 2H), 5.66 (s, 2H), 3.86 (s, 2H), and 3.70 (s, 3H); ^13^C{^1^H} NMR (100 MHz, DMSO-*d*_6_, 298 K): δ = 158.3, 152.4, 139.8, 130.5, 130.0, 129.4, 116.5, 113.6, 113.4, 112.8, 55.0, and 37.0. HRMS (ESI) *m*/*z* calculated for [M + H]^+^ = [C_14_H_15_ClNOS]^+^ 280.0557; observed, 280.0559.

#### 3.5.17. 3-Fluoro-2-((4-Methoxybenzyl)thio)aniline (**2r**)

The title compound was prepared according to general procedure 2 on a 12 mmol scale. The crystalline off-white powder was washed with hexanes, which gave the desired product in 97% (2.064 g, 11.64 mmol) yield; m.p. 71–72 °C. ^1^H NMR (400 MHz, DMSO-*d*_6_, 298 K): δ = 7.12 (d, *J* = 8.6 Hz, 2H), 7.02 (q, *J* = 6.7 Hz, 1H), 6.79 (d, *J* = 8.6 Hz, 2H), 6.52 (d, *J* = 8.2 Hz, 1H), 6.32–6.28 (m, 1H), 5.62 (s, 2H), 3.83 (s, 2H), and 3.70 (s, 3H); ^13^C{^1^H} NMR (100 MHz, DMSO-*d*_6_, 298 K): δ = 163.8 (d, *J*_C–F_ = 239.0 Hz), 158.2, 152.1 (d, *J*_C–F_ = 3.8 Hz), 130.5 (d, *J*_C–F_ = 11.0 Hz), 129.9, 129.7, 113.6, 109.8 (d, *J*_C–F_ = 2.5 Hz), 102.4 (d, *J*_C–F_ = 21.4 Hz), 101.9 (d, *J*_C–F_ = 24.0 Hz), 55.0, and 36.9; ^19^F{^1^H} NMR (376 MHz, DMSO-*d*_6_, 298 K, referenced to C_6_H_5_F): *δ* = −108.81. HRMS (ESI) *m*/*z* calculated for [M + H]^+^ = [C_14_H_15_FNOS]^+^ 264.0853; observed, 264.0854.

### 3.6. Synthesis of 4-Fluoro-2-((4-Methoxybenzyl)thio)aniline (**2s**)

#### 3.6.1. 2,2′-Disulfanediylbis(4-Fluoroaniline)

A 500 mL round-bottomed flask equipped with a stir bar was charged with 16.802 g (100 mml) of 2-amino-6-fluorobenzothiazole, 100 mL of 10 M NaOH, and 100 mL of 2-methoxyethanol. The flask was fitted with a reflux condenser and the reaction mixture was stirred at 100 °C for 4 days. After cooling to room temperature, the reaction mixture was acidified with HCl to pH 3 and extracted twice with diethyl ether. The combined organic phases were washed with brine, dried over MgSO_4,_ filtered, and evaporated. The product obtained from the extraction was sufficiently pure to be used without further purification. Yield: 69% (9.815 g, 34.52 mmol). ^1^H NMR (400 MHz, CD_3_CN, 298 K): δ = 6.96 (dt, *J* = 8.7 Hz, *J* = 3.0 Hz, 2H), 6.85 (dd, *J* = 8.6 Hz, *J* = 3.8 Hz, 1H), 6.75 (dd, *J* = 8.9 Hz, *J* = 4.9 Hz, 2H), and 4.62 (s, 2H); ^13^C{^1^H} NMR (100 MHz, CD_3_CN, 298 K): δ = 155.1 (d, *J*_C–F_ = 234.0 Hz), 147.1 (d, *J*_C–F_ = 1.7 Hz), 121.8 (d, *J*_C–F_ = 22.2 Hz), 119.4 (d, *J*_C–F_ = 22.5 Hz), 118.6 (d, *J*_C–F_ = 7.3 Hz), and 116.9 (d, *J*_C–F_ = 7.5 Hz); ^19^F{^1^H} NMR (376 MHz, CD_3_CN, 298 K, referenced to C_6_H_5_F): *δ* = −127.83.

#### 3.6.2. 4-Fluoro-2-((4-Methoxybenzyl)thio)aniline (**2s**)

Reduction of 2,2′-Disulfanediylbis(4-fluoroaniline) to 2-amino-5-fluorobenzenethiol: A 1 L round-bottomed flask equipped with a stir bar was charged with 9.815 g (34.52 mmol, 1 equiv.) of 2,2′-Disulfanediylbis(4-fluoroaniline), 200 mL of THF, and 200 mL of MeOH. While stirring in an ice bath, NaBH_4_ (5.22 g, 138 mmol, 4 equiv.) was added in small portions over a period of 15 min under a counterflow of argon. The ice bath was then removed, and the reaction mixture was stirred at room temperature for 1h. The reaction mixture was concentrated, water was added, and the product was extracted with diethyl ether. After drying the organic phase over MgSO_4_, filtration, and removal of the solvent, the crude 2-amino-5-fluorobenzenethiol was used immediately in the next step. Protection of 2-amino-5-fluorobenzenethiol: The crude 2-amino-5-fluorobenzenethiol (max. 69 mmol, 1 equiv.) was dissolved in 100 mL of EtOH and transferred to a 250 mL round-bottomed flask equipped with a stir bar, and 10.41 g (75 mmol, 1.08 equiv.) of 1-(chloromethyl)-4-methoxybenzene was added. After setting the reaction under argon, 3.25 g (81.3 mmol, 1.18 equiv.) of NaOH dissolved in 10 mL of water was added dropwise under a counterflow of argon. Then, the reaction flask was fitted with a reflux condenser and stirred under an argon atmosphere at 50 °C for 4 h. After cooling to room temperature, the reaction mixture was concentrated. Basification with 1 M NaOH, followed by extraction with diethyl ether and drying over MgSO_4_ gave the crude product as an oil upon filtration and solvent removal. Recrystallization from hot diethyl ether gave the product as a pale yellow powder in 9.747 g (37.01 mmol, 54%) yield. ^1^H NMR (400 MHz, DMSO-*d*_6_, 298 K): δ = 7.18 (dt, *J* = 8.6 Hz, *J* = 2.0 Hz, 2H), 6.92 (dd, *J* = 9.1 Hz, *J* = 3.0 Hz, 1H), 6.87–6.81 (m, 3H), 6.69 (dd, *J* = 8.8 Hz, *J* = 5.3 Hz, 1H), 5.08 (s, 2H), 3.99 (s, 2H), and 3.71 (s, 3H); ^13^C{^1^H} NMR (100 MHz, DMSO-*d*_6_, 298 K): δ = 158.3, 153.6 (d, *J*_C–F_ = 231.9 Hz), 145.1 (d, *J*_C–F_ = 1.6 Hz), 130.0, 129.4, 118.8 (d, *J*_C–F_ = 22.2 Hz), 117.5 (d, *J*_C–F_ = 7.6 Hz), 115.3 (d, *J*_C–F_ = 21.8 Hz), 114.8 (d, *J*_C–F_ = 7.6 Hz), 113.7, 55.0, and 36.5; ^19^F{^1^H} NMR (376 MHz, DMSO-*d*_6_, 298 K, referenced to C_6_H_5_F): *δ* = −128.70. HRMS (ESI) *m*/*z* calculated for [M + H]^+^ = [C_14_H_15_FNOS]^+^ 264.0853; observed, 264.0854.

### 3.7. General Procedure 3 for the Synthesis of Substituted Triazoles (**3b**–**3s**)

A 50 mL round-bottomed flask equipped with a stir bar was loaded the 2-((4-methoxybenzyl)thio)aniline derivative (**2b**–**2s**, 1 equiv.) and *N,N*-dimethylformamide azine dihydrochloride (1.1 equiv). After mixing the two compounds thoroughly, the reaction flask was placed under an inert atmosphere (argon) and stirred in an oil bath at 150 °C. The two solid compounds were observed to melt within an hour and turn dark red. After 16 h, the reaction flask was cooled to room temperature. The resulting dark solid was basified with 1 M NaOH and extracted with dichloromethane, dried over MgSO_4_, filtered, and concentrated. The crude product was purified by column chromatography or recrystallization as described below.

#### 3.7.1. 4-(2-((4-Methoxybenzyl)thio)-3-Methylphenyl)-4*H*-1,2,4-triazole (**3b**)

The title compound was prepared according to general procedure 3 on a 3.63 g (14.0 mmol) scale. Purification by column chromatography (silica gel, 1. dichloromethane, and 2. dichloromethane/acetone 4:1; R_f_ = 0.56) gave the product as an off-white powder in 72% (3.15 g, 10.1 mmol) yield; m.p. 116–118 °C. ^1^H NMR (400 MHz, CDCl_3_, 298 K): *δ* = 7.92 (s, 2H), 7.43 (d, *J* = 7.6 Hz, 1H), 7.35 (t, *J* = 7.6 Hz, 1H), 7.04 (t, *J* = 7.9 Hz, 1H), 6.68 (s, 4H), 3.74 (s, 1H), 3.53 (s, 1H), and 2.61 (s, 1H); ^13^C{^1^H} NMR (100 MHz, CDCl_3_, 298 K): *δ* = 158.7, 145.5, 143.1, 137.8, 131.6, 129.6, 129.5 (2 signals), 128.7, 124.1, 113.7, 55.1, 38.7, and 21.4. HRMS (ESI) *m*/*z:* [M + H]^+^ calculated for C_17_H_17_N_3_OS, 312.1165; observed, 312.1165.

#### 3.7.2. 4-(2-((4-Methoxybenzyl)thio)-6-Methylphenyl)-4*H*-1,2,4-triazole (**3c**)

The title compound was prepared according to general procedure 3 on a 4.67 g (18.0 mmol) scale. Purification by column chromatography (silica gel, 1. dichloromethane, and 2. dichloromethane/acetone 4:1; R_f_ = 0.41) gave the product as an off-white powder in 49% (2.76 g, 7.55 mmol) yield; m.p. 191–193 °C. ^1^H NMR (400 MHz, DMSO-*d*_6_, 298 K): *δ* = 8.59 (s, 2H), 7.46–7.40 (m, 2H), 7.27 (d, *J* = 6.7 Hz, 1H), 7.21 (d, *J* = 8.5 Hz, 1H), 6.86 (d, *J* = 8.5 Hz, 1H), 4.12 (s, 1H), 3.71 (s, 1H), and 1.96 (s, 1H); ^13^C{^1^H} NMR (100 MHz, DMSO-*d*_6_, 298 K): *δ* = 158.5, 143.2, 135.9, 135.7, 131.3, 130.0 (2 signals), 128.0 (2 signals), 126.2, 113.9, 55.1, 35.7, and 17.2. HRMS (ESI) *m*/*z:* [M + H]^+^ calculated for C_17_H_17_N_3_OS, 312.1165; observed, 312.1165.

#### 3.7.3. 4-(1-((4-Methoxybenzyl)thio)naphthalen-2-yl)-4*H*-1,2,4-triazole (**3d**)

The title compound was prepared according to general procedure 3 on a 975 mg (3.30 mmol) scale. Purification by column chromatography (silica gel, 1. dichloromethane, and 2. dichloromethane/acetone 4:1; R_f_ = 0.67) gave the product as a pale yellow powder in 17% (195 mg, 0.56 mmol) yield; m.p. 129–130 °C. ^1^H NMR (400 MHz, DMSO-*d*_6_, 298 K): *δ* = 8.65 (d, *J* = 8.4 Hz, 1H), 8.52 (s, 2H), 8.18 (d, *J* = 8.6 Hz, 1H), 8.13 (d, *J* = 7.9 Hz, 1H), 7.81–7.77 (m, 1 H), 7.73–7.69 (m, 1 H), 7.57 (d, *J* = 8.6 Hz, 1H), 6.70 (s, 4H), 3.81 (s, 3H), and 3.68 (s, 3H); ^13^C{^1^H} NMR (100 MHz, DMSO-*d*_6_, 298 K): *δ* = 158.4, 143.6, 136.3, 134.2, 133.3, 131.0, 129.7, 129.0, 128.7, 128.5, 127.7, 127.4, 126.7, 124.4, 113.7, and 55.1. HRMS (ESI) *m*/*z:* [M + H]^+^ calculated C_20_H_17_N_3_OS, 348.1165; observed, 348.1163.

#### 3.7.4. N-(4-((4-Methoxybenzyl)thio)-3-(4H-1,2,4-Triazol-4-Yl)phenyl)acetamide (**3e**)

The title compound was prepared according to general procedure 2 on a 3.26 g (10.8 mmol) scale. Purification by column chromatography (silica gel, 1. dichloromethane, and 2. dichloromethane/acetone 4:1; R_f_ = 0.19) gave the product as a pale brown powder in 54% (2.06 g, 5.80 mmol) yield; m.p. 176–178 °C. ^1^H NMR (400 MHz, DMSO-*d*_6_, 298 K): *δ* = 10.26 (s, 1H), 8.58 (s, 1H), 7.69 (d, *J* = 1.8 Hz, 1H), 7.62 (dd, *J* = 8.6 Hz, *J* = 2.0 Hz, 1H), 7.56 (d, *J* = 8.6 Hz, 1H), 7.06 (d, *J* = 8.6 Hz, 2H), 6.82 (d, *J* = 8.6 Hz, 2H), 3.97 (s, 2H), 3.71 (s, 3H), and 2.06 (s, 3H); ^13^C{^1^H} NMR (100 MHz, DMSO-*d*_6_, 298 K): *δ* = 168.9, 158.5, 143.4, 139.1, 134.3, 133.1, 130.0, 128.6, 125.6, 120.1, 117.1, 113.9, 55.1, 37.8, and 24.1. HRMS (ESI) *m*/*z:* [M + H]^+^ calculated C_18_H_18_N_4_O_2_S, 355.1223; observed, 355.1225.

#### 3.7.5. 4-((4-Methoxybenzyl)thio)-3-(4H-1,2,4-Triazol-4-Yl)aniline (**3f**)

The title compound was obtained as a by-product of the synthesis of compound **3f′**, which was prepared according to general procedure 3 on a 3.64 mg (14.0 mmol) scale; for this reaction, 2.2 equiv. of *N,N*-dimethylformamide azine dihydrochloride was used. Purification by column chromatography (silica gel, 1. dichloromethane, and 2. dichloromethane/acetone 4:1; R_f_ = 0.26) gave the product as a pale brown powder in 19% (818 mg, 2.24 mmol) yield; m.p. 147–149 °C. ^1^H NMR (400 MHz, DMSO-*d*_6_, 298 K): *δ* = 9.01 (s, 2H), 7.24 (d, *J* = 8.2 Hz, 1H), 7.18 (dt, *J* = 8.6 Hz, *J* = 2.0 Hz, 2H), 6.92 (d, *J* = 2.4 Hz, 1H), 6.83 (dt, *J* = 8.6 Hz, *J* = 2.0 Hz, 2H), 6.74 (dd, *J* = 8.2 Hz, *J* = 2.4 Hz, 2H), 7.96–7.90 (m, 3 H), 7.86–7.80 (m, 3 H), 7.70 (tt, *J* = 7.4 Hz, *J* = 1.1 Hz, 1H), 5.46 (s, 2H), 3.96 (s, 2H), and 3.71 (s, 3H); ^13^C{^1^H} NMR (100 MHz, DMSO-*d*_6_, 298 K): *δ* = 158.7, 150.7, 141.8, 136.3, 135.0, 130.5, 130.0, 116.5, 114.3, 114.2, 109.3, 106.6, 55.5, and 37.5. HRMS (ESI) *m*/*z:* [M + H]^+^ calculated C_16_H_16_N_4_OS, 313.1118; observed, 313.1120.

#### 3.7.6. 4,4′-(4-((4-. Methoxybenzyl)thio)-1,3-Phenylene)bis(4H-1,2,4-Triazole) (**3f**′)

The title compound was prepared according to general procedure 3 on a 3.64 g (14.0 mmol) scale; for this reaction, 2.2 equiv. of *N,N*-dimethylformamide azine dihydrochloride was used. Purification by column chromatography (silica gel, 1. dichloromethane, and 2. dichloromethane/methanol 10:1; R_f_ = 0.49) gave the product as a pale yellow powder in 21% (1.08 mg, 2.95 mmol) yield; m.p. 250–253 °C. ^1^H NMR (400 MHz, DMSO-*d*_6_, 298 K): *δ* = 9.21 (s, 2H), 8.74 (s, 2H), 7.99 (s, 1H), 7.91 (d, *J* = 7.0 Hz, 1H), 7.81 (d, *J* = 7.6 Hz, 1H), 7.20 (d, *J* = 6.2 Hz, 2H), 6.85 (d, *J* = 6.4 Hz, 2H), 4.20 (s, 2H), and 3.71 (s, 3H); ^13^C{^1^H} NMR (100 MHz, DMSO-*d*_6_, 298 K): *δ* = 158.6, 143.3, 141.1, 133.7, 132.8, 132.4, 131.4, 130.0, 127.9, 121.8, 119.7, 113.9, 55.1, and 36.4. HRMS (ESI) *m*/*z:* [M + H]^+^ calculated C_18_H_16_N_6_OS, 365.1179; observed, 365.1180.

#### 3.7.7. 4-(5-Methoxy-2-((4-Methoxybenzyl)thio)phenyl)-4*H*-1,2,4-triazole (**3g**)

The title compound was prepared according to general procedure 3 on a 4.13 g (15.0 mmol) scale. Purification by column chromatography (silica gel, 1. dichloromethane, and 2. dichloromethane/acetone 4:1; R_f_ = 0.44) gave the product as a white powder in 60% (2.92 g, 8.93 mmol) yield; m.p. 128–130 °C. ^1^H NMR (400 MHz, DMSO-*d*_6_, 298 K): *δ* = 8.56 (s, 2H), 7.57 (dd, *J* = 9.2 Hz, *J* = 2.1 Hz, 1H), 7.11–7.08 (m, 2H), 7.00 (d, *J* = 8.6 Hz, 2H), 6.80 (d, *J* = 8.6 Hz, 2H), 3.89 (s, 2H), 3.80 (s, 3H), and 3.71 (s, 3H); ^13^C{^1^H} NMR (100 MHz, DMSO-*d*_6_, 298 K): *δ* = 159.3, 158.4, 143.3, 136.0, 135.1, 129.9, 128.9, 122.1, 116.0, 113.8, 112.9, 55.8, 55.1, and 36.7 HRMS (ESI) *m/z:* [M + H]^+^ calculated C_17_H_18_N_3_O_2_S, 328.1114; observed 328.1114.

#### 3.7.8. 4-((4-Methoxybenzyl)thio)-3-(4H-1,2,4-Triazol-4-Yl)phenol (**3h**)

The title compound was prepared according to general procedure 3 on a 900 mg (3.44 mmol) scale. Purification by column chromatography (silica gel, 1. dichloromethane, and 2. dichloromethane/acetone 4:1; R_f_ = 0.22) gave the product as a white powder in 55% (570 mg, 1.91 mmol) yield; m.p. 212–214 °C. ^1^H NMR (400 MHz, DMSO-*d*_6_, 298 K): *δ* = 10.25 (s, 1H), 8.52 (s, 2H), 7.45 (d, *J* = 8.6 Hz, 1H), 6.97 (d, *J* = 8.6 Hz, 2H), 6.91 (dd, *J* = 8.6 Hz, *J* = 2.6 Hz, 1H), 6.80–6.78 (m, 3H) 3.81 (s, 2H), and 3.71 (s, 3H); ^13^C{^1^H} NMR (100 MHz, DMSO-*d*_6_, 298 K): *δ* = 158.3, 158.0, 143.3, 136.4, 136.1, 129.9, 129.0, 119.7, 117.1, 114.1, 113.7, and 55.1. HRMS (ESI) *m*/*z:* [M + H]^+^ calculated C_16_H_15_N_3_O_2_S, 314.0958; observed, 314.0959.

#### 3.7.9. 3-((4-Methoxybenzyl)thio)-2-(4H-1,2,4-Triazol-4-Yl)pyridine (**3i**)

The title compound was prepared according to general procedure 3 on a 3.45 g (14.0 mmol) scale. Purification by column chromatography (silica gel, 1. dichloromethane, and 2. dichloromethane/acetone 4:1; R_f_ = 0.44) gave the product as an orange powder in 10% (421 mg, 1.41 mmol) yield; m.p. 154–156 °C. ^1^H NMR (400 MHz, DMSO-*d*_6_, 298 K): *δ* = 8.71 (s, 2H), 8.46 (d, *J* = 6.3 Hz, 1H), 7.77 (d, *J* = 7.2 Hz, 1H), 7.39 (d, *J* = 8.28 Hz, 2H), 6.99–6.92 (m, 3H), 5.83 (s, 2 H), and 3.74 (s, 3 H); ^13^C{^1^H} NMR (100 MHz, DMSO-*d*_6_, 298 K): *δ* = 175.6, 159.0, 143.3, 143.2, 136.1, 133.0, 129.8, 127.5, 114.0, 111.8, 58.2, and 55.1. HRMS (ESI) *m*/*z:* [M + H]^+^ calculated C_15_H_14_N_4_OS, 299.0961; observed, 299.0961.

#### 3.7.10. 2-((4-Methoxybenzyl)thio)-3-(4H-1,2,4-Triazol-4-Yl)pyridine (**3j**)

The title compound was prepared according to general procedure 3 on a 3.70 g (15.0 mmol) scale. Purification by column chromatography (silica gel, 1. dichloromethane, and 2. dichloromethane/acetone 4:1; R_f_ = 0.48) gave the product as a white powder in 11% yield (504 mg, 1.70 mmol); m.p. 219–220 °C. ^1^H NMR (400 MHz, DMSO-*d*_6_, 298 K): *δ* = 8.85 (s, 2H), 8.42 (dd, *J* = 4.7 Hz, *J* = 1.4 Hz, 1H), 8.16 (dd, *J* = 8.0 Hz, *J* = 1.4 Hz, 1H), 7.56 (dd, *J* = 8.0 Hz, *J* = 4.7 Hz, 1H), 7.18 (d, *J* = 8.6 Hz, 2H), 6.83 (d, *J* = 8.6 Hz, 2H), 4.20 (s, 2H), and 3.71 (s, 3H); ^13^C{^1^H} NMR (100 MHz, DMSO-*d*_6_, 298 K): *δ* = 158.6, 146.4, 144.9, 142.4, 140.2, 130.1, 128.5, 127.6, 125.0, 113.9, 55.1, and 36.4. HRMS (ESI) *m*/*z:* [M + H]^+^ calculated C_15_H_14_N_4_OS, 299.0961; observed, 299.0960.

#### 3.7.11. 4-(2-((4-Methoxybenzyl)thio)-5-(Trifluoromethyl)phenyl)-4*H*-1,2,4-triazole (**3k**)

The title compound was prepared according to general procedure 3 on an 8.15 g (26.0 mmol) scale. Purification by column chromatography (silica gel, 1. dichloromethane, and 2. dichloromethane/acetone 4:1; R_f_ = 0.62) gave the product as an off-white powder in 48% (4.54 g, 12.41 mmol) yield; m.p. 126–127 °C. ^1^H NMR (400 MHz, CD_3_CN, 298 K): δ = 8.32 (s, 2H), 7.76 (dd, *J* = 8.5 Hz, *J* = 1.6 Hz, 1H), 7.73 (d, *J* = 8.4 Hz, 1H), 7.68 (s, 1H), 7.19 (dt, *J* = 8.7 Hz, *J* = 2.0 Hz, 2H), 6.83 (dt, *J* = 8.7 Hz, *J* = 2.1 Hz, 2H), 4.16 (s, 2H), and 3.74 (s, 3H); ^13^C{^1^H} NMR (100 MHz, CD_3_CN, 298 K): δ = 160.2, 143.9, 141.2, 133.8, 131.0, 130.7, 128.7 (q, *J*_C–F_ = 33.2 Hz), 128.4, 127.6 (q, *J*_C–F_ = 3.7 Hz), 125.3 (q, *J*_C–F_ = 3.8 Hz), 124.6 (q, *J*_C–F_ = 269.7 Hz), 115.0, 55.9, and 37.4; ^19^F{^1^H} NMR (376 MHz, CD_3_CN, 298 K, referenced to C_6_H_5_F): *δ* = −61.45. HRMS (ESI) *m*/*z:* [M + H]^+^ calculated C_17_H_14_F_3_N_3_OS, 366.0883; observed, 366.0882.

#### 3.7.12. 4-((4-Methoxybenzyl)thio)-3-(4H-1,2,4-Triazol-4-yl)benzonitrile (**3l**)

The title compound was prepared according to general procedure 3 on a 1.94 g (7.16 mmol) scale. Purification by column chromatography (silica gel, 1. dichloromethane, and 2. dichloromethane/acetone 4:1; R_f_ = 0.53) gave the product as a pale yellow powder in 32% (750 mg, 2.33 mmol) yield; m.p. 209–210 °C. ^1^H NMR (400 MHz, DMSO-*d*_6_, 298 K): δ = 8.75 (s, 2H), 8.05 (d, *J* = 1.7 Hz, 2H), 7.97 (dd, *J* = 8.3 Hz, *J* = 1.7 Hz, 1H), 7.78 (d, *J* = 8.4 Hz, 1H), 7.28 (d, *J* = 8.7 Hz, 2H), 6.87 (d, *J* = 8.7 Hz, 2H), 4.32 (s, 2H), and 3.72 (s, 3H); ^13^C{^1^H} NMR (100 MHz, DMSO-*d*_6_, 298 K): δ = 158.7, 143.2, 142.4, 133.3, 131.7, 130.8, 130.2, 127.9, 126.9, 117.7, 114.0, 108.0, 55.1, and 34.9. HRMS (ESI) *m*/*z:* [M + H]^+^ calculated C_17_H_14_N_4_OS, 323.0961; observed, 323.0962.

#### 3.7.13. 4-((4-Methoxybenzyl)thio)-3-(4H-1,2,4-Triazol-4-Yl)benzoic Acid (**3m**)

A 50 mL round-bottomed flask was charged with compound **3m**′ (1.50 mmol, 1 equiv.), KOH (2 equiv.), and MeOH, and then heated to 35 °C for 1 h. After removal of the solvent under reduced pressure, the crude product was purified by column chromatography (silica gel, 1. dichloromethane, and 2. dichloromethane/methanol 10:1, R_f_ = 0.19), which gave the product as a white powder in 87% (413 mg, 1.21 mmol) yield; m.p. > 360 °C. ^1^H NMR (400 MHz, DMSO-*d*_6_, 298 K): *δ* = 13.29 (s, 1H), 8.74 (s, 2H), 8.02 (d, *J* = 8.0 Hz, 1H), 7.85 (s, 1H), 7.74 (d, *J* = 8.3 Hz, 1H), 7.27 (d, *J* = 8.1 Hz, *J*, 2H), 6.86 (d, *J* = 8.4 Hz, 2H), 4.27 (s, 2H), and 3.71 (s, 3H); ^13^C{^1^H} NMR (100 MHz, DMSO-*d*_6_, 298 K): *δ* = 166.0, 158.6, 143.3, 140.7, 131.5, 130.4, 130.2, 128.6, 127.8, 127.7, 127.3, 114.0, 7, 55.1, and 35.2. HRMS (ESI) *m*/*z:* [M + H]^+^ calculated C_17_H_15_N_3_O_3_S, 342.0907; observed, 342.0908.

#### 3.7.14. Ethyl 4-((4-Methoxybenzyl)thio)-3-(4H-1,2,4-Triazol-4-Yl)benzoate (**3m**′)

The title compound was prepared according to general procedure 3 on a 6.89 g (15.0 mmol) scale. Purification by column chromatography (silica gel, 1. dichloromethane, and 2. dichloromethane/acetone 4:1; R_f_ = 0.64) gave the product as a pale yellow powder in 32% (1.76 g, 4.76 mmol) yield; m.p. 126–127 °C. ^1^H NMR (400 MHz, DMSO-*d*_6_, 298 K): *δ* = 8.74 (s, 2H), 8.03 (dd, *J* = 8.4 Hz, *J* = 1.6 Hz, 1H), 7.89 (d, *J* = 1.5 Hz, 1H), 7.76 (d, *J* = 8.4 Hz, 1H), 7.28 (d, *J* = 8.5 Hz, 2H), 6.86 (d, *J* = 8.6 Hz, 2H), 4.34–4.28 (m, 4H), 3.71 (s, 3H), and 1.31 (t, *J* = 7.1 Hz, 1H); ^13^C{^1^H} NMR (100 MHz, DMSO-*d*_6_, 298 K): *δ* = 164.5, 158.6, 143.2, 141.5, 131.5, 130.2, 127.7, 127.6, 127.3, 127.1, 114.0, 61.2, 55.1, 35.1, and 14.1. HRMS (ESI) *m*/*z:* [M + H]^+^ calculated C_19_H_19_N_3_O_3_S, 370.1220; observed, 370.1222.

#### 3.7.15. 4-(5-Bromo-2-((4-Methoxybenzyl)thio)phenyl)-4*H*-1,2,4-triazole (**3n**)

The title compound was prepared according to general procedure 3 on a 1.30 g (4.00 mmol) scale. Purification by column chromatography (silica gel, 1. dichloromethane, and 2. dichloromethane/acetone 4:1; R_f_ = 0.62) gave the product as a pale brown powder in 28% (421 mg, 1.15 mmol) yield; m.p. 163–165 °C. ^1^H NMR (400 MHz, DMSO-*d*_6_, 298 K): *δ* = 8.67 (s, 2H), 7.77 (s, 1H), 7.71 (d, *J* = 8.0 Hz, 1H), 7.57 (d, *J* = 8.2 Hz, 1H), 7.17 (d, *J* = 7.3 Hz, 1H), 6.84 (d, *J* = 7.2 Hz, 1H), 4.13 (s, 2H), and 3.71 (s, 3H); ^13^C{^1^H} NMR (100 MHz, DMSO-*d*_6_, 298 K): *δ* = 158.5, 143.2, 133.9, 133.3, 132.7, 131.5, 130.0 (2 signals), 127.8, 119.0, 113.9, 55.1, and 36.3. HRMS (ESI) *m*/*z:* [M + H]^+^ calculated C_16_H_14_BrN_3_OS, 376.0114; observed, 376.0113.

#### 3.7.16. 4-(4-Bromo-2-((4-Methoxybenzyl)thio)phenyl)-4*H*-1,2,4-triazole (**3o**)

The title compound was prepared according to general procedure 3 on a 7.78 g (24.0 mmol) scale. Purification by column chromatography (silica gel, 1. dichloromethane, and 2. dichloromethane/acetone 4:1; R_f_ = 0.60) gave the product as a pale brown powder in 32% (2.86 g, 7.83 mmol) yield; m.p. 173–175 °C. ^1^H NMR (400 MHz, DMSO-*d*_6_, 298 K): *δ* = 8.67 (s, 2H), 7.80 (d, *J* = 2.1 Hz, 1H), 7.58 (dd, *J* = 8.6 Hz, *J* = 2.1 Hz, 1H), 7.40 (d, *J* = 8.3 Hz, 1H), 7.20 (d, *J* = 8.6 Hz, 2H), 6.86 (d, *J* = 8.6 Hz, 2H), 4.22 (s, 2H), and 3.72 (s, 3H); ^13^C{^1^H} NMR (100 MHz, DMSO-*d*_6_, 298 K): *δ* = 158.6, 143.3, 136.6, 131.6, 131.4, 130.2, 129.5, 129.1, 127.5, 123.0, 114.0, 55.1, and 35.9. HRMS (ESI) *m*/*z:* [M + H]^+^ calculated C_16_H_14_BrN_3_OS, 376.0114; observed, 376.0112.

#### 3.7.17. 4-(5-Chloro-2-((4-Methoxybenzyl)thio)phenyl)-4*H*-1,2,4-triazole (**3p**)

The title compound was prepared according to general procedure 3 on a 3.36 g (12.0 mmol) scale. Purification by column chromatography (silica gel, 1. dichloromethane, and 2. dichloromethane/acetone 4:1; R_f_ = 0.60) gave the product as a pale yellow powder in 32% (1.29 g, 3.88 mmol) yield; m.p. 158–159 °C. ^1^H NMR (400 MHz, CDCl_3_, 298 K): *δ* = 8.08 (s, 2H), 7.48 (d, *J* = 8.5 Hz, 1H), 7.39 (dd, *J* = 8.5 Hz, *J* = 2.1 Hz, 1H), 7.19 (d, *J* = 2.1 Hz, 1H), 6.94 (d, *J* = 8.6 Hz, 1H), 6.73 (d, *J* = 8.6 Hz, 1H), 3.85 (s, 1H), and 3.73 (s, 1H); ^13^C{^1^H} NMR (100 MHz, CDCl_3_, 298 K): *δ* = 159.1, 142.7, 134.9, 133.8, 133.6, 131.7, 130.0 129.8, 127.6, 126.8, 114.1, 55.3, and 39.0. HRMS (ESI) *m*/*z:* [M + H]^+^ calculated C_16_H_14_ClN_3_OS, 332.0619; observed, 332.0618.

#### 3.7.18. 4-(3-Chloro-2-((4-Methoxybenzyl)thio)phenyl)-4*H*-1,2,4-triazole (**3q**)

The title compound was prepared according to general procedure 3 on a 4.20 mg (15.0 mmol) scale. Purification by column chromatography (silica gel, 1. dichloromethane, and 2. dichloromethane/acetone 4:1; R_f_ = 0.43) gave the product as an off-white powder in 39% (1.93 mg, 5.82 mmol) yield; m.p. 121–123 °C. ^1^H NMR (400 MHz, DMSO-*d*_6_, 298 K): *δ* = 8.41 (s, 2H), 7.79 (dd, *J* = 8.1 Hz, *J* = 1.3 Hz, 1H), 7.57 (t, *J* = 8.0 Hz, 1H), 7.46 (dd, *J* = 7.9 Hz, *J* = 1.3 Hz, 1H), 3.87 (s, 2H), and 3.71 (s, 3H); ^13^C{^1^H} NMR (100 MHz, DMSO-*d*_6_, 298 K): *δ* = 158.5, 143.4, 140.4, 139.1, 131.0 (2 signals), 129.8, 129.8, 128.6, 126.3, 113.8, 55.1, and 38.0. HRMS (ESI) *m*/*z:* [M + H]^+^ calculated C_16_H_14_ClN_3_OS, 332.0619; observed, 332.0617.

#### 3.7.19. 4-(3-Fluoro-2-((4-Methoxybenzyl)thio)phenyl)-4*H*-1,2,4-triazole (**3r**)

The title compound was prepared according to general procedure 3 on a 2.11 g (8.00 mmol) scale. Purification by column chromatography (silica gel, 1. dichloromethane, and 2. dichloromethane/acetone 4:1; R_f_ = 0.41) gave the product as a pale brown powder in 87% (2.20 g, 6.984 mmol) yield; m.p. 218–219 °C. ^1^H NMR (400 MHz, DMSO-*d*_6_, 298 K): *δ* = 8.46 (s, 2H), 7.62–7.56 (m, 1H), 7.50 (td, *J* = 9.7 Hz, *J* = 1.0 Hz, 1H), 7.35 (d, *J* = 7.9 Hz, 1H), 6.91 (d, *J* = 8.6 Hz, 2H), 6.77 (d, *J* = 8.6 Hz, 2H), 3.88 (s, 2H), and 3.70 (s, 3H); ^13^C{^1^H} NMR (100 MHz, DMSO-*d*_6_, 298 K): *δ* = 163.0 (d, *J*_C–F_ = 244.5 Hz), 158.5, 143.3, 137.9 (d, *J*_C–F_ = 3.0 Hz), 131.2 (d, *J*_C–F_ = 9.9 Hz), 129.8, 128.8, 123.2 (d, *J*_C–F_ = 3.2 Hz), 118.7 (d, *J*_C–F_ = 21.0 Hz), 116.9 (d, *J*_C–F_ = 23.8 Hz), 131.8, 55.1, and 38.0. ^19^F{^1^H} NMR (376 MHz, DMSO-*d*_6_, 298 K, referenced to C_6_H_5_F): *δ* = −102.99. HRMS (ESI) *m*/*z:* [M + H]^+^ calculated C_16_H_14_FN_3_OS, 316.0915; observed, 316.0913.

#### 3.7.20. 4-(4-Fluoro-2-((4-Methoxybenzyl)thio)phenyl)-4*H*-1,2,4-triazole (**3s**)

The title compound was prepared according to general procedure 3 on a 2.788 g (10.58 mmol) scale. Purification by column chromatography (silica gel, 1. dichloromethane, and 2. dichloromethane/acetone 4:1; R_f_ = 0.38) gave the product as a pale brown powder in 35% (1.148 g, 3.64 mmol) yield; m.p. 127–128 °C. ^1^H NMR (400 MHz, DMSO-*d*_6_, 298 K): *δ* = 8.65 (s, 2H), 7.54–7.49 (m, 2H), 7.25–7.20 (m, 3H), 4.23 (s, 2H), and 3.71 (s, 2H); ^13^C{^1^H} NMR (100 MHz, DMSO-*d*_6_, 298 K): *δ* = 162.3 (d, *J*_C–F_ = 247.1 Hz), 158.6, 143.5, 137.3 (d, *J*_C–F_ = 9.1 Hz), 130.1, 129.4 (d, *J*_C–F_ = 9.6 Hz), 128.4 (d, *J*_C–F_ = 2.8 Hz), 127.5, 115.5 (d, *J*_C–F_ = 25.4 Hz), 114.0, 113.3 (d, *J*_C–F_ = 23.0 Hz), 131.8, 55.0, and 36.7. ^19^F{^1^H} NMR (376 MHz, DMSO-*d*_6_, 298 K, referenced to C_6_H_5_F): *δ* = -110.77. HRMS (ESI) *m*/*z:* [M + H]^+^ calculated C_16_H_14_FN_3_OS, 316.0915; observed, 316.0913.

### 3.8. Synthesis and Isolation of 2-(4H-1,2,4-Triazol-4-Yl)benzenethiol (**4a**) for Optimization Reactions and NMR Studies

A 25 mL round-bottomed flask equipped with a stir bar was loaded with 594.8 mg of triazole **3a** (2 mmol, 1 equiv.) and 7.65 mL of trifluoroacetic acid (100 mmol, 50 equiv.). After flushing with argon, 1.09 mL of anisole (10 mmol, 5 equiv.) and 0.99 mL of trifluoromethanesulfonic acid (10 mmol, 5 equiv.) were added to this solution, and the reaction mixture was stirred at 0 °C for 1 h. The solution was then concentrated under high vacuum. The resulting dark red oil was triturated with hexanes and then diluted with 20 mL of water and extracted twice with dichloromethane (50 mL). The combined organic phases were dried over MgSO_4_, filtered, and concentrated. The target compound was obtained as a yellow oil (304.8 mg, 86%) and was used without any further purification. ^1^H NMR (400 MHz, DMSO-*d*_6_, 298 K): *δ* = 8.78 (s, 2H), 7.66 (dd, *J* = 8.0 Hz, *J* = 1.3 Hz, 1H), 7.45–7.41 (m, 2 H), 7.34 (t, *J* = 7.0 Hz, 1H), and 5.79 (s, 1H); LCMS *m*/*z:* [M + H]^+^ calculated C_9_H_7_N_3_O, 178.0; observed, 178.1.

### 3.9. General Procedure 4 for the Synthesis of benzo[4,5]thiazolo[2,3-*c*][1,2,4]triazoles (**6a**–**6s**)

A 25 mL round-bottomed flask equipped with a stir bar was loaded with the triazole (**3a**–**3s**, 1 equiv.) and trifluoroacetic acid (50 equiv.). After flushing with argon, anisole (5 equiv.) and trifluoromethanesulfonic acid (5 equiv.) were added to this solution, and the reaction mixture was stirred at 0 °C for 1 h. The solution was then concentrated under high vacuum. The resulting dark red oil was diluted with 20 mL of water and extracted twice with dichloromethane (50 mL). Following concentration of the combined organic phases, the crude thiol was dissolved in 2 mL of DMSO and heated to 100 C. After completion of the cyclization reaction, the reaction mixture was diluted with 20 mL of sat. NaHCO_3_ and extracted twice with dichloromethane (50 mL). The combined organic phases were dried over MgSO_4_, filtered, and concentrated. The resulting crude product was purified by column chromatography or recrystallization as described below.

#### 3.9.1. benzo[4,5]thiazolo[2,3-*c*][1,2,4]triazole (**6a**)

The title compound was prepared twice according to general procedure 4 on a 297 mg (1.0 mmol) and a 1.49 g (5.00 mmol) scale. Purification by column chromatography (silica gel, 1. dichloromethane, and 2. dichloromethane/acetone 4:1, R_f_ = 0.50) gave the product as a light yellow powder in 85% (149 mg, 0.85 mmol) and 90% (788 mg, 4.5 mmol) yield; m.p. 179–181 °C. ^1^H NMR (400 MHz, DMSO-*d*_6_, 298 K): *δ* = 9.64 (s, 1H), 8.11 (dd, *J* = 8.0 Hz, *J* = 0.5 Hz, 1H), 8.05 (dd, *J* = 8.0 Hz, *J* = 0.4 Hz, 1H), 7.59 (td, *J* = 8.2 Hz, *J* = 1.0 Hz, 1H), and 7.49 (td, *J* = 8.3 Hz, *J* = 1.1 Hz, 1H); ^13^C{^1^H} NMR (100 MHz, DMSO-*d*_6_, 298 K): *δ* = 154.4, 136.8, 131.5, 129.0, 127.0, 126.6, 125.5, and 114.8. HRMS (ESI) *m*/*z:* [M + H] calculated for C_8_H_5_N_3_S, 176.0277; observed, 176.0276.

#### 3.9.2. 8-Methylbenzo[4,5]thiazolo[2,3-*c*][1,2,4]triazole (**6b**)

The title compound was prepared according to general procedure 4 on a 156 mg (0.50 mmol) scale. Purification by column chromatography (silica gel, 1. dichloromethane, and 2. dichloromethane/methanol 5:1, R_f_ = 0.48) gave the product as an off-white powder in 89% (38 mg, 0.445 mmol) yield; m.p. 185–187 °C. ^1^H NMR (400 MHz, DMSO-*d*_6_, 298 K): *δ* = 9.62 (s, 1H), 7.92 (d, *J* = 8.0 Hz, 1H), 7.48 (t, *J* = 7.7 Hz, 1H), 7.32 (d, *J* = 7.6 Hz, 1H), and 2.43 (s, 3H); ^13^C{^1^H} NMR (100 MHz, DMSO-*d*_6_, 298 K): *δ* = 153.6, 136.9, 134.2, 131.0, 128.7, 127.1, 127.0, 122.3, and 19.2. HRMS (ESI) *m*/*z:* [M + H]^+^ calculated for C_9_H_7_N_3_S, 190.0434; observed, 190.0435.

#### 3.9.3. 5-Methylbenzo[4,5]thiazolo[2,3-*c*][1,2,4]triazole (**6c**)

The title compound was prepared according to general procedure 4 on a 311 mg (1.00 mmol) scale. Purification by column chromatography (silica gel, 1. dichloromethane, and 2. dichloromethane/methanol 5:1, R_f_ = 0.46) gave the product as a light yellow powder in 82% (156 mg, 0.825 mmol) yield; m.p. 189–191 °C. ^1^H NMR (400 MHz, DMSO-*d*_6_, 298 K): *δ* = 9.49 (s, 1H), 7.83–7.79 (m, 1H), 7.34 (t, *J* = 5.3 Hz, 1H), and 2.69 (s, 3H); ^13^C{^1^H} NMR (100 MHz, DMSO-*d*_6_, 298 K): *δ* = 154.4, 138.0, 131.3, 128.6, 128.5, 126.1, 125.6, 122.6, and 18.7. HRMS (ESI) *m*/*z:* [M + H]^+^ calculated for C_9_H_7_N_3_S, 190.0434; observed, 190.0433.

#### 3.9.4. Naphtho[2′,1′:4,5]thiazolo[2,3-c][1,2,4]triazole (**6d**)

The title compound was prepared according to general procedure 4 on a 174 mg (0.50 mmol) scale. Purification by column chromatography (silica gel, 1. dichloromethane, and 2. dichloromethane/methanol 5:1, R_f_ = 0.46) gave the product as a light yellow powder in 87% (98 mg, 0.433 mmol) yield; m.p. 238–239 °C. ^1^H NMR (400 MHz, DMSO-*d*_6_, 298 K): *δ* = 9.72 (s, 1H), 8.25 (d, *J* = 8.8 Hz, 1H), 8.15 (t, *J* = 8.6 Hz, 2H), 8.00 (d, *J* = 8.2 Hz, 1H), 7.74 (td, *J* = 7.1 Hz, *J* = 0.9 Hz, 1H), and 7.67 (td, *J* = 8.1 Hz, *J* = 1.0 Hz, 1H); ^13^C{^1^H} NMR (100 MHz, DMSO-*d*_6_, 298 K): *δ* = 159.8, 136.8, 131.1, 129.4, 128.5, 128.1, 128.1, 127.2, 126.9, 126.5, 123.2, and 113.9. HRMS (ESI) *m*/*z:* [M + H]^+^ calculated for C_12_H_7_N_3_S, 226.0434; observed, 266.0433.

#### 3.9.5. N-(benzo[4,5]thiazolo[2,3-*c*][1,2,4]triazol-6-Yl)acetamide (**6e**)

The title compound was prepared according to general procedure 4 on a 354 mg (1.00 mmol) scale. Purification by column chromatography (silica gel, 1. dichloromethane, and 2. dichloromethane/methanol 5:1, R_f_ = 0.05) gave the product as a light yellow powder in 82% (190 mg, 0.82 mmol) yield; m.p. 313–315 °C. ^1^H NMR (400 MHz, DMSO-*d*_6_, 298 K): *δ* = 10.43 (s, 1H), 9.69 (s, 1H), 8.56 (d, *J* = 1.8 Hz, 1H), 7.93 (d, *J* = 8.8 Hz, 1H), 7.44 (dd, *J* = 8.8 Hz, *J* = 2.0 Hz, 1H), and 2.11 (s, 3H); ^13^C{^1^H} NMR (100 MHz, DMSO-d_6_, 298 K): *δ* = 168.8, 160.4, 151.4, 138.4, 136.7, 129.1, 125.5, 117.9, 105.3, and 24.0. HRMS (ESI) *m*/*z:* [M + H]^+^ calculated for C_10_H_8_N_4_OS, 233.0492; observed, 233.0492.

#### 3.9.6. benzo[4,5]thiazolo[2,3-*c*][1,2,4]triazol-6-Amine (**6f**)

The title compound was prepared according to general procedure 4 on a 156 mg (0.50 mmol) scale. Purification by column chromatography (silica gel, 1. dichloromethane, and 2. dichloromethane/methanol 5:1, R_f_ = 0.10) gave the product as a light yellow powder in 60% (114 mg, 0.30 mmol) yield; m.p. 220–222 °C. ^1^H NMR (400 MHz, DMSO-*d_6_*, 298 K): δ = 9.51 (s, 1H), 7.57 (d, *J* = 8.7 Hz, 1H), 7.15 (d, *J* = 2.1 Hz, 1H), 6.71 (dd, *J* = 8.7 Hz, *J* = 2.1 Hz, 1H), and 5.68 (s, 2H); ^13^C{^1^H} NMR (100 MHz, DMSO-*d_6_*, 298 K): δ = 155.2, 148.7, 136.3, 129.8, 125.5, 115.7, 133.7, and 98.9. HRMS (ESI) *m*/*z*: [M + H]^+^ calculated for C_8_H_6_N_4_S, 191.0386; observed, 191.0387.

#### 3.9.7. 6-(4H-1,2,4-Triazol-4-Yl)benzo[4,5]thiazolo[2,3-*c*][1,2,4]triazole (**6f**′)

The title compound was prepared according to general procedure 4 on a 182 mg (0.50 mmol) scale. Purification by column chromatography (silica gel, 1. dichloromethane, and 2. dichloromethane/methanol 5:1, R_f_ = 0.09) gave the product as an off-white powder in 90% (375 mg, 0.471 mmol) yield. ^1^H NMR (400 MHz, DMSO-*d*_6_, 298 K): *δ* = 9.53 (s, 1H), 9.17 (s, 2H), 8.60 (d, *J* = 2.2 Hz, 1H), 8.27 (d, *J* = 8.8 Hz, 1H), and 7.86 (dd, *J* = 8.7 Hz, *J* = 2.2 Hz, 1H); ^13^C{^1^H} NMR was not obtained due to poor solubility. HRMS (ESI) *m*/*z:* [M + H]^+^ calculated for C_10_H_7_N_6_S, 243.0448; observed, 243.0447.

#### 3.9.8. 6-Methoxybenzo[4,5]thiazolo[2,3-*c*][1,2,4]triazole (**6g**)

The title compound was prepared according to general procedure 4 on an 82 mg (0.25 mmol) scale. Purification by column chromatography (silica gel, 1. dichloromethane, and 2. dichloromethane/methanol 5:1, R_f_ = 0.38) gave the product as an off-figurewhite powder in 81% (42 mg, 0.202 mmol) yield; m.p. 223–224 °C. ^1^H NMR (400 MHz, DMSO-*d_6_*, 298 K): δ = 9.57 (s, 1H), 7.89 (d, *J* = 8.9 Hz, 1H), 7.82 (d, *J* = 2.4 Hz, 1H), 7.08 (dd, *J* = 8.9 Hz, *J* = 2.5 Hz, 1H), and 3.86 (s, 3H); ^13^C{^1^H} NMR (100 MHz, DMSO-*d_6_*, 298 K): δ = 158.8, 155.4, 136.5, 129.7, 126.0, 122.1, 114.1, 100.4, and 55.9. HRMS (ESI) *m*/*z*: [M + H]^+^ calculated for C_9_H_7_N_3_OS, 206.0383; observed, 206.0382.

#### 3.9.9. benzo[4,5]thiazolo[2,3-*c*][1,2,4]triazol-6-Ol (**6h**)

The title compound was prepared according to general procedure 4 on a 313 mg (1.00 mmol) scale. Purification by column chromatography (silica gel, 1. dichloromethane, and 2. dichloromethane/methanol 10:1, R_f_ = 0.05) gave the product as an off-white powder in 84% (159.7 mg, 0.84 mmol) yield; m.p. 217–219 °C. ^1^H NMR (400 MHz, DMSO-*d_6_*, 298 K): δ = 9.57 (s, 1H), 7.78 (d, *J* = 8.8 Hz, 1H), 7.50 (d, *J* = 2.3 Hz, 1H), and 6.93 (dd, *J* = 8.8 Hz, *J* = 2.3 Hz, 1H); ^13^C{^1^H} NMR (100 MHz, DMSO-*d_6_*, 298 K): δ = 157.0, 155.3, 136.6, 129.8, 126.0, 120.2, 115.0, and 102.0. HRMS (ESI) *m*/*z*: [M + H]^+^ calculated for C_8_H_4_N_3_OS, 192.0226; observed, 192.0224.

#### 3.9.10. [1,2,4]. triazolo[3′,4′:2,3]thiazolo[4,5-b]pyridine (**6i**)

The title compound was prepared according to general procedure 4 on a 75 mg (0.25 mmol) scale. Purification by column chromatography (silica gel, 1. dichloromethane, and 2. dichloromethane/methanol 5:1, R_f_ = 0.55) gave the product as a light orange powder in 76% (34 mg, 0.190 mmol) yield; m.p. 218–220 °C. ^1^H NMR (400 MHz, DMSO-*d_6_*, 298 K): δ = 9.63 (s, 1H), 8.56–8.52 (m, 2H), and 7.56 (dd, *J* = 8.1 Hz, *J* = 4.9 Hz, 1H); ^13^C{^1^H} NMR (100 MHz, DMSO-*d_6_*, 298 K): δ = 153.8, 146.2, 141.4, 136.1, 135.1, 127.0, and 122.2. HRMS (ESI) *m*/*z*: [M + H]^+^ calculated for C_7_H_4_N_4_S, 177.0230; observed, 177.0230.

#### 3.9.11. [1,2,4]. triazolo[3′,4′:2,3]thiazolo[5,4-b]pyridine (**6j**)

The title compound was prepared according to general procedure 4 on a 75 mg (0.25 mmol) scale. Purification by column chromatography (silica gel, 1. dichloromethane, and 2. dichloromethane/methanol 5:1, R_f_ = 0.55) gave the product as a light orange powder in 70% (31 mg, 0.176 mmol) yield; m.p. 220–221 °C. ^1^H NMR (400 MHz, DMSO-*d_6_*, 298 K): δ = 9.69 (s, 1H), 8.58 (dd, *J* = 4.8 Hz, *J* = 1.4 Hz, 1H), 8.51 (dd, *J* = 8.2 Hz, *J* = 1.4 Hz, 1H), and 7.68 (dd, *J* = 8.2 Hz, *J* = 4.9 Hz, 1H); ^13^C{^1^H} NMR (100 MHz, DMSO-*d_6_*, 298 K): δ = 153.9, 151.1, 147.6, 137.7, 125.4, 122.7, and 122.1. HRMS (ESI) *m*/*z*: [M + H]^+^ calculated for C_7_H_4_N_4_S, 177.0230; observed, 177.0229.

#### 3.9.12. 6-(Trifluoromethyl)benzo[4,5]thiazolo[2,3-*c*][1,2,4]triazole (**6k**)

The title compound was prepared according to general procedure 4 on a 186 mg (0.50 mmol) scale. Purification by column chromatography (silica gel, 1. dichloromethane, and 2. dichloromethane/methanol 5:1, R_f_ = 0.38) gave the product as a light yellow powder in 86% (105 mg, 0.430 mmol) yield; m.p. 203–204 °C. ^1^H NMR (400 MHz, DMSO-*d_6_*, 298 K): δ = 9.69 (s, 1H), 8.63 (s, 1H), 8.28 (d, *J* = 8.5 Hz, 1H), and 7.83 (d, *J* = 8.4 Hz, 1H); ^13^C{^1^H} NMR (100 MHz, DMSO-*d_6_*, 298 K): δ = 154.8, 137.2, 136.6, 129.3, 127.3 (q, *J*_C–F_ = 32.6 Hz), 126.8, 123.9 (q, *J*_C–F_ = 270.6 Hz), 122.9 (q, *J*_C–F_ = 3.7 Hz), and 112. 3 (q, *J*_C–F_ = 4.1 Hz). ^19^F{^1^H} NMR (376 MHz, DMSO-*d_6_*, 298 K, referenced to C_6_H_5_F): δ = −60.85. HRMS (ESI) *m*/*z*: [M + H]^+^ calculated for C_9_H_4_F_3_N_3_S, 244.0151; observed, 244.0151.

#### 3.9.13. benzo[4,5]thiazolo[2,3-*c*][1,2,4]triazole-6-Carbonitrile (**6l**)

The title compound was prepared according to general procedure 4 on a 322 mg (1.00 mmol) scale. Purification by column chromatography (silica gel, 1. dichloromethane, and 2. dichloromethane/methanol 10:1, R_f_ = 0.38) gave the product as a light yellow powder in 80% (159 mg, 0.795 mmol) yield; m.p. 267–269 °C. ^1^H NMR (400 MHz, DMSO-*d_6_*, 298 K): δ = 9.61 (s, 1H), 8.69 (s, 1H), 8.30 (d, *J* = 8.4 Hz, 1H), and 7.95 (d, *J* = 8.0 Hz, 1H); ^13^C{^1^H} NMR (100 MHz, DMSO-*d_6_*, 298 K): δ = 154.7, 137.8, 137.1, 129.6, 129.3, 126.7, 118.6, 118.1, and 108.9. HRMS (ESI) *m*/*z*: [M + H]^+^ calculated for C_9_H_4_N_4_S, 201.0229; observed, 201.0230.

#### 3.9.14. benzo[4,5]thiazolo[2,3-*c*][1,2,4]triazole-6-Carboxylic acid (**6m**)

The title compound was prepared according to general procedure 4 on a 341 mg (1.00 mmol) scale. Purification by column chromatography (silica gel, 1. dichloromethane, and 2. dichloromethane/methanol 5:1, R_f_ = 0.38) gave the product as a white powder in 85% (186 mg, 0.847 mmol) yield; m.p. > 360 °C. ^1^H NMR (400 MHz, DMSO-*d_6_*, 298 K): δ = 9.78 (s, 1H), 8.74 (d, *J* = 1.1 Hz, 1H), 8.16 (d, *J* = 8.4 Hz, 1H), and 8.03 (dd, *J* = 8.5 Hz, *J* = 1.6 Hz, 1H); ^13^C{^1^H} NMR (100 MHz, DMSO-*d_6_*, 298 K): δ = 167.1, 158.0, 152.9, 138.3, 131.8, 129.1, 125.7, 123.9, and 113.8. HRMS (ESI) *m*/*z*: [M + H]^+^ calculated for C_9_H_5_N_3_O_2_S, 220.0175; observed, 220.0176.

#### 3.9.15. Ethyl benzo[4,5]thiazolo[2,3-*c*][1,2,4]triazole-6-Carboxylate (**6m**′)

The title compound was prepared according to general procedure 4 on a 341 mg (1.00 mmol) scale. Purification by column chromatography (silica gel, 1. dichloromethane, and 2. dichloromethane/methanol 5:1, R_f_ = 0.38) gave the product as a light yellow powder in 86% (173 mg, 0.865 mmol) yield; m.p. 209–210 °C. ^1^H NMR (400 MHz, DMSO-*d_6_*, 298 K): δ = 9.80 (s, 1H), 8.73 (s, 1H), 8.19 (d, *J* = 8.5 Hz, 1H), 8.04 (d, *J* = 8.4 Hz, 1H), 4.39 (q, *J* = 7.1 Hz, 2H), 3.86 (s, 3H), and 1.37 (t, *J* = 7.1 Hz, 3H); ^13^C{^1^H} NMR (100 MHz, DMSO-*d_6_*, 298 K): δ = 164.8, 137.2, 129.3, 128.5, 126.9, 125.7, 115.5, 61.4, and 14.2. HRMS (ESI) *m*/*z*: [M + H]^+^ calculated for C_11_H_9_N_3_O_2_S, 248.0488; observed, 248.0490.

#### 3.9.16. 6-Bromobenzo[4,5]thiazolo[2,3-*c*][1,2,4]triazole (**6n**)

The title compound was prepared according to general procedure 4 on a 118 mg (0.50 mmol) scale. Purification by column chromatography (silica gel, 1. dichloromethane, and 2. dichloromethane/methanol 5:1, R_f_ = 0.66) gave the product as an off-white powder in 88% (112 mg, 0.442 mmol) yield; m.p. 240–243 °C. ^1^H NMR (400 MHz, DMSO-*d_6_*, 298 K): δ = 9.59 (s, 1H), 8.47 (s, 1H), 8.01 (d, *J* = 8.4 Hz, 1H), and 8.67 (d, *J* = 8.3 Hz, 1H); ^13^C{^1^H} NMR (100 MHz, DMSO-*d_6_*, 298 K): δ = 154.8, 136.9, 131.1, 130.0, 129.2, 127.2, 119.2, and 118.0. HRMS (ESI) *m*/*z*: [M + H]^+^ calculated for C_8_H_4_BrN_3_S, 253.9382; observed, 253.9381.

#### 3.9.17. 7-Bromobenzo[4,5]thiazolo[2,3-*c*][1,2,4]triazole (**6o**)

The title compound was prepared twice according to general procedure 4 on a 376 mg (1.0 mmol) scale and a 3.76 g (10 mmol) scale. The title compound was triturated with acetone to afford an off-white powder in 90% (229 mg, 0.90 mmol) and 92% (2.34 g, 9.2 mmol) yield; m.p. 273–275 °C. ^1^H NMR (400 MHz, DMSO-*d_6_*, 298 K): δ = 9.65 (s, 1H), 8.35 (d, *J* = 1.9 Hz, 1H), 8.08 (t, *J* = 8.6 Hz, 1H), and 7.79 (dd, *J* = 8.6 Hz, *J* = 2.0 Hz, 1H); ^13^C{^1^H} NMR (100 MHz, DMSO-*d_6_*, 298 K): δ = 154.5, 134.0, 133.8, 129.9, 128.4, 127.8, 118.3, and 116.5. HRMS (ESI) *m*/*z*: [M + H]^+^ calculated for C_8_H_4_BrN_3_S, 253.9382; observed, 253.9381.

#### 3.9.18. 6-Chlorobenzo[4,5]thiazolo[2,3-*c*][1,2,4]triazole (**6p**)

The title compound was prepared according to general procedure 4 on a 166 mg (0.50 mmol) scale. Purification by column chromatography (silica gel, 1. dichloromethane, and 2. dichloromethane/methanol 5:1, R_f_ = 0.38) gave the product as an off-white powder in 91% (99 mg, 0.457 mmol) yield; m.p. 229–231 °C. ^1^H NMR (400 MHz, DMSO-*d_6_*, 298 K): δ = 9.59 (s, 1H), 8.36 (d, *J* = 1.8 Hz, 1H), 8.08 (d, *J* = 8.6 Hz, 1H), and 7.56 (dd, *J* = 8.7 Hz, *J* = 2.0 Hz, 1H); ^13^C{^1^H} NMR (100 MHz, DMSO-*d_6_*, 298 K): δ = 154.7, 137.4, 131.8, 131.0, 130.3, 127.4, 127.0, and 115.7. HRMS (ESI) *m*/*z*: [M + H]^+^ calculated for C_8_H_4_ClN_3_S, 209.9887; observed, 209.9887.

#### 3.9.19. 8-Chlorobenzo[4,5]thiazolo[2,3-*c*][1,2,4]triazole (**6q**)

The title compound was prepared according to general procedure 4 on a 166 mg (1.00 mmol) scale. Purification by column chromatography (silica gel, 1. dichloromethane, and 2. dichloromethane/methanol 5:1, R_f_ = 0.36) gave the product as an off-white powder in 90% (93.8 mg, 0.448 mmol) yield; m.p. 249–251 °C. ^1^H NMR (400 MHz, DMSO-*d_6_*, 298 K): δ = 9.68 (s, 1H), 8.14–8.10 (m, 1H), and 7.66–7.64 (m, 2H); ^13^C{^1^H} NMR (100 MHz, DMSO-*d_6_*, 298 K): δ = 153.0, 137.4, 130.8, 130.2, 128.6, 127.7, 126.3, and 113.8. HRMS (ESI) *m*/*z*: [M + H]^+^ calculated for C_8_H_4_ClN_3_S, 209.9887; observed, 209.9886.

#### 3.9.20. 8-Fluorobenzo[4,5]thiazolo[2,3-*c*][1,2,4]triazole (**6r**)

The title compound was prepared according to general procedure 4 on a 315 mg (1.00 mmol) scale. Purification by column chromatography (silica gel, 1. dichloromethane, and 2. dichloromethane/methanol 5:1, R_f_ = 0.40) gave the product as a light yellow powder in 98% (188 mg, 0.975 mmol) yield; m.p. 211–212 °C. ^1^H NMR (400 MHz, DMSO-*d_6_*, 298 K): δ = 9.69 (s, 1H), 7.99 (d, *J* = 8.1 Hz, 1H), 7.69–7.63 (m, 1H), and 7.46 (t, *J* = 7.6 Hz, 1H); ^13^C{^1^H} NMR (100 MHz, DMSO-*d_6_*, 298 K): δ = 156.5 (d, *J*_C–F_ = 244.7 Hz), 153.9, 137.3, 130.8 (d, *J*_C–F_ = 6.8 Hz), 129.0 (d, *J*_C–F_ = 7.8 Hz), 118.1 (d, *J*_C–F_ = 22.3 Hz), 112.9 (d, *J*_C–F_ = 18.1 Hz), and 111.4 (d, *J*_C–F_ = 3.4 Hz). ^19^F{^1^H} NMR (376 MHz, DMSO-*d_6_*, 298 K, referenced to C_6_H_5_F): δ = −114.97. HRMS (ESI) *m*/*z*: [M + H]^+^ calculated for C_8_H_4_FN_3_S, 194.0183; observed, 194.0182.

#### 3.9.21. 7-Fluorobenzo[[1,3]4,5]thiazolo[2,3-c][1,2,4]triazole (**6s**)

The title compound was prepared according to general procedure 4 on a 315 mg (1.00 mmol) scale. Purification by column chromatography (silica gel, 1. dichloromethane, and 2. dichloromethane/methanol 5:1, R_f_ = 0.38) gave the product as an off-white powder in 98% (188 mg, 0.976 mmol) yield; m.p. 255–258 °C. ^1^H NMR (400 MHz, DMSO-*d*_6_, 298 K): *δ* = 9.59 (s, 1H), 8.14 (dd, *J* = 8.9 Hz, *J* = 4.6 Hz, 1H), 8.01 (dd, *J* = 8.8 Hz, *J* = 2.6 Hz, 1H), and 7.47 (td, *J* = 9.0 Hz, *J* = 2.6 Hz, 1H); ^13^C{^1^H} NMR (100 MHz, DMSO-*d_6_*, 298 K): *δ* = 159.7 (d, *J*_C–F_ = 242.0 Hz), 154.6, 136.8, 133.3 (q, *J*_C–F_ = 11.0 Hz), 125.9 (q, *J*_C–F_ = 2.1 Hz), 116.1 (d, *J*_C–F_ = 9,4 Hz), 114.5 (d, *J*_C–F_ = 24.7 Hz), and 112.5 (d, *J*_C–F_ = 28.2 Hz). ^19^F{^1^H} NMR (376 MHz, DMSO-*d*_6_, 298 K, referenced to C_6_H_5_F): *δ* = −114.27. HRMS (ESI) *m*/*z:* [M + H]^+^ calculated for C_8_H_4_FN_3_S, 194.0183; observed, 194.0183.

## 4. Conclusions

In summary, a novel and efficient method for the synthesis of substituted benzo[4,5]thiazolo[2,3-*c*][1,2,4]triazoles species (**6a**–**6s**) from the corresponding *p*-methoxybenzyl-protected 4-(2-mercaptophenyl)triazoles (**3a**–**3s**) has been developed. Following the selective removal of the protecting group, the free thiols (**4a**–**4b**) are oxidized to their corresponding disulfides (**5a**–**5s**). These disulfides are thought to undergo C-H bond functionalization, thus leading to an intramolecular ring closure, thereby forming the C-S bond of the target heterocycle. Our synthetic approach allows the preparation of benzo[4,5]thiazolo[2,3-*c*][1,2,4]triazoles (**6a**–**6s**) containing synthetically valuable functional groups on their benzene rings. The combination of short reaction times and good to excellent isolated yields, regardless of the nature of the substituents, is a clear advantage of this scalable reaction protocol.

## Figures and Tables

**Table 1:** Optimization of cyclization conditions *^a^*.

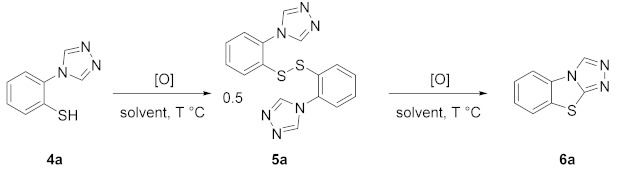						
Entry	Oxidant	Solvent	T (°C)	Time (h)	Outcome	Yield (%) *^b^*
1	1 equiv. H_2_O_2_	50% aq. Ethanol	RT	16	**5a**	0
2	1 equiv. H_2_O_2_	50% aq. Ethanol	80	16	**6a**	78
3	5 equiv. H_2_O_2_	Ethanol	80	16	**6a**	67
4	1 equiv. H_2_O_2_	10% aq. NaOH	RT	16	**5a**	0
5	DMSO	DMSO	RT	16	**5a**	0
6	DMSO	DMSO	100	4	**6a**	83
7	1 equiv. I_2_	DMSO	RT	16	**5a**	0
8	1 equiv. I_2_	DMSO	100	4	**6a**	82

*^a^* Reaction conditions: argon atmosphere, thiol **4a** (0.5 mmol), solvent (2 mL), oxidant, solvent, temperature, and time. *^b^*Isolated product **6a** after column chromatography.

## Data Availability

The data presented in this study are available in supplementary material.

## Supplementary Materials

The following supporting information can be downloaded, Copies of the ^1^H NMR and ^13^C NMR spectra for compounds 1b–1r, 2b–2s, 3b–3s, and 6a–6s.

## Sample Availability

Samples of the compounds are not available from the authors.
