# Supplementary material for: Synthesis of Benzo[4,5]thiazolo[2,3-c][1,2,4]triazole Derivatives via C-H Bond Functionalization of Disulfide Intermediates
Source: Molecules. 2022 Feb 22;27(5):1464. doi: 10.3390/molecules27051464 (PMC8911890; doi:10.3390/molecules27051464)
Supplement: Supplementary file 1 [file molecules-27-01464-s001.zip › molecules-1601400-supplementary.pdf]

# Synthesis of Benzo[4,5]thiazolo[2,3-*c*][1,2,4]triazole Derivatives via C-H Bond Functionalization of Disulfide Intermediates

Luis G. Ardón-Muñoz, Jeanne L. Bolliger*

Department of Chemistry, 107 Physical Sciences, Oklahoma State University, Stillwater, OK, 74078, United States.

Corresponding Author: jeanne.bolliger@okstate.edu

Table of Contents

Page

1. NMR Spectra of Isolated Compounds ........................................................ S3

1.1 NMR Spectra of 2-Fluoronitrobenzene Starting Materials ............................ S3

1.2 NMR Spectra of Substituted (4-Methoxybenzyl)(2-nitrophenyl)sulfanes (**1b**-**1s**) ........................................................................................................ S6

1.3 NMR Spectra of Substituted 2-((4-Methoxybenzyl)thio)anilines (**2b**-**2s**)... S25

1.4 NMR Spectra of Substituted 4-(2-((4-Methoxybenzyl)thio)phenyl)-4*H*-1,2,4-triazoles (**3b**-**3s**) ............................................................................. S46

1.5 NMR Spectra of Benzo[4,5]thiazolo[2,3-*c*][1,2,4]triazoles (**6a**-**6s**) ............ S68

# 1. NMR Spectra of Isolated Compounds

## *1.1 NMR Spectra of 2-Fluoronitrobenzene Starting Materials*

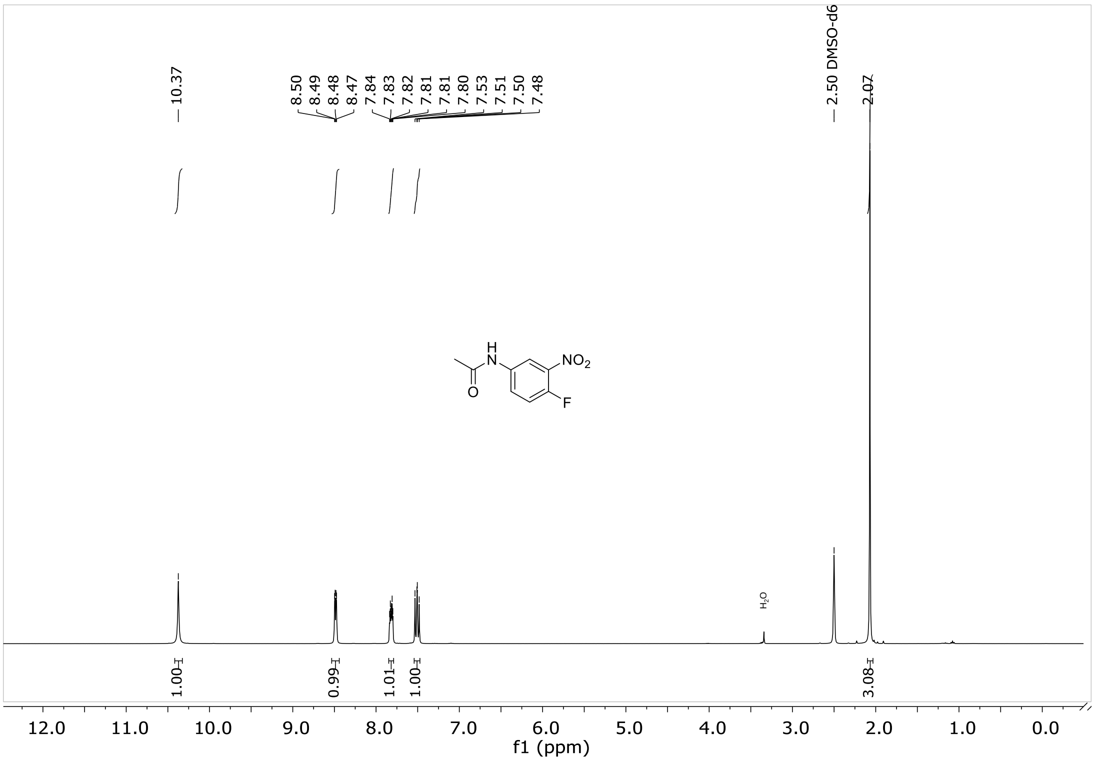


**Figure S001**: $^{1}H$ NMR spectrum of *N*-(4-Fluoro-3-nitrophenyl)acetamide (400 MHz, DMSO-$d_6$, 298 K).

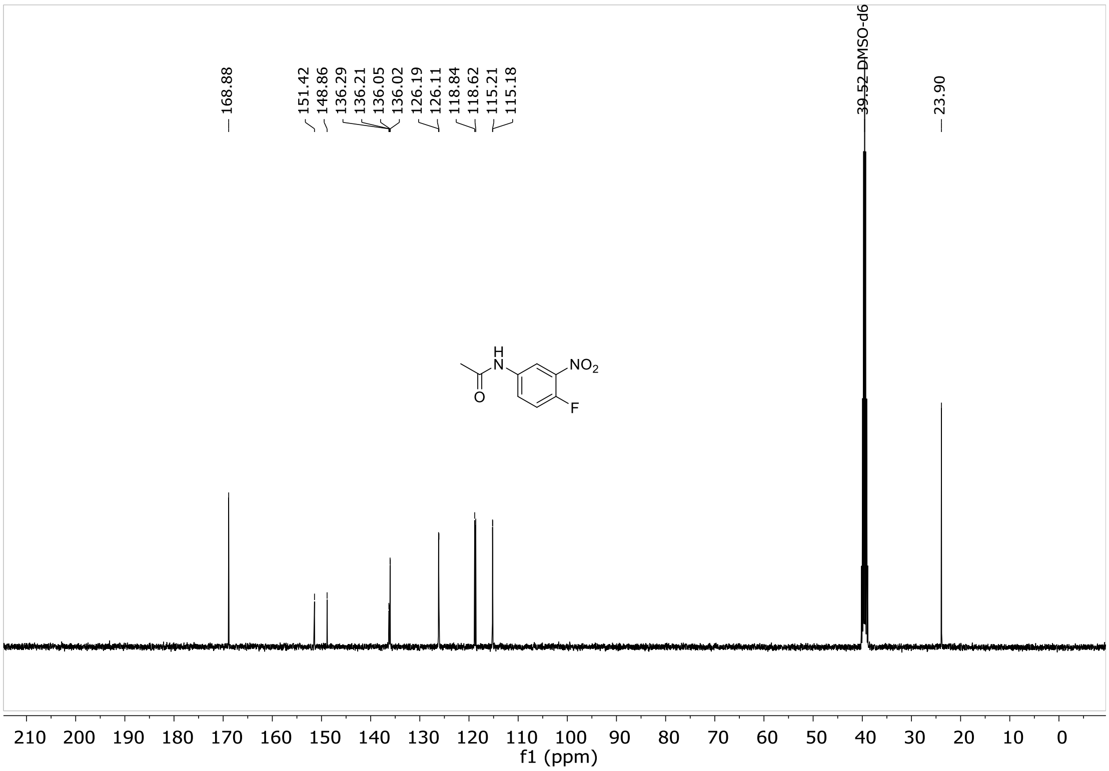


**Figure S002**: $^{13}C\{^{1}H\}$ NMR spectrum of *N*-(4-Fluoro-3-nitrophenyl)acetamide (100 MHz, DMSO-$d_6$, 298 K).

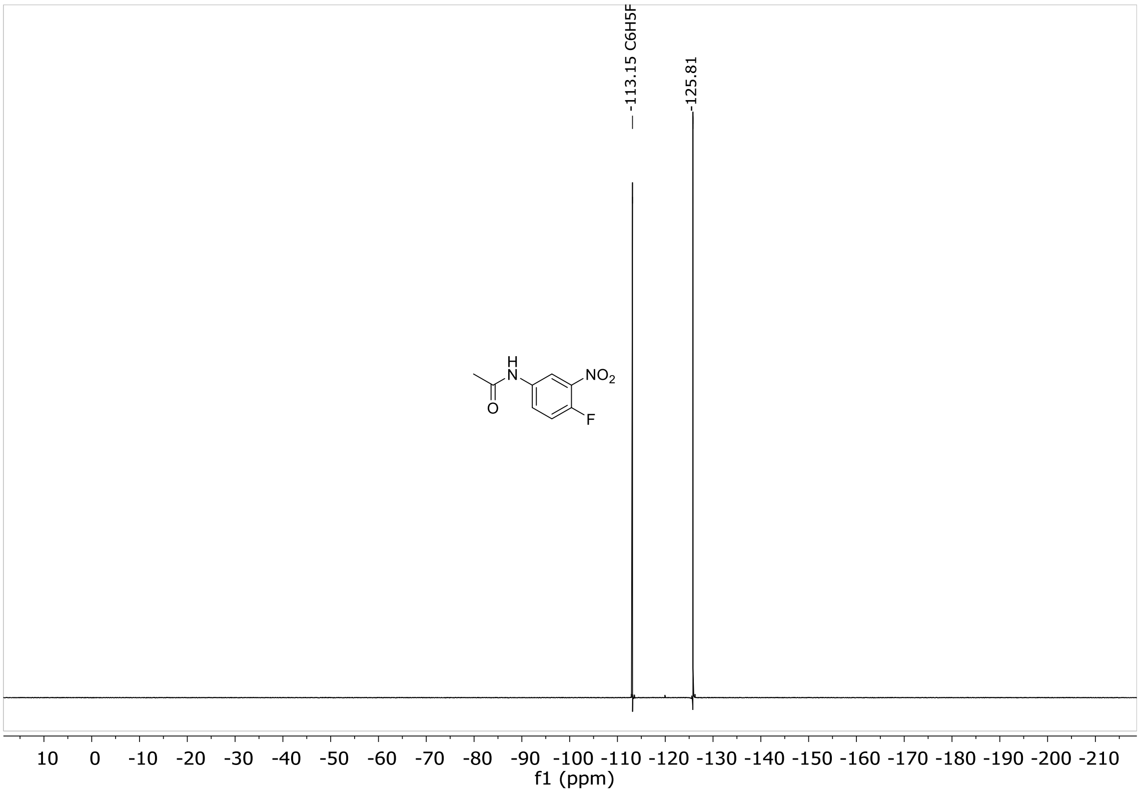


**Figure S003**: $^{19}F\{^1H\}$ NMR spectrum of *N*-(4-Fluoro-3-nitrophenyl)acetamide (376 MHz, DMSO-$d_6$, 298 K, referenced to fluorobenzene).

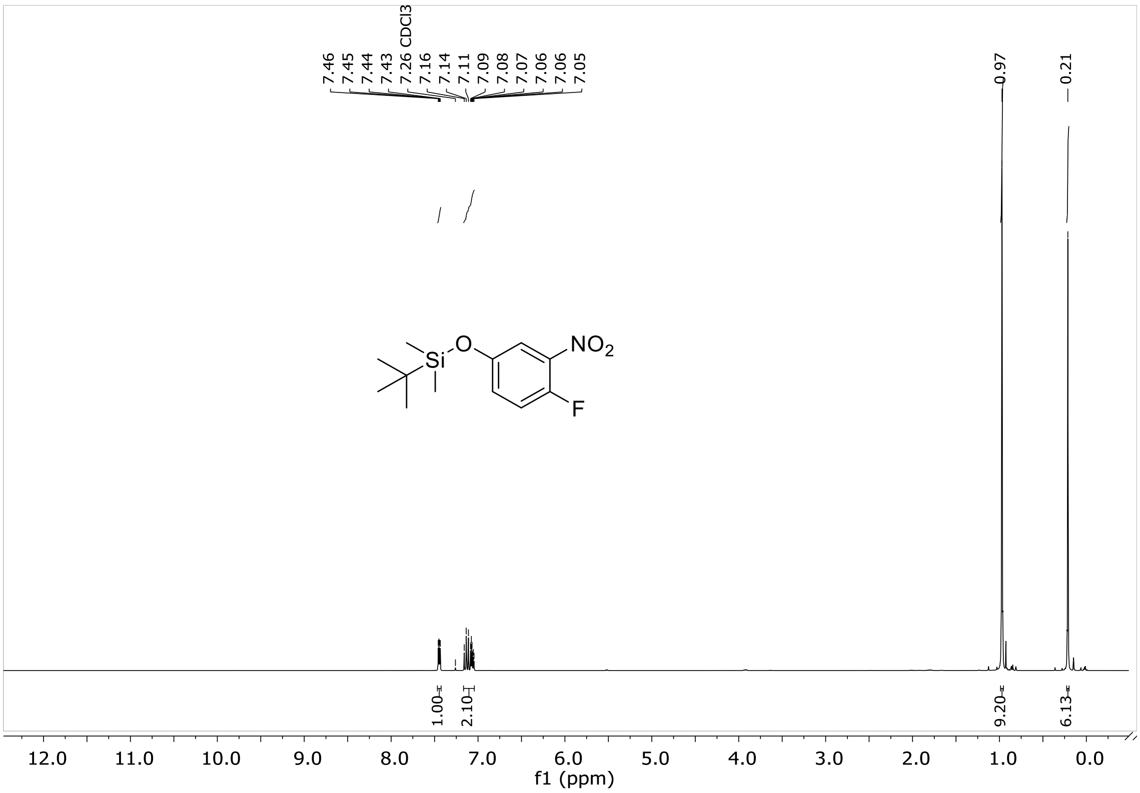


**Figure S004**: $^1H$ NMR spectrum of *tert*-butyl(4-fluoro-3-nitrophenoxy)dimethylsilane (400 MHz, $CDCl_3$, 298 K).

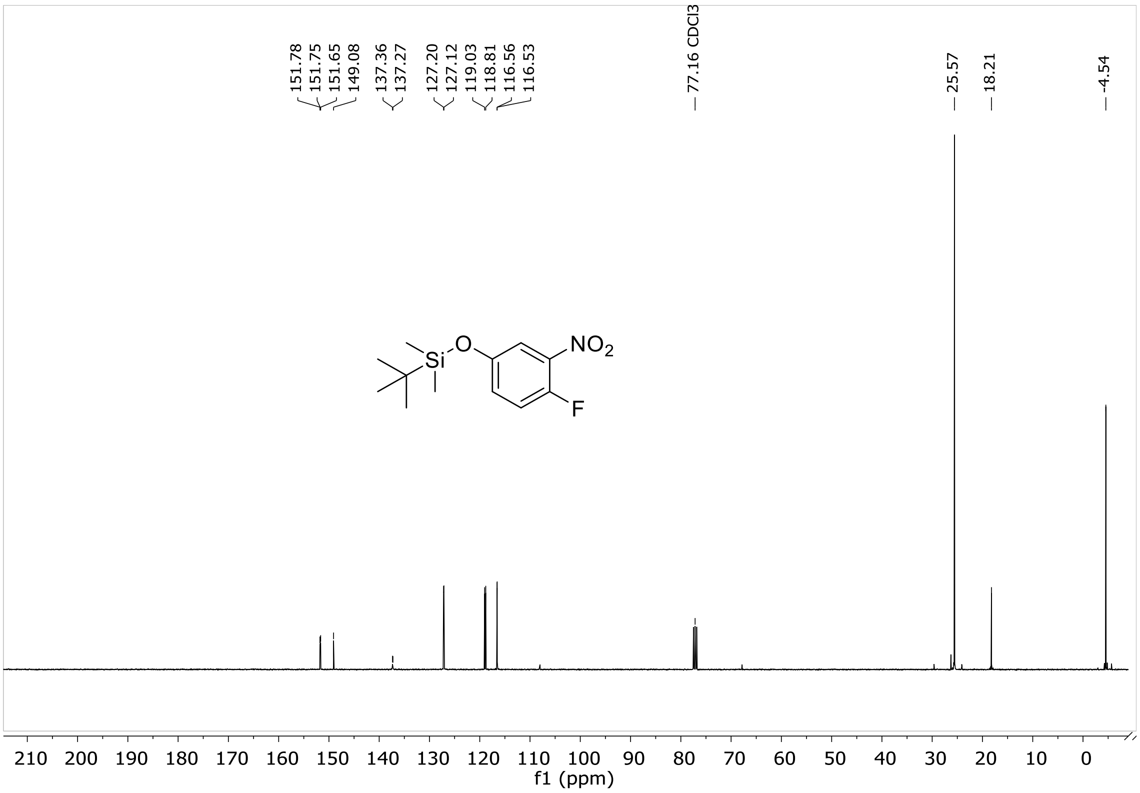


**Figure S005**: $^{13}C\{^{1}H\}$ NMR spectrum of *tert*-butyl(4-fluoro-3-nitrophenoxy)dimethylsilane (100 MHz, $CDCl_3$, 298 K).

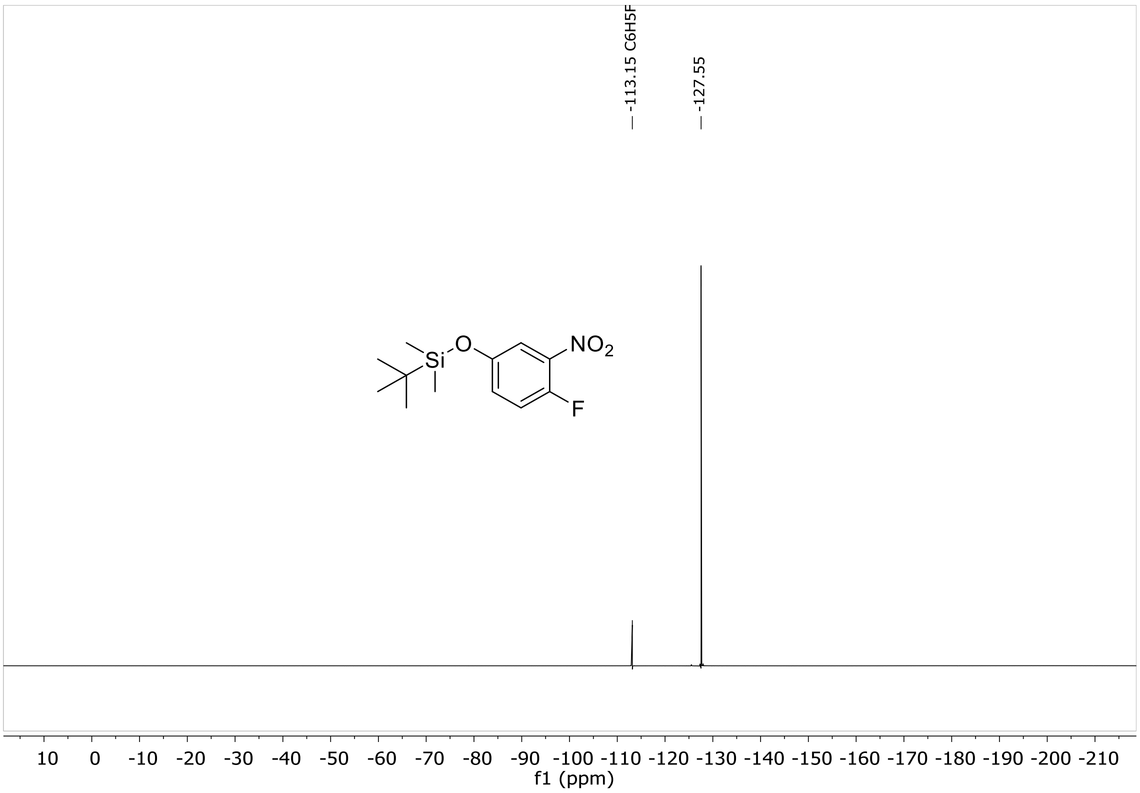


**Figure S006**: $^{19}F\{^{1}H\}$ NMR spectrum of *tert-b*utyl(4-fluoro-3-nitrophenoxy)dimethylsilane (376 MHz, $CDCl_3$, 298 K, referenced to fluorobenzene).

## ***1.2 NMR Spectra of Substituted (4-Methoxybenzyl)(2-nitrophenyl)sulfanes (1b-1r)***

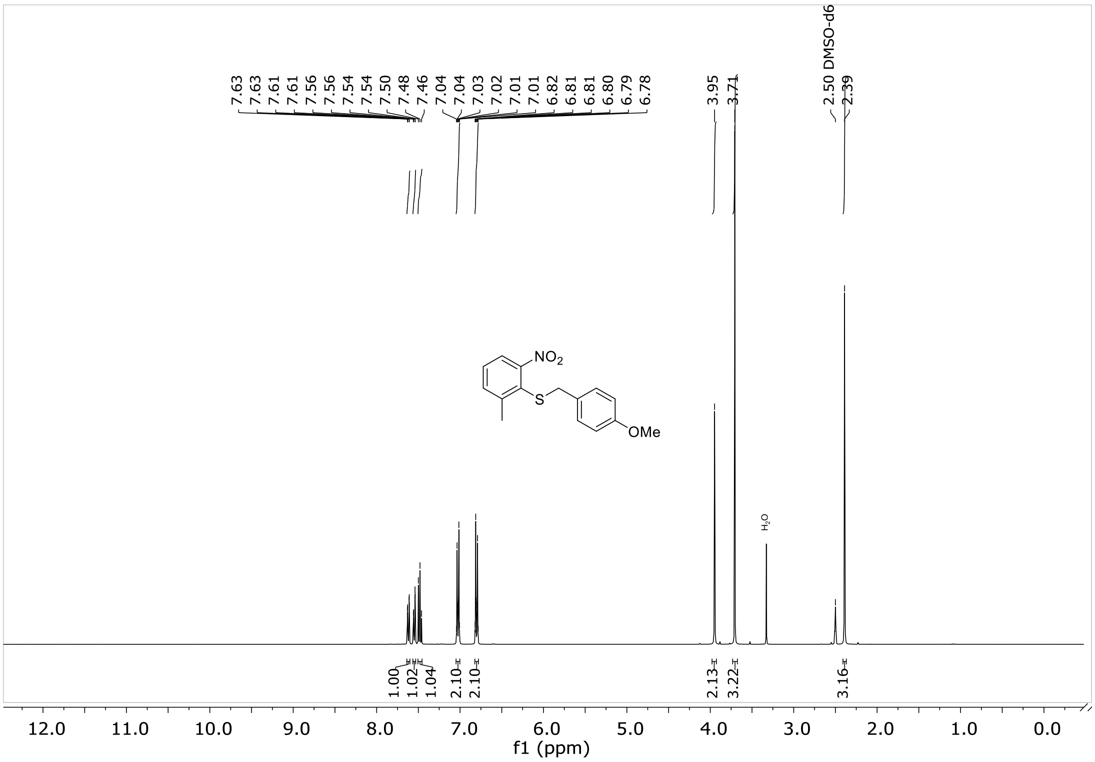


**Figure S007**: $^{1}H$ NMR spectrum of (4-methoxybenzyl)(2-methyl-6-nitrophenyl)sulfane (**1b**) (400 MHz, DMSO-*d*$_6$, 298 K).

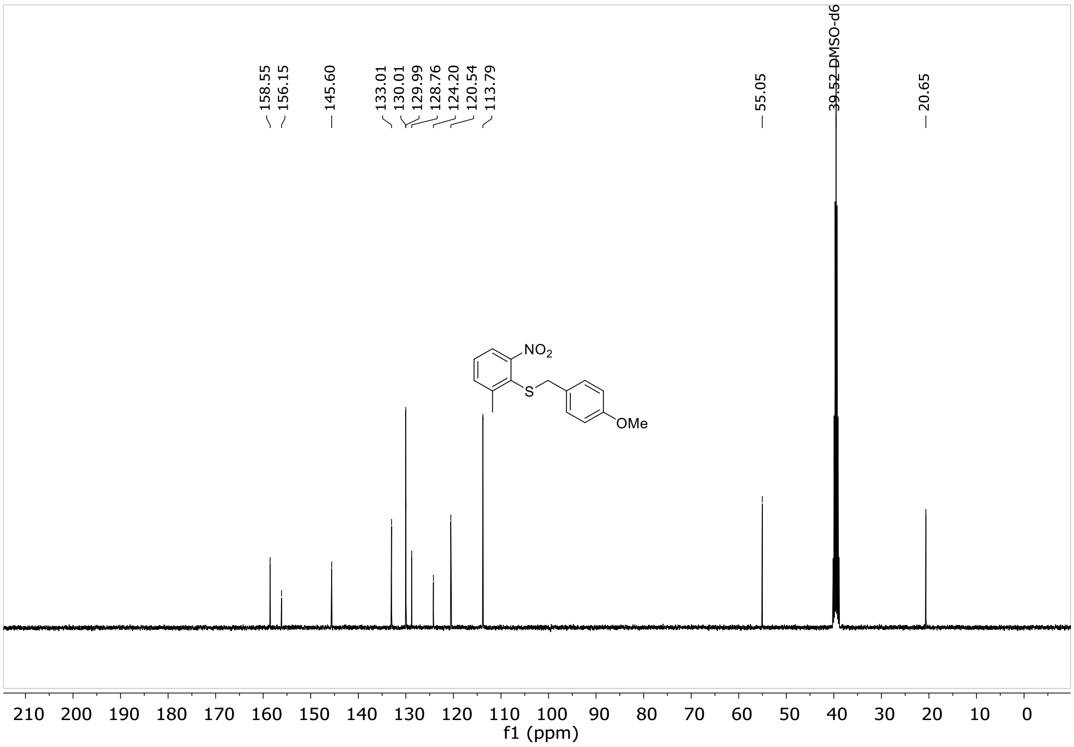


**Figure S008**: $^{13}C\{^{1}H\}$ NMR spectrum of (4-methoxybenzyl)(2-methyl-6-nitrophenyl)sulfane (**1b**) (100 MHz, DMSO-*d*$_6$, 298 K).

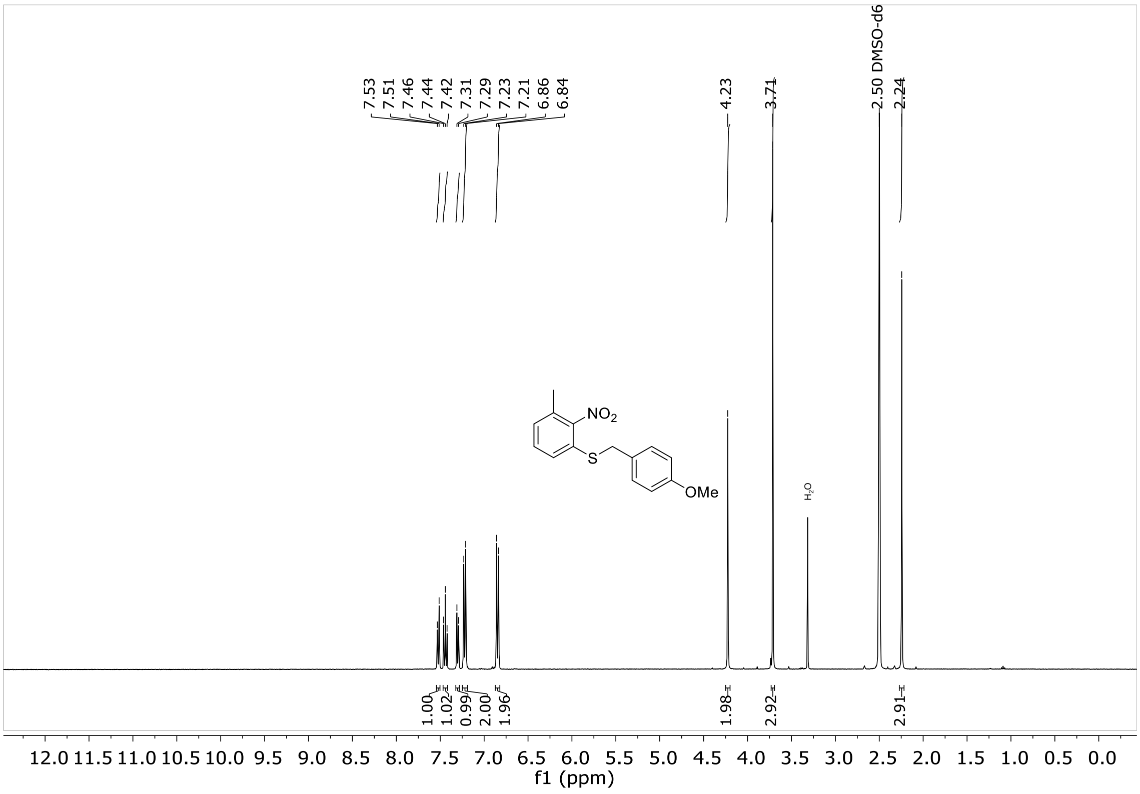


**Figure S009**: $^{1}$H NMR spectrum of (4-methoxybenzyl)(3-methyl-2-nitrophenyl)sulfane (**1c**) (400 MHz, DMSO-$d_6$, 298 K).

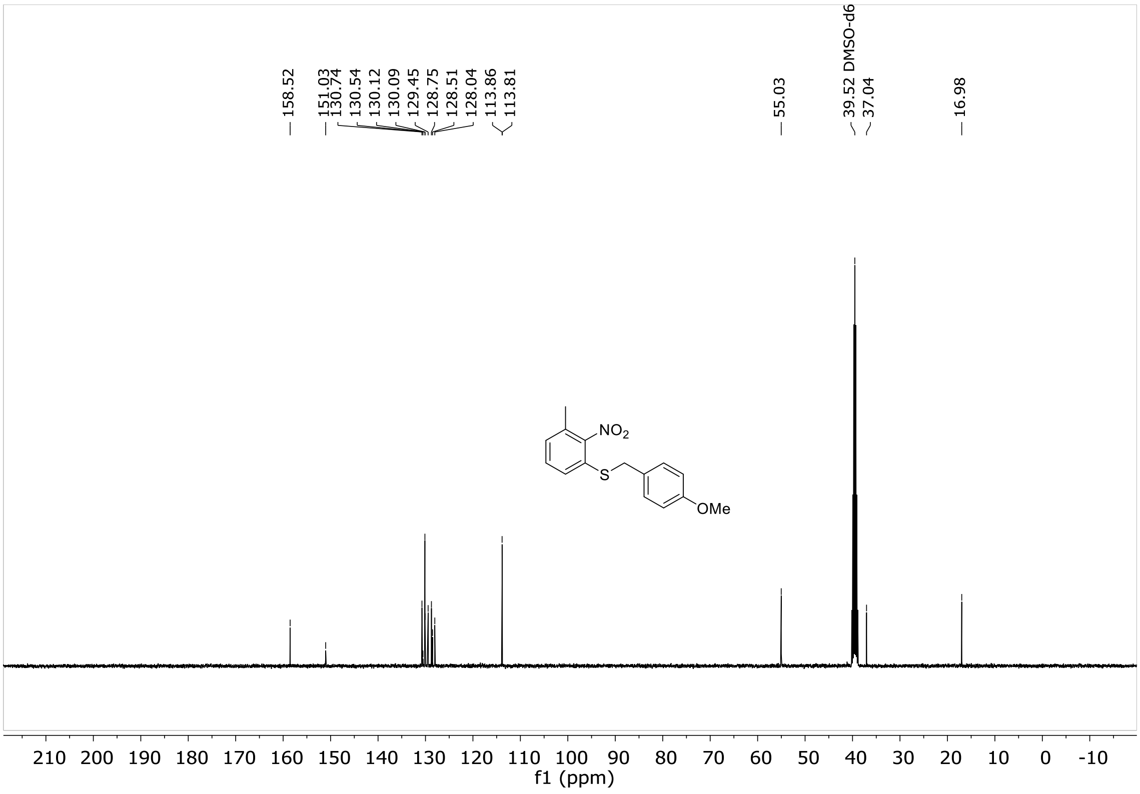


**Figure S010**: $^{13}$C{$^{1}$H}NMR spectrum of (4-methoxybenzyl)(3-methyl-2-nitrophenyl) (**1c**) (100 MHz, DMSO-$d_6$, 298 K).

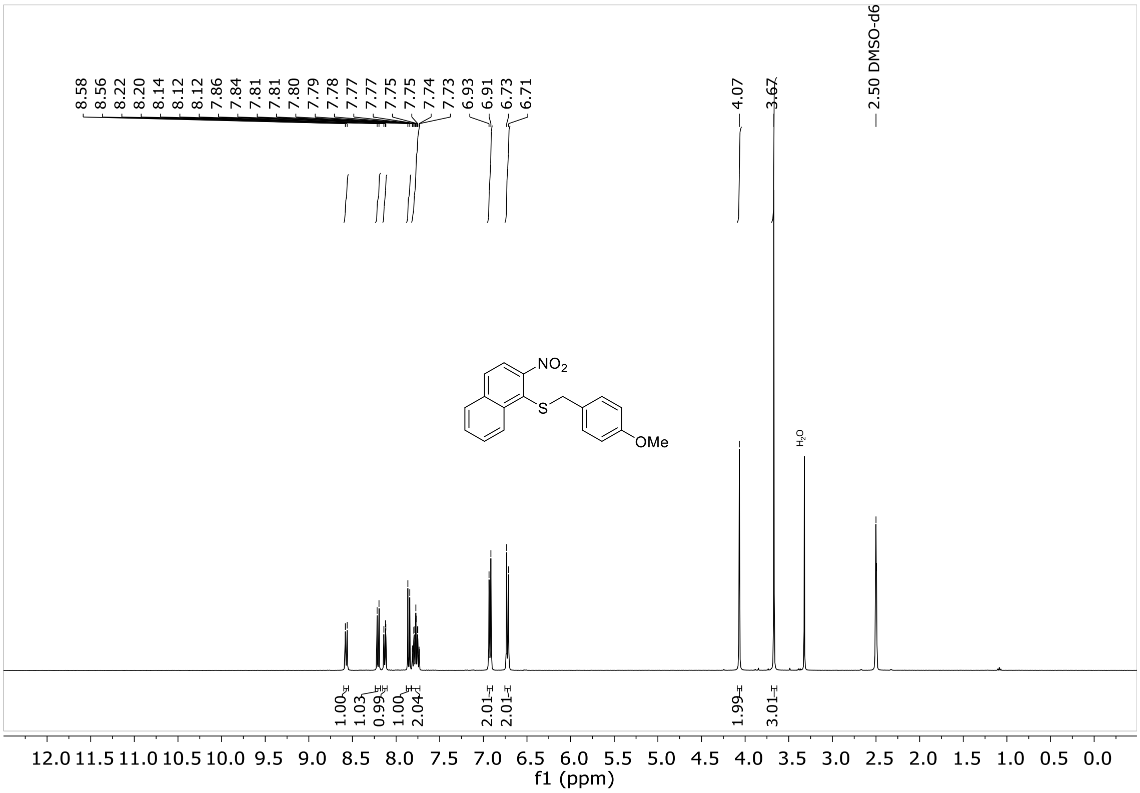

**Figure S011**: $^1$H NMR spectrum of (4-methoxybenzyl)(2-nitronaphthalen-1-yl)sulfane (**1d**) (400 MHz, DMSO-*d*$_6$, 298 K).

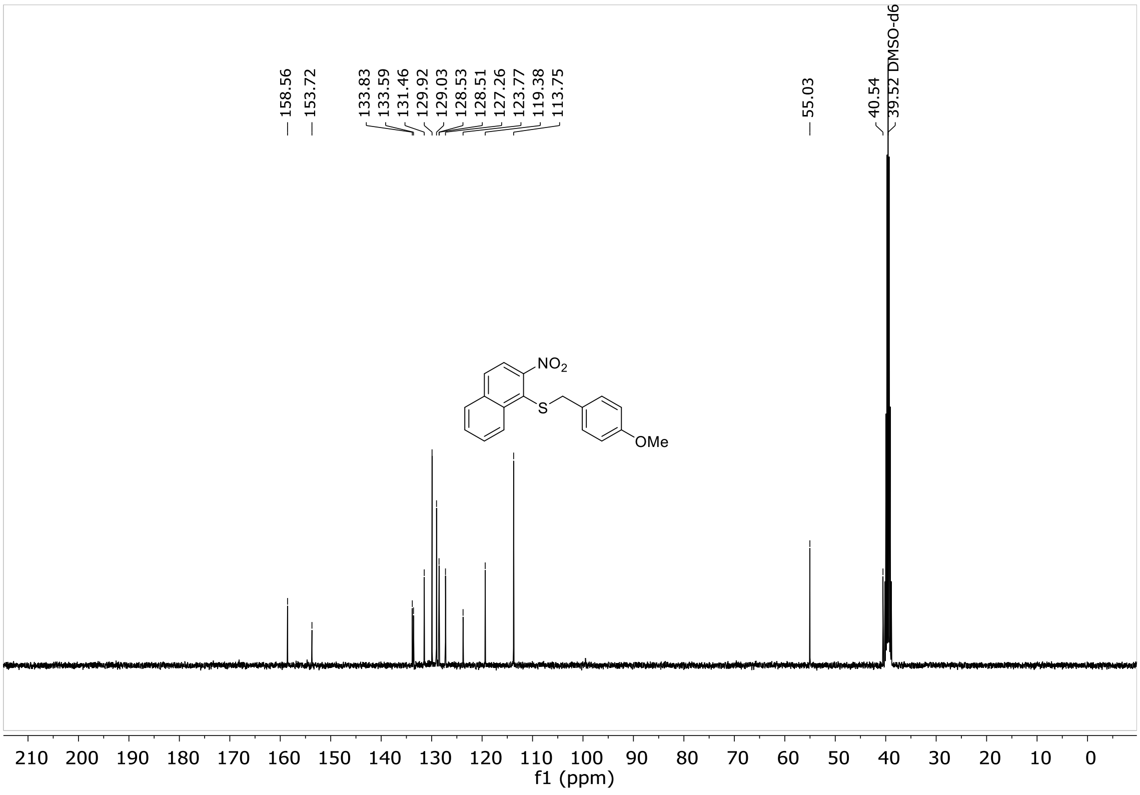

**Figure S012**: $^{13}$C{$^1$H} NMR spectrum of (4-methoxybenzyl)(2-nitronaphthalen-1-yl)sulfane (**1d**) (100 MHz, DMSO-*d*$_6$, 298 K).

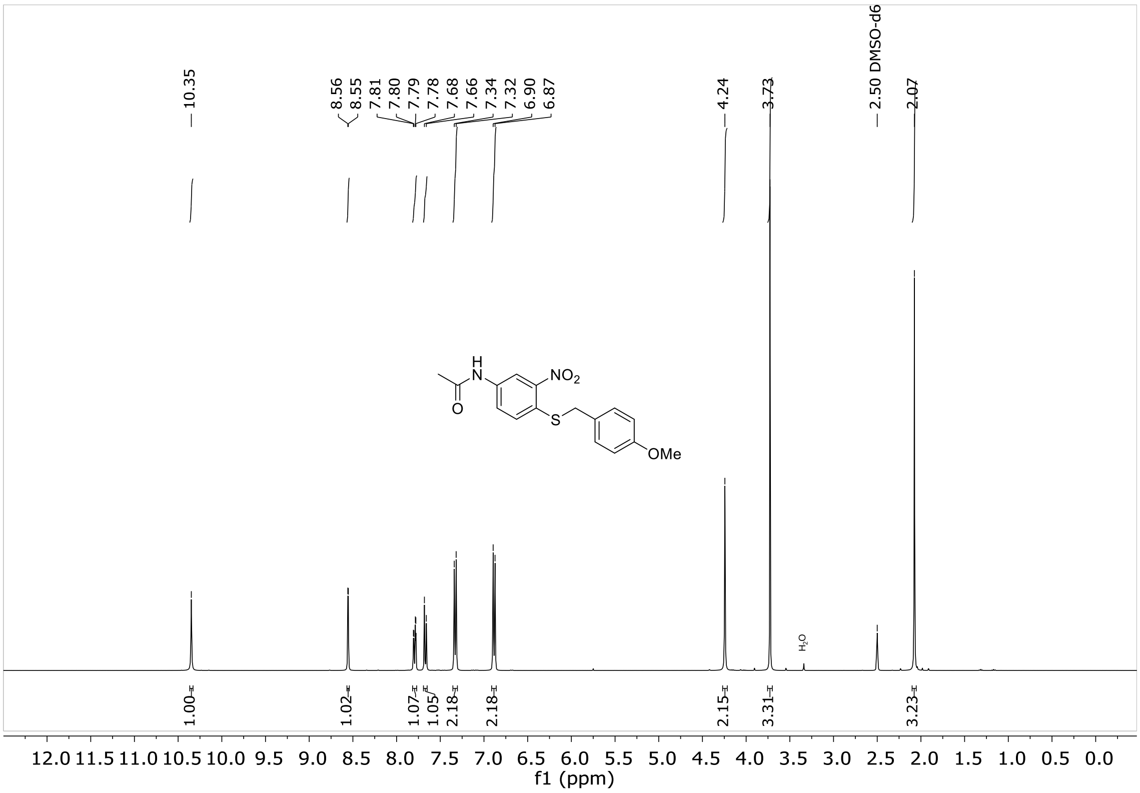


**Figure S013**: $^{1}$H NMR spectrum of *N*-(4-((4-methoxybenzyl)thio)-3-nitrophenyl)acetamide (**1f**) (400 MHz, DMSO-$d_6$, 298 K).

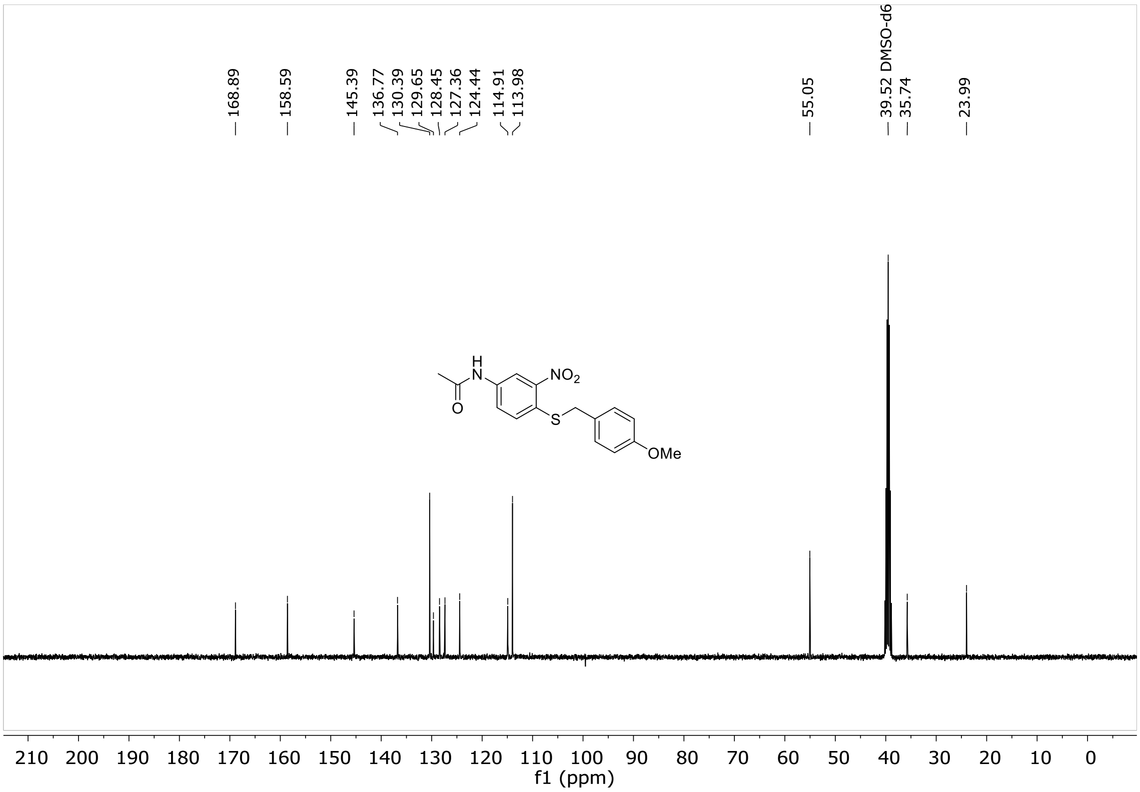


**Figure S014**: $^{13}$C{$^{1}$H} NMR spectrum of *N*-(4-((4-methoxybenzyl)thio)-3-nitrophenyl)acetamide (**1f**) (100 MHz, DMSO-$d_6$, 298 K).

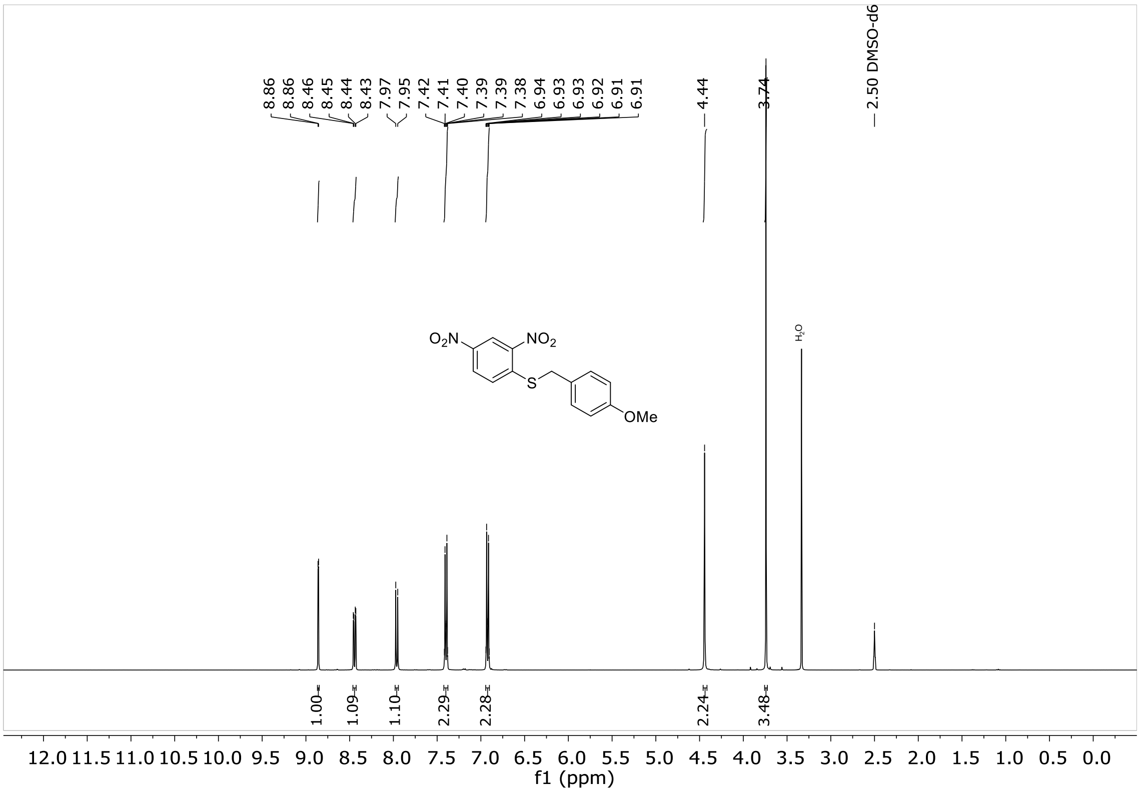


**Figure S0015**: $^{1}H$ NMR spectrum of (2,4-dinitrophenyl)(4-methoxybenzyl)sulfane (**1e**) (400 MHz, DMSO-$d_6$, 298 K).

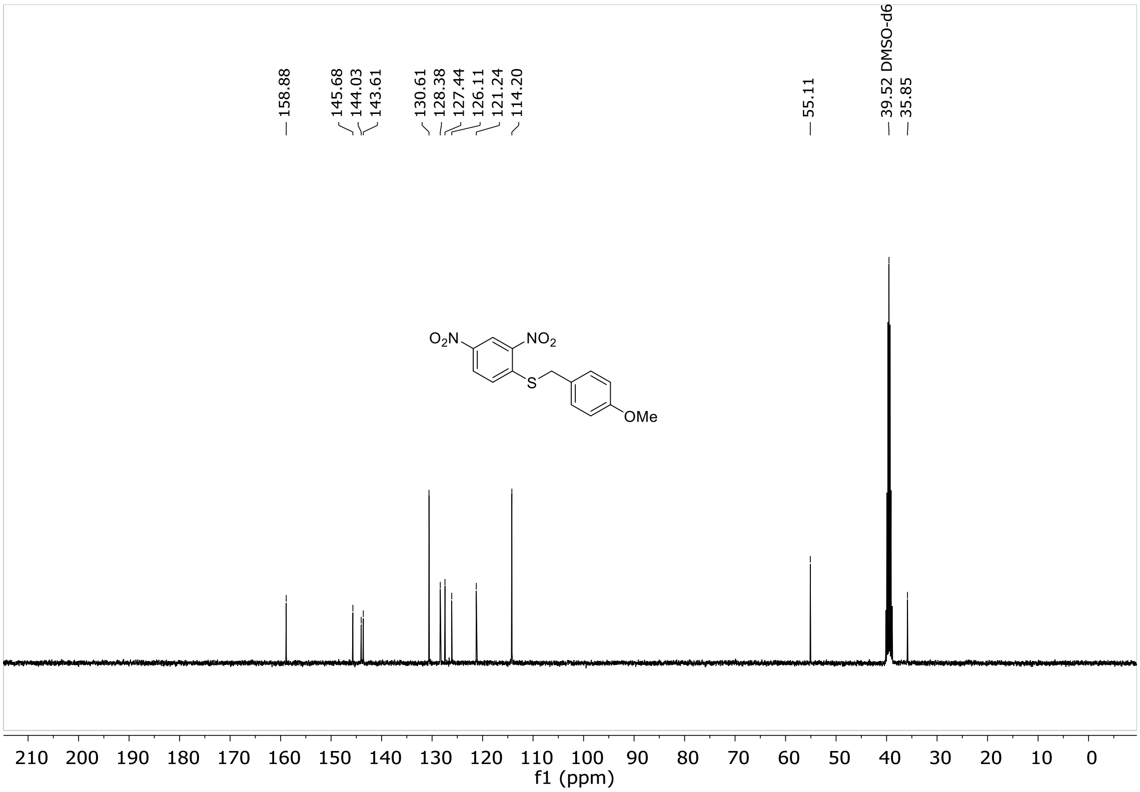


**Figure S016**: $^{13}C\{^{1}H\}$ NMR spectrum of (2,4-dinitrophenyl)(4-methoxybenzyl)sulfane (**1e**) (100 MHz, DMSO-$d_6$, 298 K).

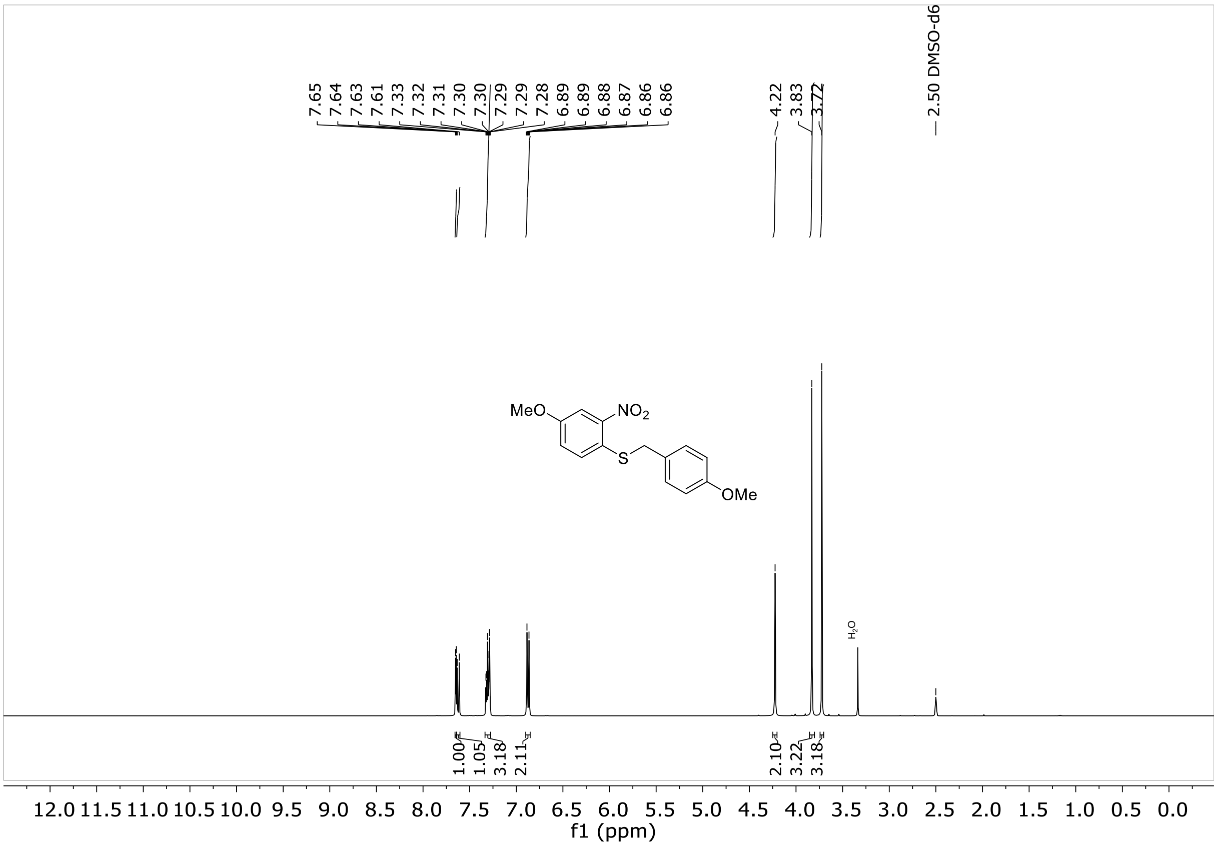


**Figure S017**: $^{1}H$ NMR spectrum of (4-methoxy-2-nitrophenyl)(4-methoxybenzyl)sulfane (**1g**) (400 MHz, DMSO-*d*$_6$, 298 K).

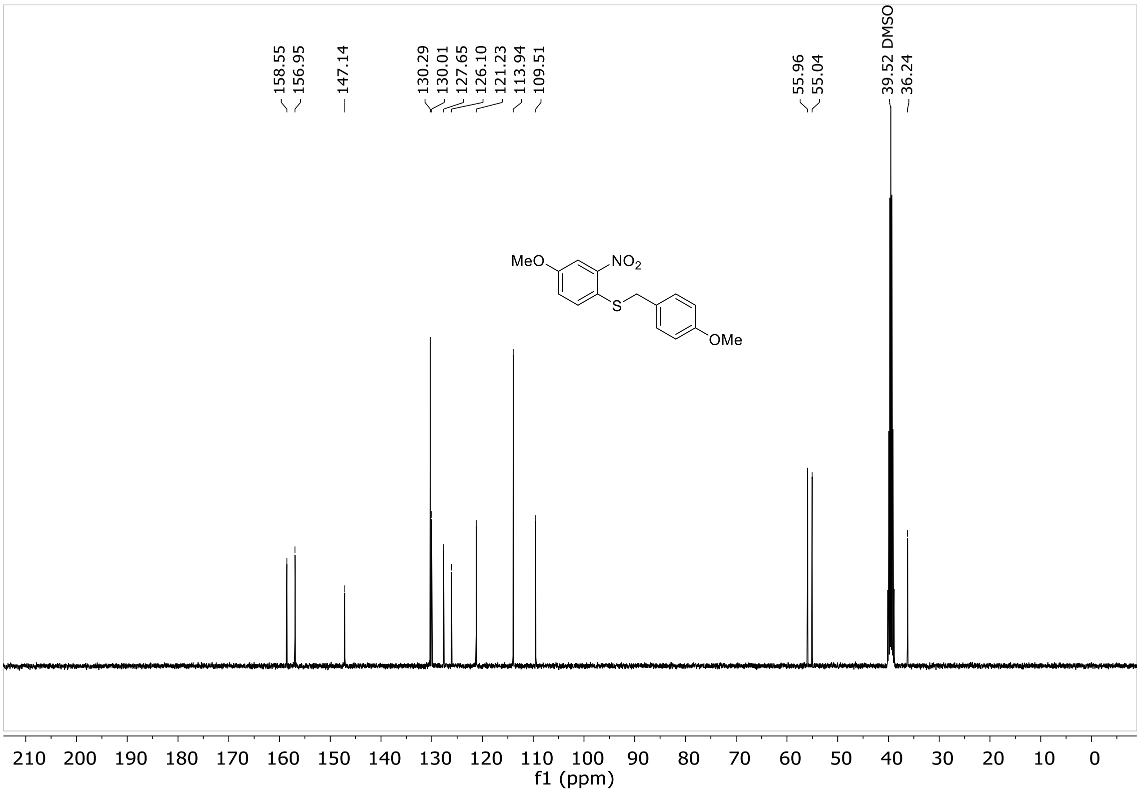


**Figure S018**: $^{13}C\{^{1}H\}$ NMR spectrum of (4-methoxy-2-nitrophenyl)(4-methoxybenzyl)sulfane (**1g**) (100 MHz, DMSO-*d*$_6$, 298 K).

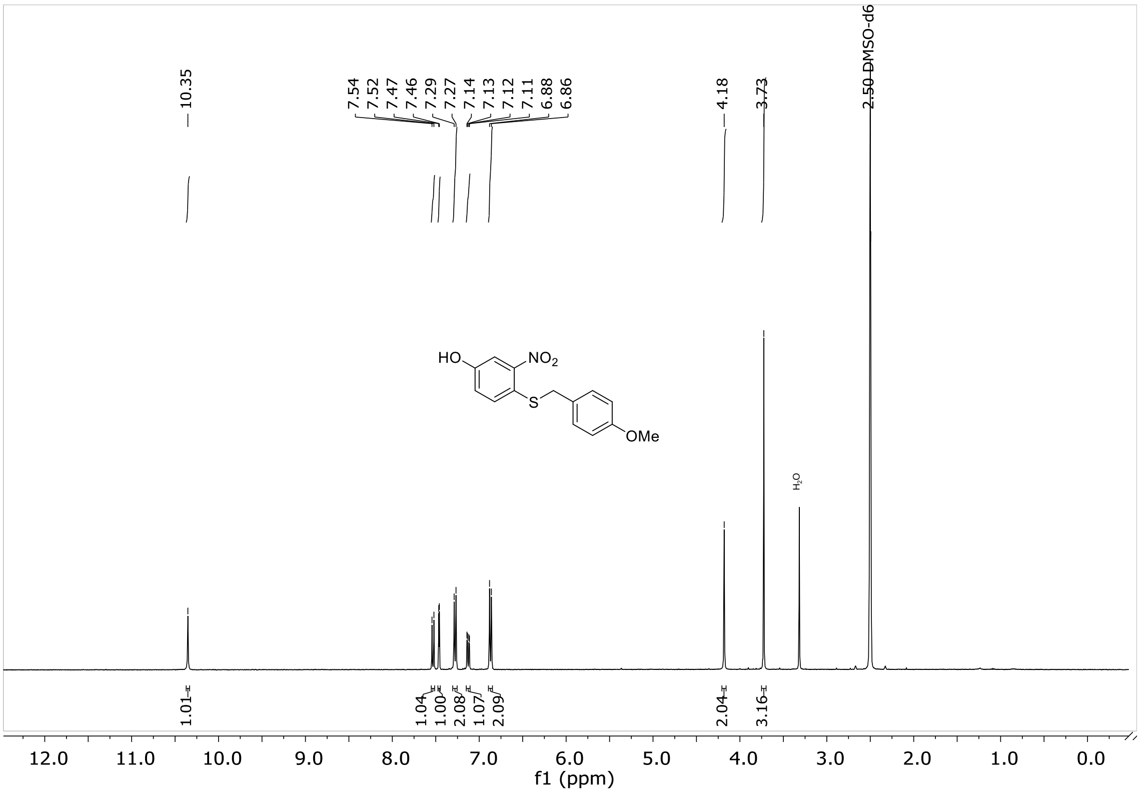


**Figure S019**: $^1$H NMR spectrum of 4-((4-methoxybenzyl)thio)-3-nitrophenol (**1h)** (400 MHz, DMSO-$d_6$, 298 K).

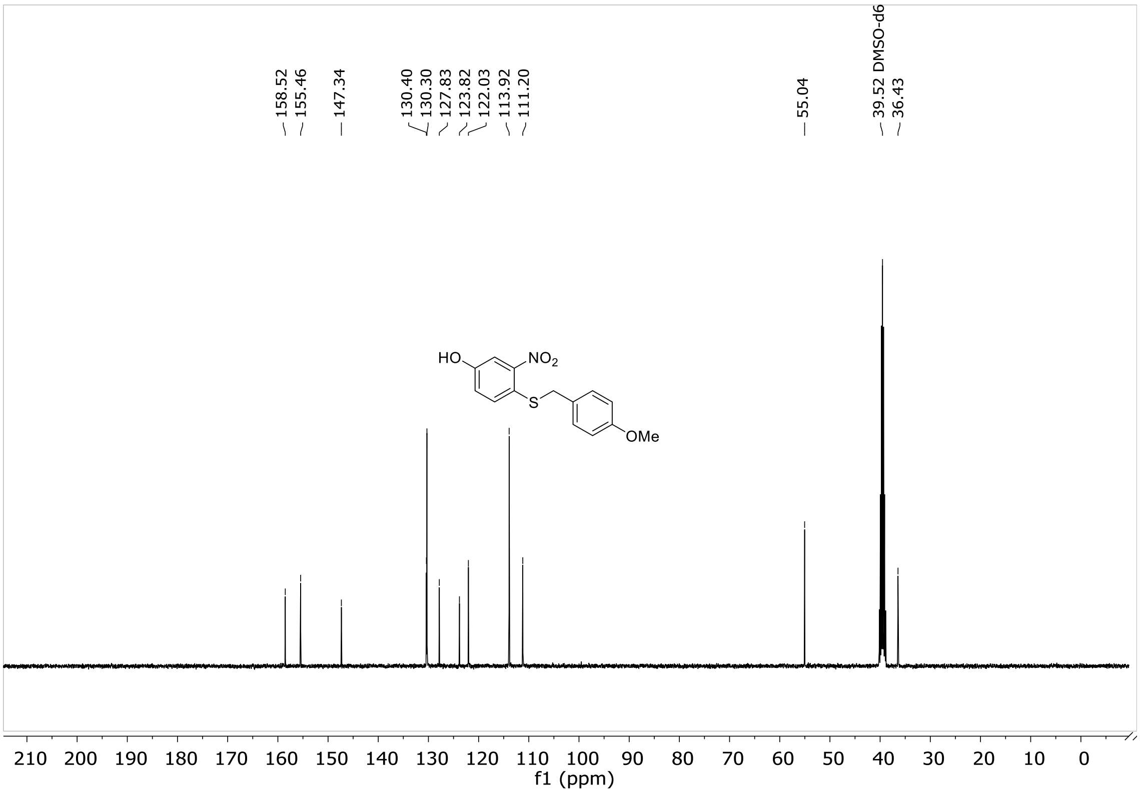


**Figure S020**: $^{13}C\{^1H\}$ NMR spectrum of 4-((4-methoxybenzyl)thio)-3-nitrophenol (**1h)** (100 MHz, DMSO-$d_6$, 298 K).

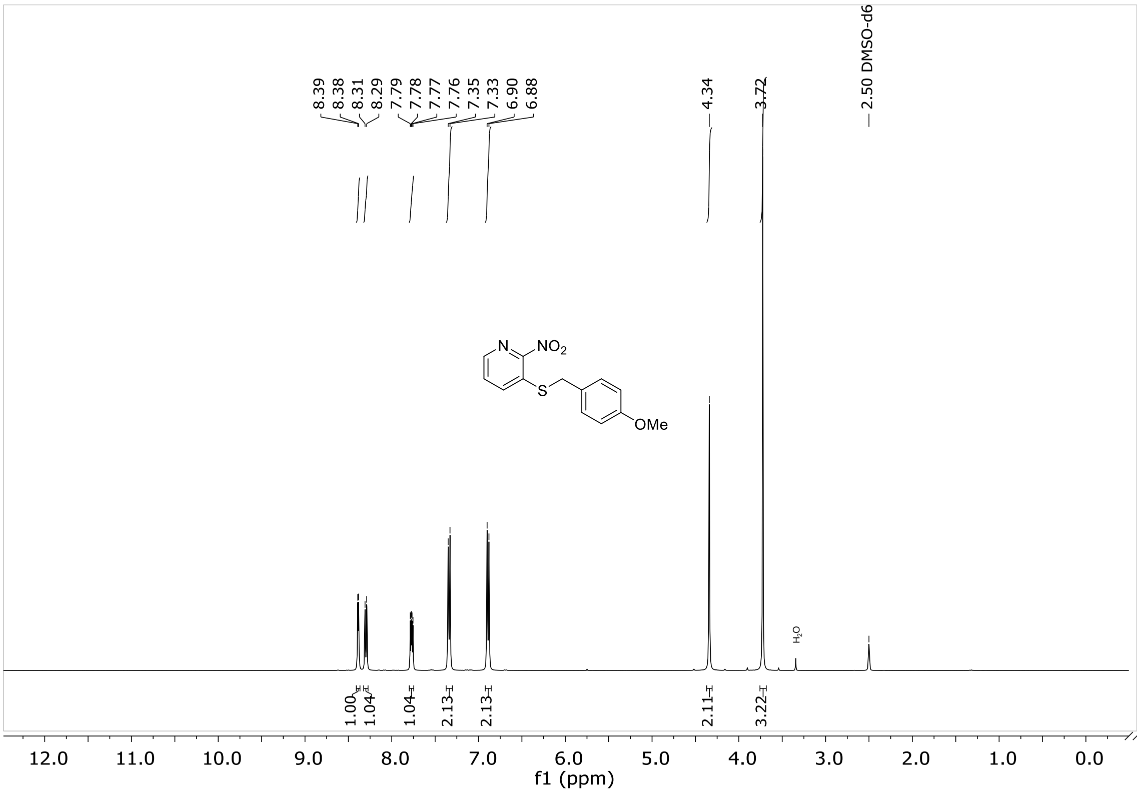


**Figure S021**: $^{1}H$ NMR spectrum of 3-((4-methoxybenzyl)thio)-2-nitropyridine (**1i**) (400 MHz, DMSO-$d_6$, 298 K).

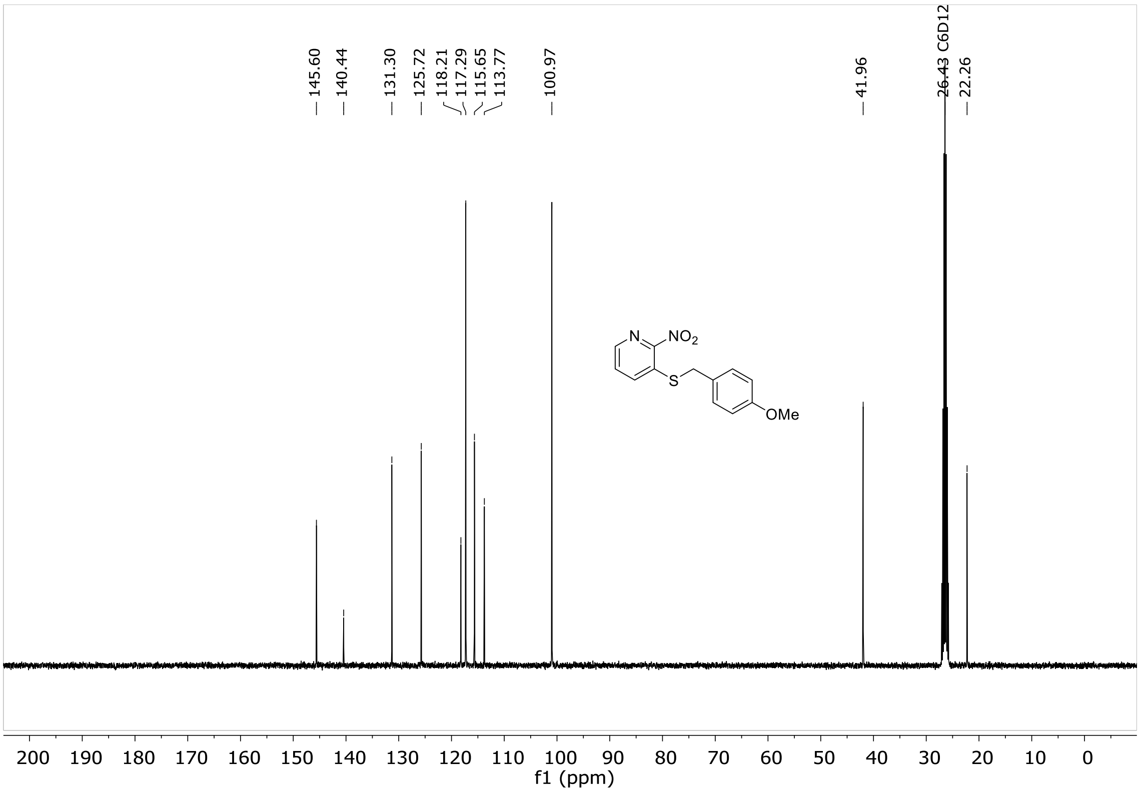


**Figure S022**: $^{13}C\{^{1}H\}$ NMR spectrum of 3-((4-methoxybenzyl)thio)-2-nitropyridine (**1i**) (100 MHz, DMSO-$d_6$, 298 K).

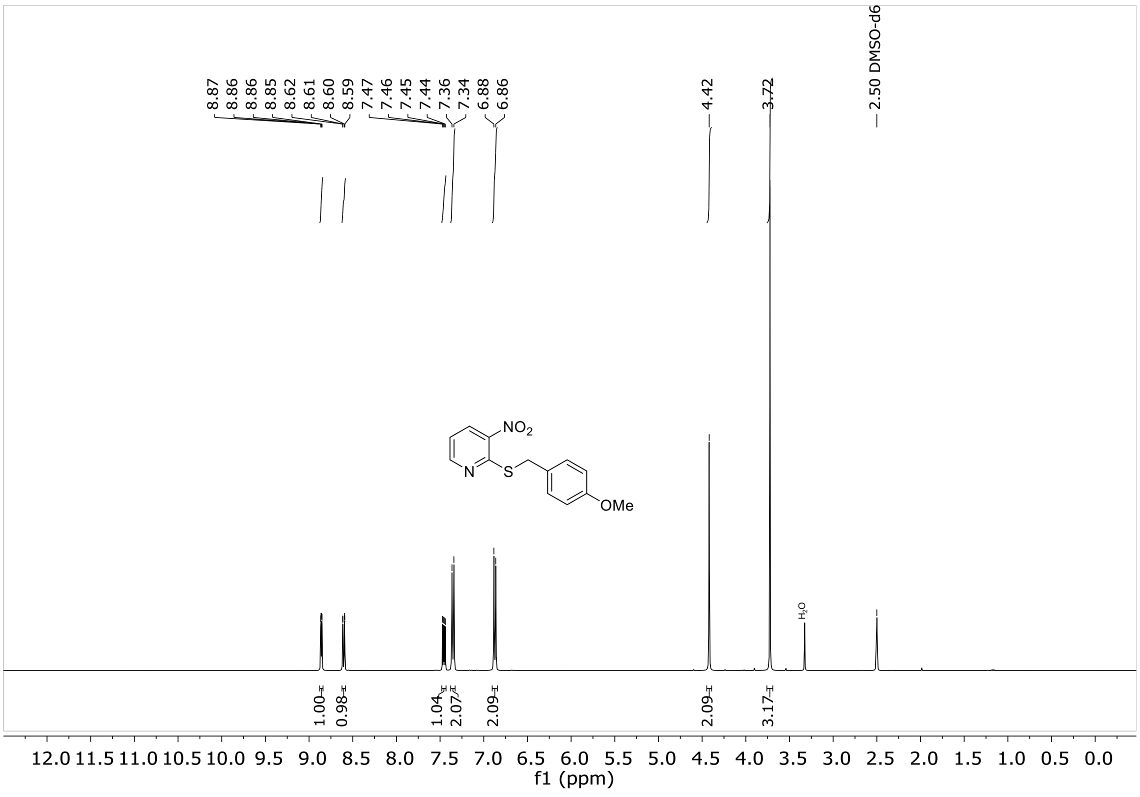

**Figure S023**: $^{1}$H NMR spectrum of 2-((4-methoxybenzyl)thio)-3-nitropyridine (**1j**) (400 MHz, DMSO-$d_6$, 298 K).

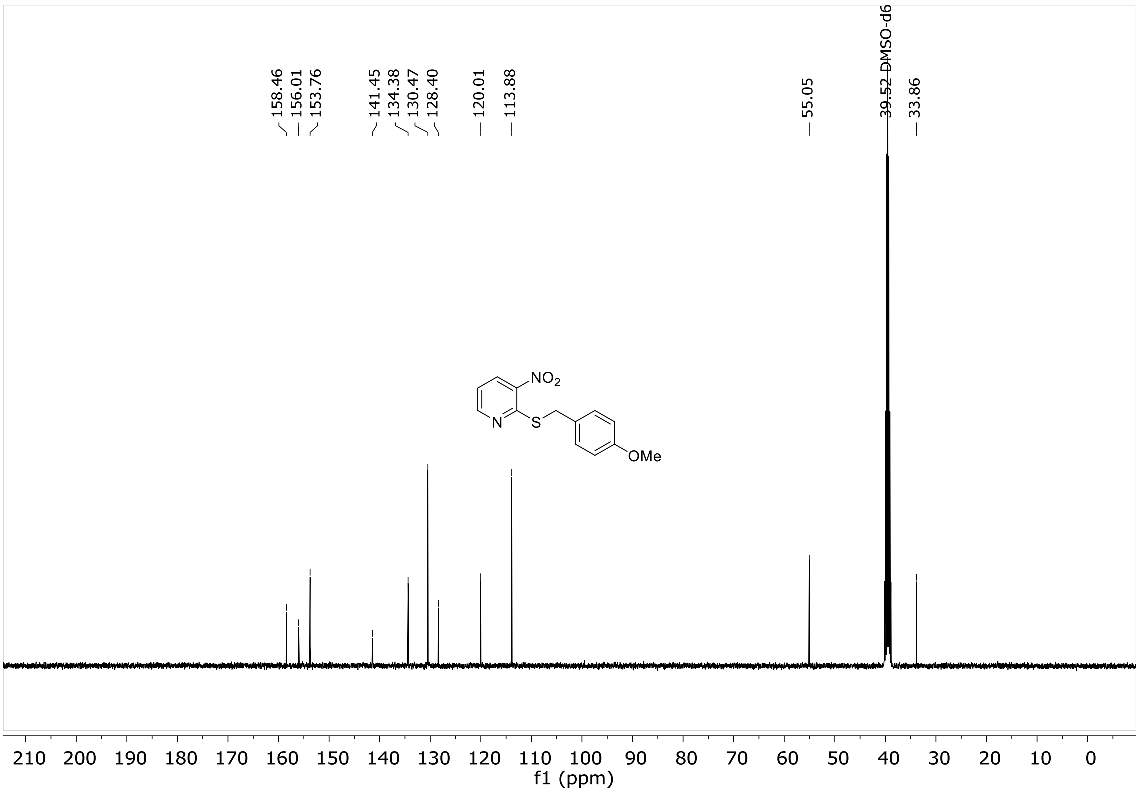

**Figure S024**: $^{13}$C{$^{1}$H} NMR spectrum of 2-((4-methoxybenzyl)thio)-3-nitropyridine (**1j**) (100 MHz, DMSO-$d_6$, 298 K).

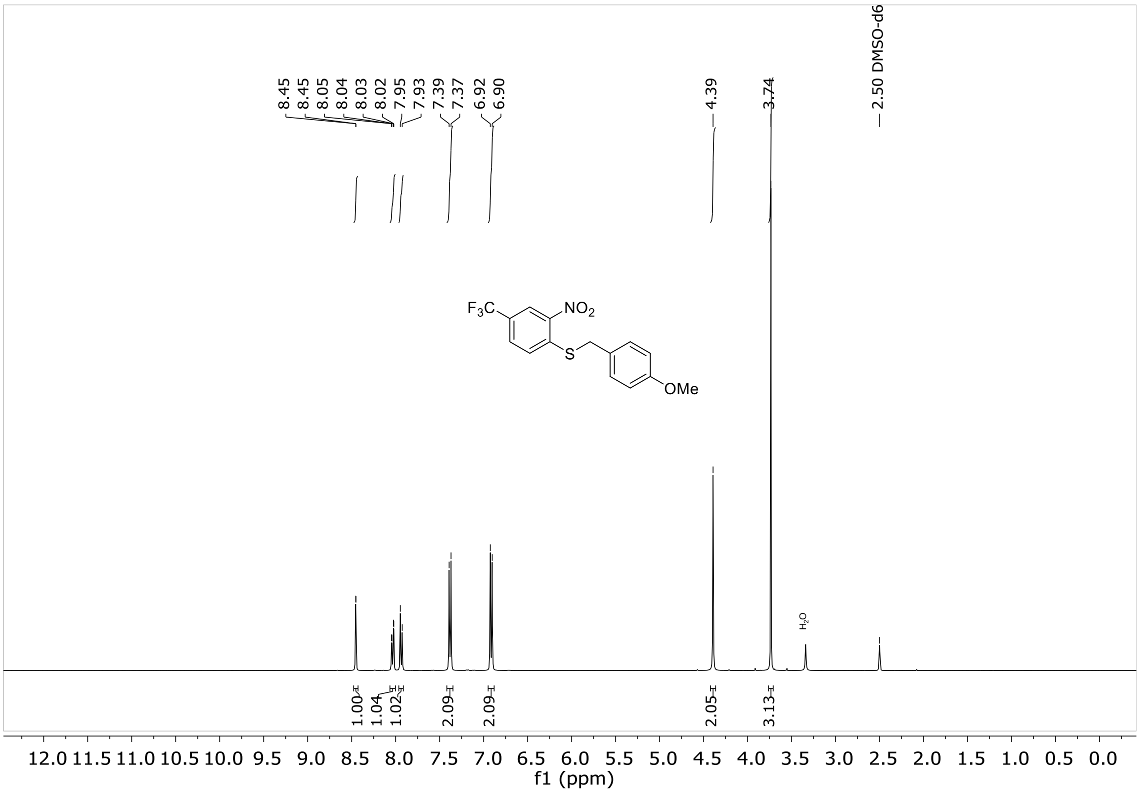


**Figure S025**: $^{1}$H NMR spectrum of (4-methoxybenzyl)(2-nitro-4-(trifluoromethyl)phenyl)sulfane (**1k**) (400 MHz, DMSO-$d_6$, 298 K).

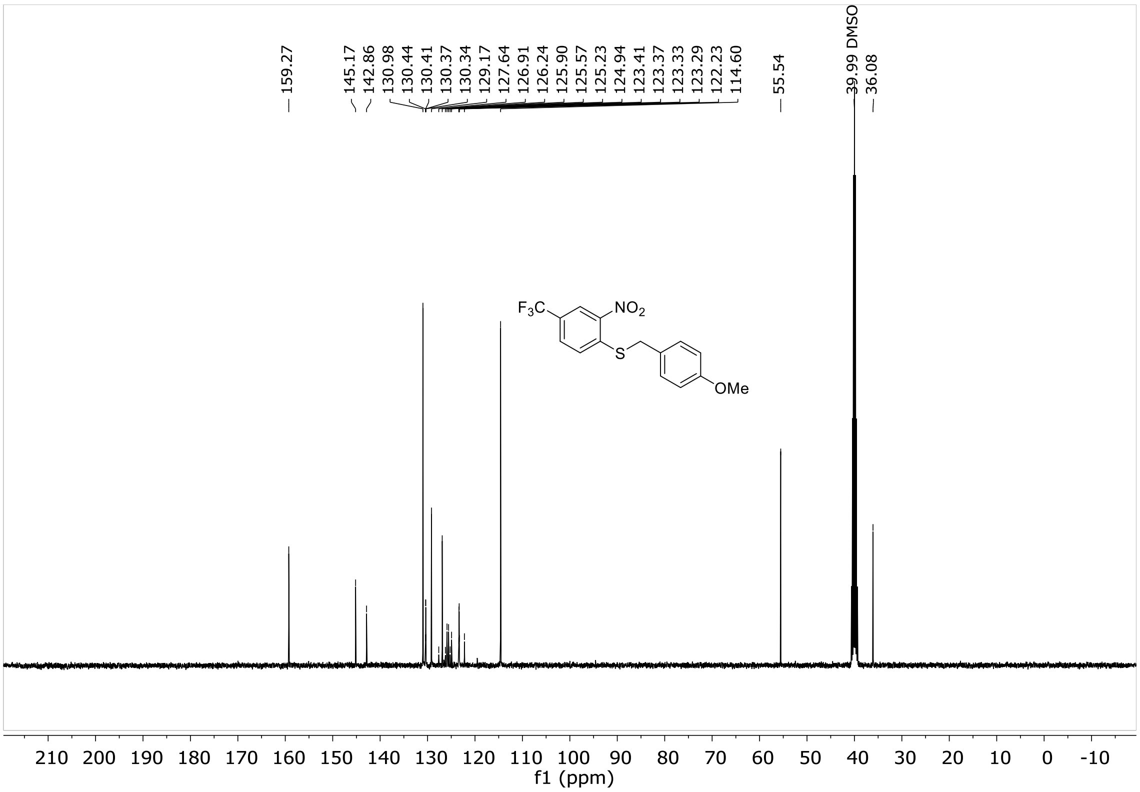


**Figure S026**: $^{13}$C{$^{1}$H} NMR spectrum of (4-methoxybenzyl)(2-nitro-4-(trifluoromethyl)phenyl)sulfane (**1k**) (100 MHz, DMSO-$d_6$, 298 K).

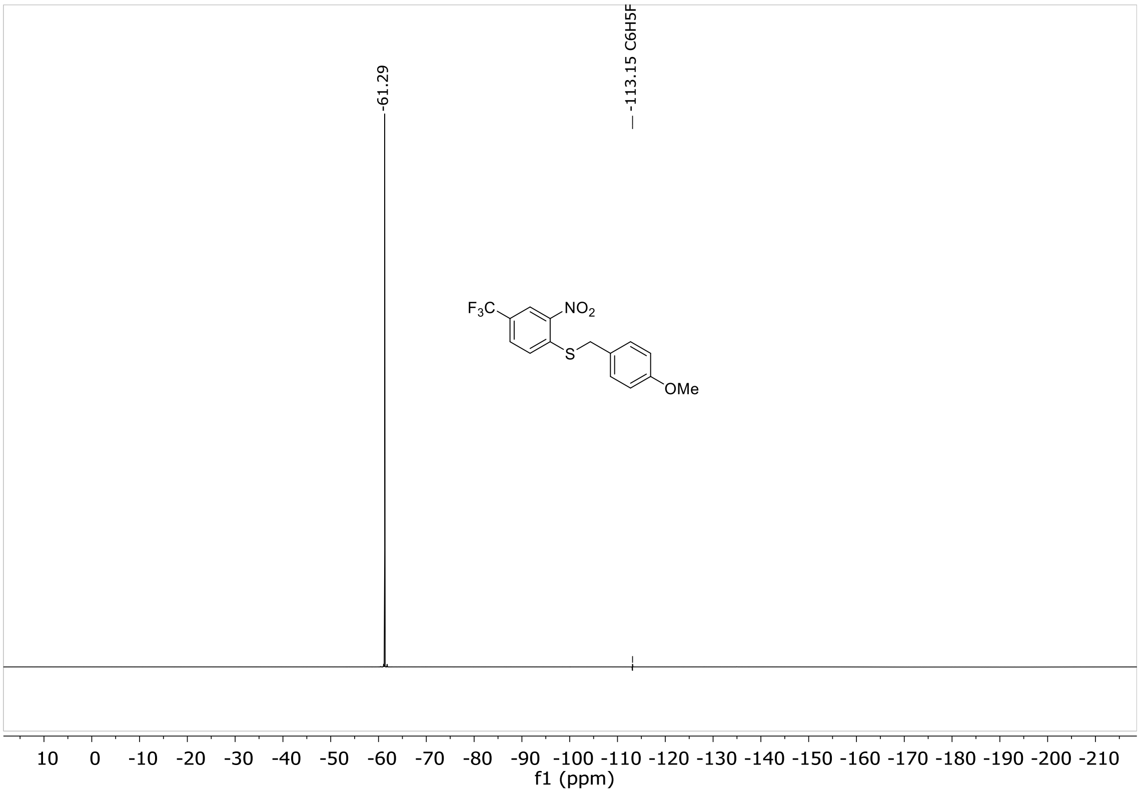


**Figure S027**: $^{13}C\{^{1}H\}$ NMR spectrum of (4-methoxybenzyl)(2-nitro-4-(trifluoromethyl)phenyl)sulfane (**1k**) (376 MHz, DMSO-$d_6$, 298 K, referenced to fluorobenzene).

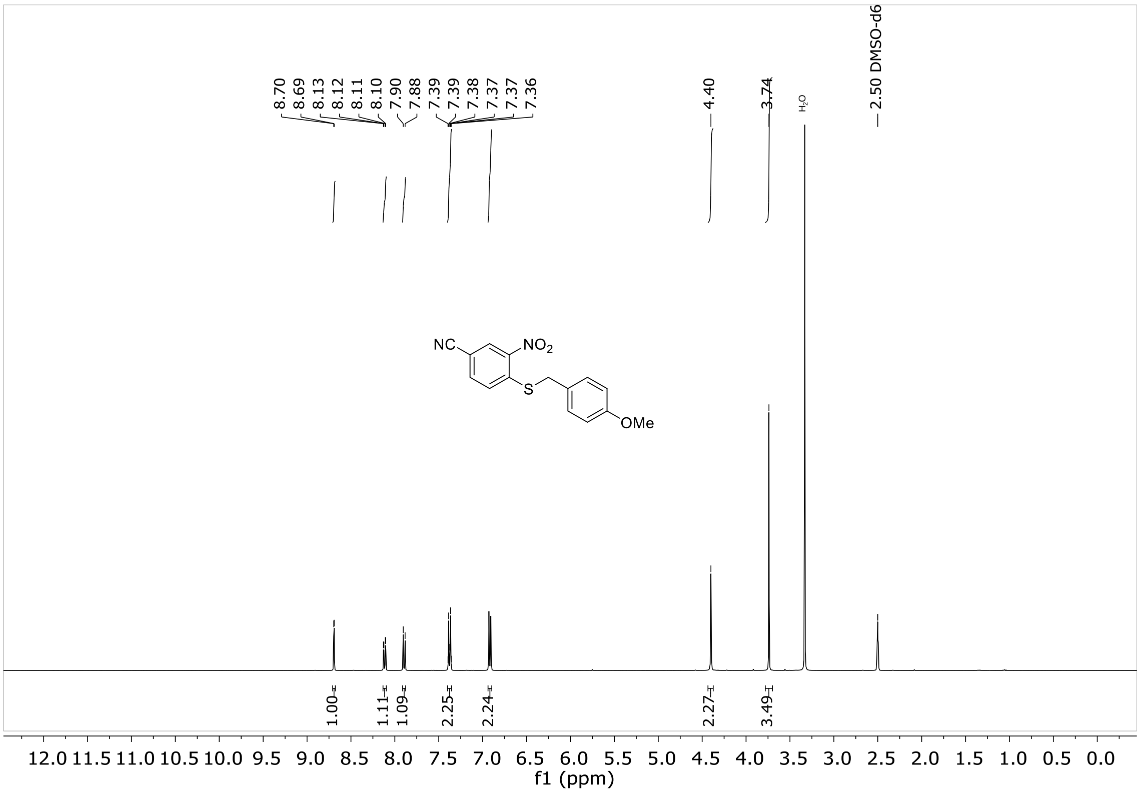


**Figure S028**: $^{1}H$ NMR spectrum of 4-((4-methoxybenzyl)thio)-3-nitrobenzonitrile (**1l**) (400 MHz, DMSO-$d_6$, 298 K).

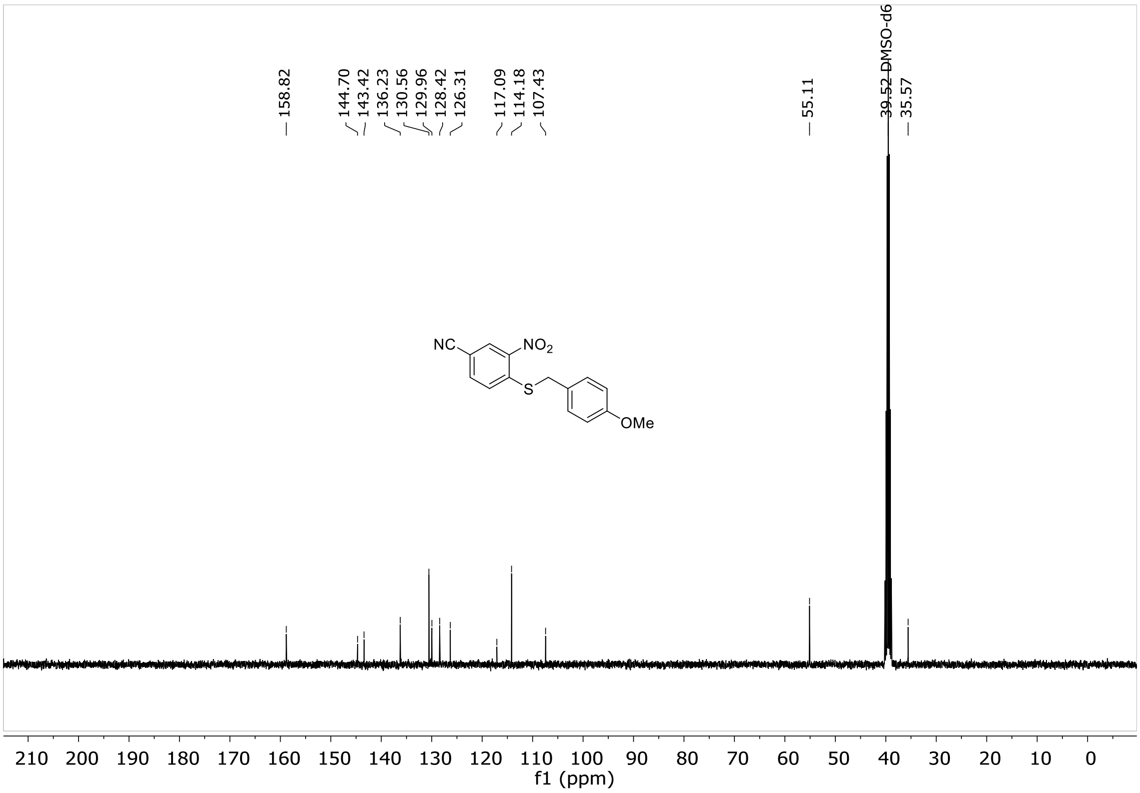


**Figure S029**: $^{13}C\{^1H\}$ NMR spectrum of 4-((4-methoxybenzyl)thio)-3-nitrobenzonitrile (**1l**) (100 MHz, DMSO-$d_6$, 298 K).

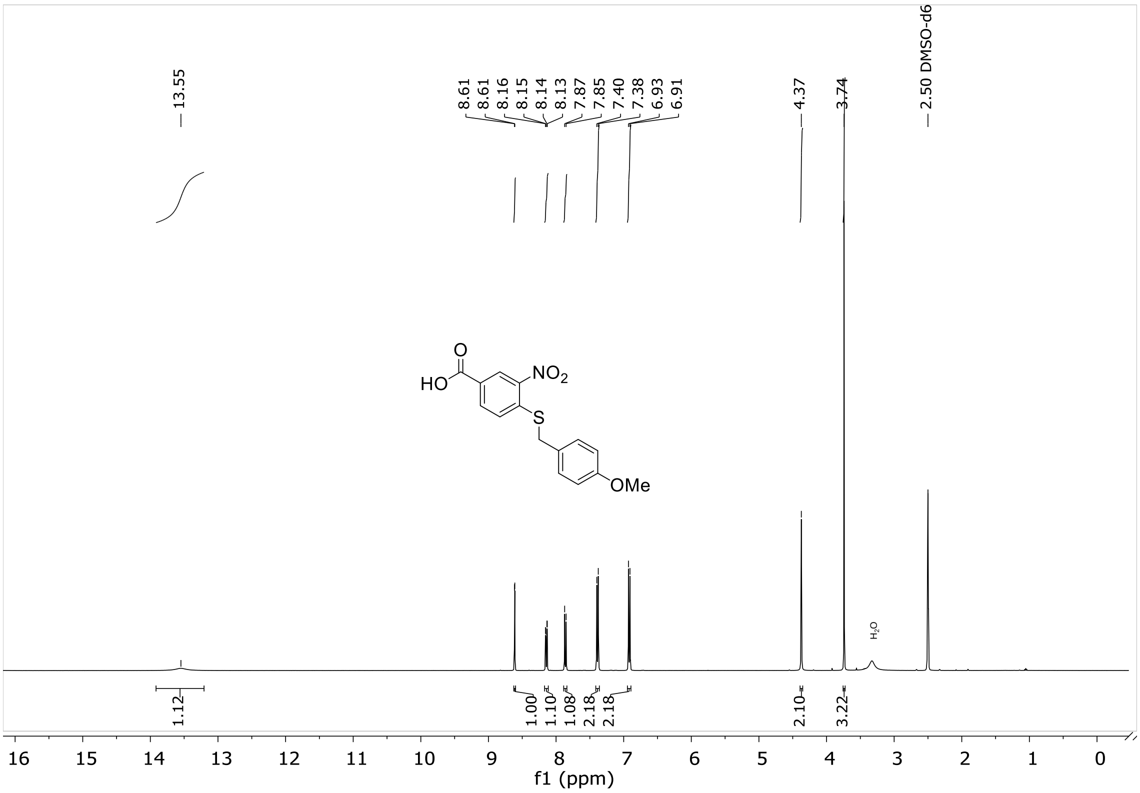


**Figure S030**: $^1H$ NMR spectrum of 4-((4-methoxybenzyl)thio)-3-nitrobenzoic acid (**1m**) (400 MHz, DMSO-$d_6$, 298 K).

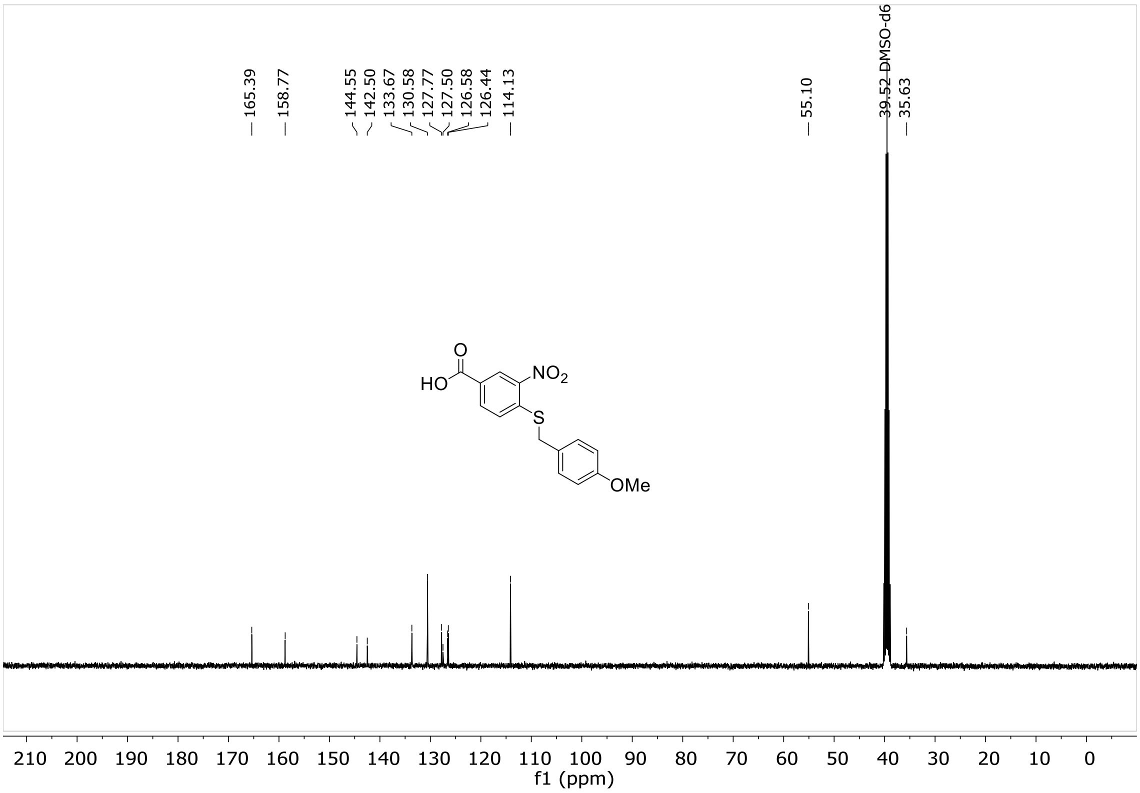


**Figure S031**: $^{13}C\{^{1}H\}$ NMR spectrum of 4-((4-methoxybenzyl)thio)-3-nitrobenzoic acid (**1m**) (400 MHz, DMSO-$d_6$, 298 K).

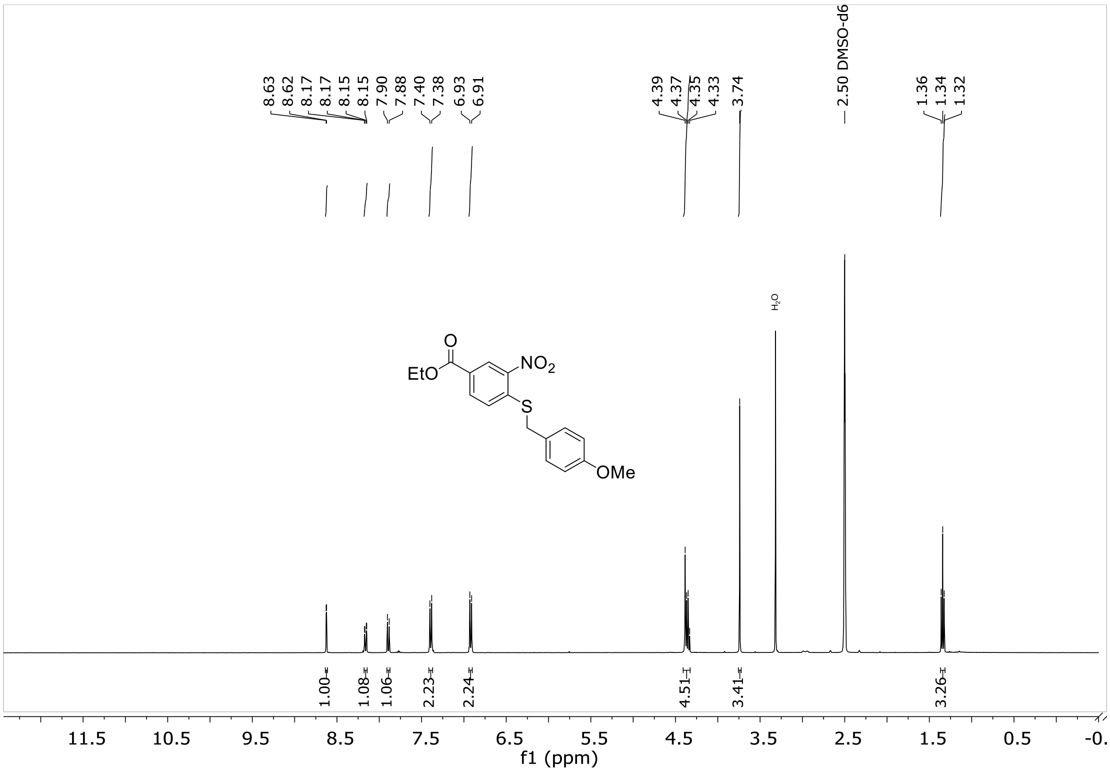


**Figure S032**: $^{1}H$ NMR spectrum of ethyl 4-((4-methoxybenzyl)thio)-3-nitrobenzoate (**1m’**) (400 MHz, DMSO-$d_6$, 298 K).

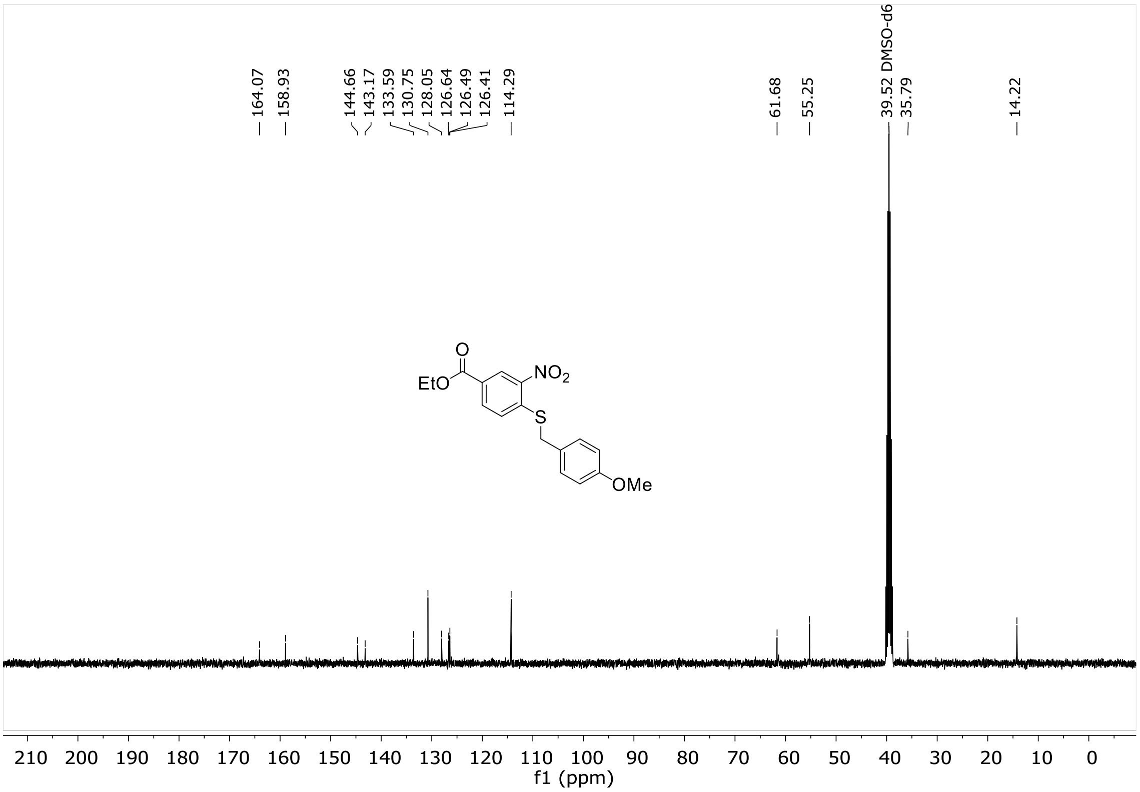

**Figure S033**: $^{13}C\{^{1}H\}$ NMR spectrum of ethyl 4-((4-methoxybenzyl)thio)-3-nitrobenzoate (**1m**') (100 MHz, DMSO-$d_6$, 298 K).

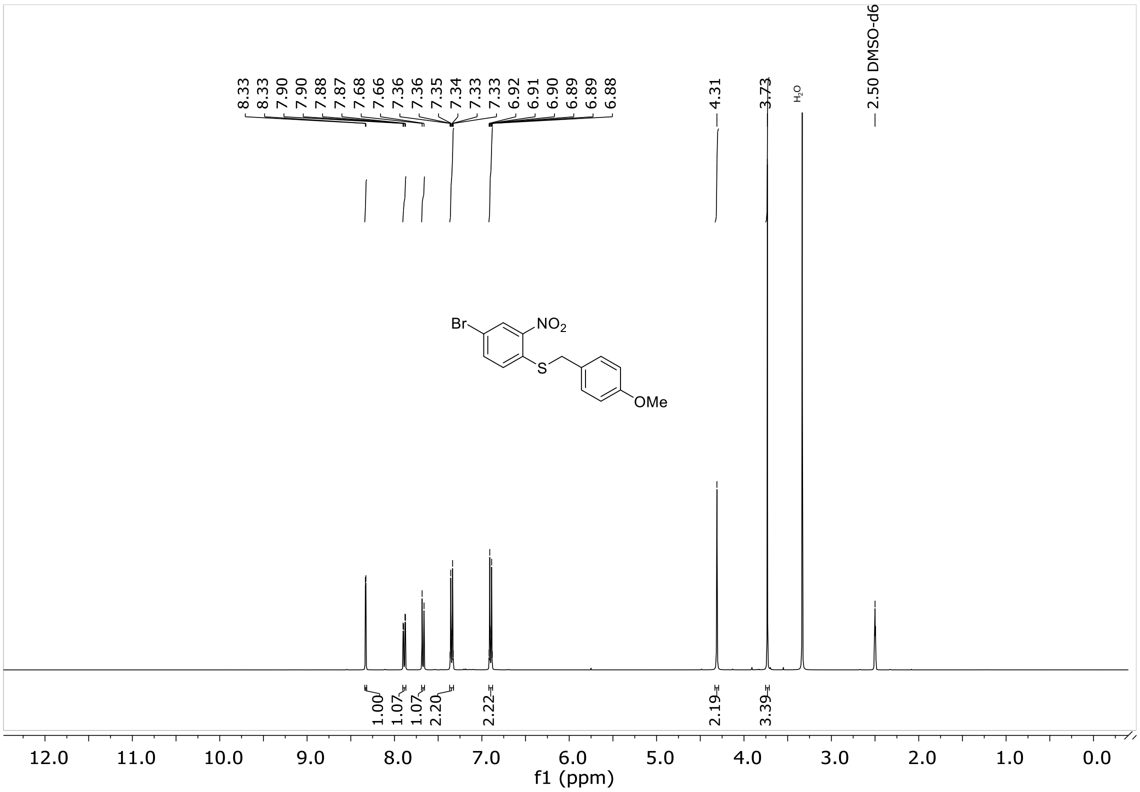

**Figure S034**: $^{1}H$ NMR spectrum of (4-bromo-2-nitrophenyl)(4-methoxybenzyl)sulfane (**1n**) (400 MHz, DMSO-$d_6$, 298 K).

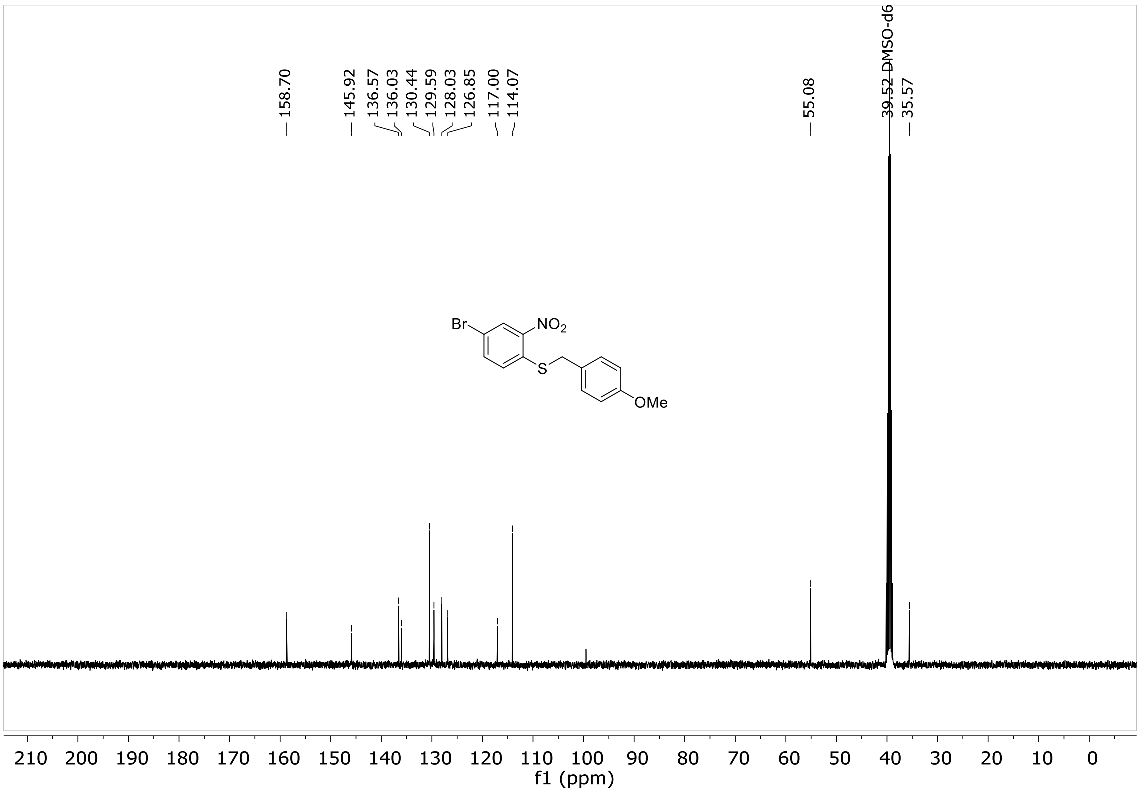


**Figure S035**: $^{13}C\{^{1}H\}$ NMR spectrum of (4-bromo-2-nitrophenyl)(4-methoxybenzyl)sulfane (**1n**) (100 MHz, DMSO-$d_6$, 298 K).

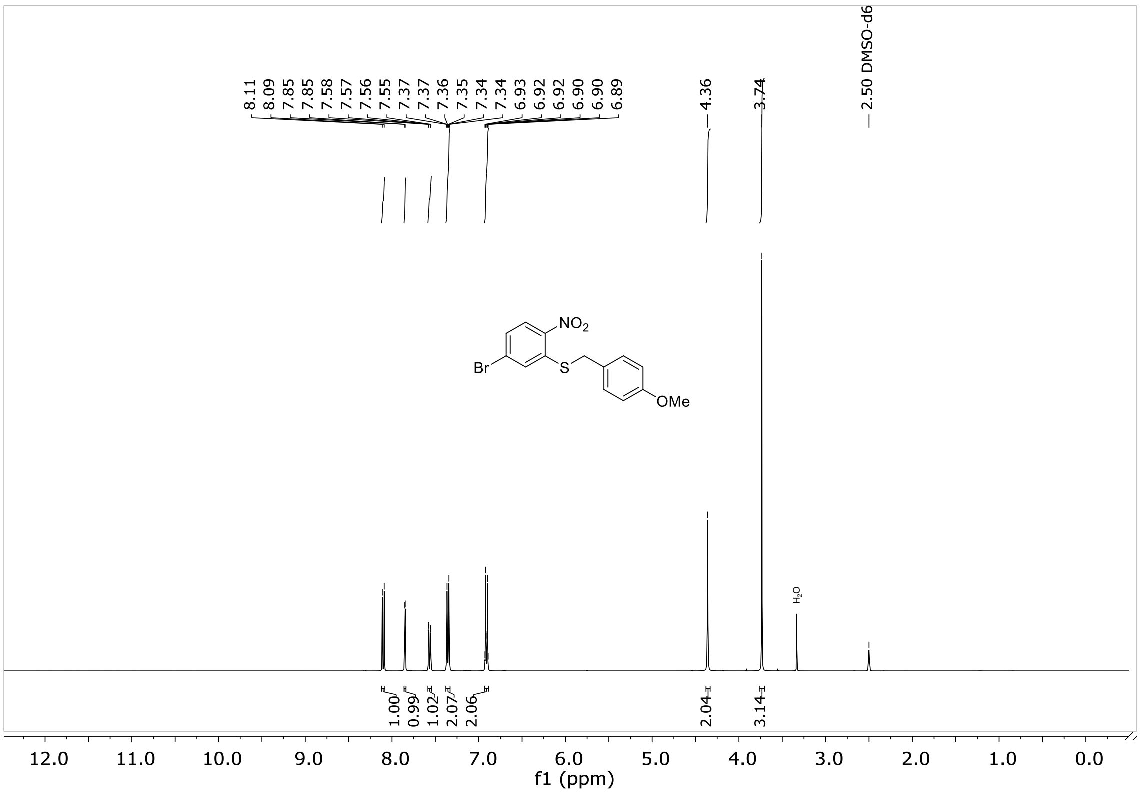


**Figure S036**: $^{1}H$ NMR spectrum of (5-bromo-2-nitrophenyl)(4-methoxybenzyl)sulfane (**1o**) (400 MHz, DMSO-$d_6$, 298 K).

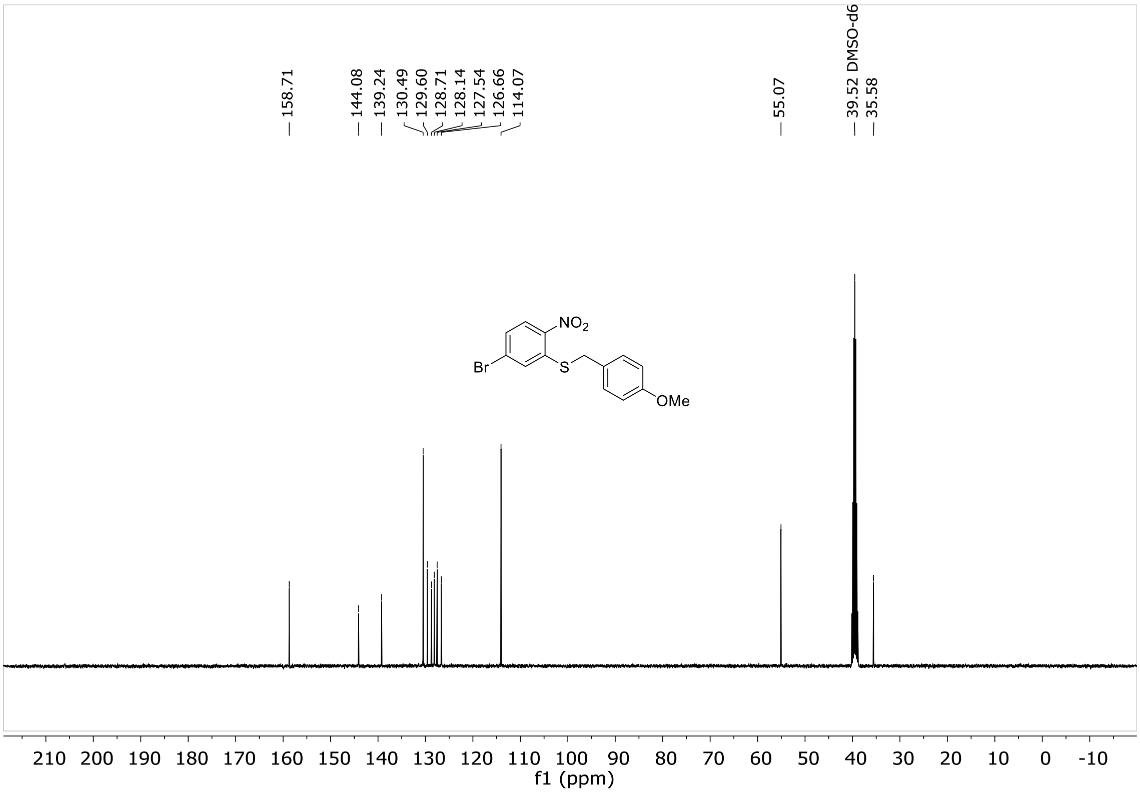


**Figure S037**: $^{13}C\{^{1}H\}$ NMR spectrum of (5-bromo-2-nitrophenyl)(4-methoxybenzyl)sulfane (**1o**) (100 MHz, DMSO-$d_6$, 298 K).

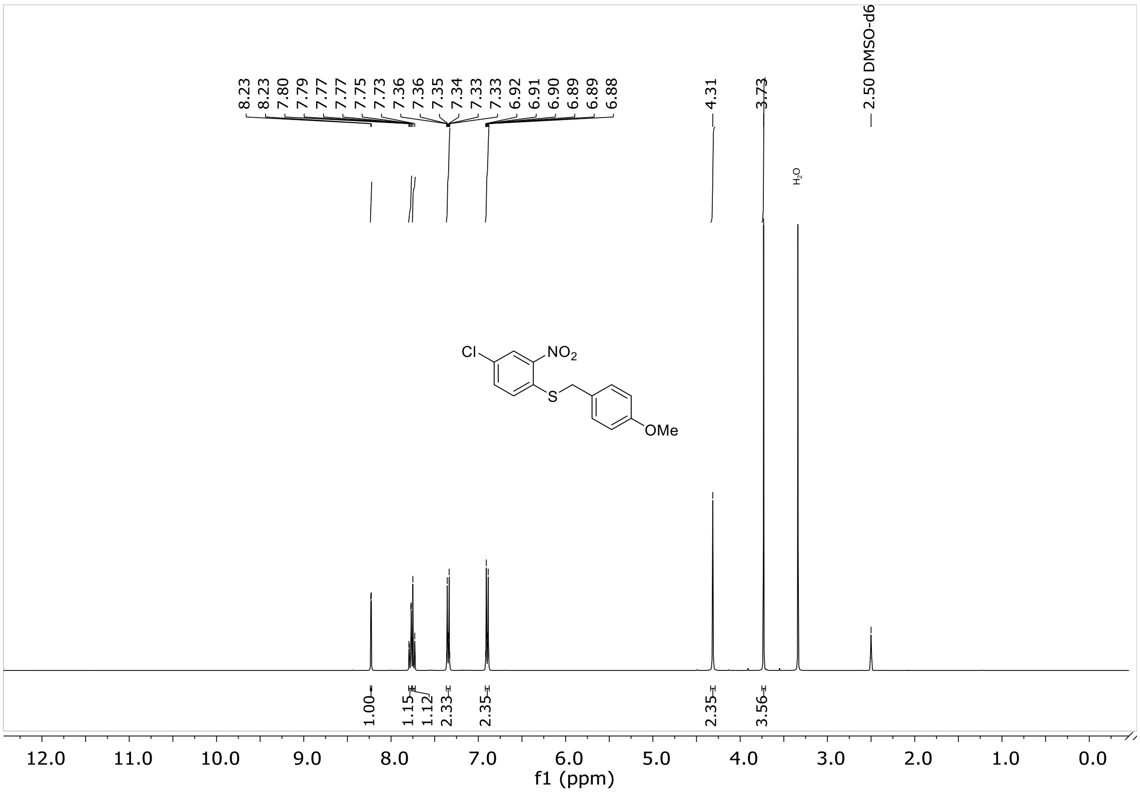


**Figure S038**: $^{1}H$ NMR spectrum of (4-chloro-2-nitrophenyl)(4-methoxybenzyl)sulfane (**1p**) (400 MHz, DMSO-$d_6$, 298 K).

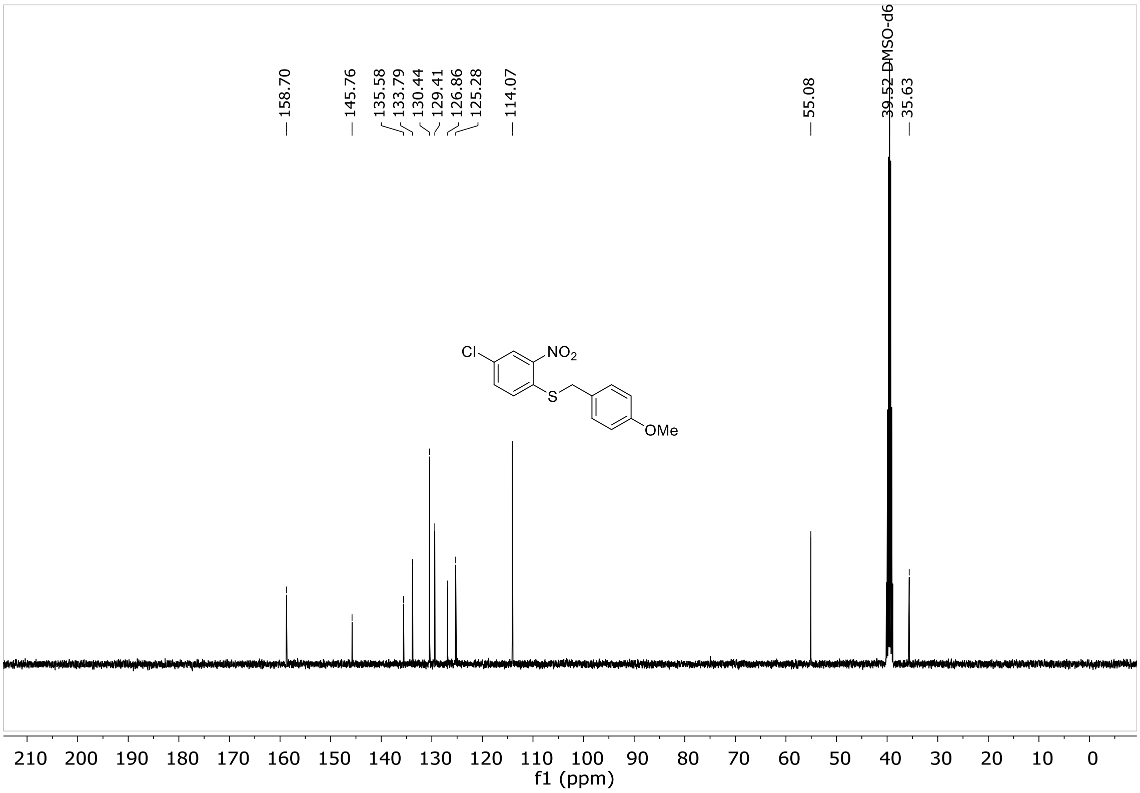


**Figure S039**: $^{13}C\{^{1}H\}$ NMR spectrum of (4-chloro-2-nitrophenyl)(4-methoxybenzyl)sulfane (**1p**) (100 MHz, DMSO-$d_6$, 298 K).

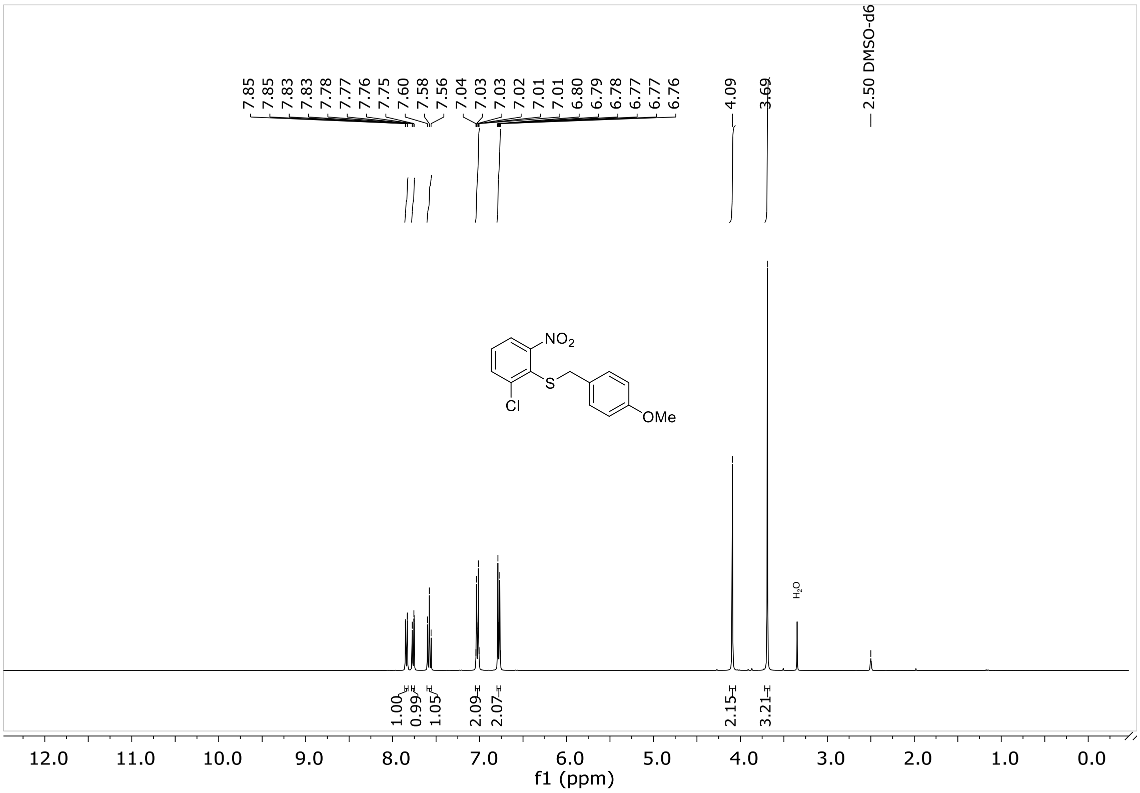


**Figure S040**: $^{1}H$ NMR spectrum of (2-chloro-6-nitrophenyl)(4-methoxybenzyl)sulfane (**1q**) (400 MHz, DMSO-$d_6$, 298 K).

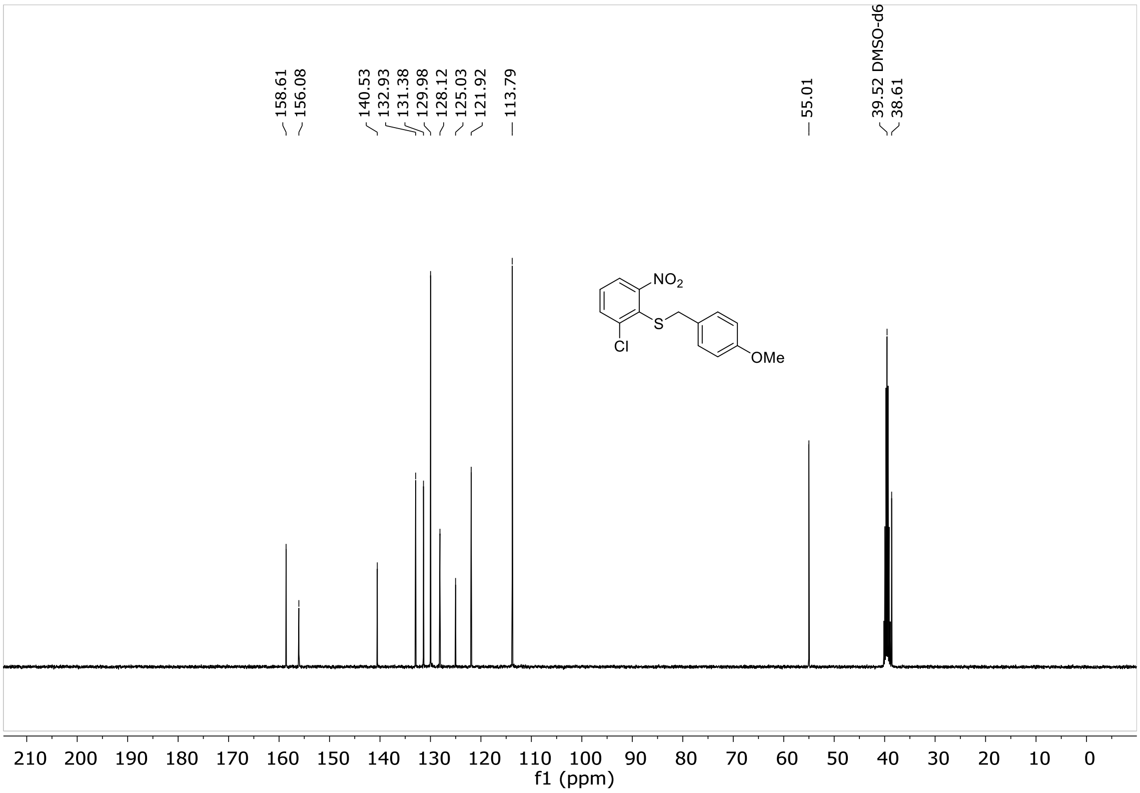


**Figure S041**: $^{13}C\{^{1}H\}$ NMR spectrum of (2-chloro-6-nitrophenyl)(4-methoxybenzyl)sulfane (**1q**) (100 MHz, DMSO-$d_6$, 298 K).

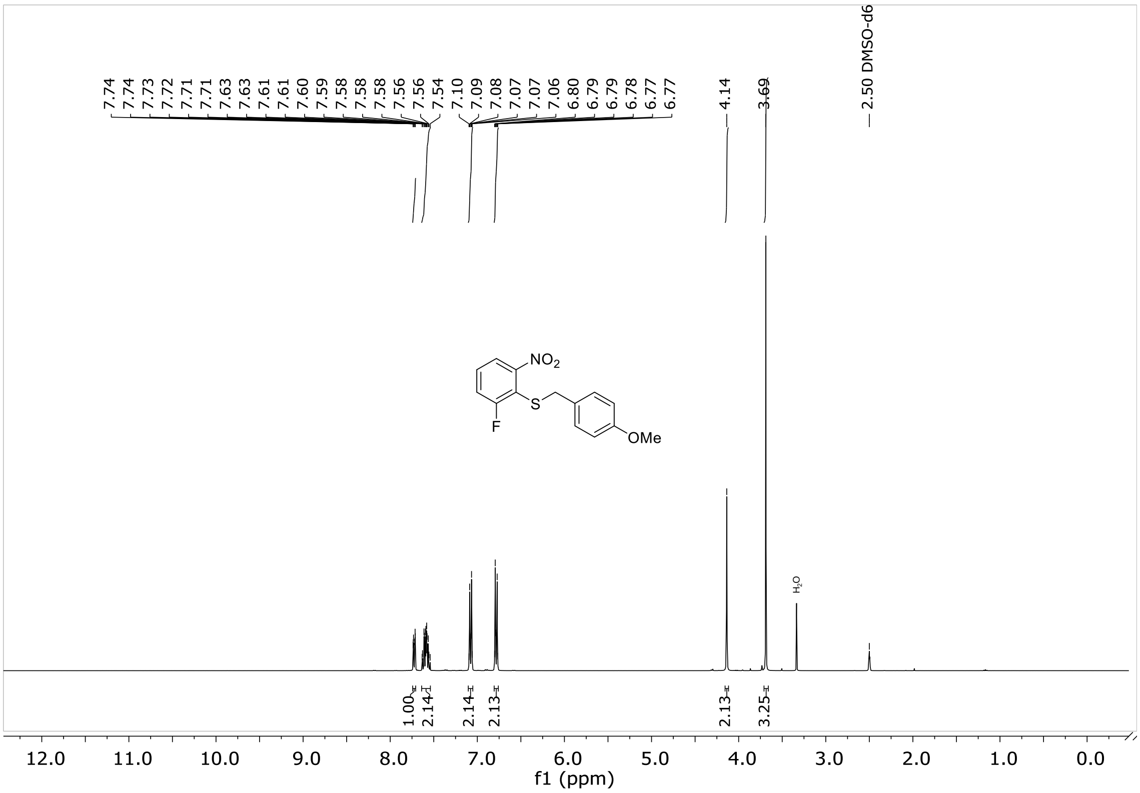


**Figure S042**: $^{1}H$ NMR spectrum of (2-fluoro-6-nitrophenyl)(4-methoxybenzyl)sulfane (**1r**) (400 MHz, DMSO-$d_6$, 298 K).

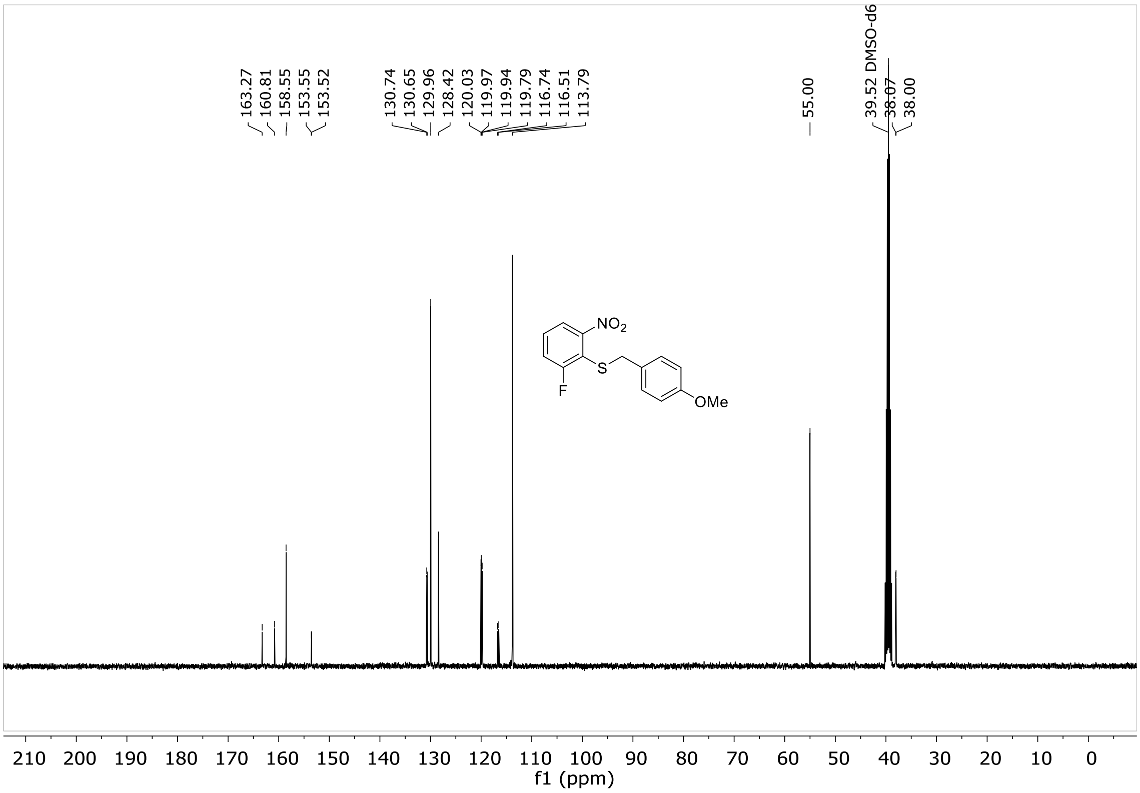


**Figure S043**: $^{13}C\{^{1}H\}$ NMR spectrum of (2-fluoro-6-nitrophenyl)(4-methoxybenzyl)sulfane (**1r**) (100 MHz, DMSO-$d_6$, 298 K).

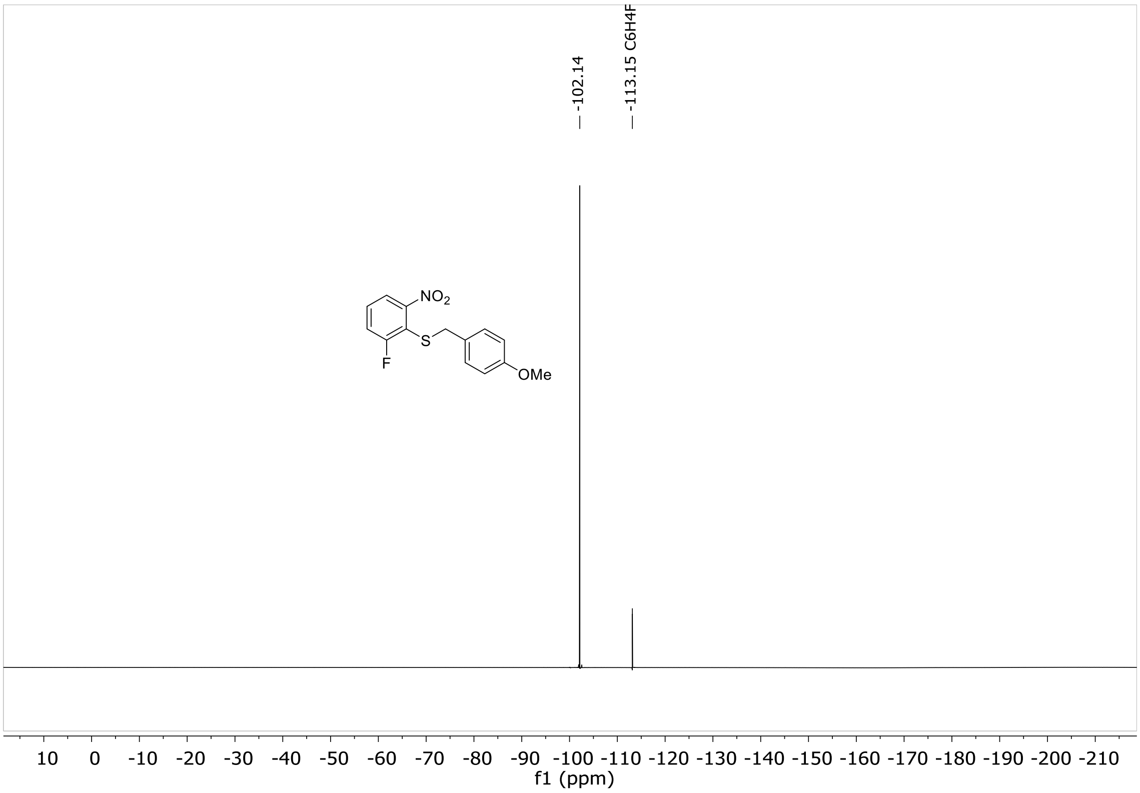


**Figure S044**: $^{19}F\{^{1}H\}$ NMR spectrum of (2-fluoro-6-nitrophenyl)(4-methoxybenzyl)sulfane (**1r**) (376 MHz, DMSO-$d_6$, 298 K, referenced to fluorobenzene).

## *1.3 NMR Spectra of Substituted 2-((4-Methoxybenzyl)thio)anilines (2b-2s)*

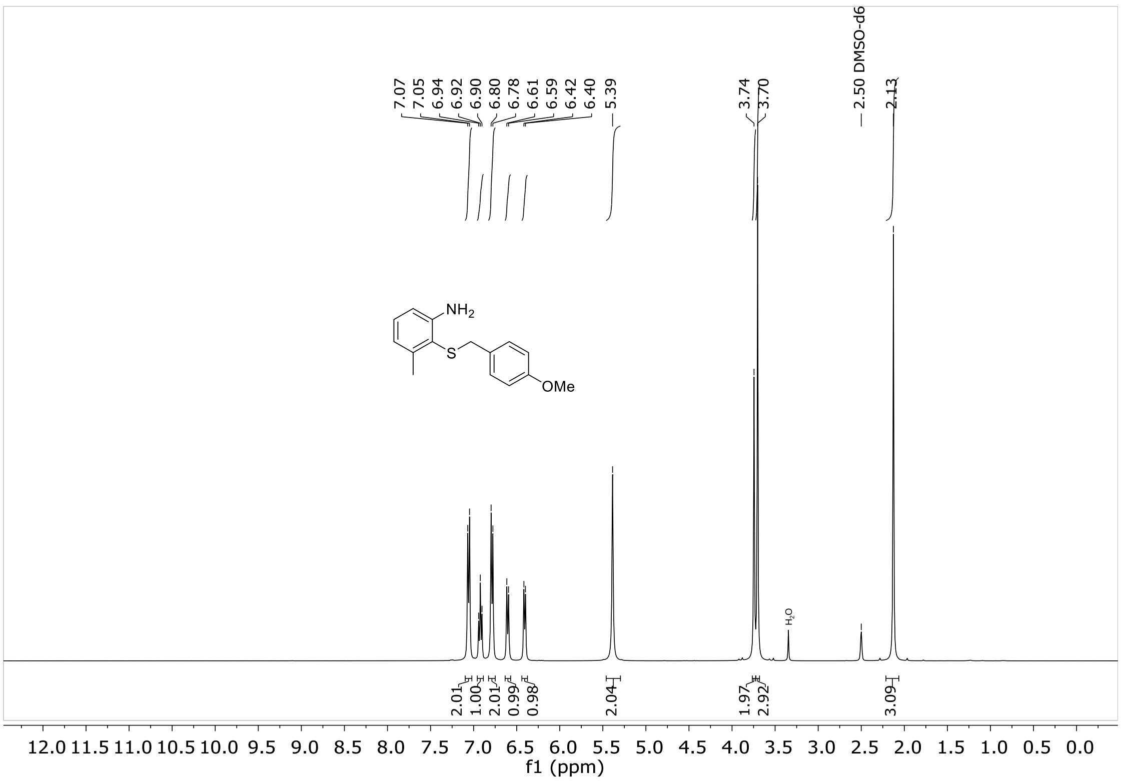


**Figure S045**: $^{1}H$ NMR spectrum of 2-((4-methoxybenzyl)thio)-3-methylaniline (**2b**) (400 MHz, DMSO-$d_6$, 298 K).

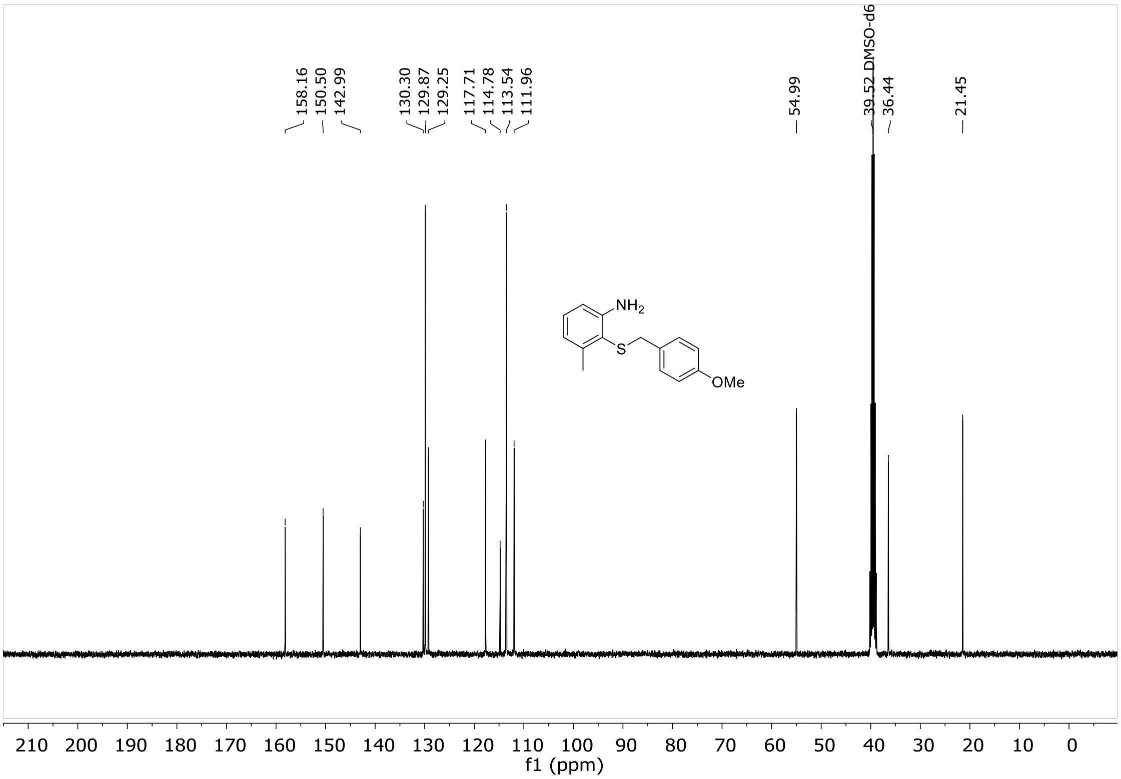


**Figure S046**: $^{13}C\{^{1}H\}$ NMR spectrum of 2-((4-methoxybenzyl)thio)-3-methylaniline (**2b**) (100 MHz, DMSO-$d_6$, 298 K).

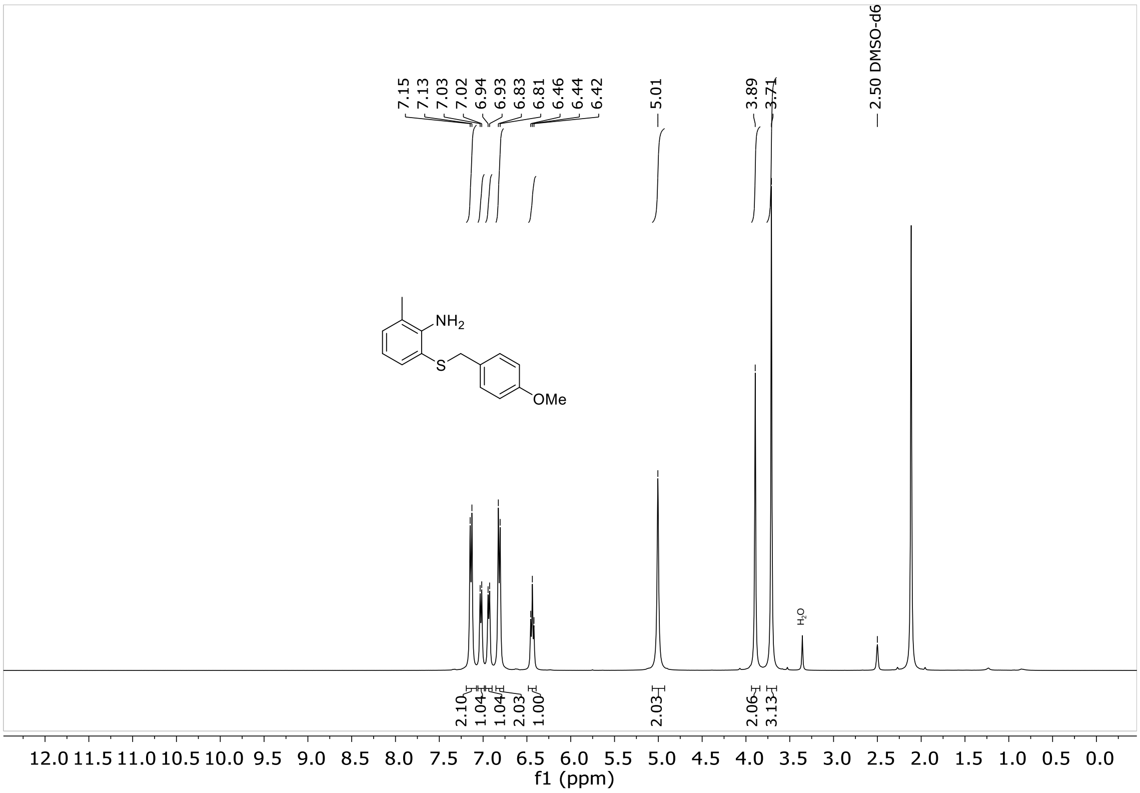


**Figure S047**: $^{1}H$ NMR spectrum of 2-((4-methoxybenzyl)thio)-6-methylaniline (**2c**) (400 MHz, DMSO-$d_6$, 298 K).

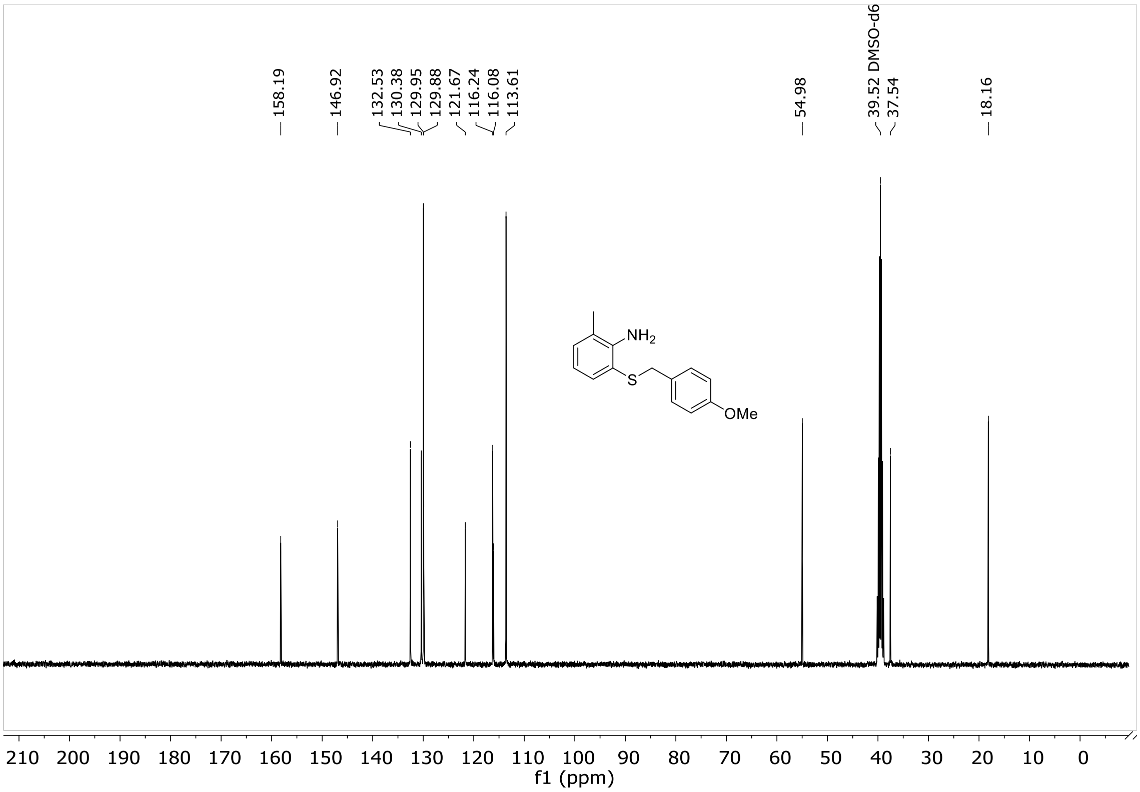


**Figure S048**: $^{13}C\{^{1}H\}$ NMR spectrum of 2-((4-methoxybenzyl)thio)-6-methylaniline (**2c**) (100 MHz, DMSO-$d_6$, 298 K).

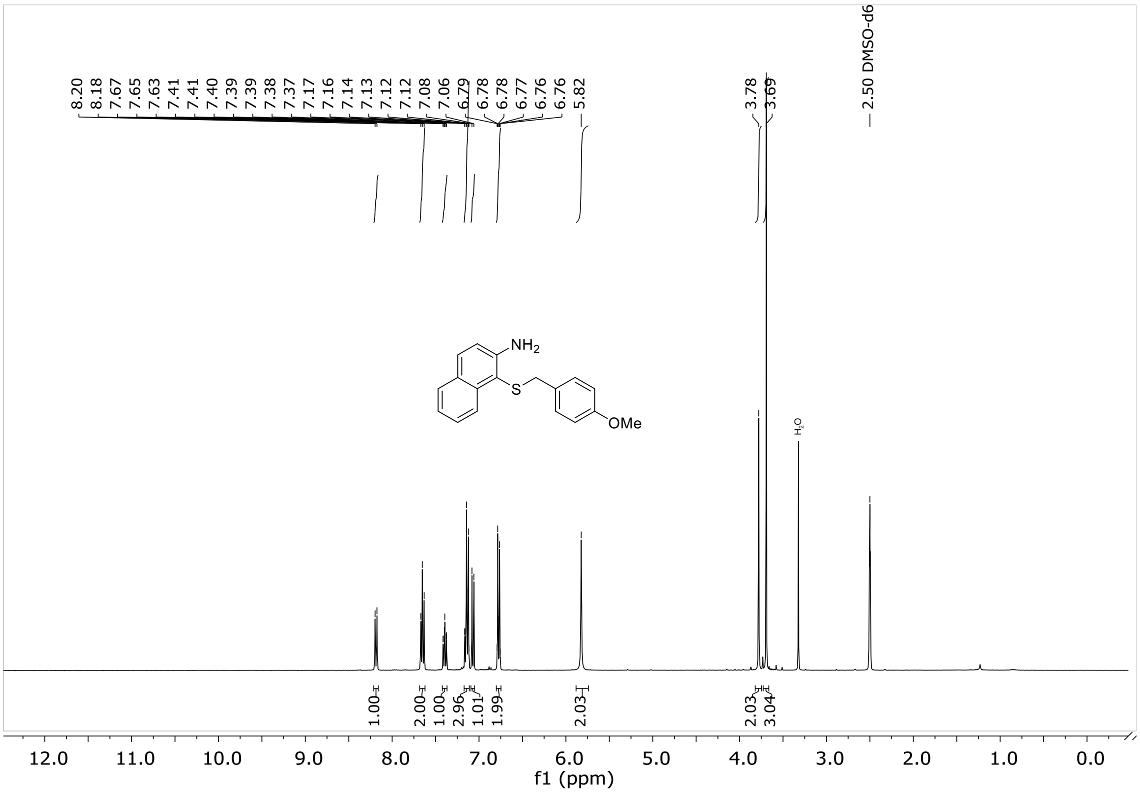


**Figure S049**: $^1$H NMR spectrum of 1-((4-methoxybenzyl)thio)naphthalen-2-amine (**2d**) (400 MHz, DMSO-$d_6$, 298 K).

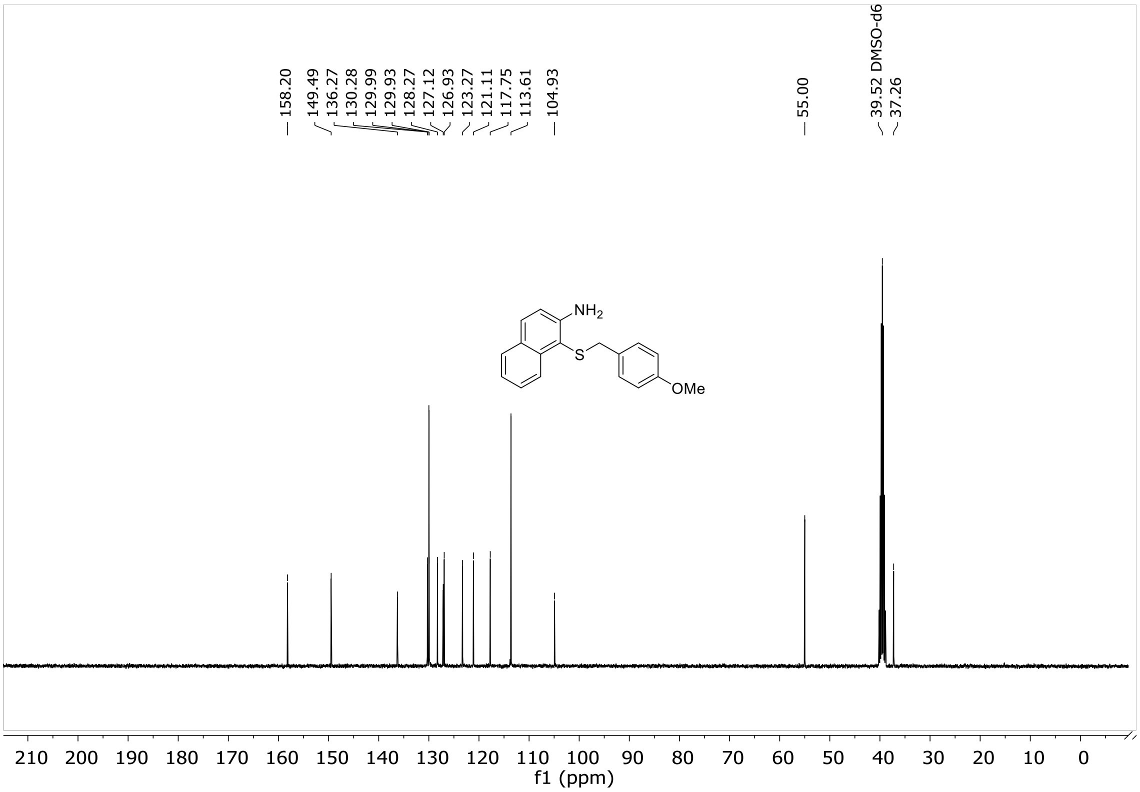


**Figure S050**: $^{13}C\{^1H\}$ NMR spectrum of 1-((4-methoxybenzyl)thio)naphthalen-2-amine (**2d**) (100 MHz, DMSO-$d_6$, 298 K).

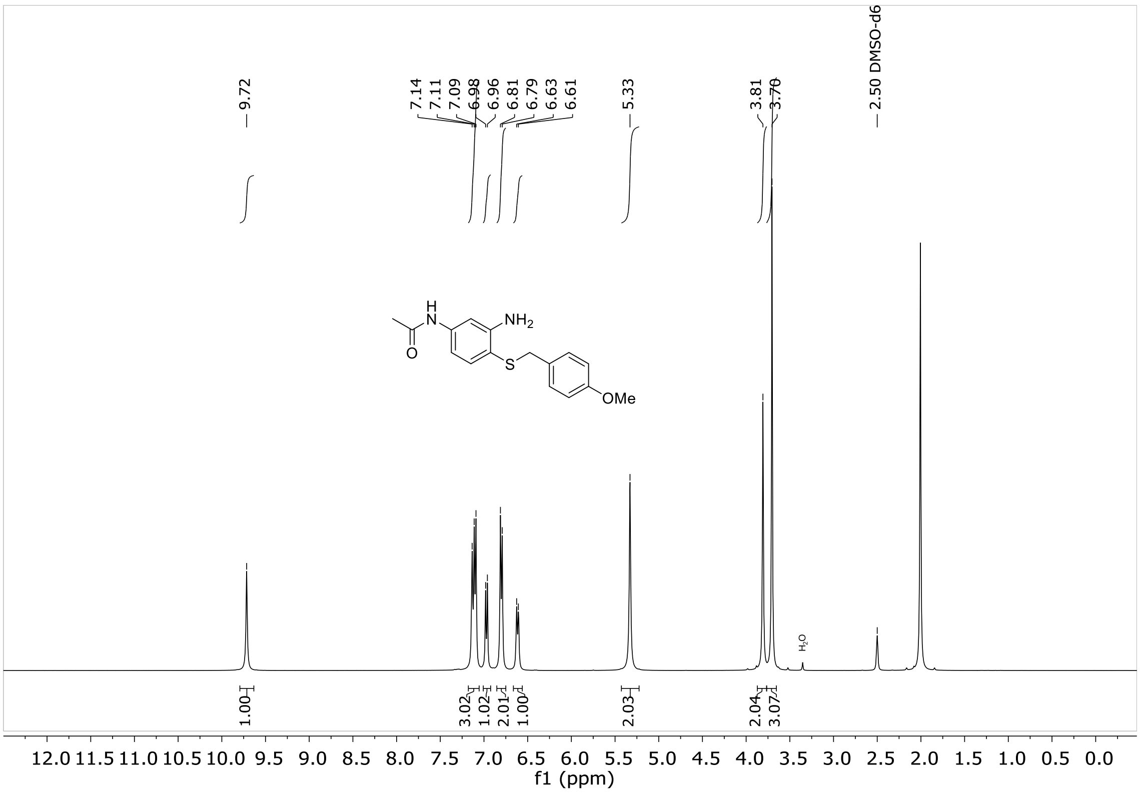


**Figure S051**: $^{1}H$ NMR spectrum of *N*-(3-amino-4-((4-methoxybenzyl)thio)phenyl)acetamide (**2f**) (400 MHz, DMSO-$d_6$, 298 K).

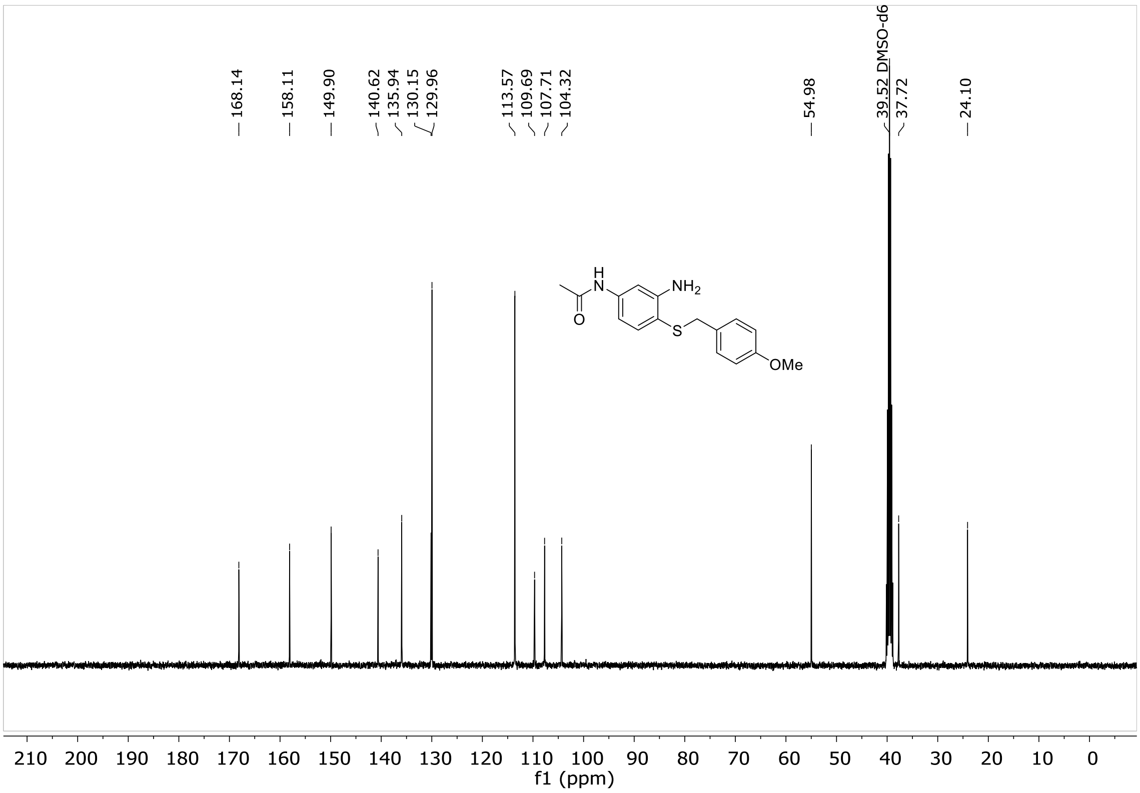


**Figure S052**: $^{13}C\{^{1}H\}$ NMR spectrum of *N*-(3-amino-4-((4-methoxybenzyl)thio)phenyl)acetamide (**2f**) (100 MHz, DMSO-$d_6$, 298 K).

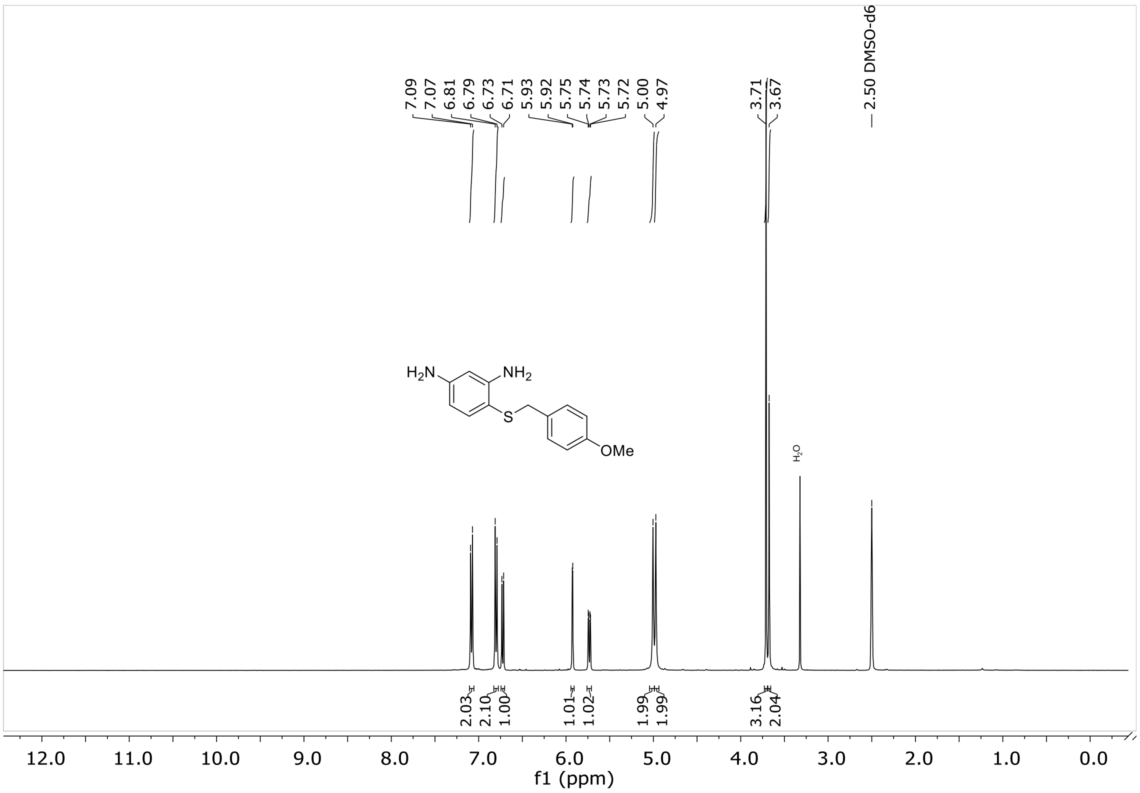


**Figure S053**: $^{1}H$ NMR spectrum of 4-((4-methoxybenzyl)thio)benzene-1,3-diamine (**2e**) (400 MHz, DMSO-$d_6$, 298 K).

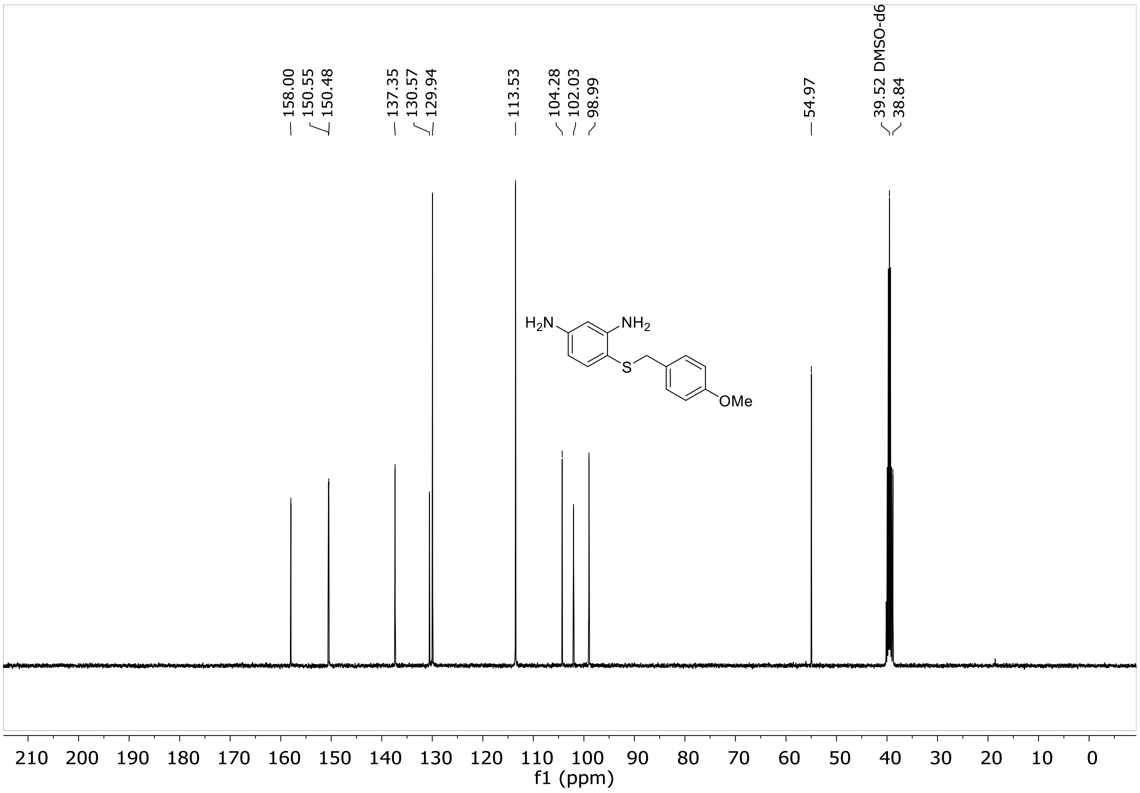


**Figure S054**: $^{13}C\{^{1}H\}$ NMR spectrum of 4-((4-methoxybenzyl)thio)benzene-1,3-diamine (**2e**) (100 MHz, DMSO-$d_6$, 298 K).

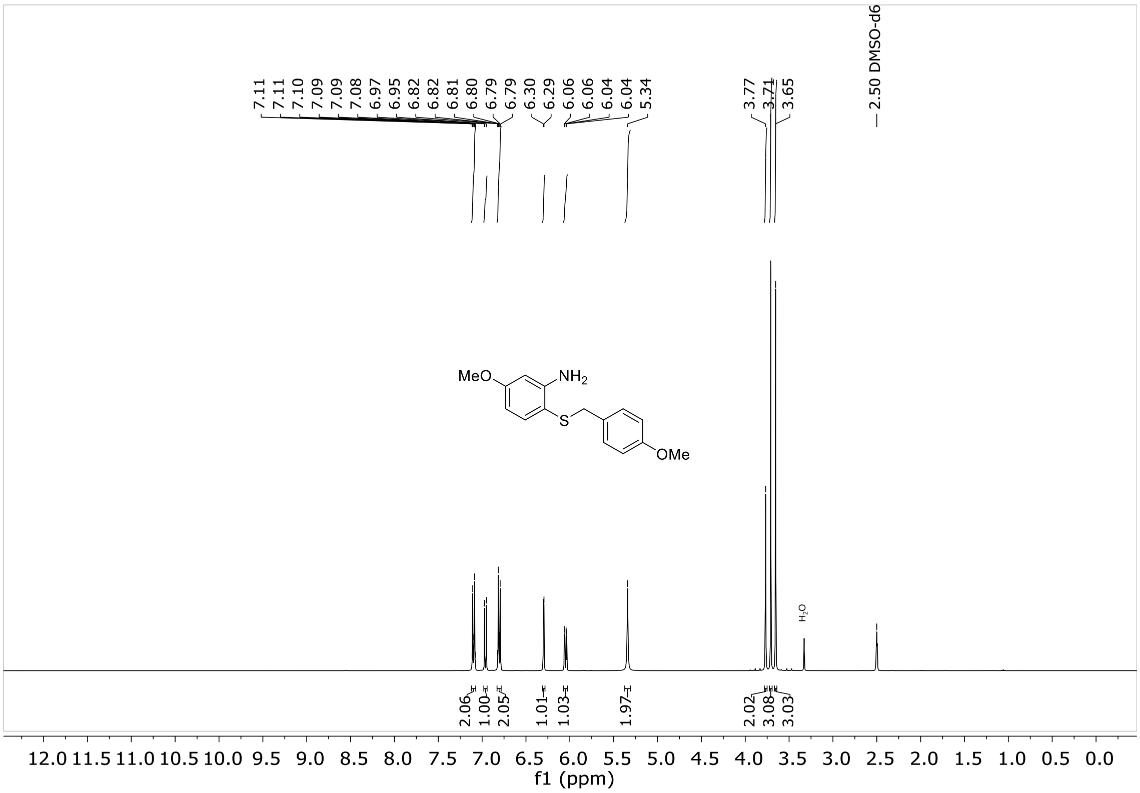


**Figure S055**: $^{1}$H NMR spectrum of 5-methoxy-2-((4-methoxybenzyl)thio)aniline (**2h)** (400 MHz, DMSO-$d_6$, 298 K).

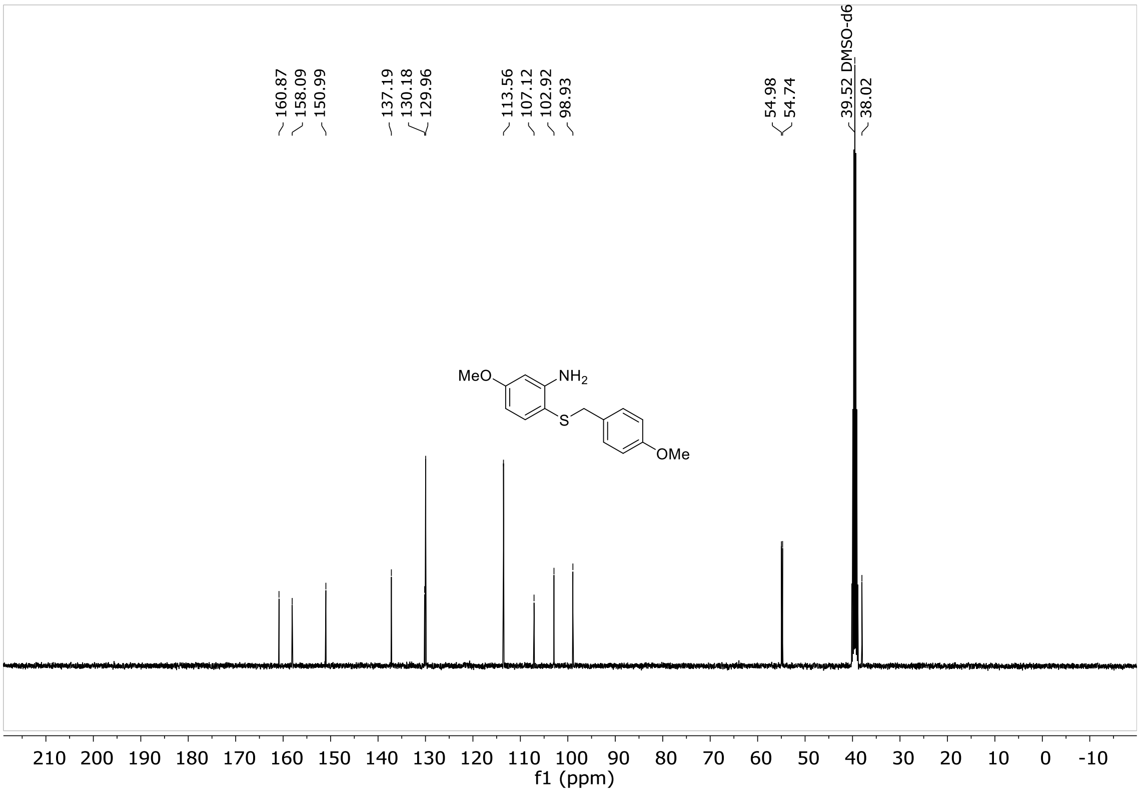


**Figure S056**: $^{13}$C{$^{1}$H} NMR spectrum of 5-methoxy-2-((4-methoxybenzyl)thio)aniline (**2H)** (100 MHz, DMSO-$d_6$, 298 K).

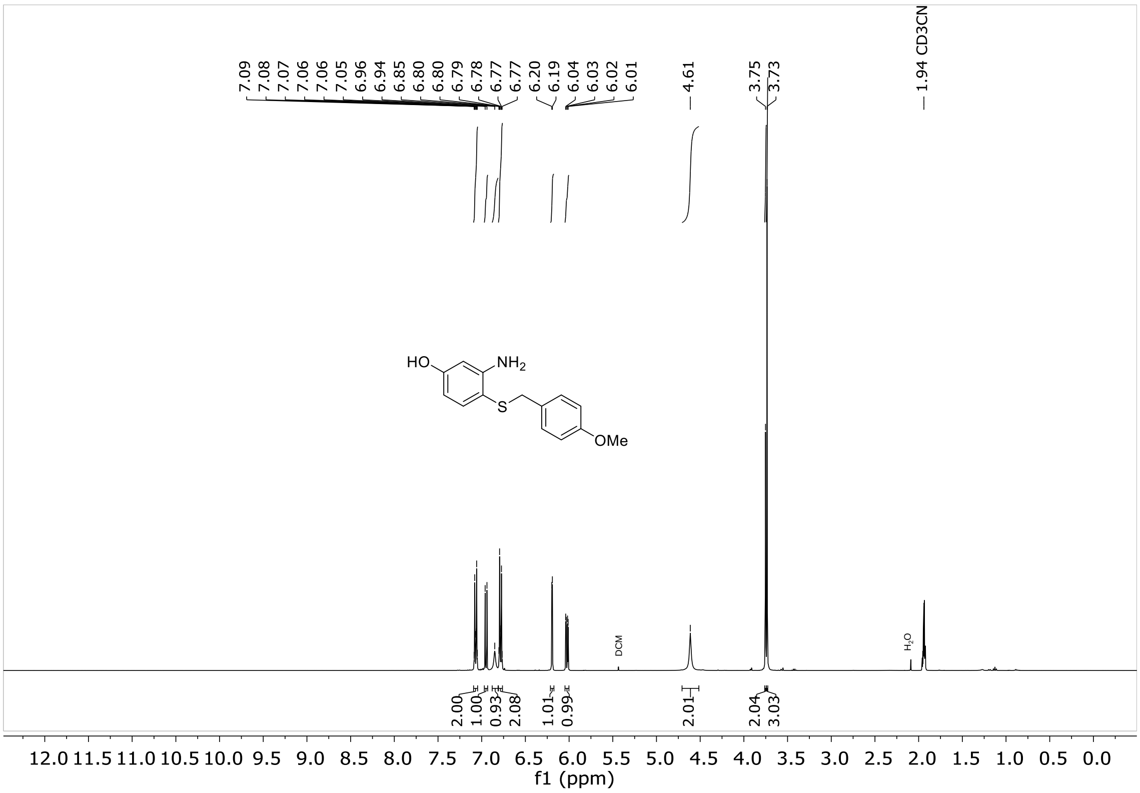


**Figure S057**: $^{1}H$ NMR spectrum of 3-amino-4-((4-methoxybenzyl)thio)phenol (**2H)** (400 MHz, DMSO-$d_6$, 298 K).

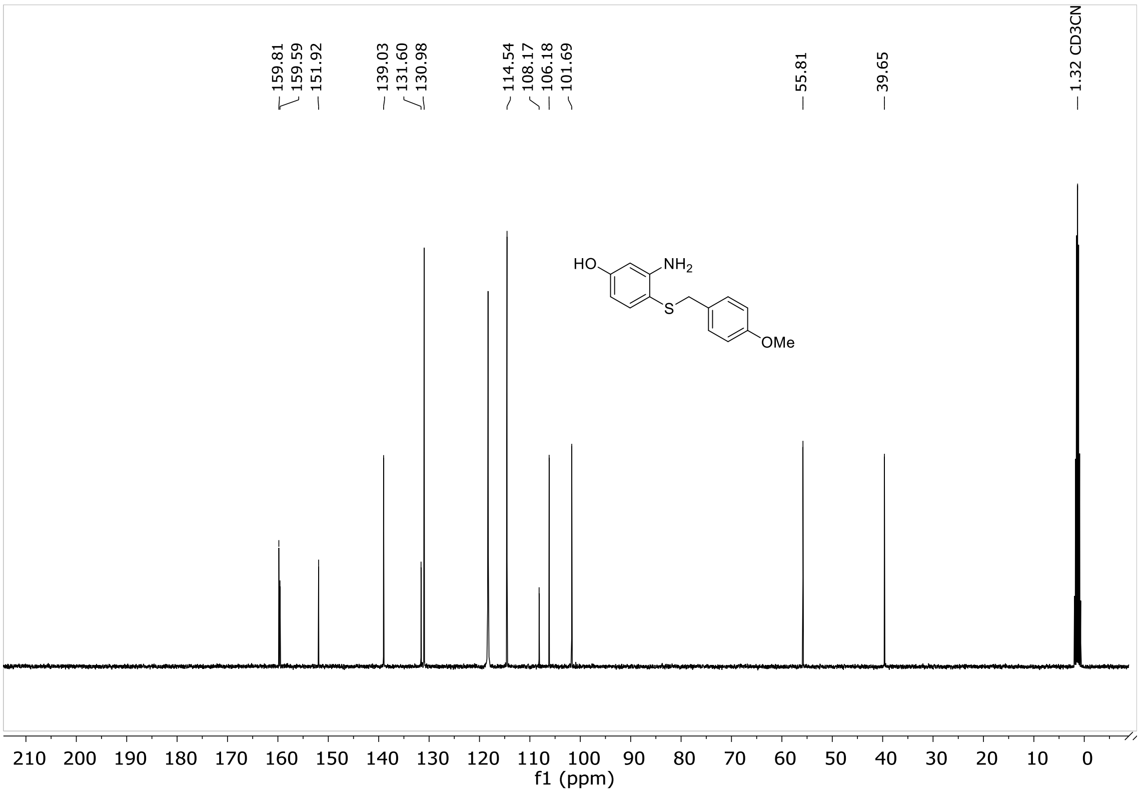


**Figure S058**: $^{13}C\{^{1}H\}$ NMR spectrum of 3-amino-4-((4-methoxybenzyl)thio)phenol (**2h)** (100 MHz, DMSO-$d_6$, 298 K).

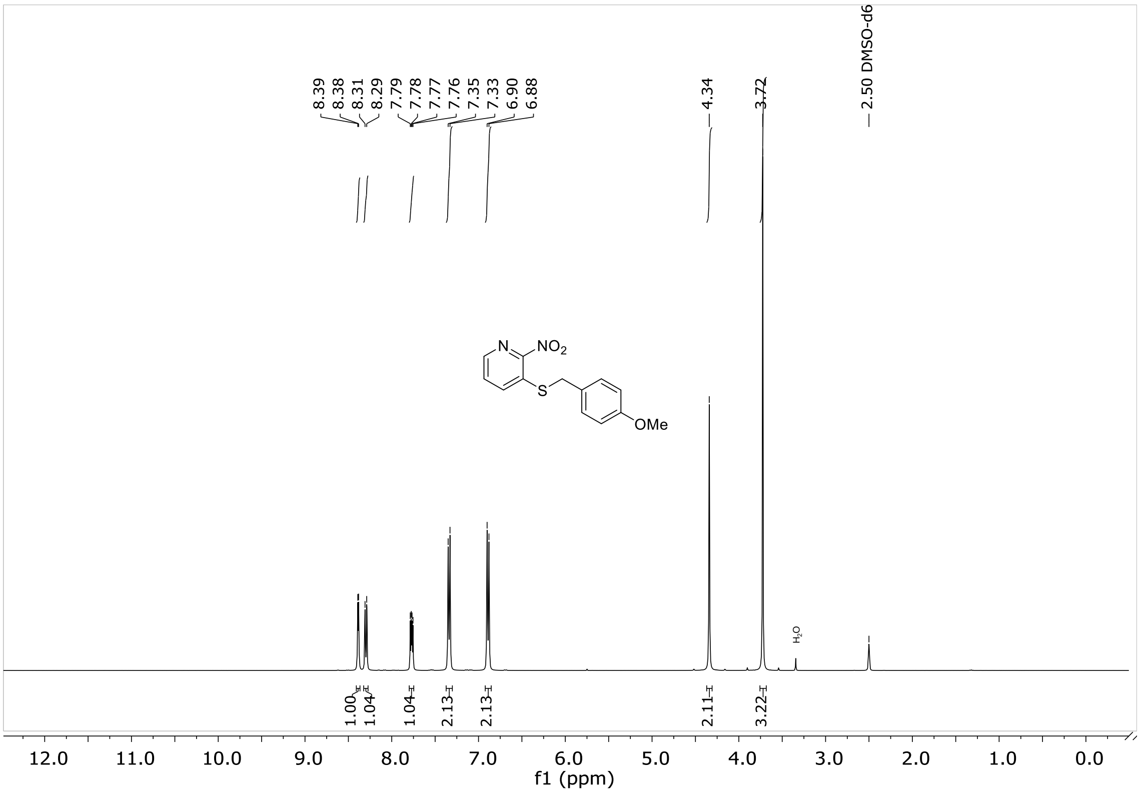


**Figure S059**: $^{1}$H NMR spectrum of 3-((4-methoxybenzyl)thio)pyridin-2-amine (**2i**) (400 MHz, DMSO-$d_6$, 298 K).

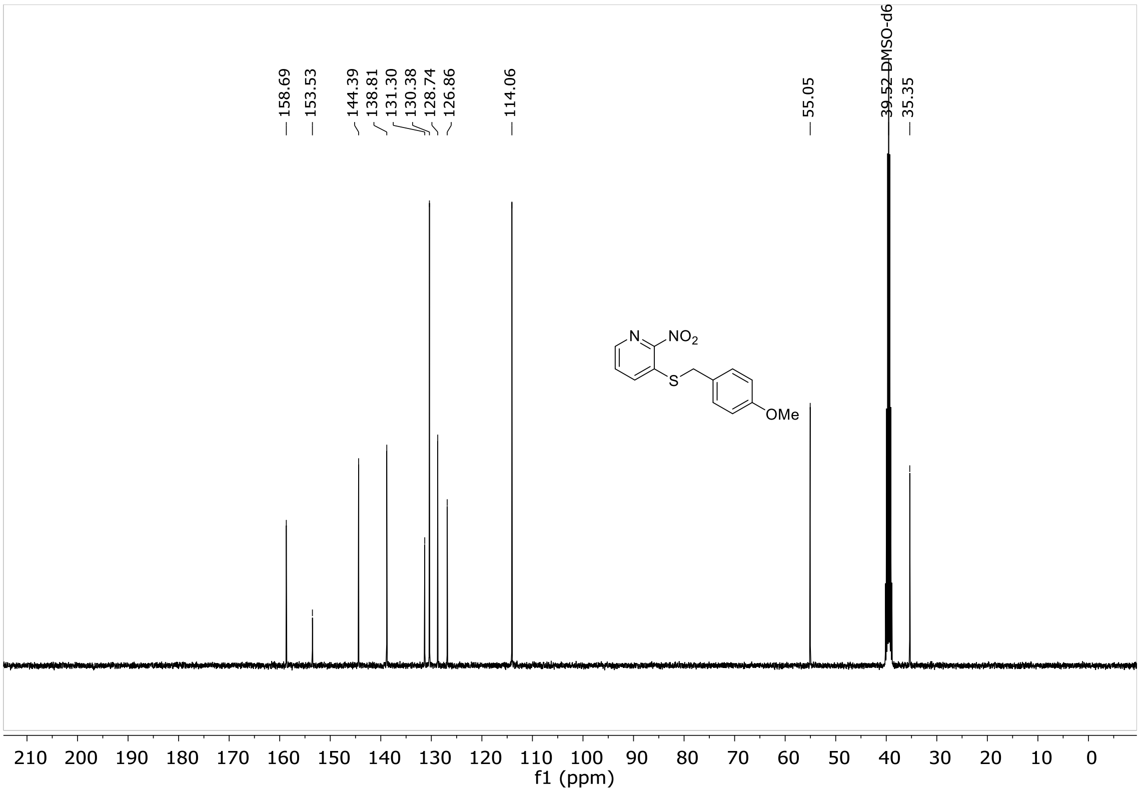


**Figure S060**: $^{13}$C{$^{1}$H} NMR spectrum of 3-((4-methoxybenzyl)thio)pyridin-2-amine (**2i**) (100 MHz, DMSO-$d_6$, 298 K).

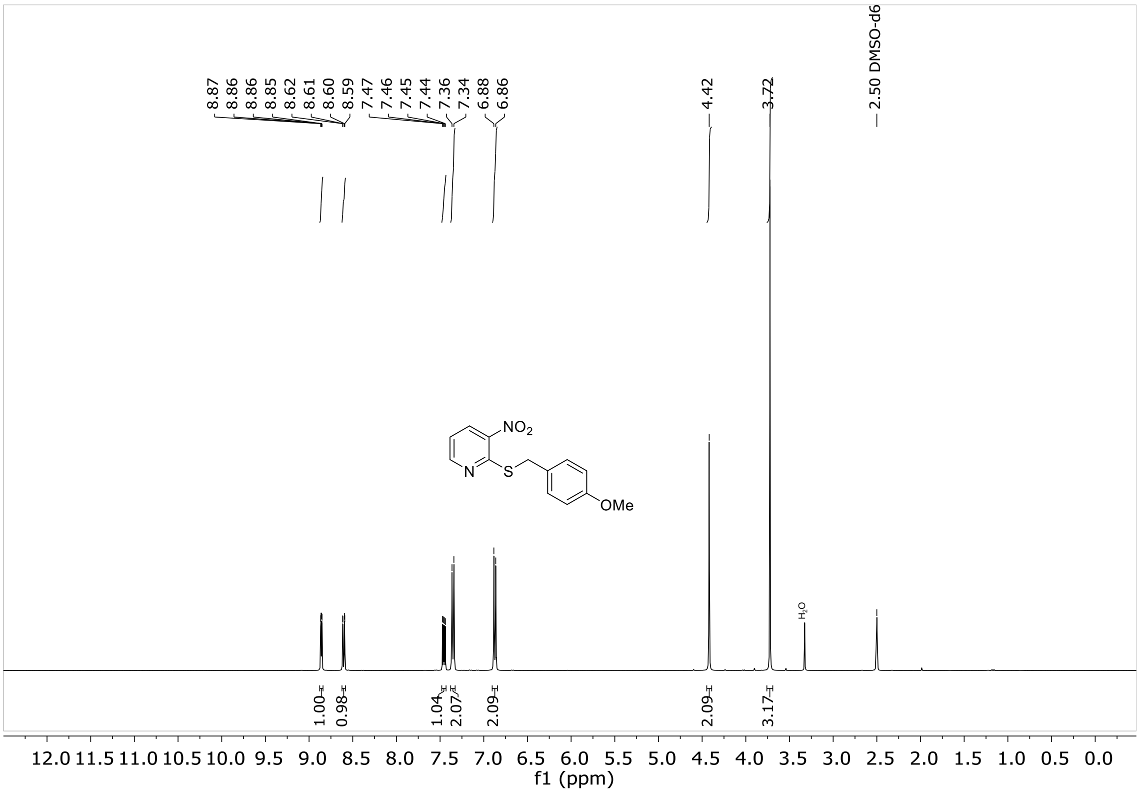

**Figure S061**: $^{1}$H NMR spectrum of 2-((4-methoxybenzyl)thio)pyridin-3-amine (**2j**) (400 MHz, DMSO-*d*$_6$, 298 K).

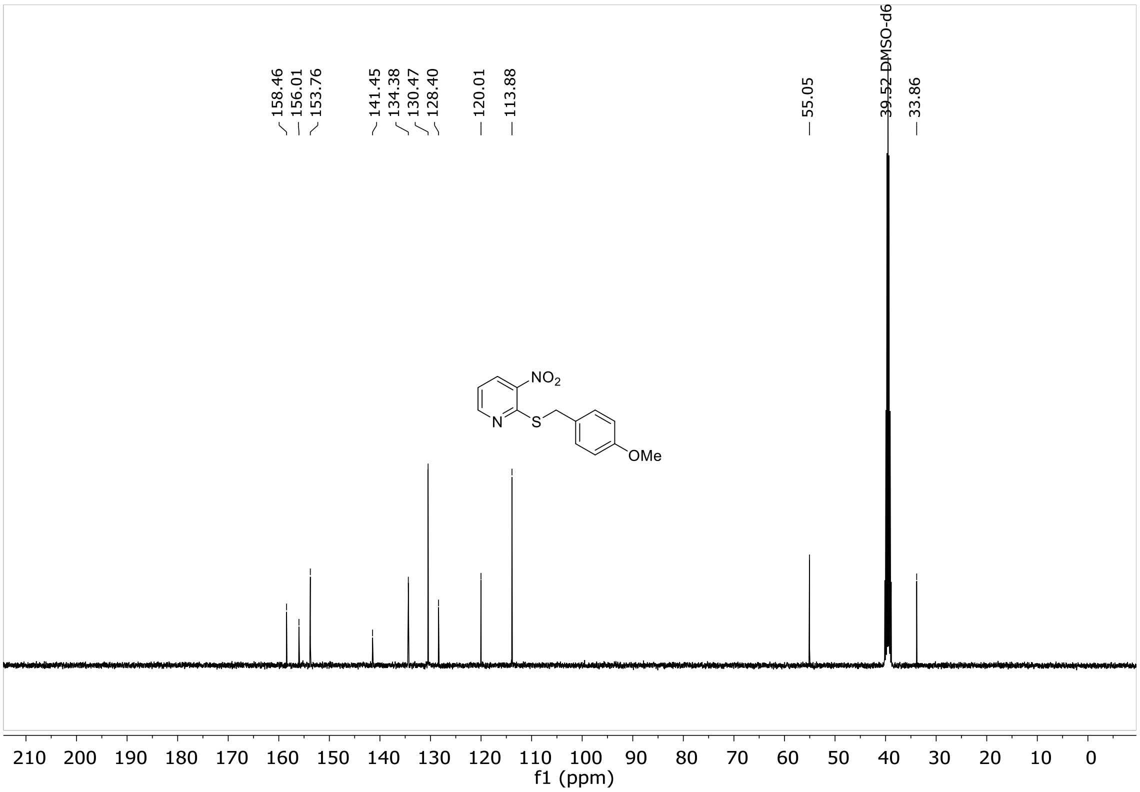

**Figure S062**: $^{13}$C{$^{1}$H} NMR spectrum of 2-((4-methoxybenzyl)thio)pyridin-3-amine (**2j**) (100 MHz, DMSO-*d*$_6$, 298 K).

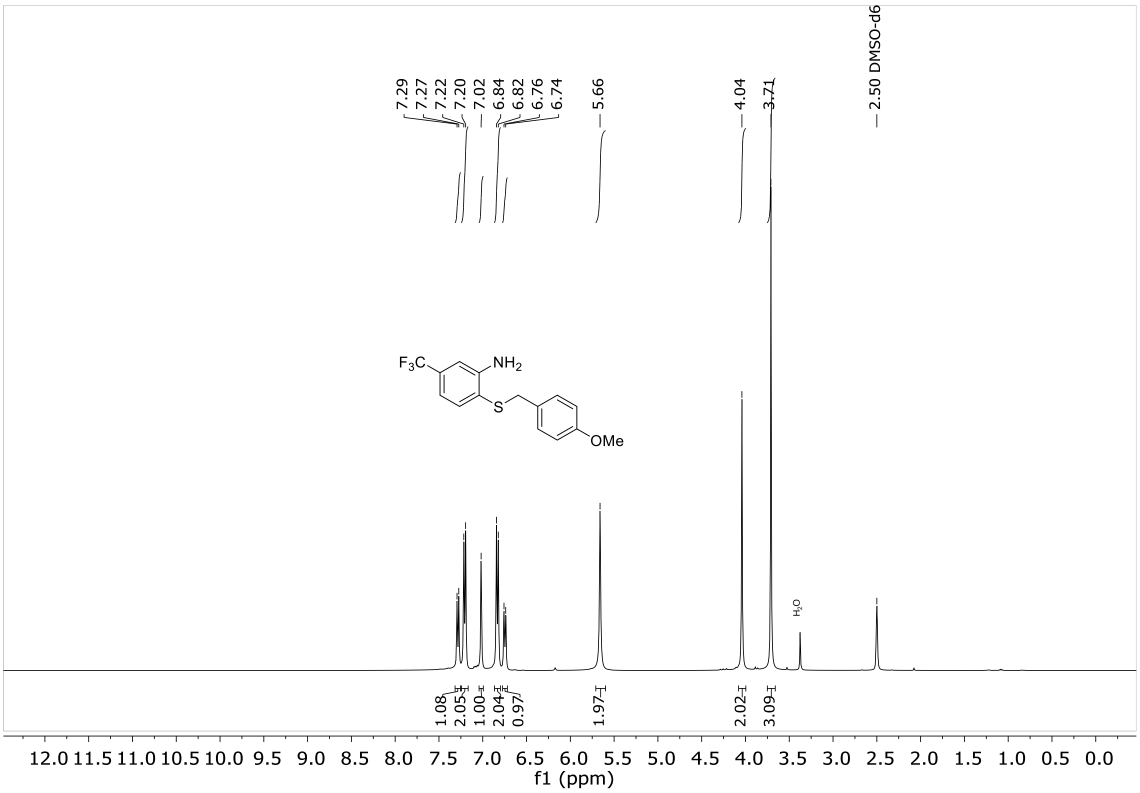


**Figure S063**: $^{1}H$ NMR spectrum of 2-((4-methoxybenzyl)thio)-5-(trifluoromethyl)aniline (**2k**) (400 MHz, DMSO-*d*$_6$, 298 K).

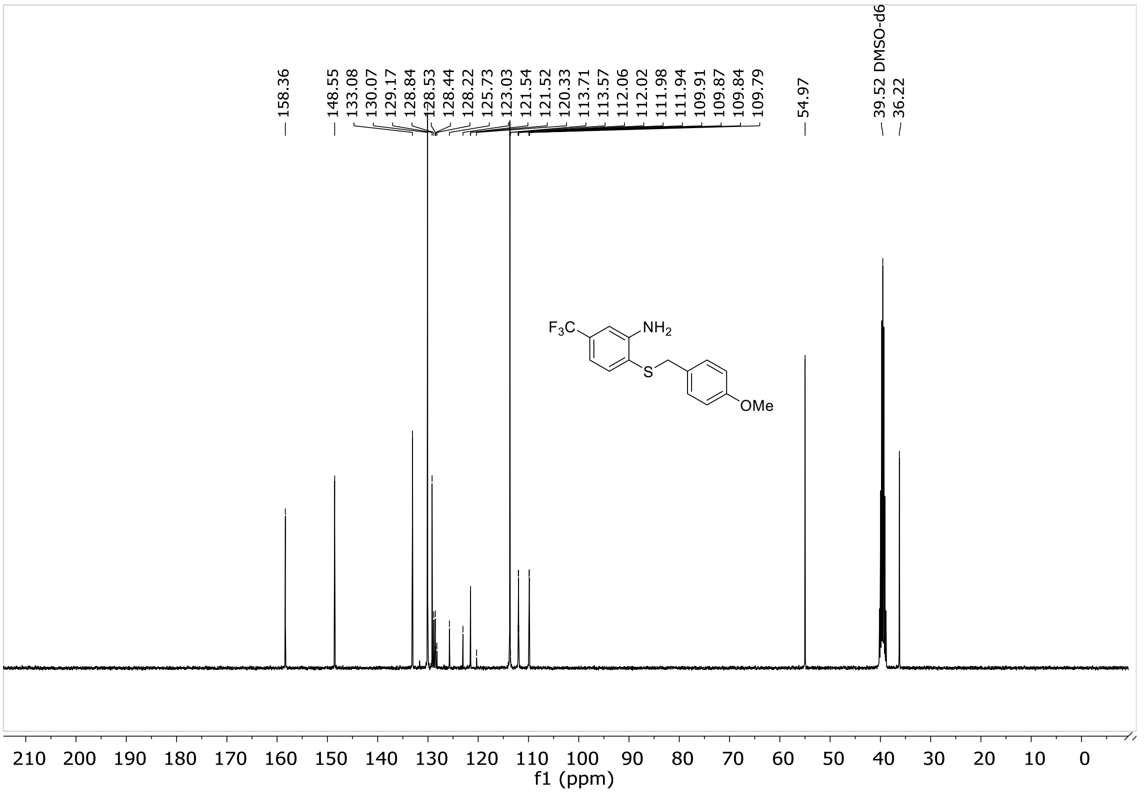


**Figure S064**: $^{13}C\{^{1}H\}$ NMR spectrum of 2-((4-methoxybenzyl)thio)-5-(trifluoromethyl)aniline (**2k**) (100 MHz, DMSO-*d*$_6$, 298 K).

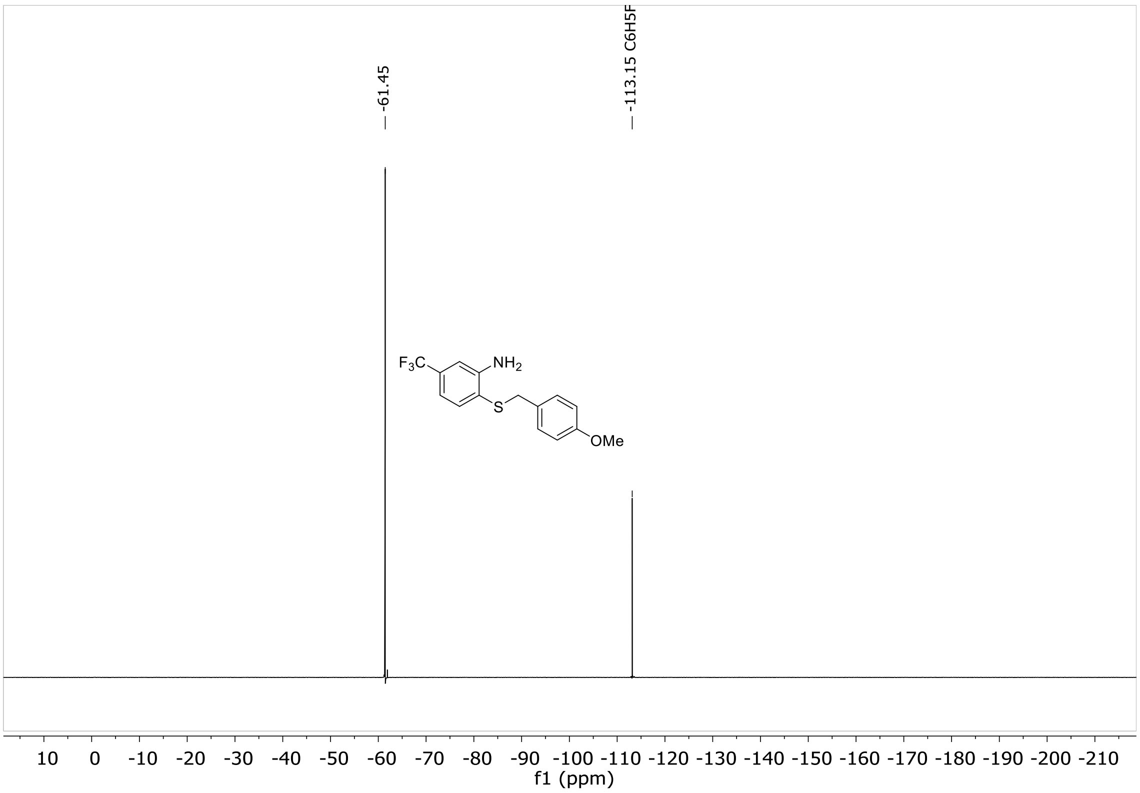

**Figure S065**: $^{19}F\{^1H\}$ NMR spectrum of 2-((4-methoxybenzyl)thio)-5-(trifluoromethyl)aniline (**2k**) (3760 MHz, DMSO-$d_6$, 298 K, referenced to fluorobenzene).

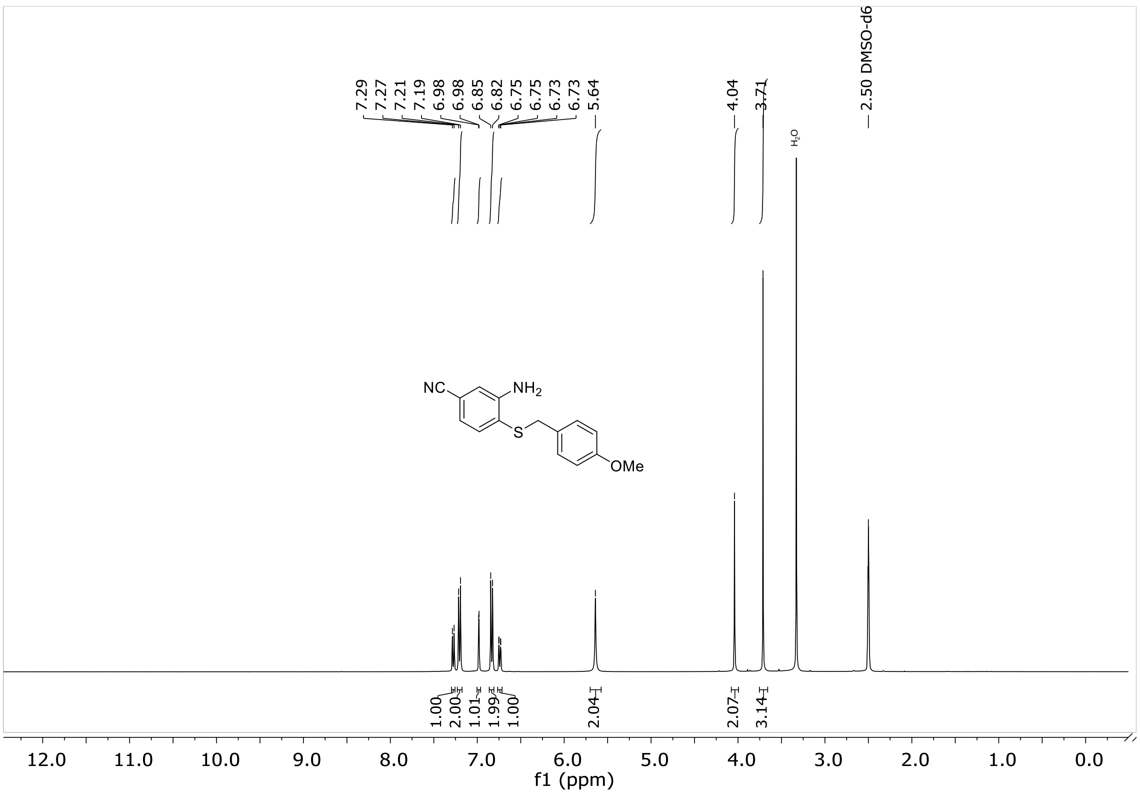

**Figure S066**: $^1H$ NMR spectrum of 3-amino-4-((4-methoxybenzyl)thio)benzonitrile (**2l**) (400 MHz, DMSO-$d_6$, 298 K).

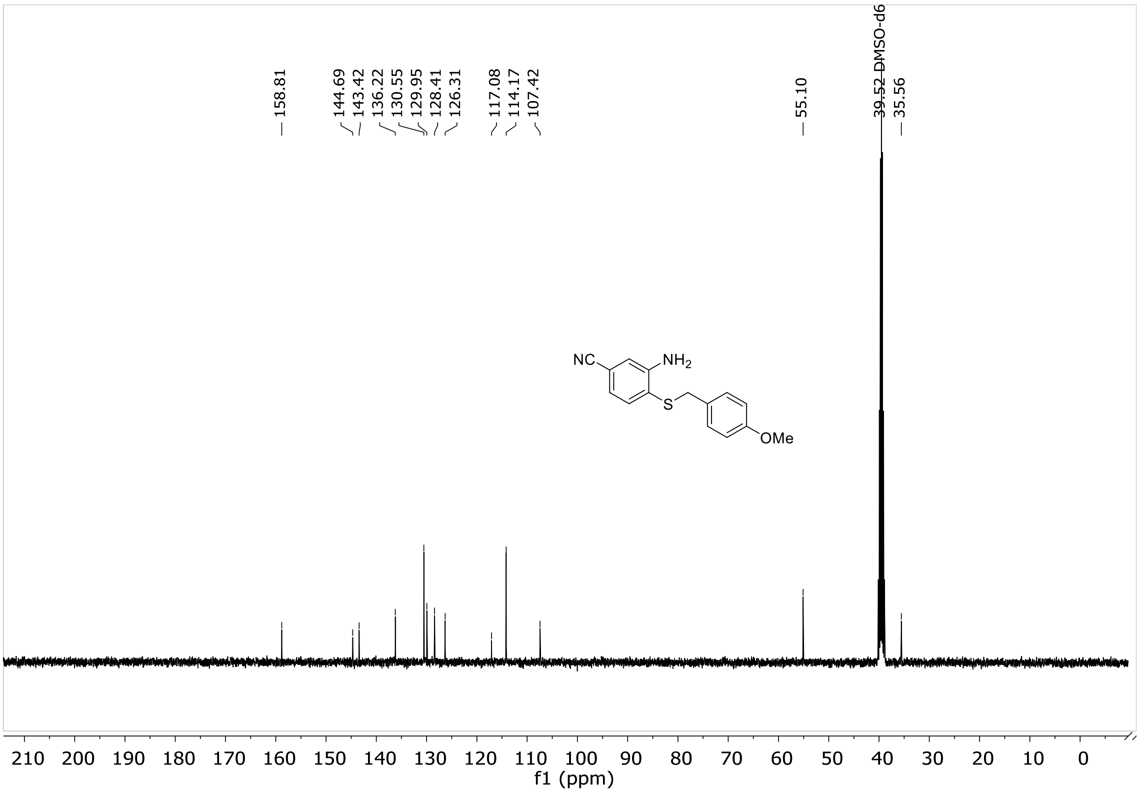


**Figure S067**: $^{13}C\{^{1}H\}$ NMR spectrum of 3-amino-4-((4-methoxybenzyl)thio)benzonitrile (**2l**) (100 MHz, DMSO-*d*$_6$, 298 K).

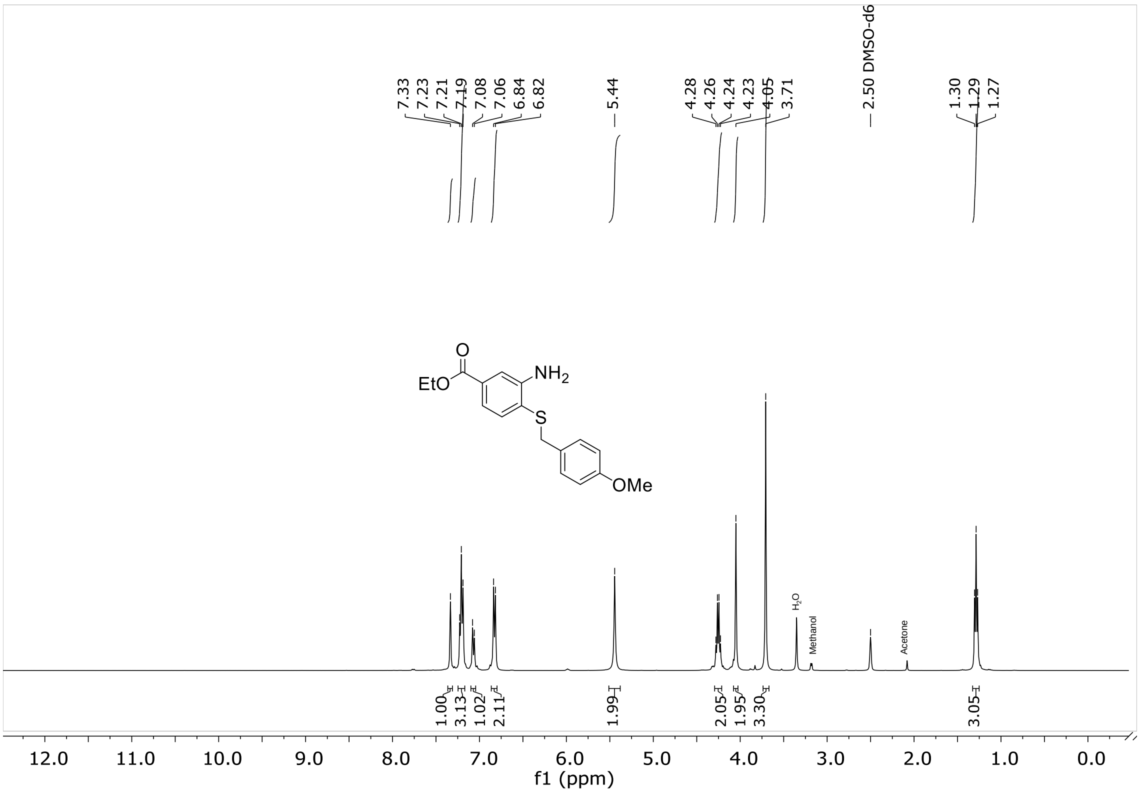


**Figure S068**: $^{1}H$ NMR spectrum of ethyl 3-amino-4-((4-methoxybenzyl)thio)benzoate (**2m**) (400 MHz, DMSO-*d*$_6$, 298 K).

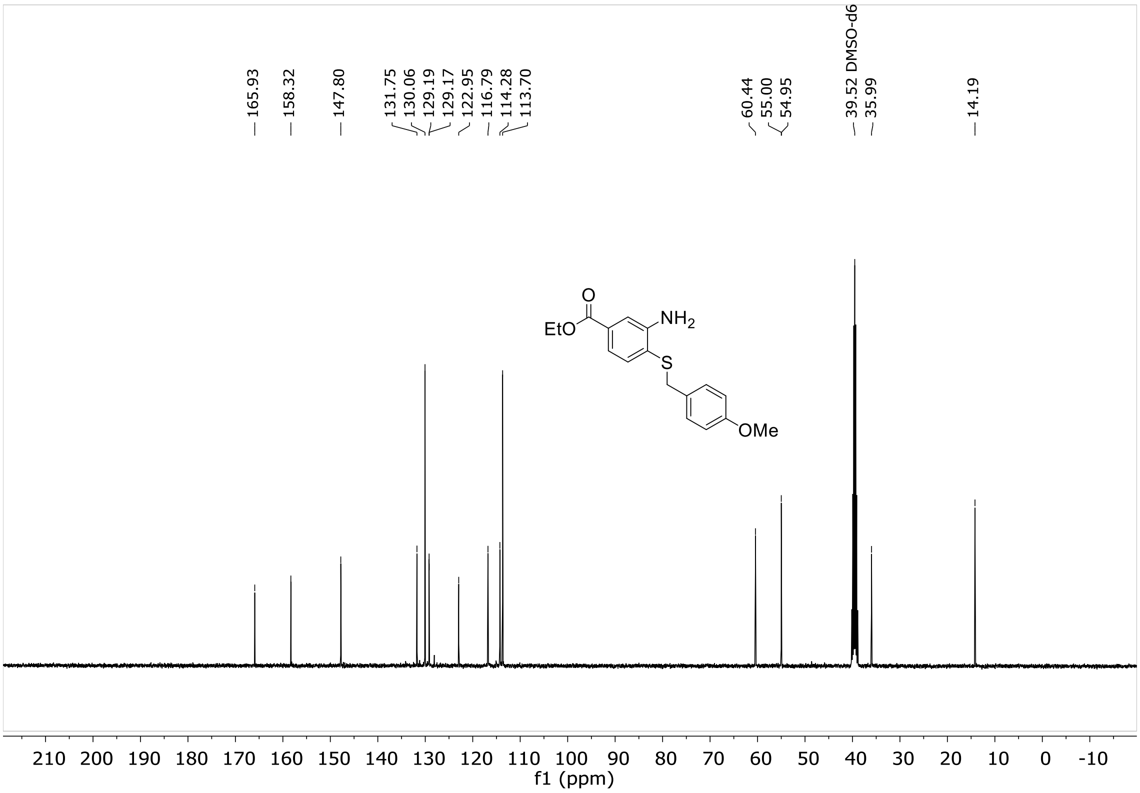


**Figure S069**: $^{13}C\{^1H\}$ NMR spectrum of ethyl 3-amino-4-((4-methoxyben zyl)thio)benzoate (**2m**) (100 MHz, DMSO-$d_6$, 298 K).

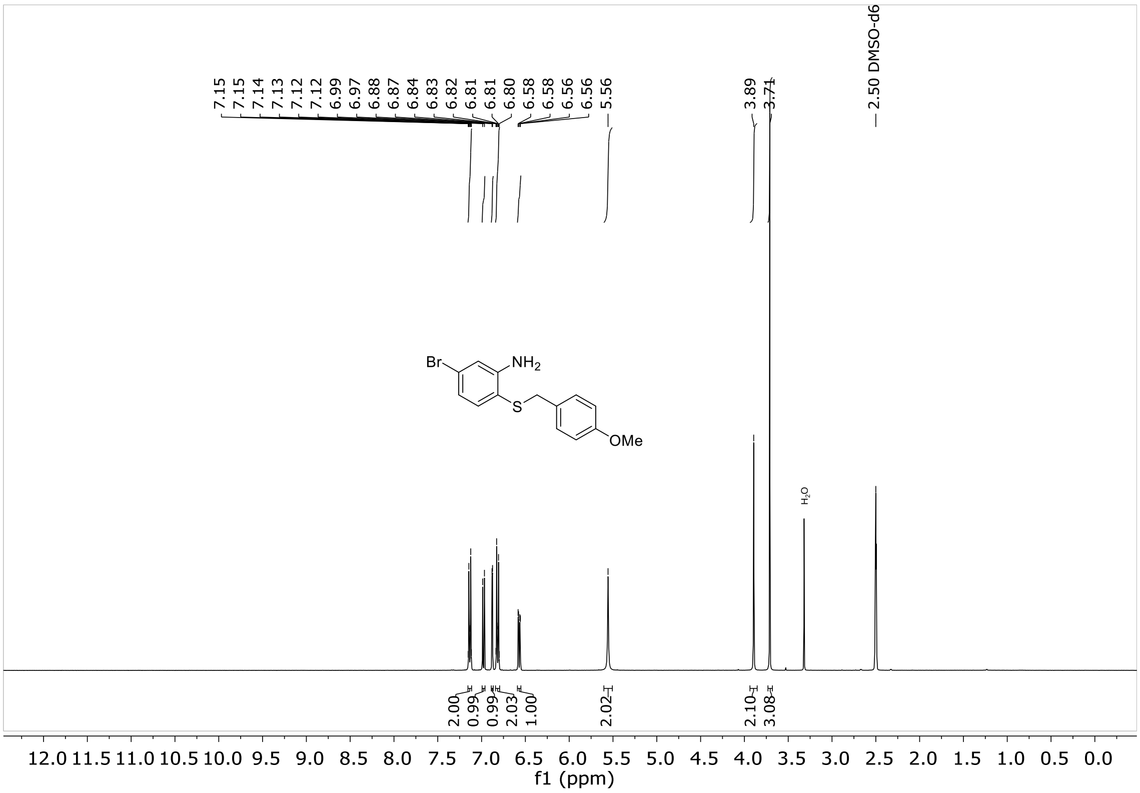


**Figure S070**: $^1H$ NMR spectrum of 5-bromo-2-((4-methoxybenzyl)thio)aniline (**2n**) (400 MHz, DMSO-$d_6$, 298 K).

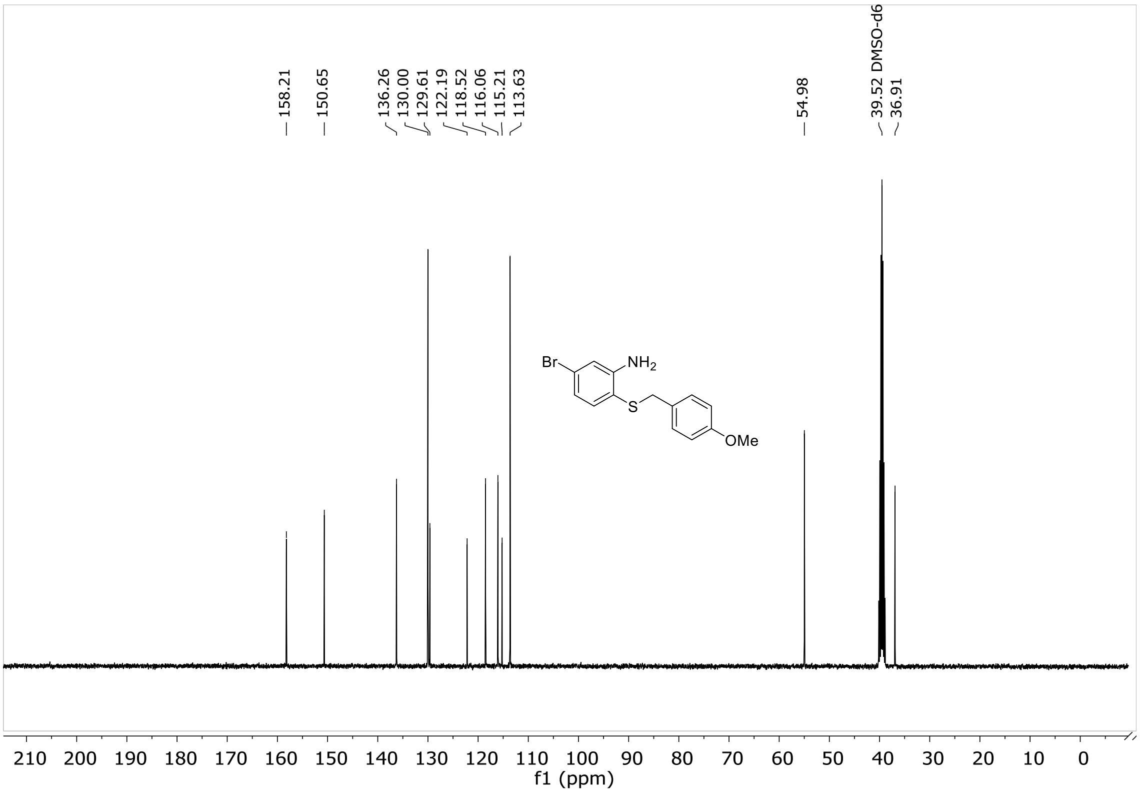

**Figure S071**: $^{13}C\{^{1}H\}$ NMR spectrum of 5-bromo-2-((4-methoxybenzyl)thio)aniline (**2n**) (100 MHz, DMSO-$d_6$, 298 K).

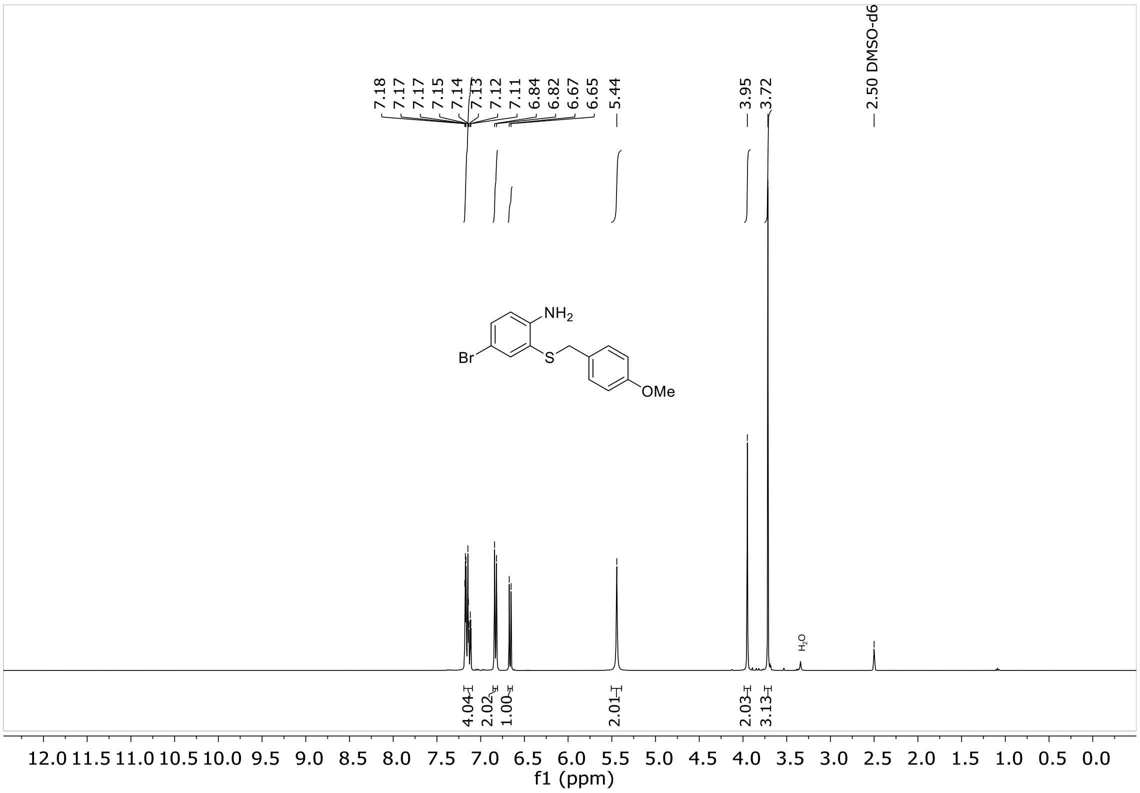

**Figure S072**: $^{1}H$ NMR spectrum of 4-bromo-2-((4-methoxybenzyl)thio)aniline (**2o**) (400 MHz, DMSO-$d_6$, 298 K).

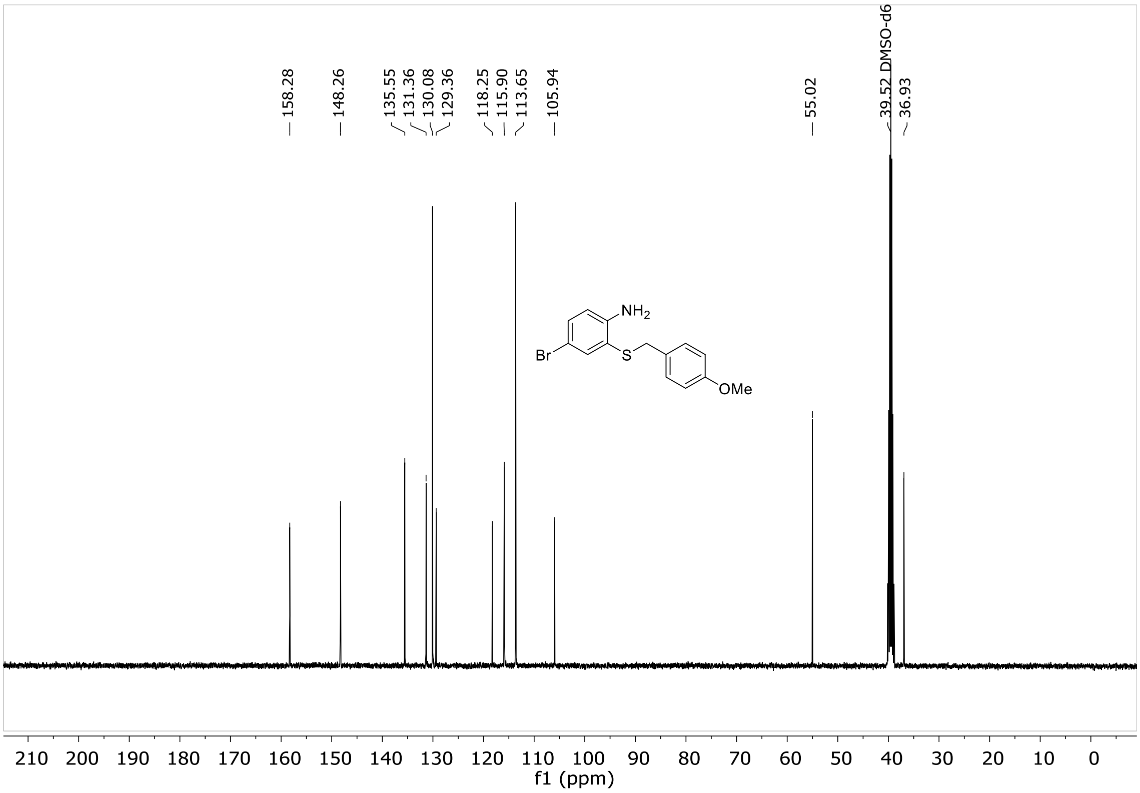


**Figure S073**: $^{13}C\{^{1}H\}$ NMR spectrum of 4-bromo-2-((4-methoxybenzyl)thio)aniline (**2o**) (100 MHz, DMSO-$d_6$, 298 K).

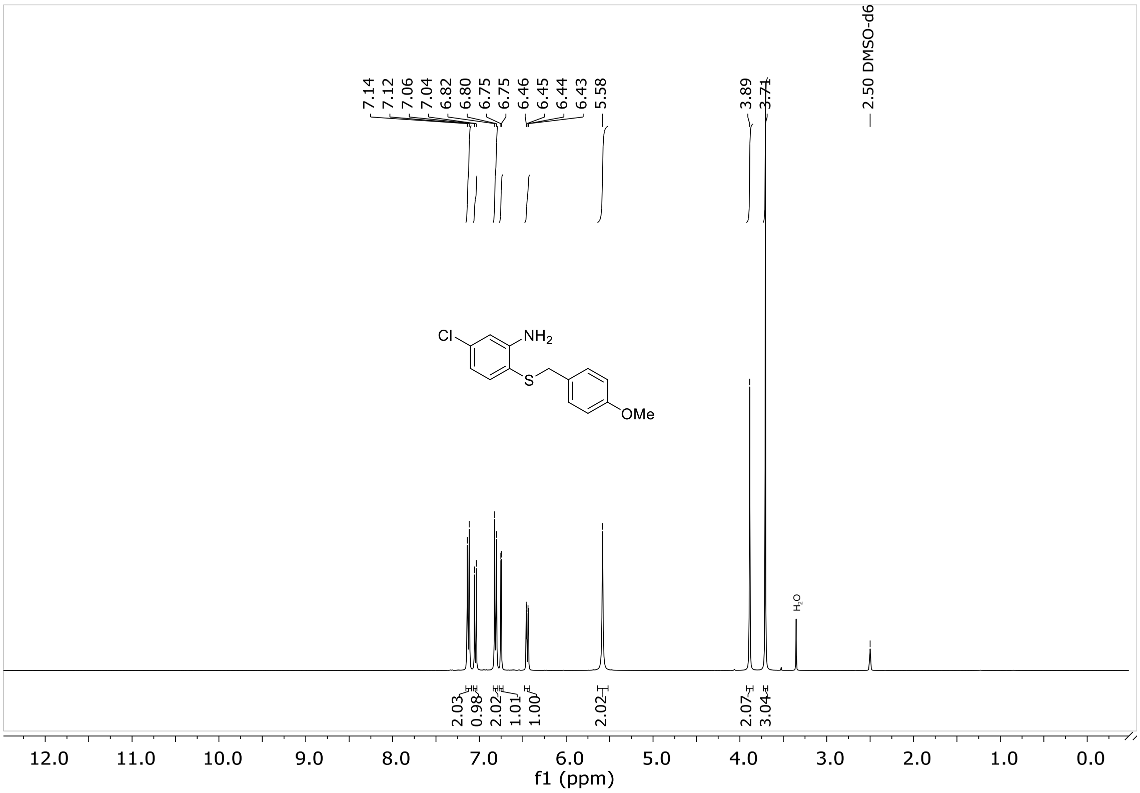


**Figure S074**: $^{1}H$ NMR spectrum of 5-chloro-2-((4-methoxybenzyl)thio)aniline (**2p**) (400 MHz, DMSO-$d_6$, 298 K).

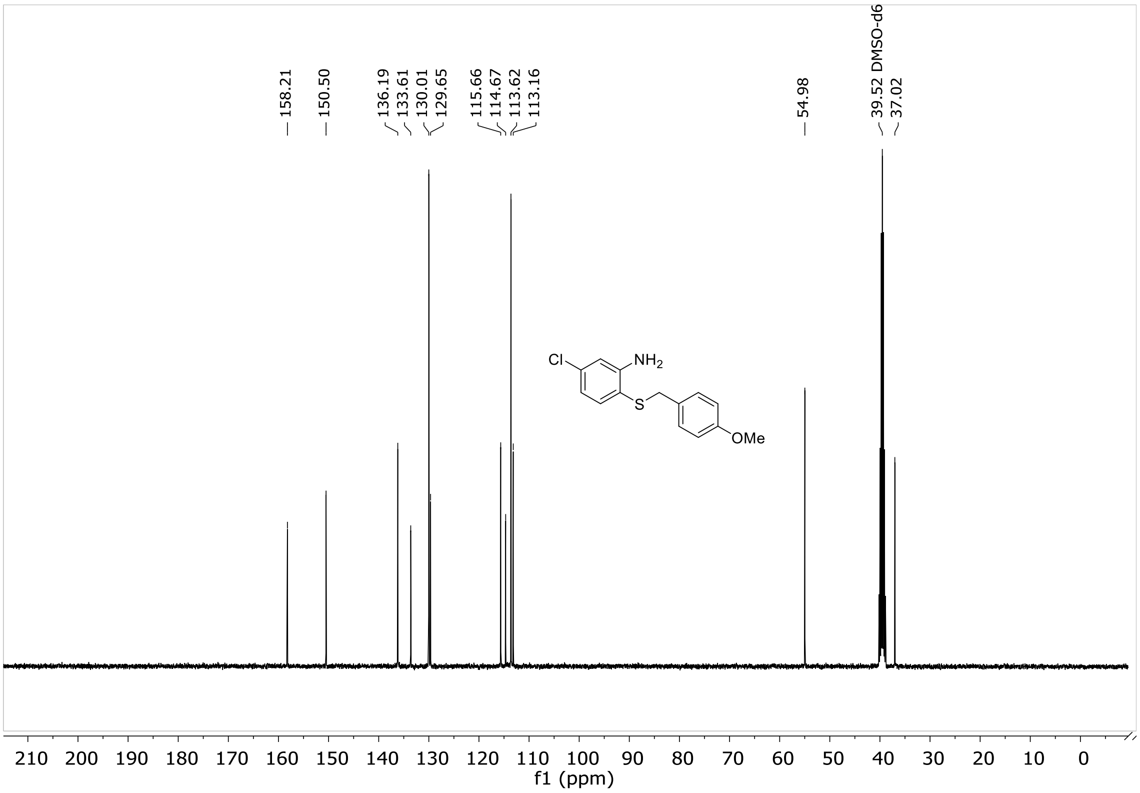


**Figure S075**: $^{13}C\{^{1}H\}$ NMR spectrum of 5-chloro-2-((4-methoxybenzyl)thio)aniline (**2p**) (100 MHz, DMSO-$d_6$, 298 K).

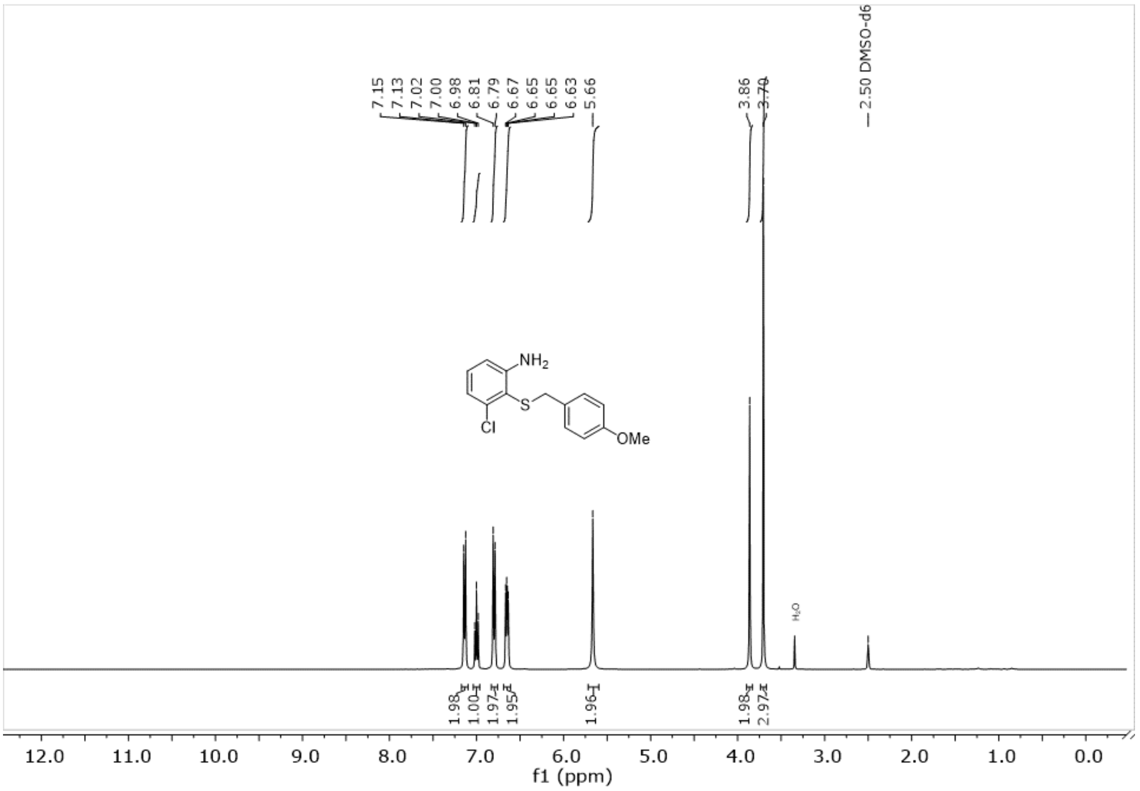


**Figure S076**: $^{1}H$ NMR spectrum of 3-chloro-2-((4-methoxybenzyl)thio)aniline (**2q**) (400 MHz, DMSO-$d_6$, 298 K).

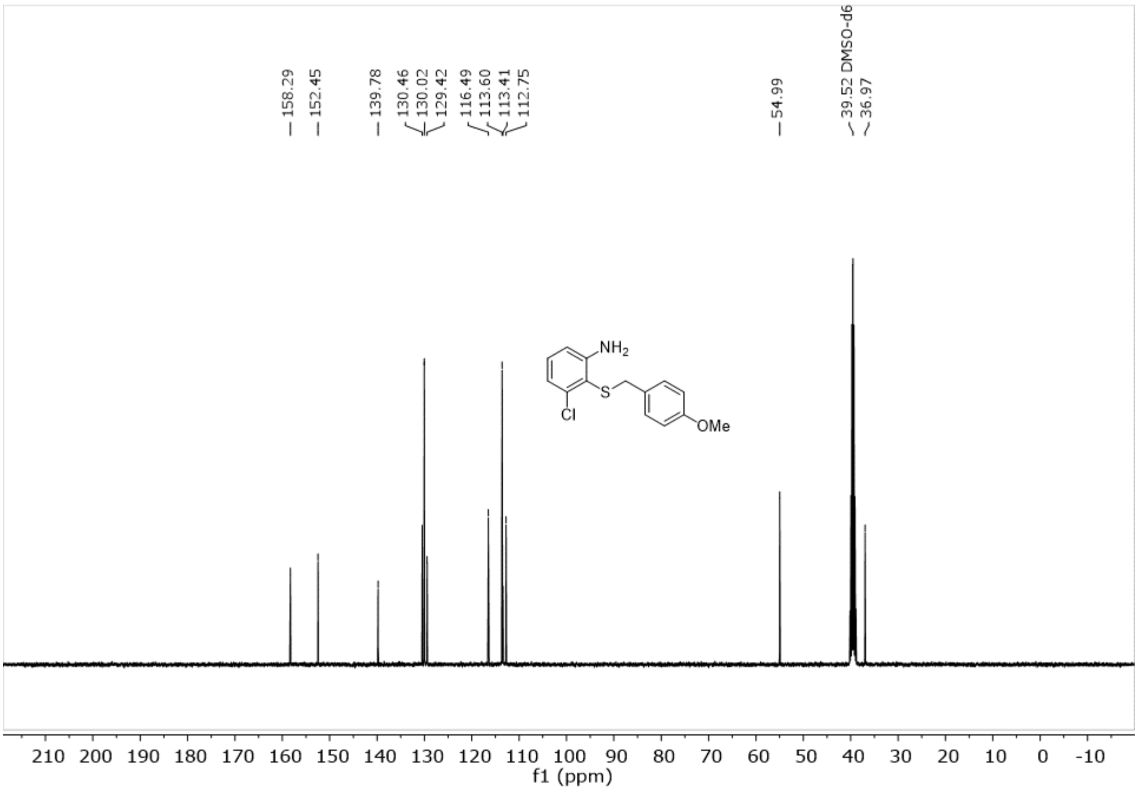

**Figure S077**: $^{13}C\{^{1}H\}$ NMR spectrum of 3-chloro-2-((4-methoxybenzyl)thio)aniline (**2q**) (100 MHz, DMSO-$d_6$, 298 K).

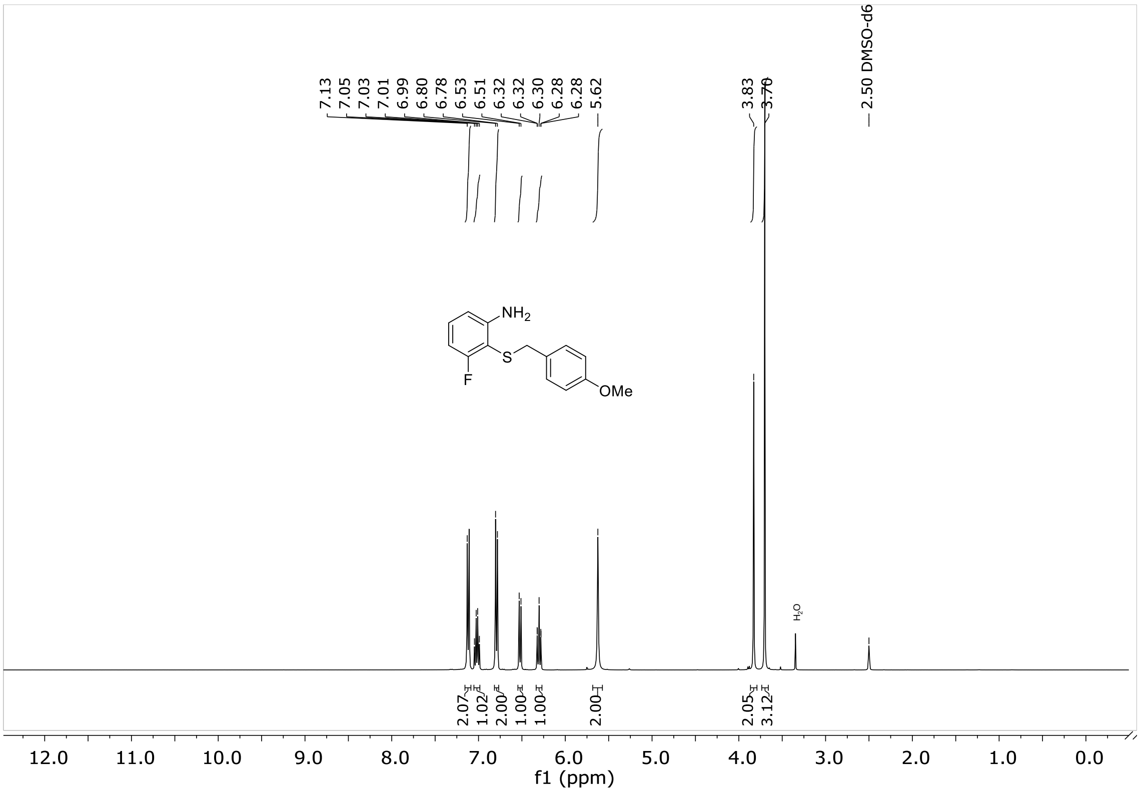

**Figure S078**: $^{1}H$ NMR spectrum of 3-fluoro-2-((4-methoxybenzyl)thio)aniline (**2r**) (400 MHz, DMSO-$d_6$, 298 K).

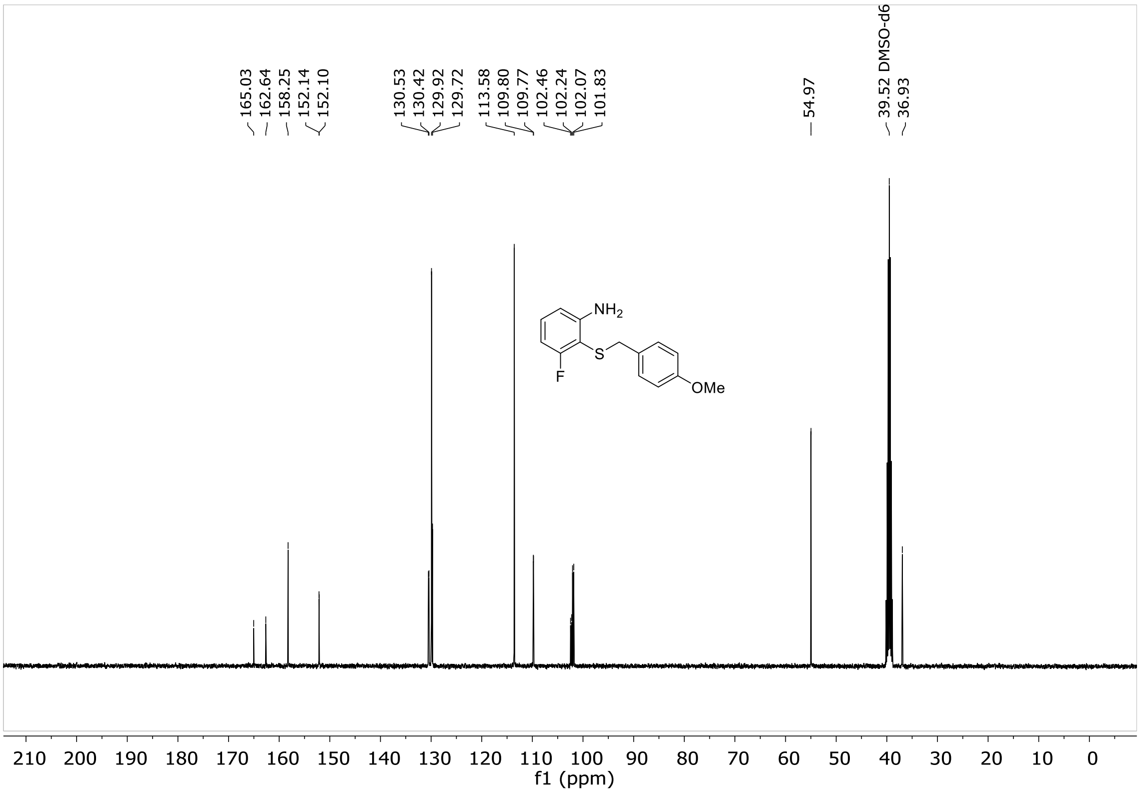


**Figure S079**: $^{13}C\{^{1}H\}$ NMR spectrum of 3-fluoro-2-((4-methoxybenzyl)thio)aniline (**2r**) (100 MHz, DMSO-*d*$_6$, 298 K).

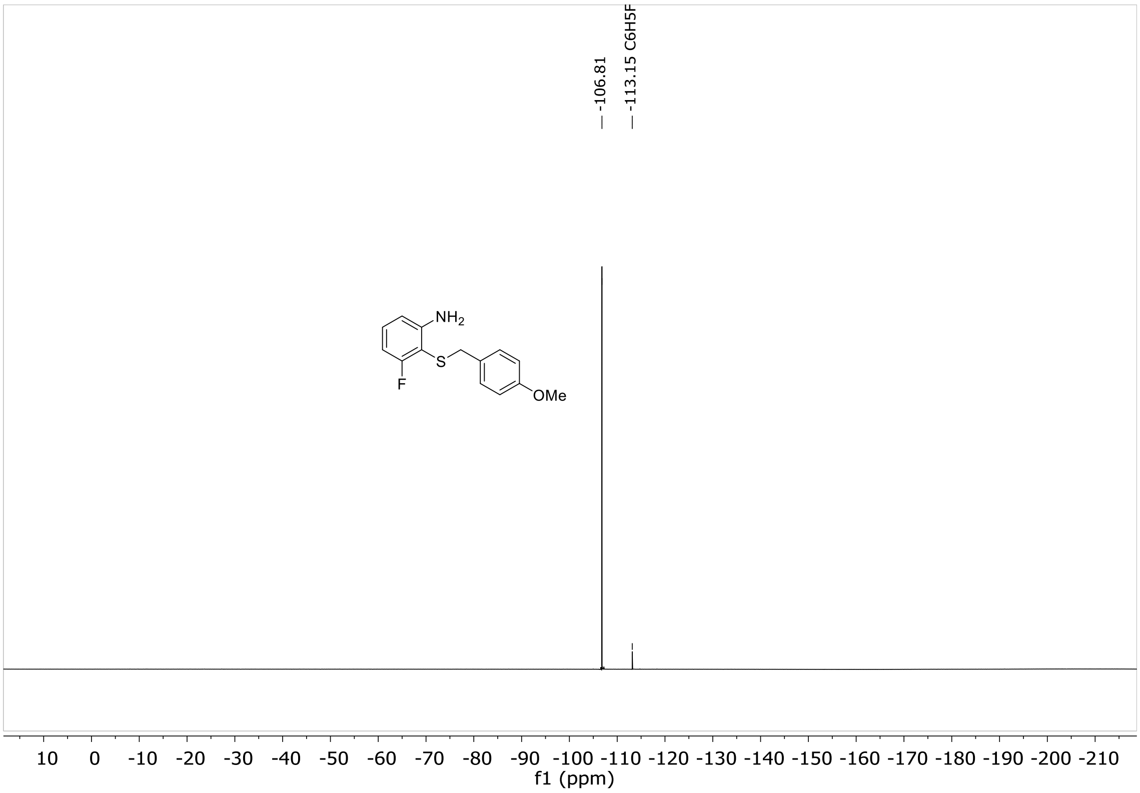


**Figure S080**: $^{19}F\{^{1}H\}$ NMR spectrum of 3-fluoro-2-((4-methoxybenzyl)thio)aniline (**2r**) (376 MHz, DMSO-*d*$_6$, 298 K, referenced to fluorobenzene).

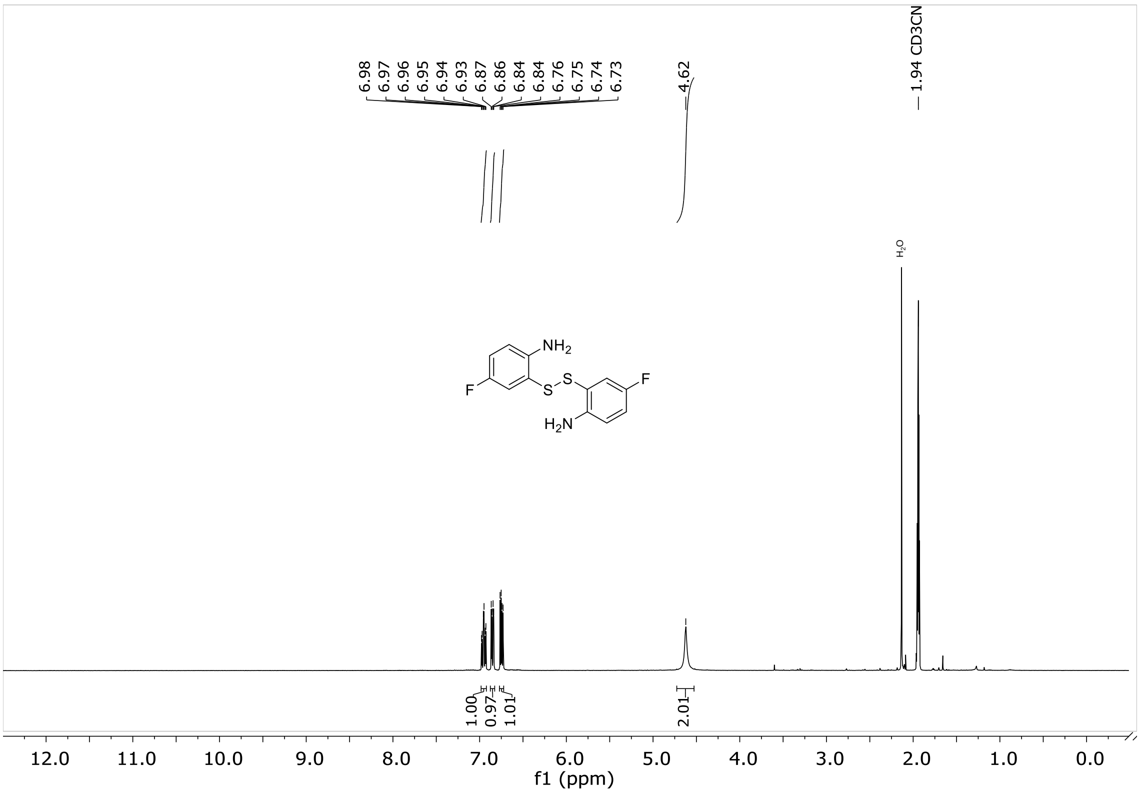


**Figure S081**: $^{1}H$ NMR spectrum of 2,2'-disulfanediylbis(4-fluoroaniline) (400 MHz, $CD_3CN$, 298 K).

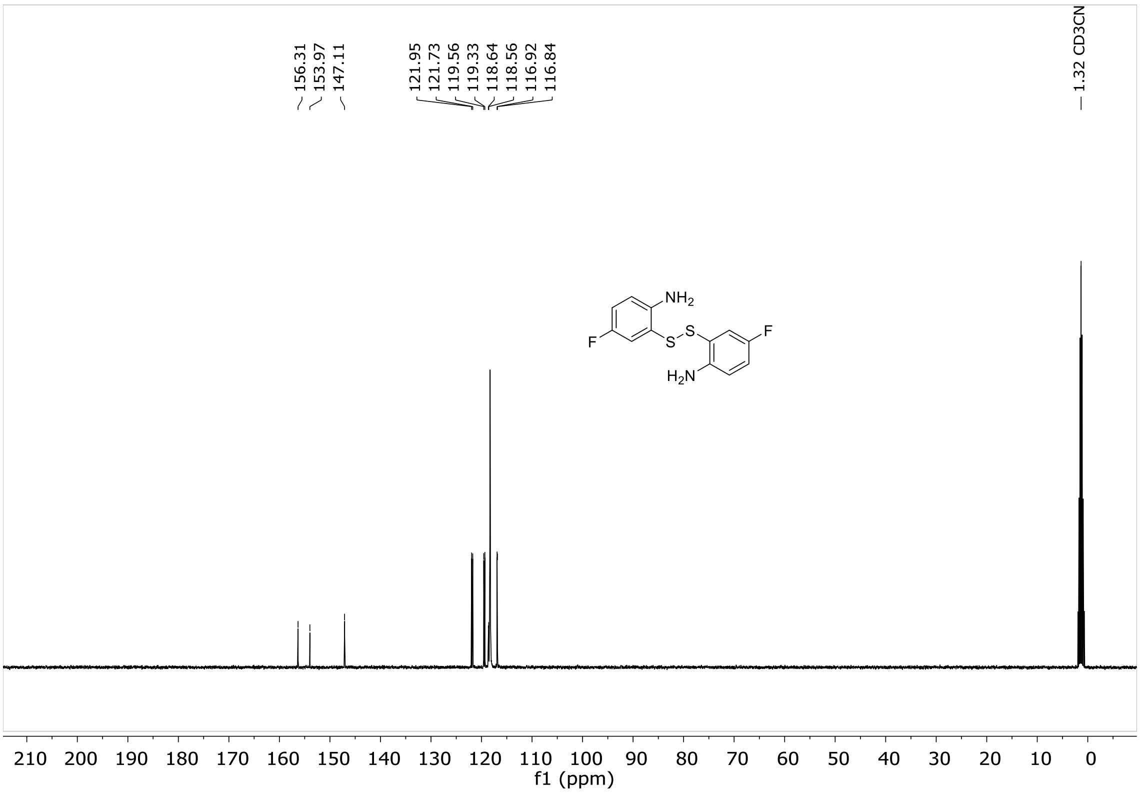


**Figure S082**: $^{13}C\{^{1}H\}$ NMR spectrum of 2,2'-disulfanediylbis(4-fluoroaniline) (100 MHz, $CD_3CN$, 298 K).

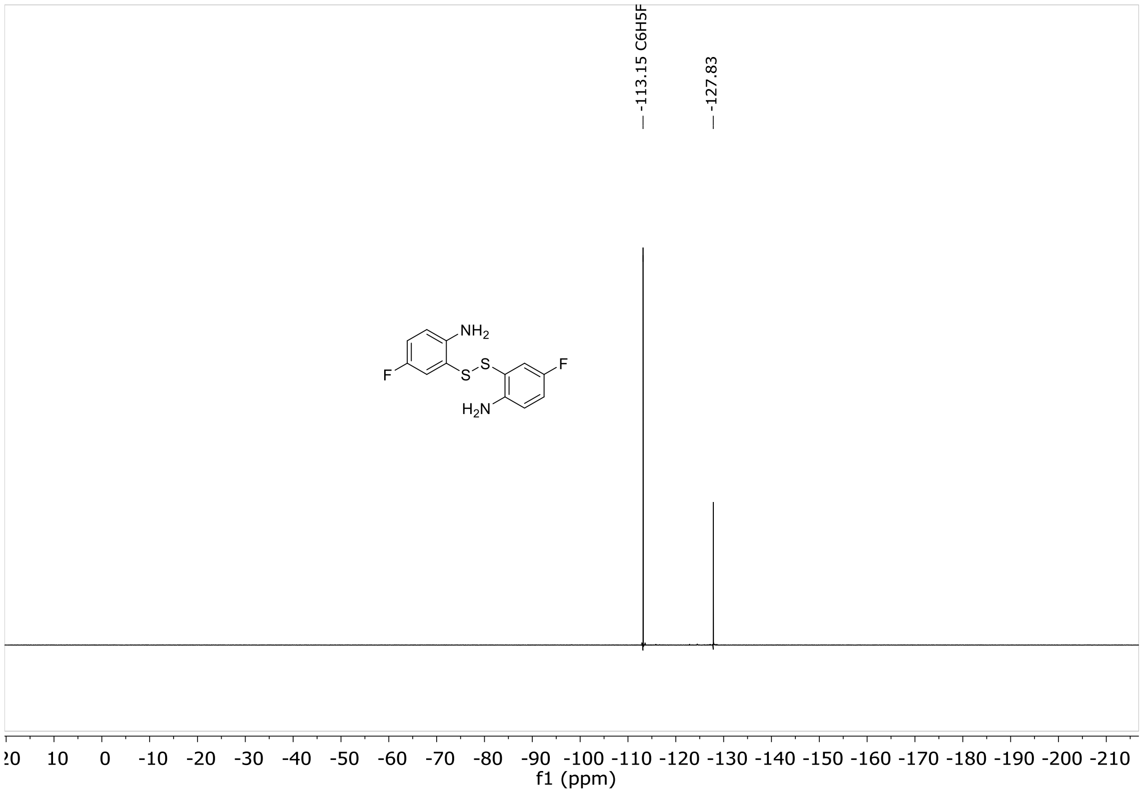

**Figure S083**: $^{19}F\{^{1}H\}$ NMR spectrum of 2,2'-disulfanediylbis(4-fluoroaniline) (376 MHz, $CD_3CN$, 298 K, referenced to fluorobenzene).

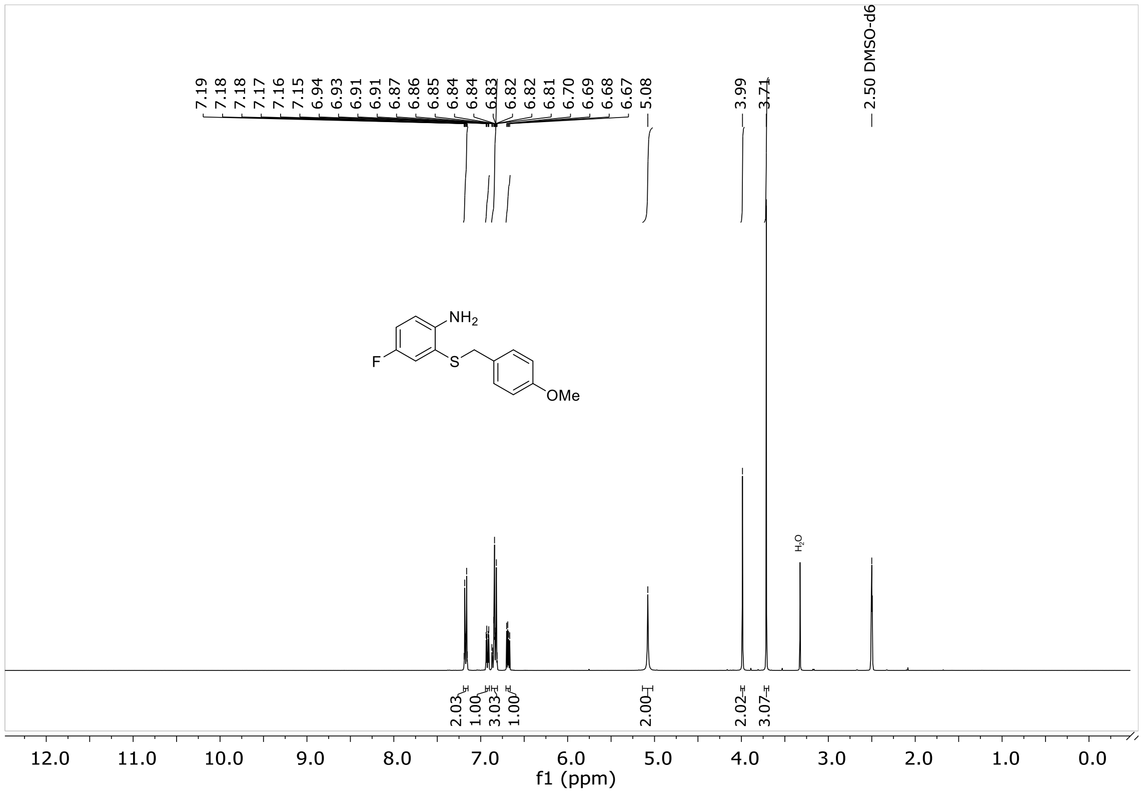

**Figure S084**: $^{1}H$ NMR spectrum of 4-fluoro-2-((4-methoxybenzyl)thio)aniline (**2s**) (400 MHz, DMSO-$d_6$, 298 K).

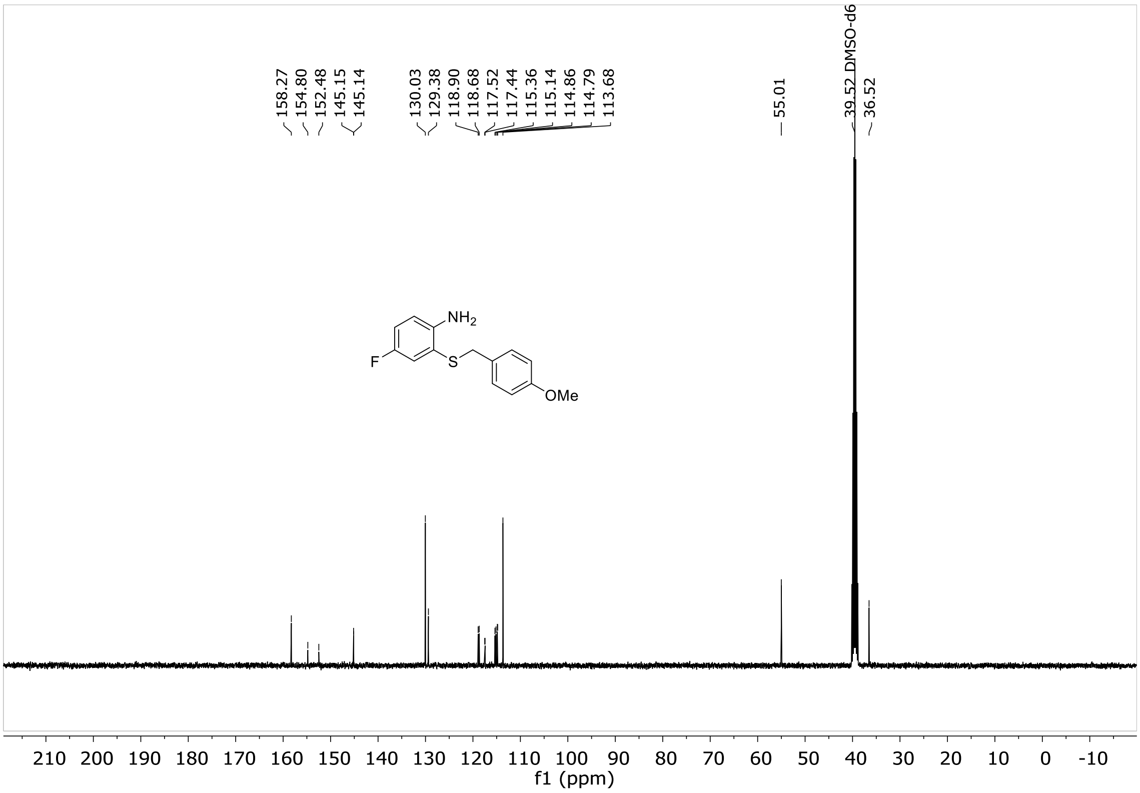


**Figure S085**: $^{13}C\{^1H\}$ NMR spectrum of 4-fluoro-2-((4-methoxybenzyl)thio)aniline (**2s**) (100 MHz, DMSO-$d_6$, 298 K).

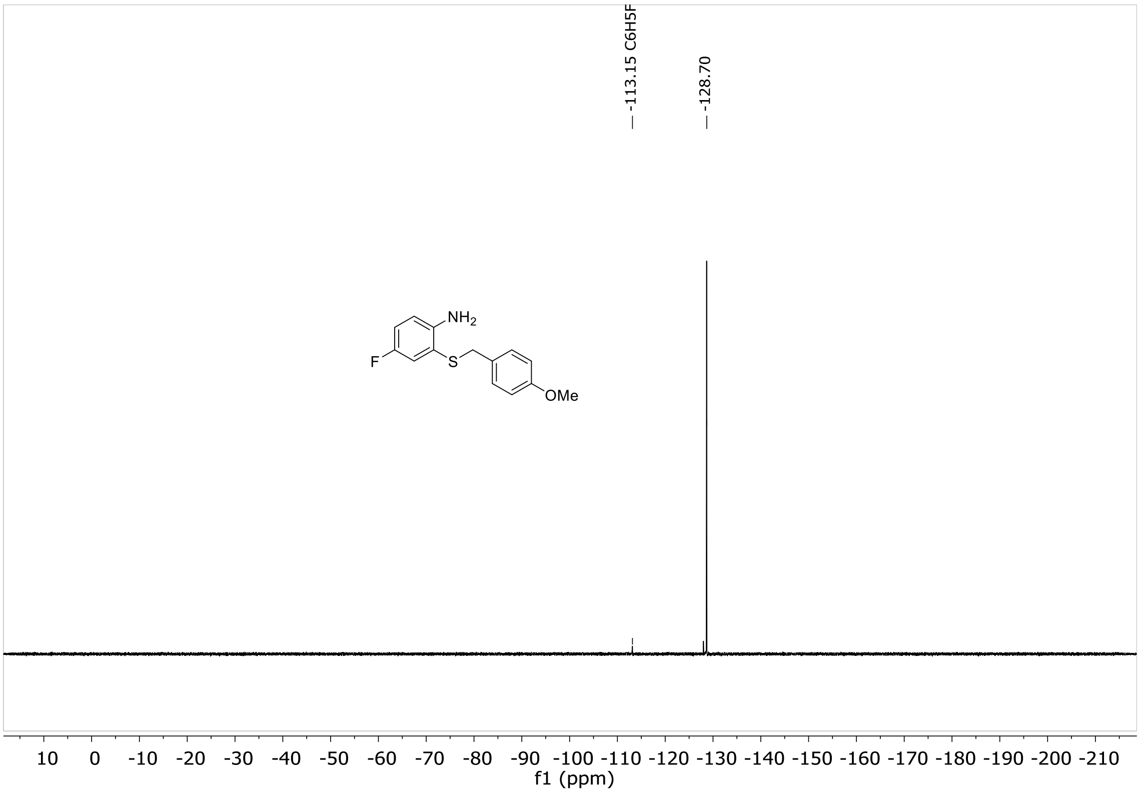


**Figure S086**: $^{19}F\{^1H\}$ NMR spectrum of 4-fluoro-2-((4-methoxybenzyl)thio)aniline (**2s**) (376 MHz, DMSO-$d_6$, 298 K, referenced to fluorobenzene).

## 1.4 NMR Spectra of Substituted 4-(2-((4-Methoxybenzyl)thio)phenyl)-4H-1,2,4-triazoles (3b-3s)

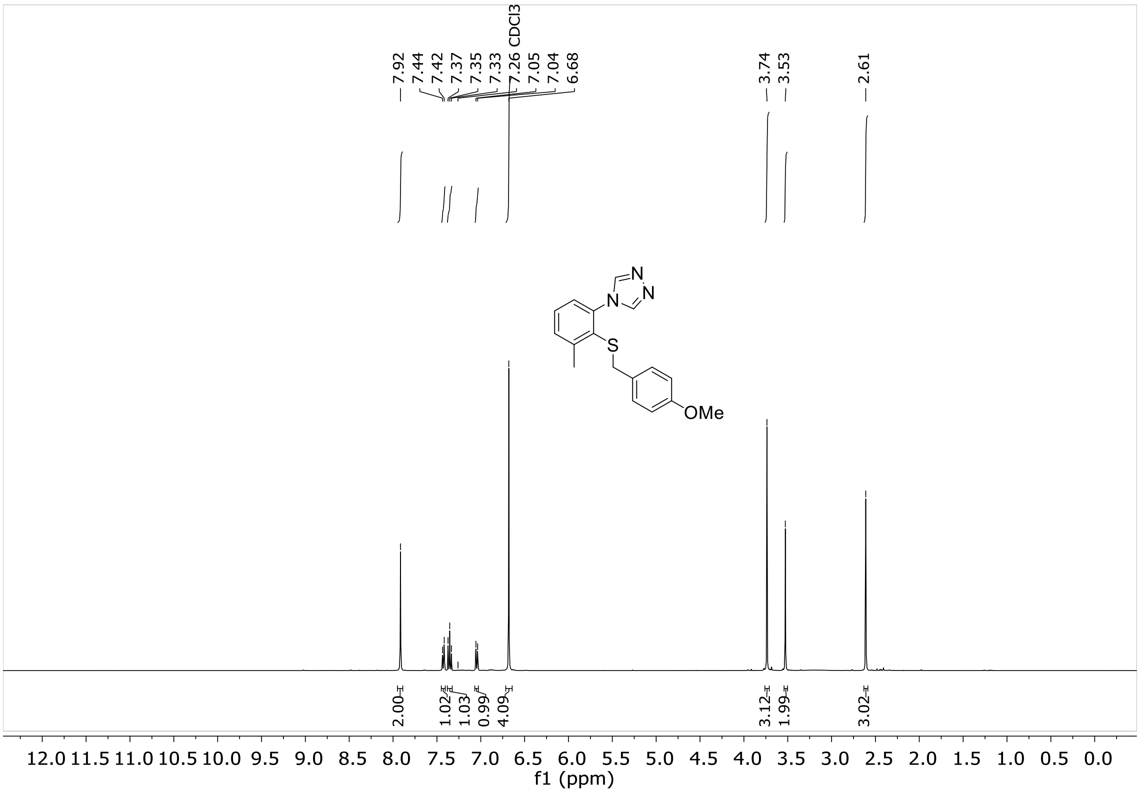


**Figure S087**: $^{1}$H NMR spectrum of 4-(2-((4-methoxybenzyl)thio)-3-methylphenyl)-4*H*-1,2,4-triazole (**3b**) (400 MHz, $CDCl_3$, 298 K).

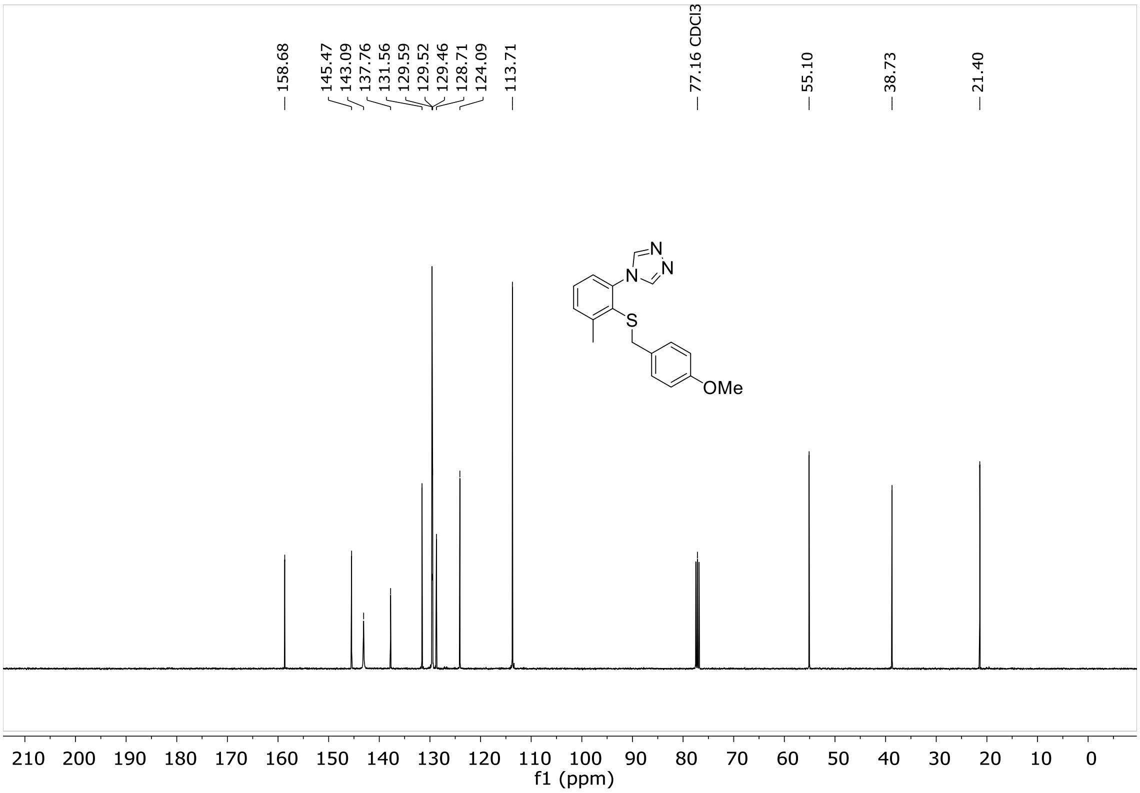


**Figure S088**: $^{13}$C{$^{1}$H} NMR spectrum of 4-(2-((4-methoxybenzyl)thio)-3-methylphenyl)-4*H*-1,2,4-triazole (**3b**) (100 MHz, $CDCl_3$, 298 K).

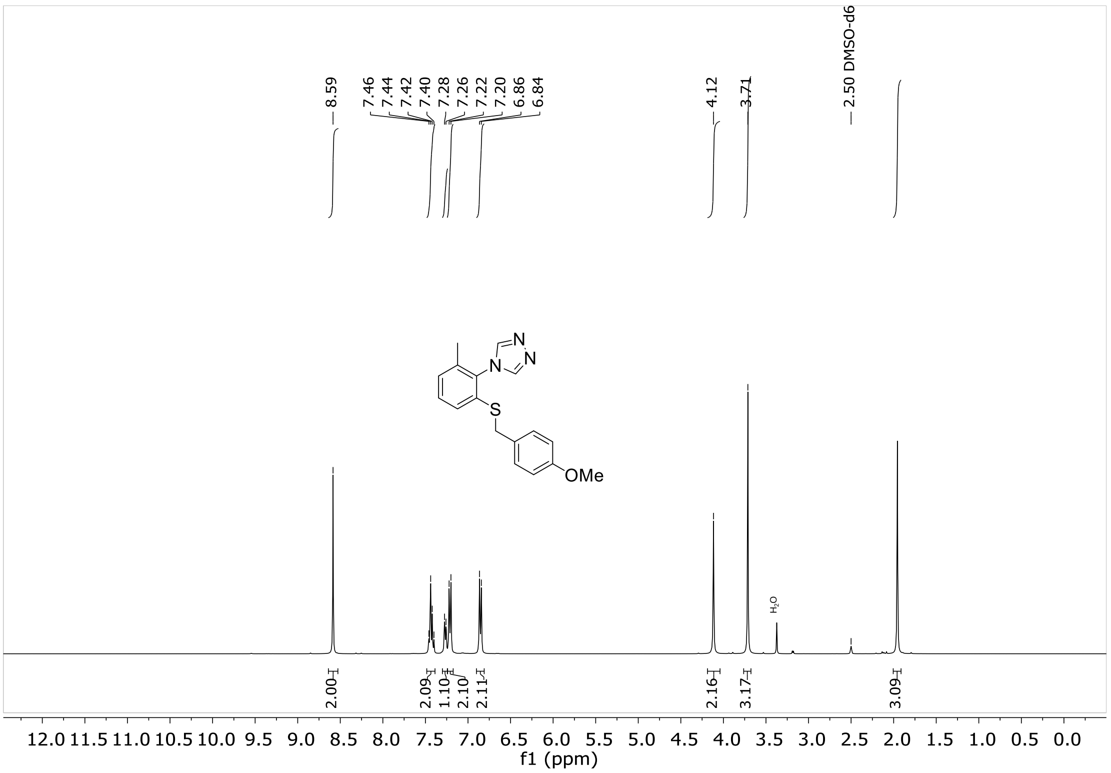


**Figure S089**: $^{1}$H NMR spectrum of 4-(2-((4-methoxybenzyl)thio)-6-methylphenyl)-4*H*-1,2,4-triazole (**3c**) (400 MHz, DMSO-$d_6$, 298 K).

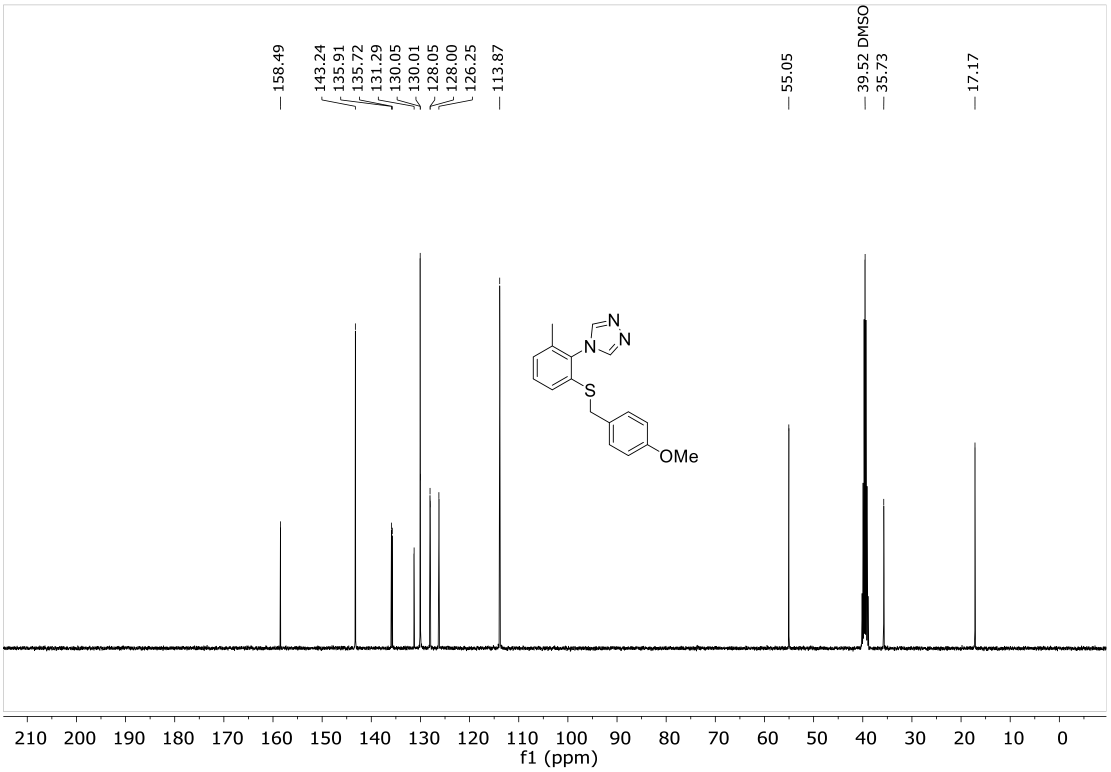


**Figure S090**: $^{13}$C{$^{1}$H} NMR spectrum of 4-(2-((4-methoxybenzyl)thio)-6-methylphenyl)-4*H*-1,2,4-triazole (**3c**) (400 MHz, DMSO-$d_6$, 298 K).

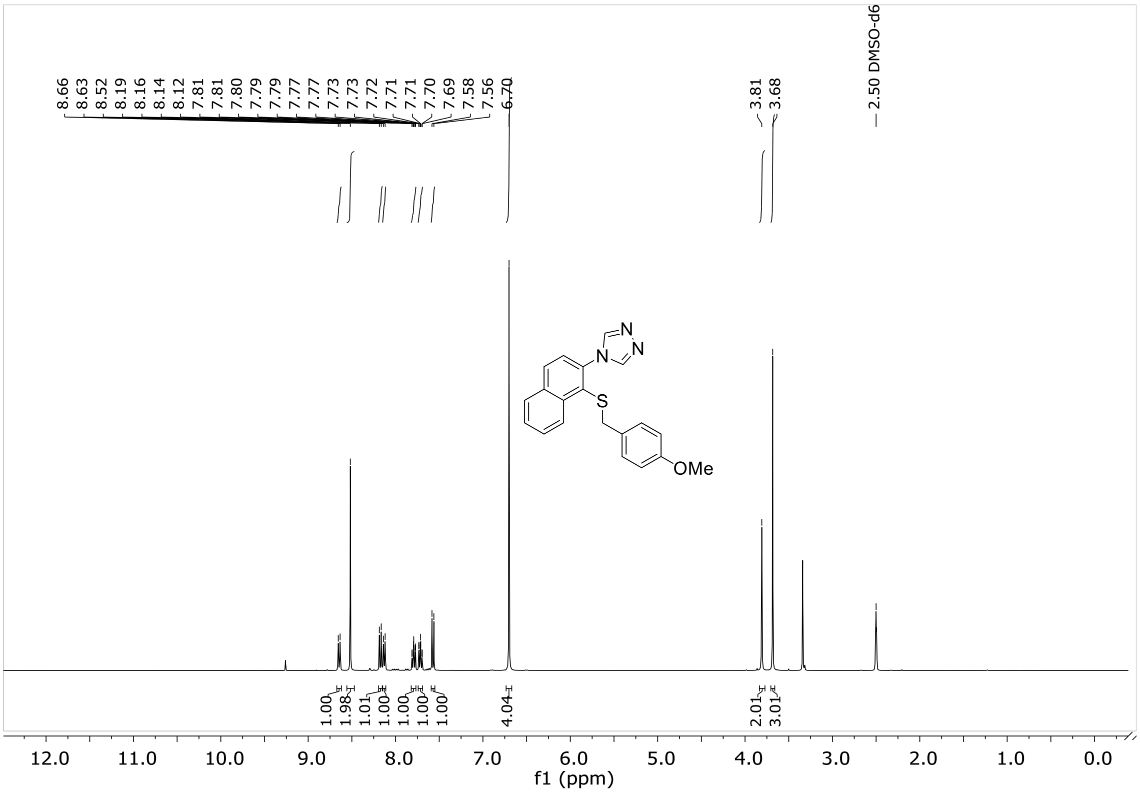


**Figure S091**: $^{1}H$ NMR spectrum of 4-(1-((4-methoxybenzyl)thio)naphthalen-2-yl)-4*H*-1,2,4-triazole (**3d**) (400 MHz, DMSO-$d_6$, 298 K).

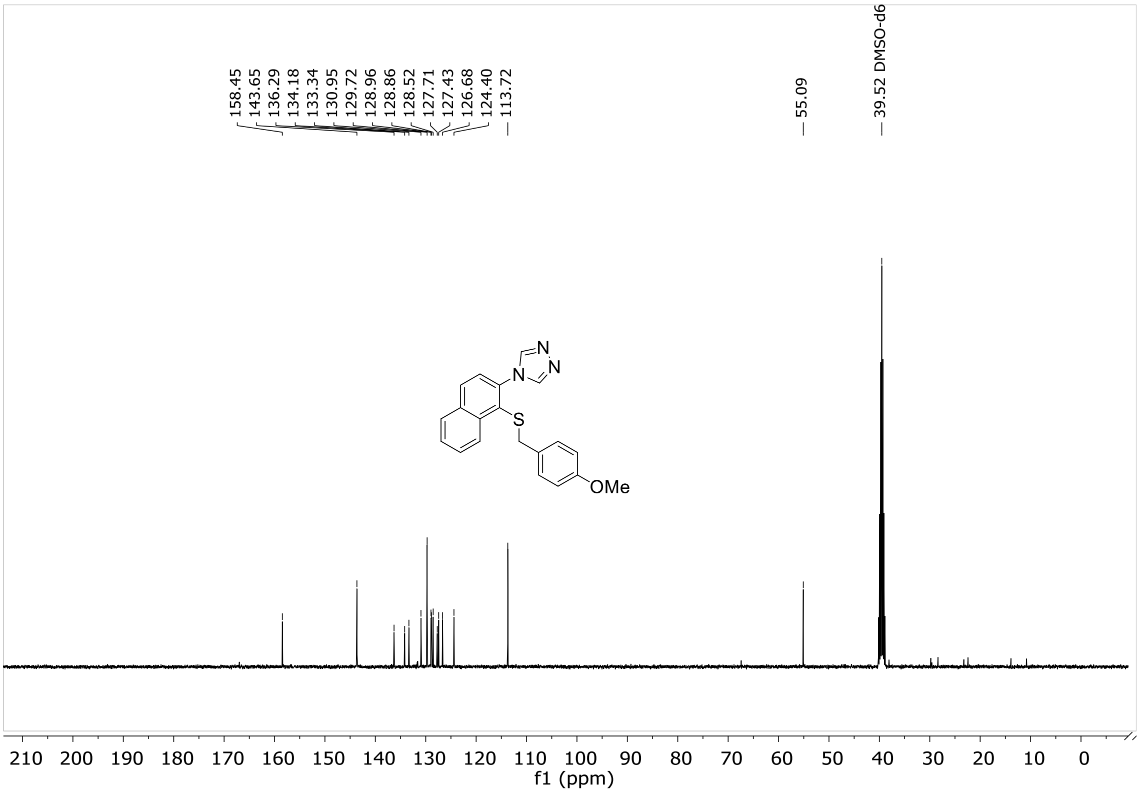


**Figure S092**: $^{13}C\{^{1}H\}$ NMR spectrum of 4-(1-((4-methoxybenzyl)thio)naphthalen-2-yl)-4*H*-1,2,4-triazole (**3d**) (400 MHz, DMSO-$d_6$, 298 K).

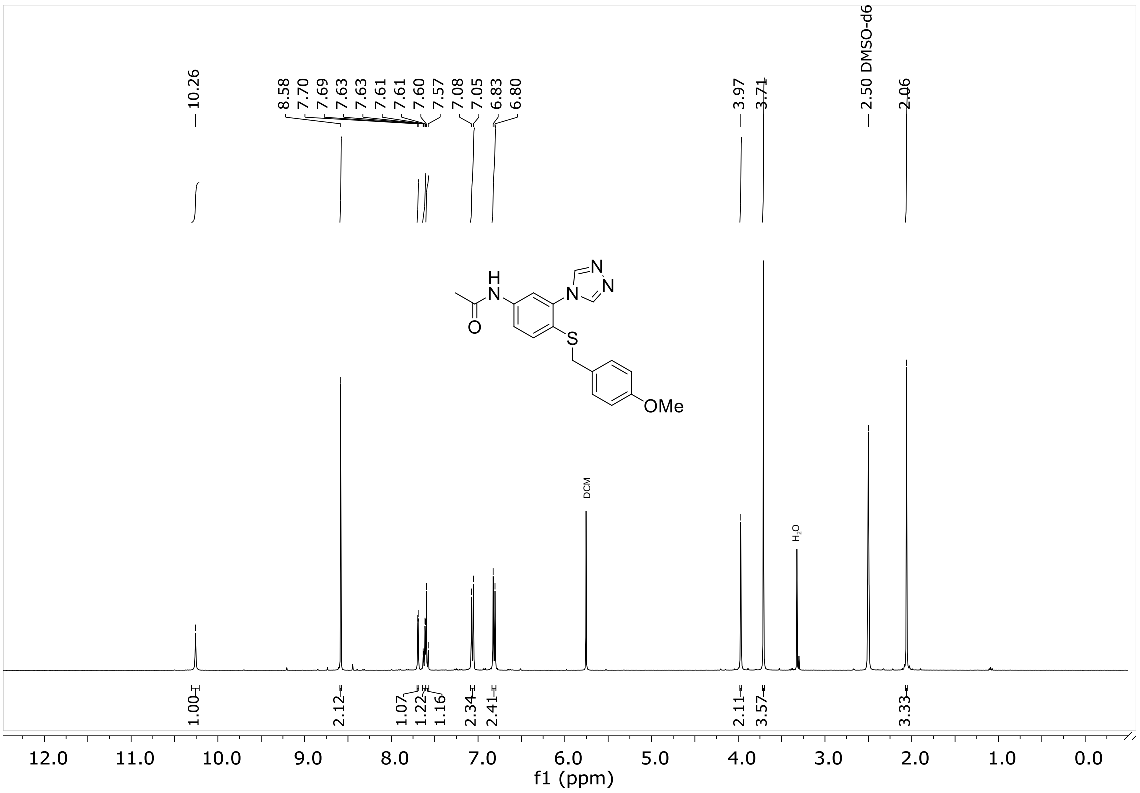

**Figure S093**: $^{1}$H NMR spectrum of *N*-(4-((4-methoxybenzyl)thio)-3-(4*H*-1,2,4-triazol-4-yl)phenyl)acetamide (**3f**) (400 MHz, DMSO-$d_6$, 298 K).

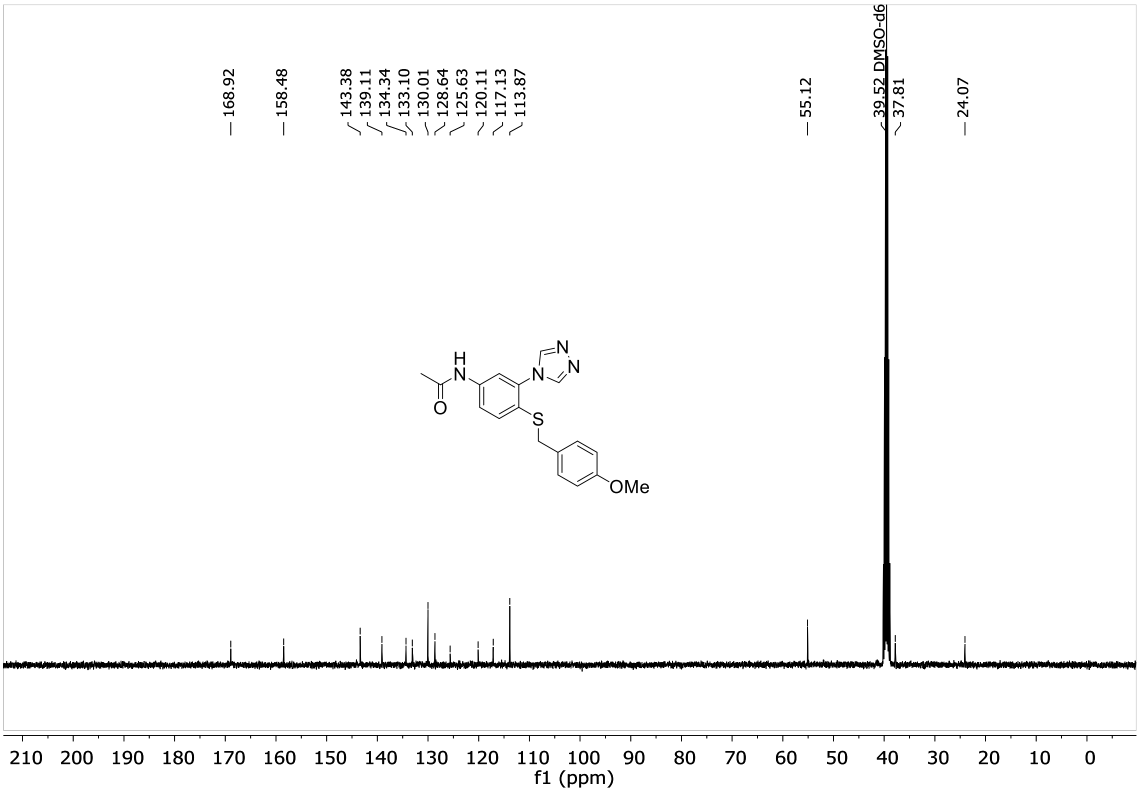

**Figure S094**: $^{13}$C{$^{1}$H} NMR spectrum of *N*-(4-((4-methoxybenzyl)thio)-3-(4*H*-1,2,4-triazol-4-yl)phenyl)acetamide (**3f**) (100 MHz, DMSO-$d_6$, 298 K).

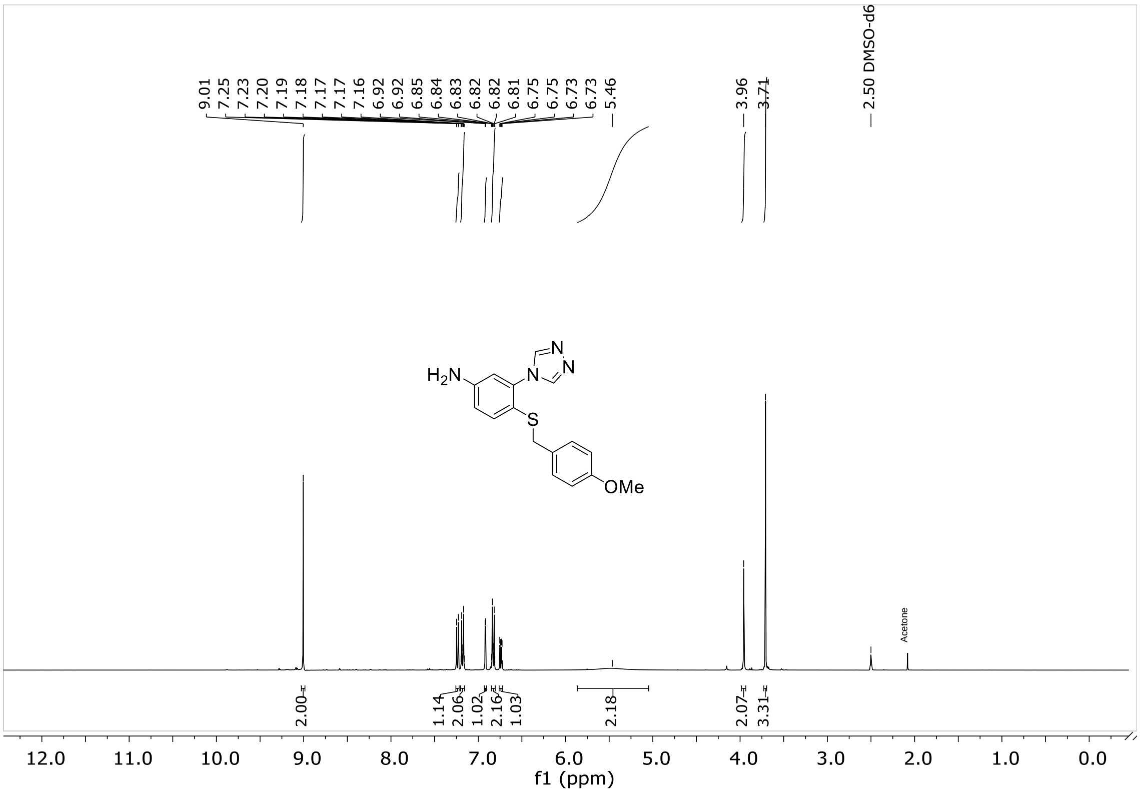

**Figure S095**: $^{1}$H NMR spectrum of 4-((4-methoxybenzyl)thio)-3-(4*H*-1,2,4-triazol-4-yl)aniline (**3e**) (400 MHz, DMSO-$d_6$, 298 K).

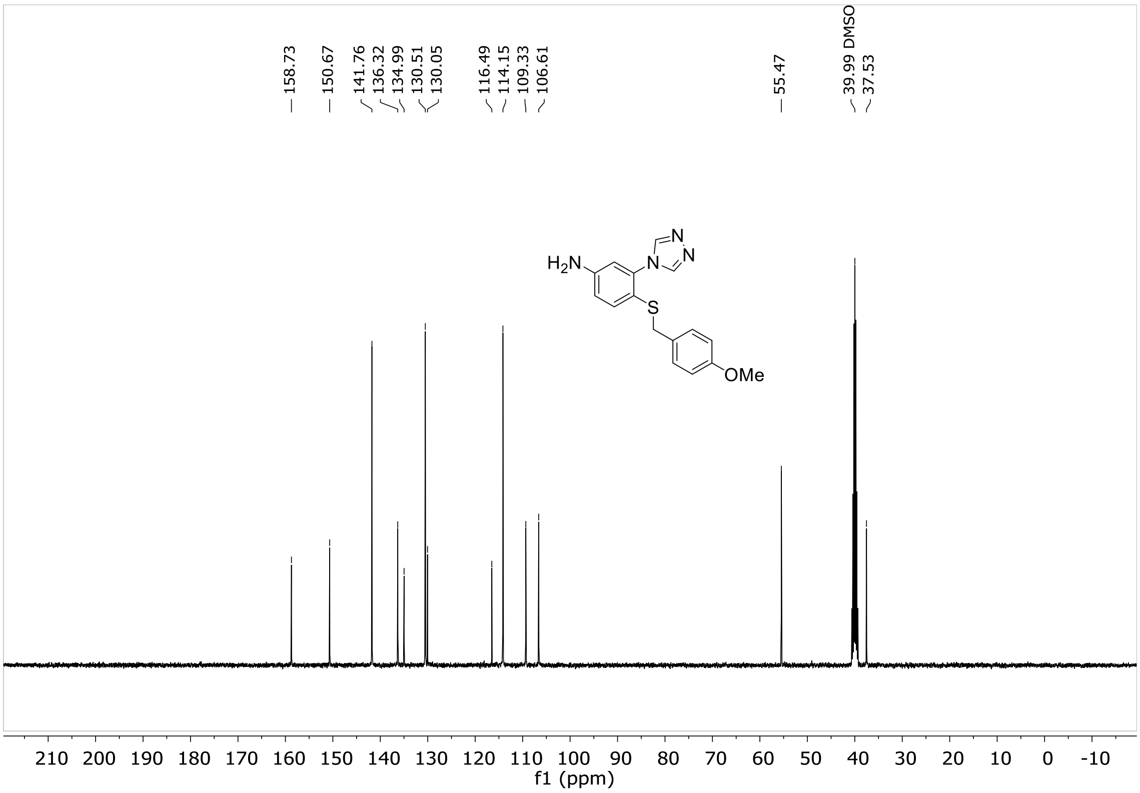

**Figure S096**: $^{13}$C{$^{1}$H} NMR spectrum of 4-((4-methoxybenzyl)thio)-3-(4*H*-1,2,4-triazol-4-yl)aniline (**3e**) (100 MHz, DMSO-$d_6$, 298 K).

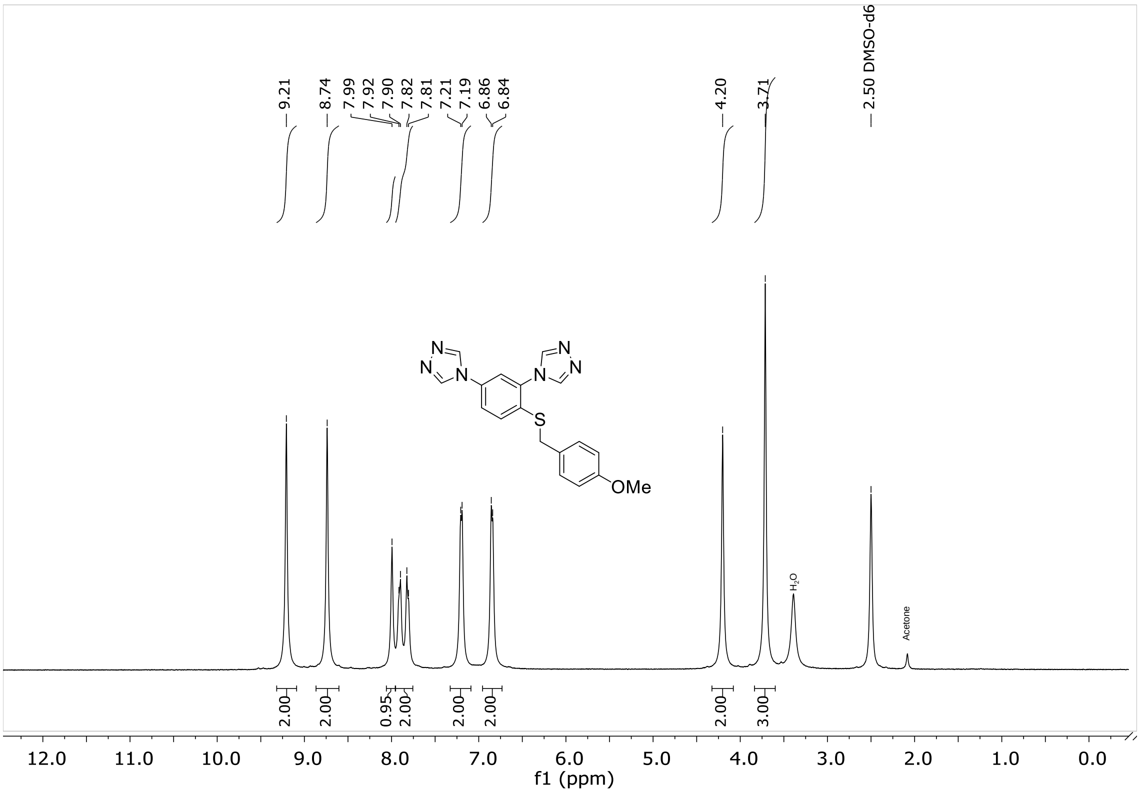


**Figure S097**: $^{1}H$ NMR 4,4'-(4-((4-methoxybenzyl)thio)-1,3-phenylene)bis(4*H*-1,2,4-triazole) (**3e**) (400 MHz, DMSO-$d_6$, 298 K).

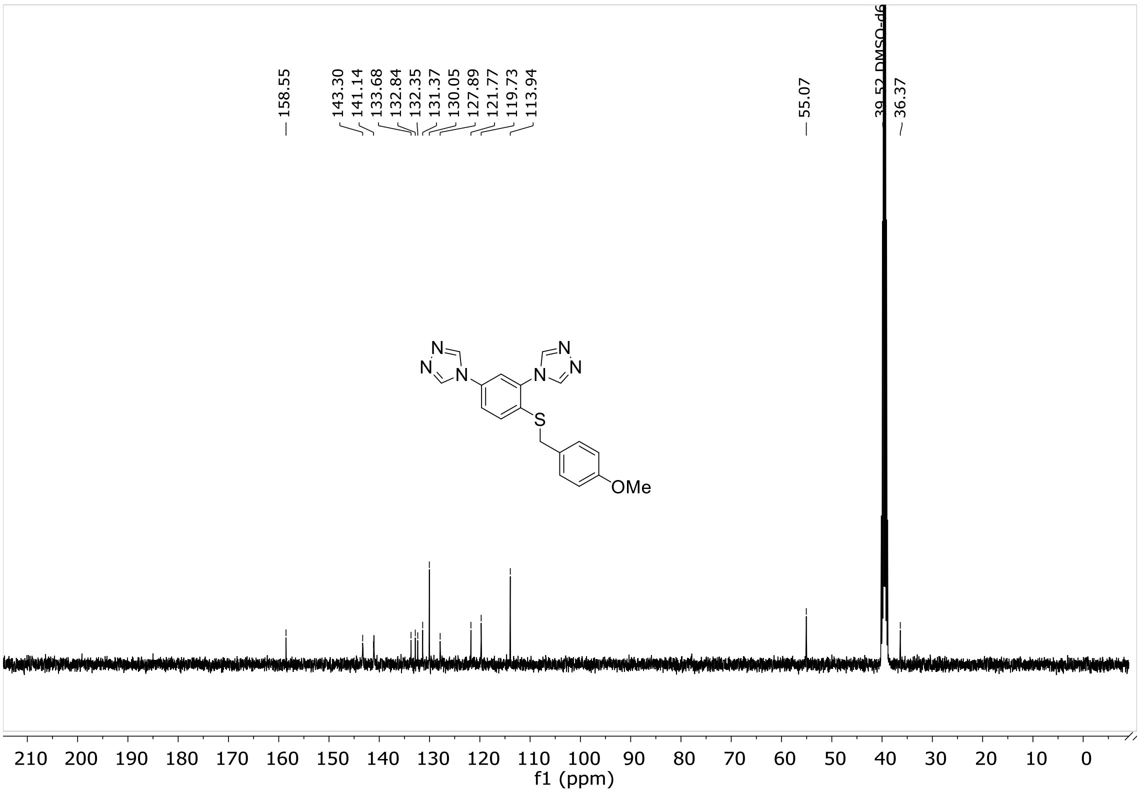


**Figure S098**: $^{13}C\{^{1}H\}$ NMR 4,4'-(4-((4-methoxybenzyl)thio)-1,3-phenylene)bis(4*H*-1,2,4-triazole) (**3e**) (100 MHz, DMSO-$d_6$, 298 K).

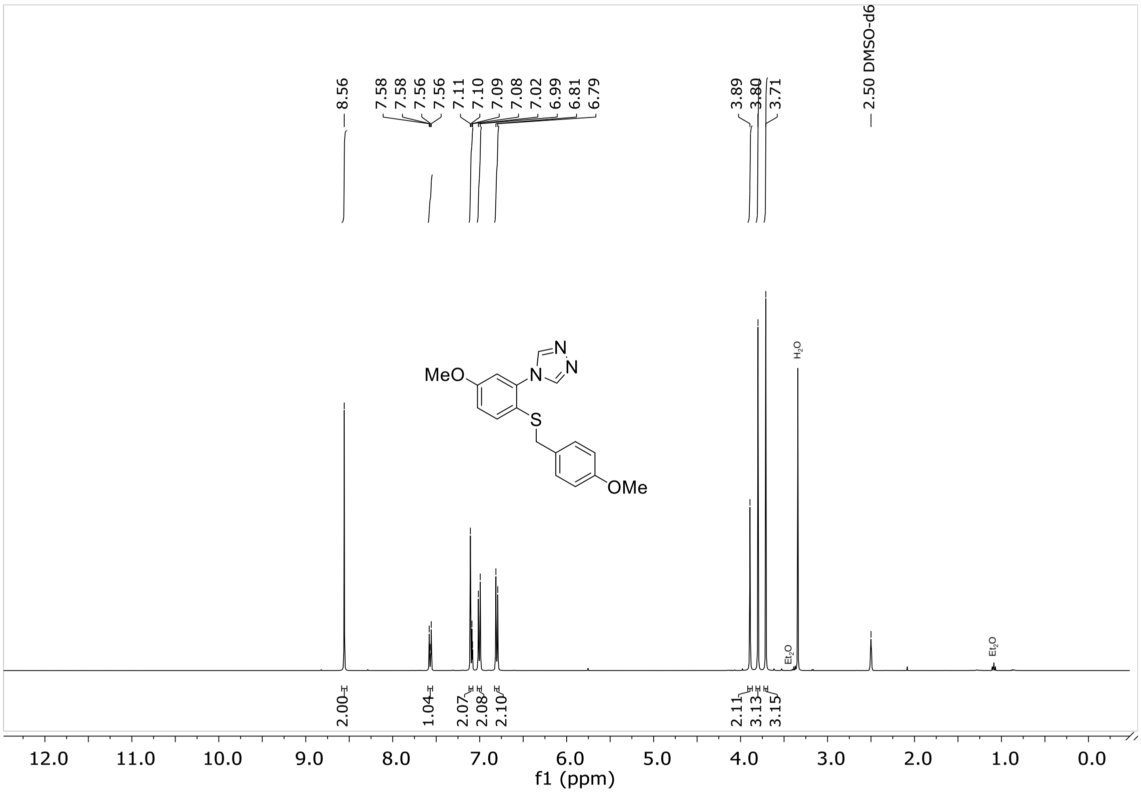


**Figure S099**: $^{1}$H NMR 4-(5-methoxy-2-((4-methoxybenzyl)thio)phenyl)-4*H*-1,2,4-triazole (**3g**) (400 MHz, DMSO-$d_6$, 298 K).

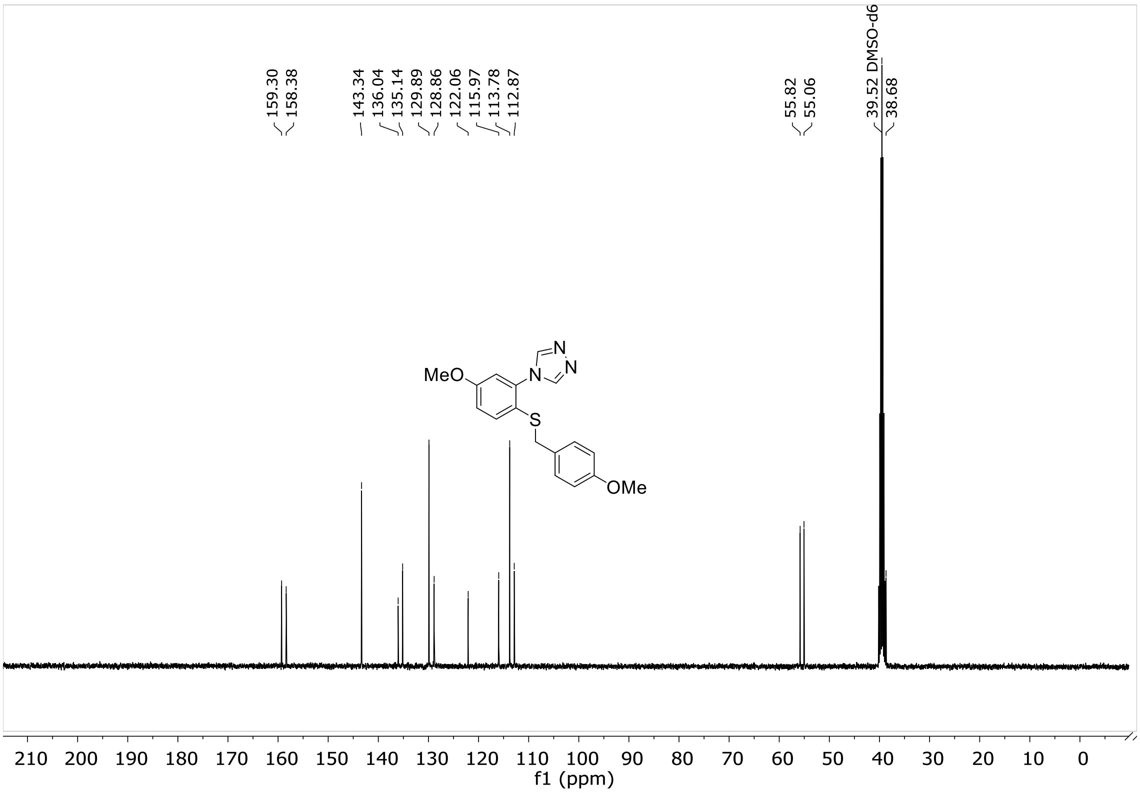


**Figure S100**: $^{13}$C{$^{1}$H} NMR 4-(5-methoxy-2-((4-methoxybenzyl)thio)phenyl)-4*H*-1,2,4-triazole (**3g**) (100 MHz, DMSO-$d_6$, 298 K).

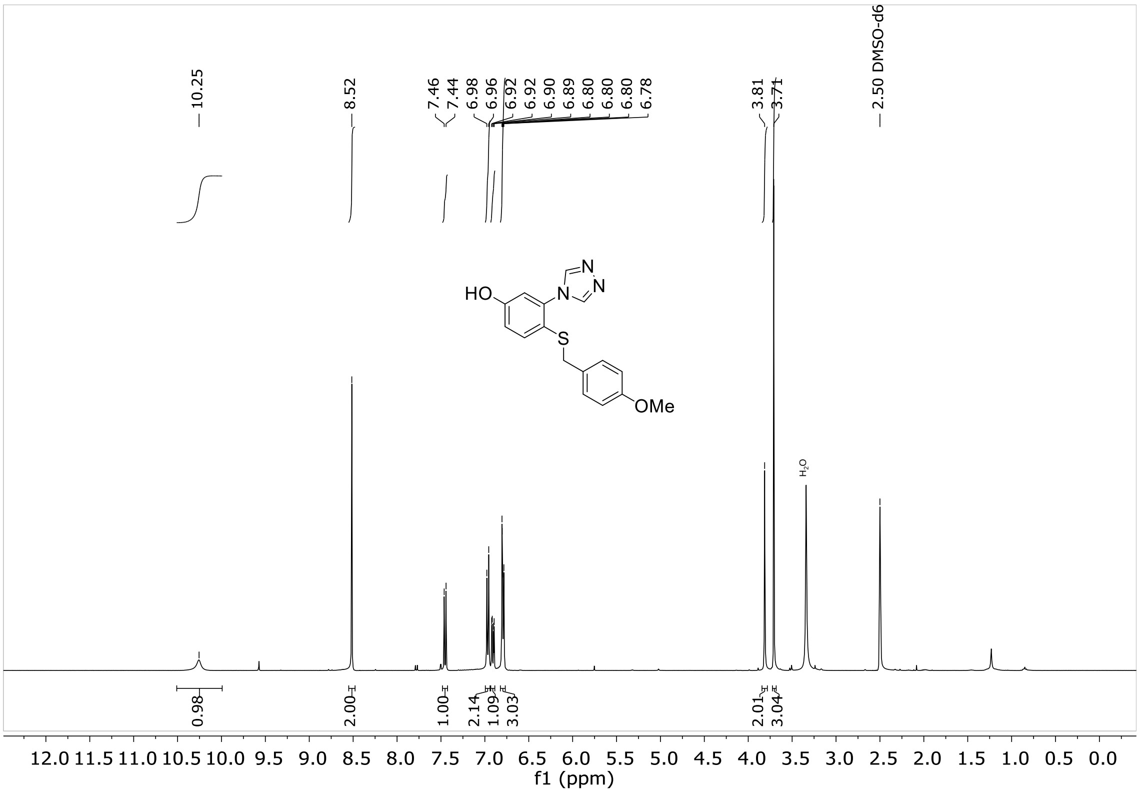


**Figure S101**: $^{1}$H NMR spectrum of 4-((4-methoxybenzyl)thio)-3-(4*H*-1,2,4-triazol-4-yl)phenol (3h) (400 MHz, DMSO-$d_6$, 298 K).

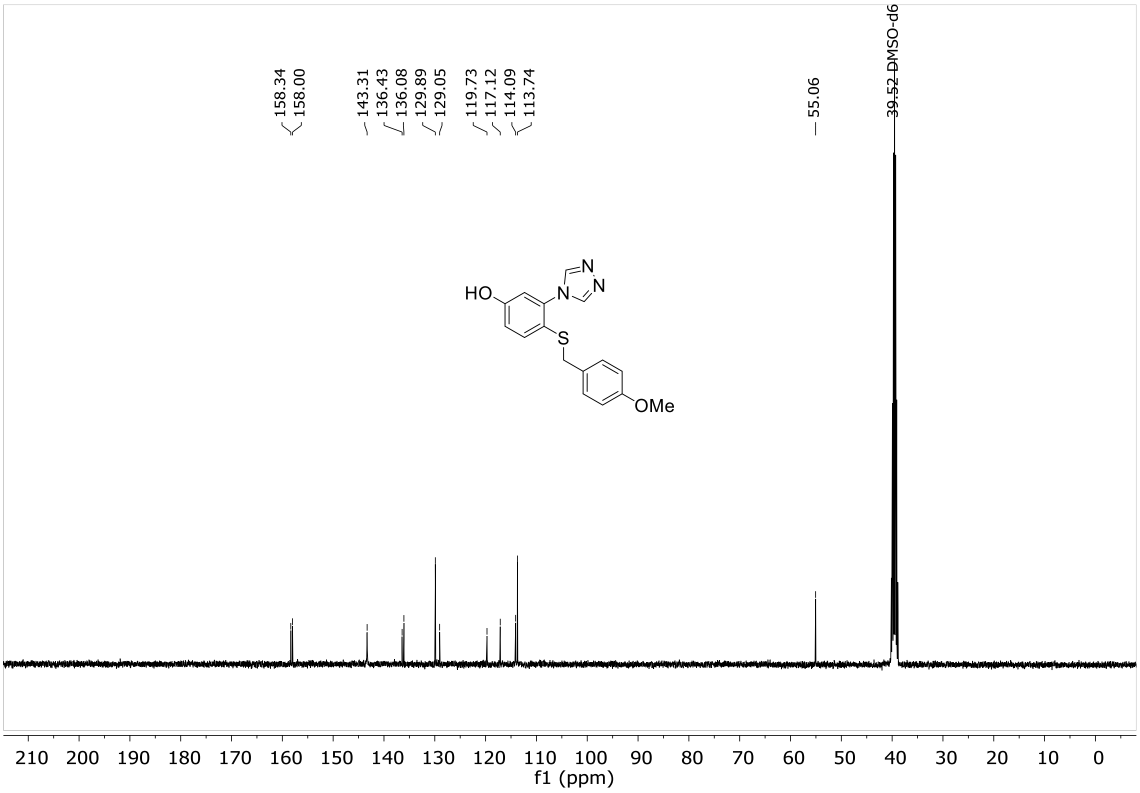


**Figure S102**: $^{13}$C{$^{1}$H} NMR spectrum of 4-((4-methoxybenzyl)thio)-3-(4*H*-1,2,4-triazol-4-yl)phenol (**3h)** (100 MHz, DMSO-$d_6$, 298 K).

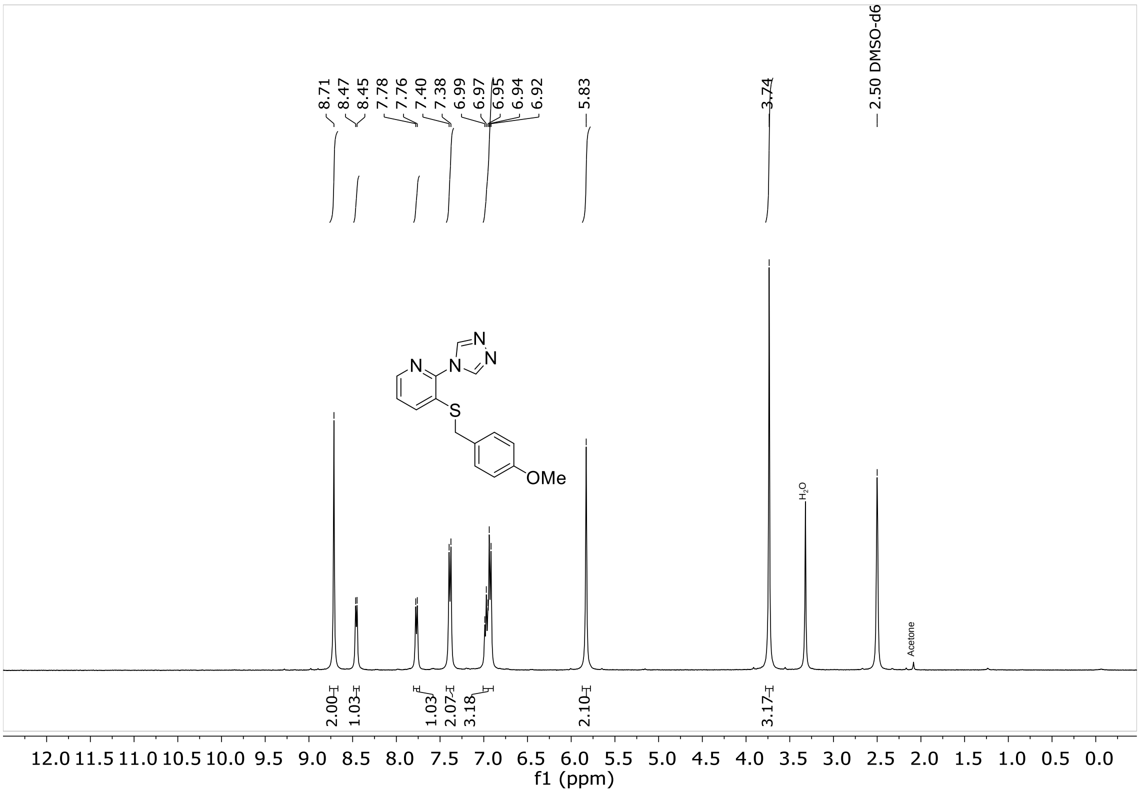


**Figure S103**: $^{1}$H NMR 3-((4-methoxybenzyl)thio)-2-(4*H*-1,2,4-triazol-4-yl)pyridine (**3i**) (400 MHz, DMSO-$d_6$, 298 K).

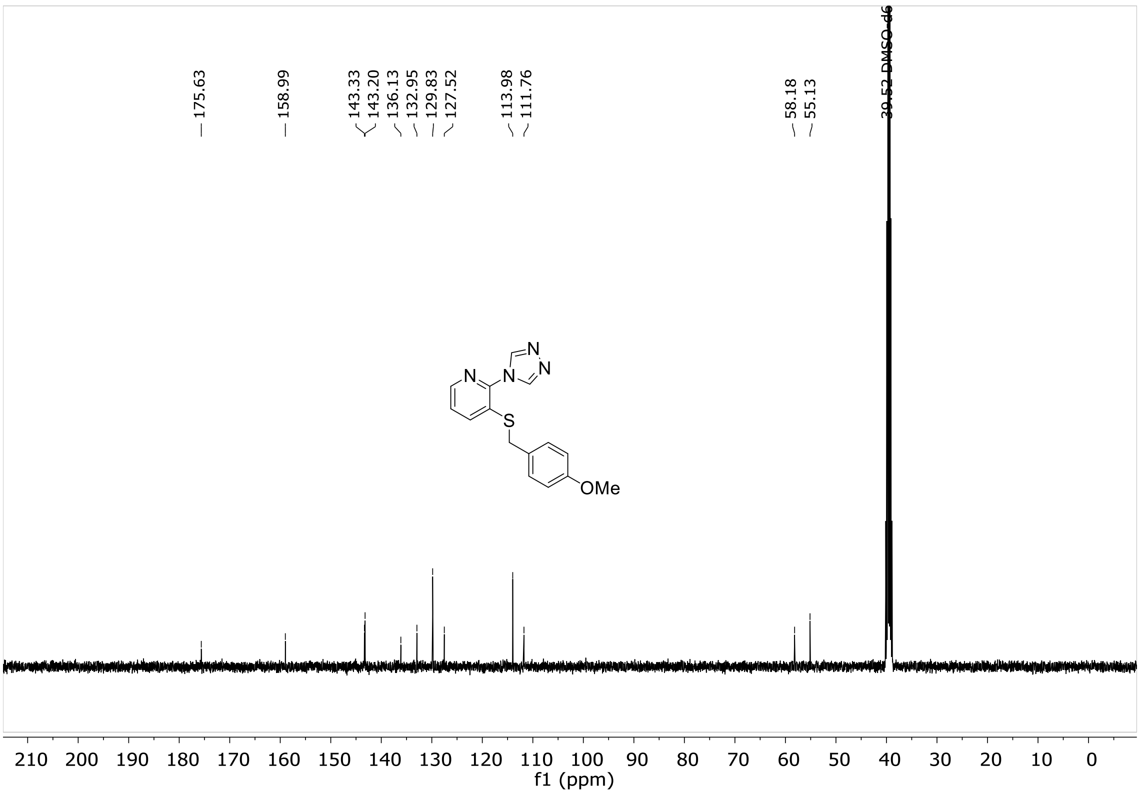


**Figure S104**: $^{13}$C{$^{1}$H} NMR 3-((4-methoxybenzyl)thio)-2-(4*H*-1,2,4-triazol-4-yl)pyridine (**3i**) (100 MHz, DMSO-$d_6$, 298 K).

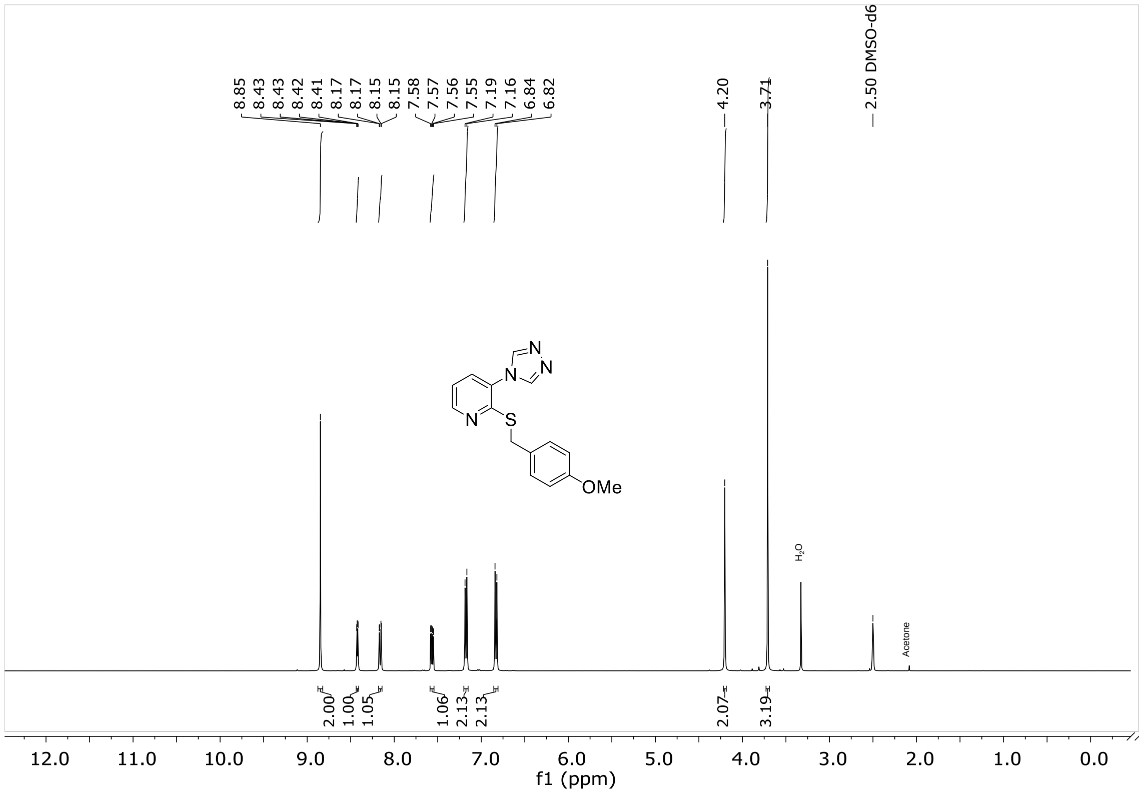

**Figure S105**: $^{1}$HNMR 2-((4-methoxybenzyl)thio)-3-(4*H*-1,2,4-triazol-4-yl)pyridine (**3j**) (400 MHz, DMSO-$d_6$, 298 K).

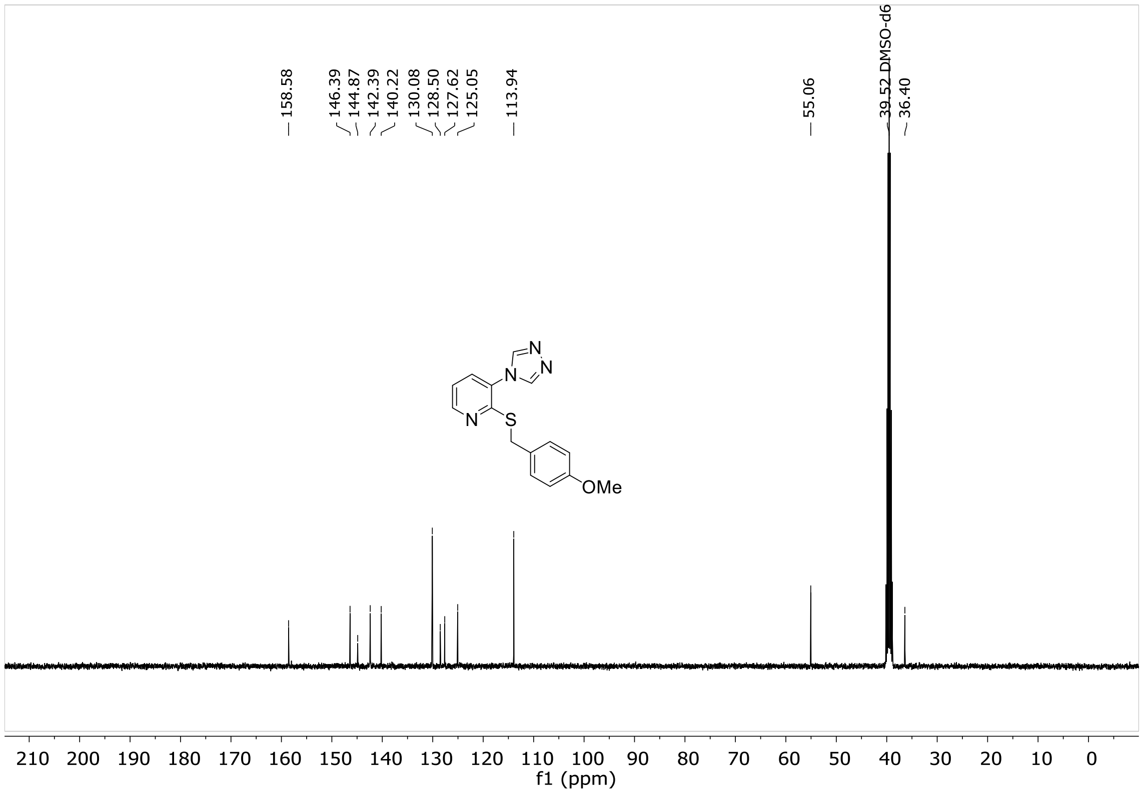

**Figure S106**: $^{13}C\{^1H\}$ NMR 2-((4-methoxybenzyl)thio)-3-(4*H*-1,2,4-triazol-4-yl)pyridine (**3j**) (100 MHz, DMSO-$d_6$, 298 K.

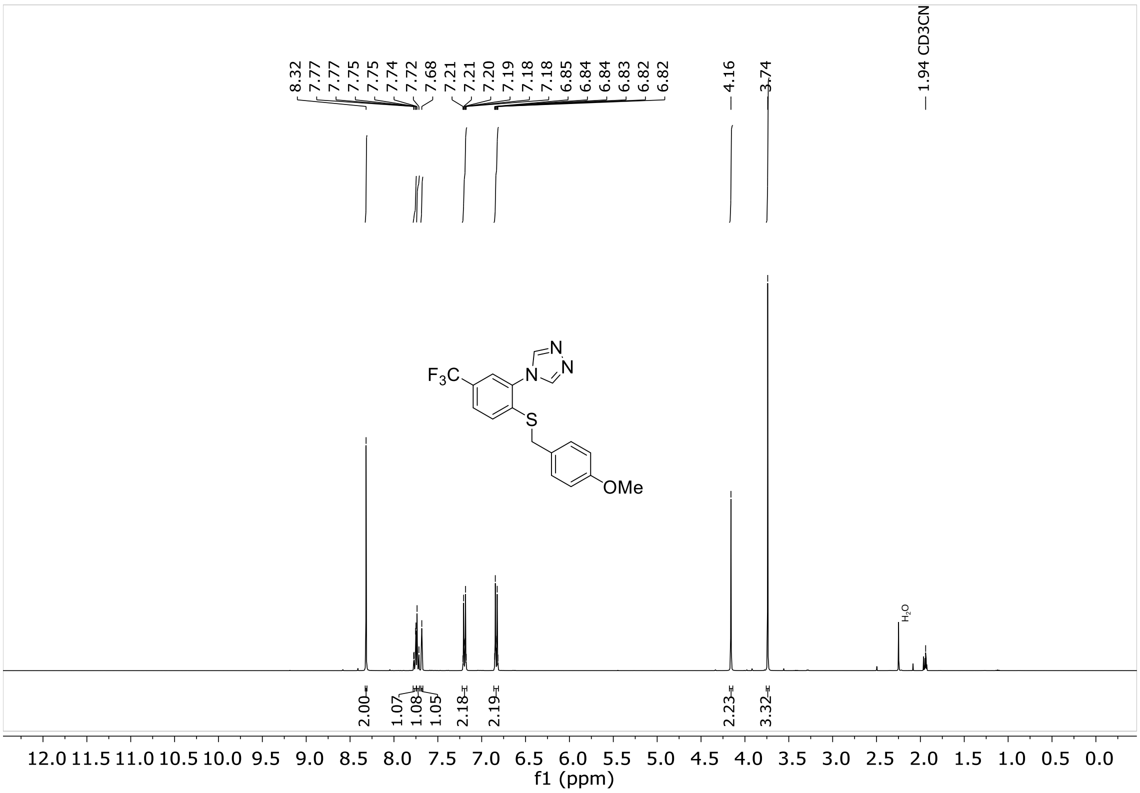

**Figure S107**: $^{1}$H NMR 4-(2-((4-methoxybenzyl)thio)-5-(trifluoromethyl)phenyl)-4*H*-1,2,4-triazole (**3k**) (400 MHz, $CD_3CN$, 298 K).

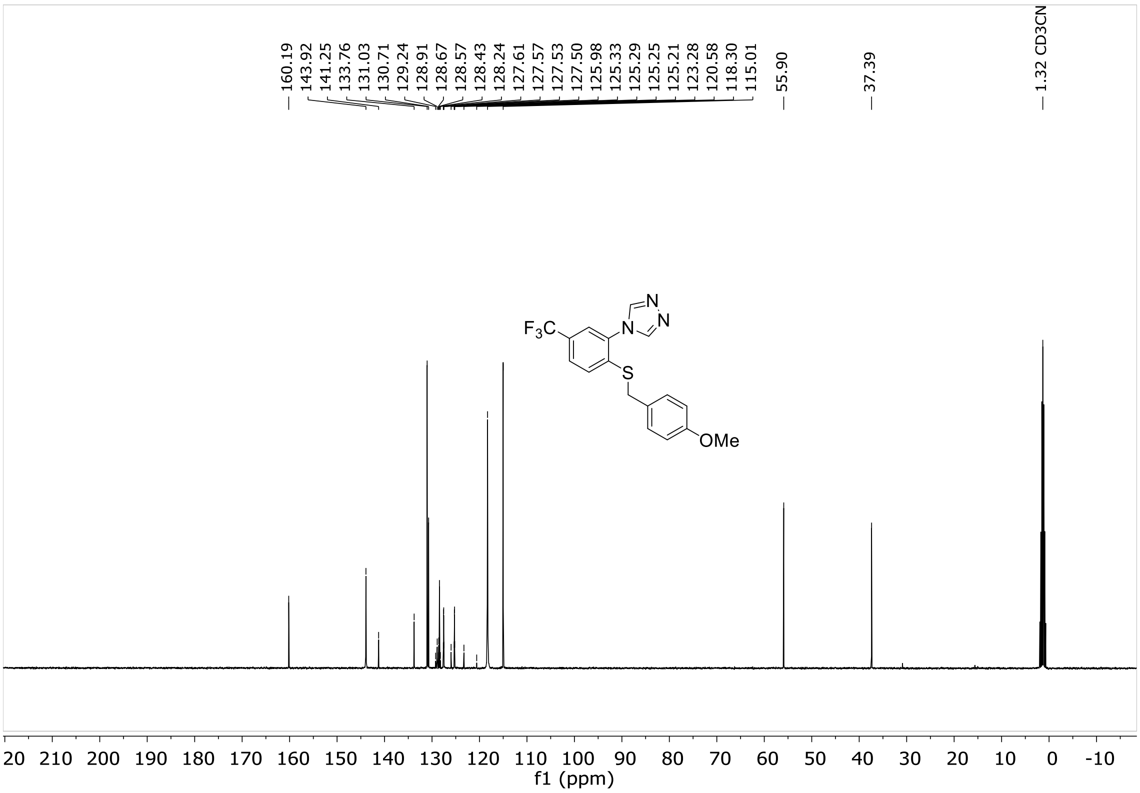

**Figure S108**: $^{13}C\{^1H\}$ NMR 4-(2-((4-methoxybenzyl)thio)-5-(trifluoromethyl)phenyl)-4*H*-1,2,4-triazole (**3k**) (100 MHz, $CD_3CN$, 298 K).

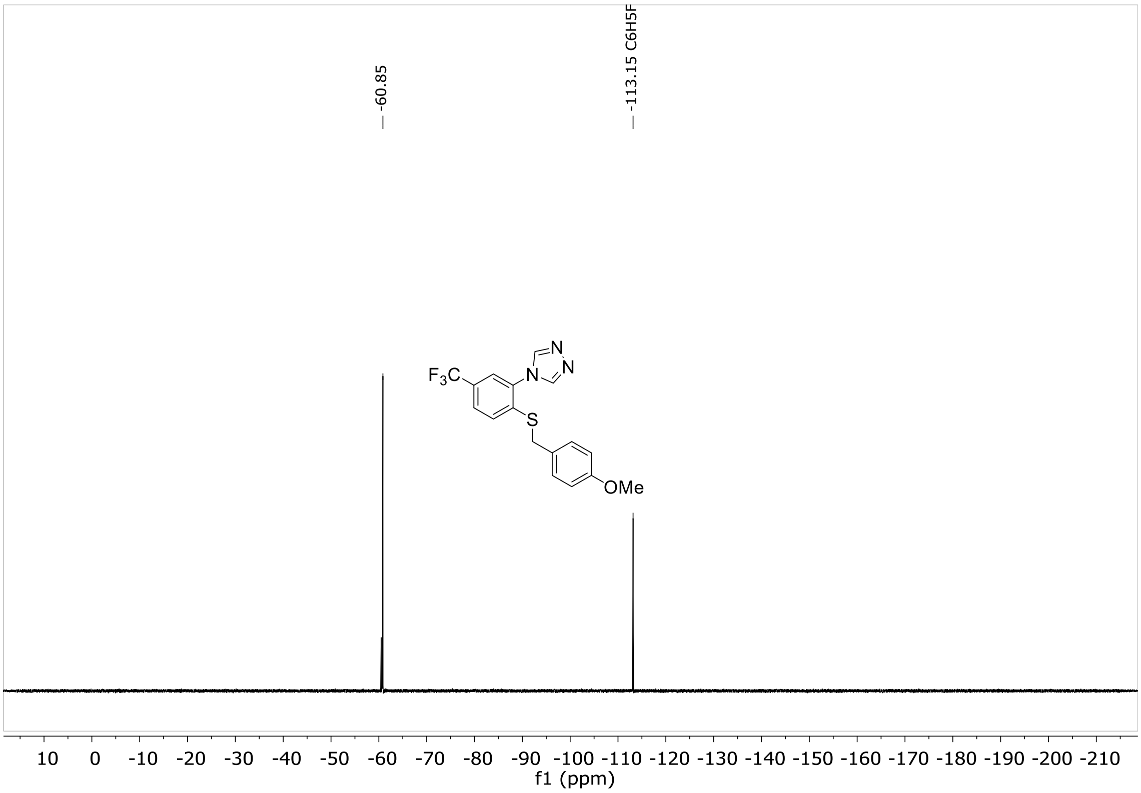


**Figure S109**: $^{19}F\{^{1}H\}$ NMR 4-(2-((4-methoxybenzyl)thio)-5-(trifluoromethyl)phenyl)-4*H*-1,2,4-triazole (**3k**) (376 MHz, $CD_3CN$, 298 K, referenced to fluorobenzene).

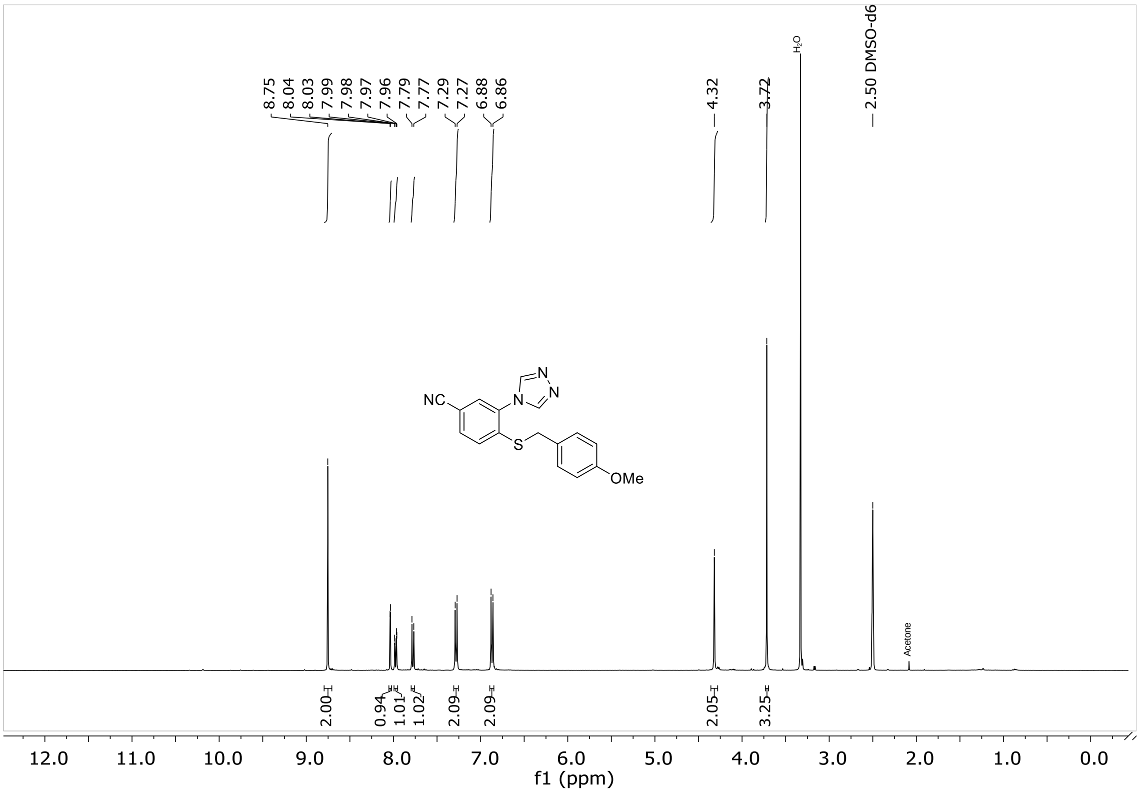


**Figure S110**: $^{1}H$ NMR spectrum of 4-((4-methoxybenzyl)thio)-3-(4*H*-1,2,4-triazol-4-yl)benzonitrile (**3l**) (400 MHz, DMSO-$d_6$, 298 K).

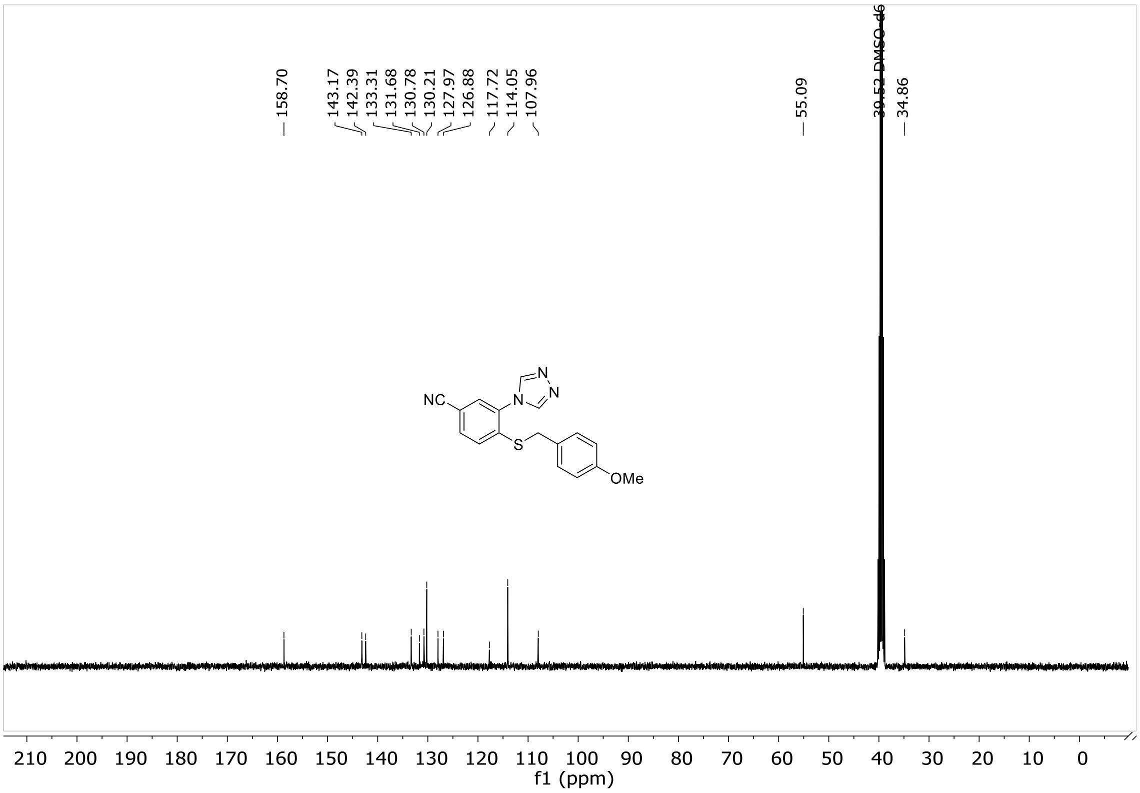


**Figure S111**: $^{13}$C{$^{1}$H} NMR spectrum of 4-((4-methoxybenzyl)thio)-3-(4*H*-1,2,4-triazol-4-yl)benzonitrile (**3l**) (100 MHz, DMSO-$d_6$, 298 K).

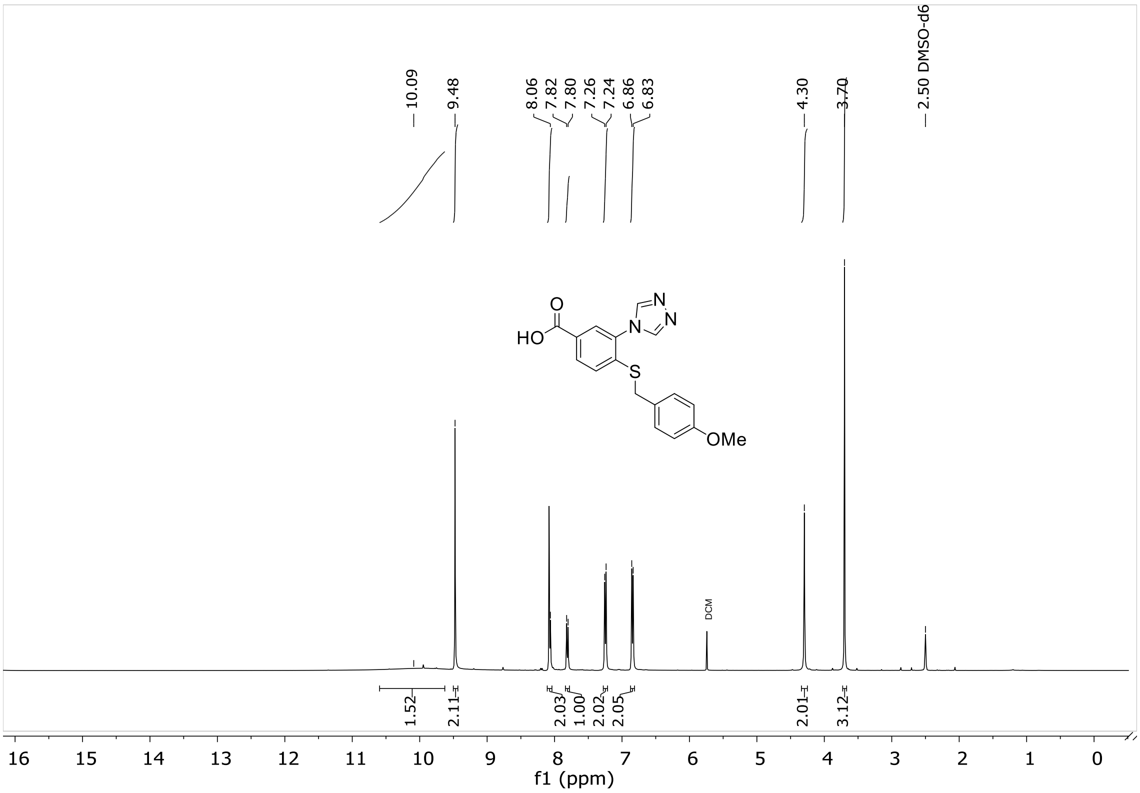


**Figure S112**: $^{1}$H NMR spectrum of 4-((4-methoxybenzyl)thio)-3-(4*H*-1,2,4-triazol-4-yl)benzoic acid (**3m**) (400 MHz, DMSO-$d_6$, 298 K).

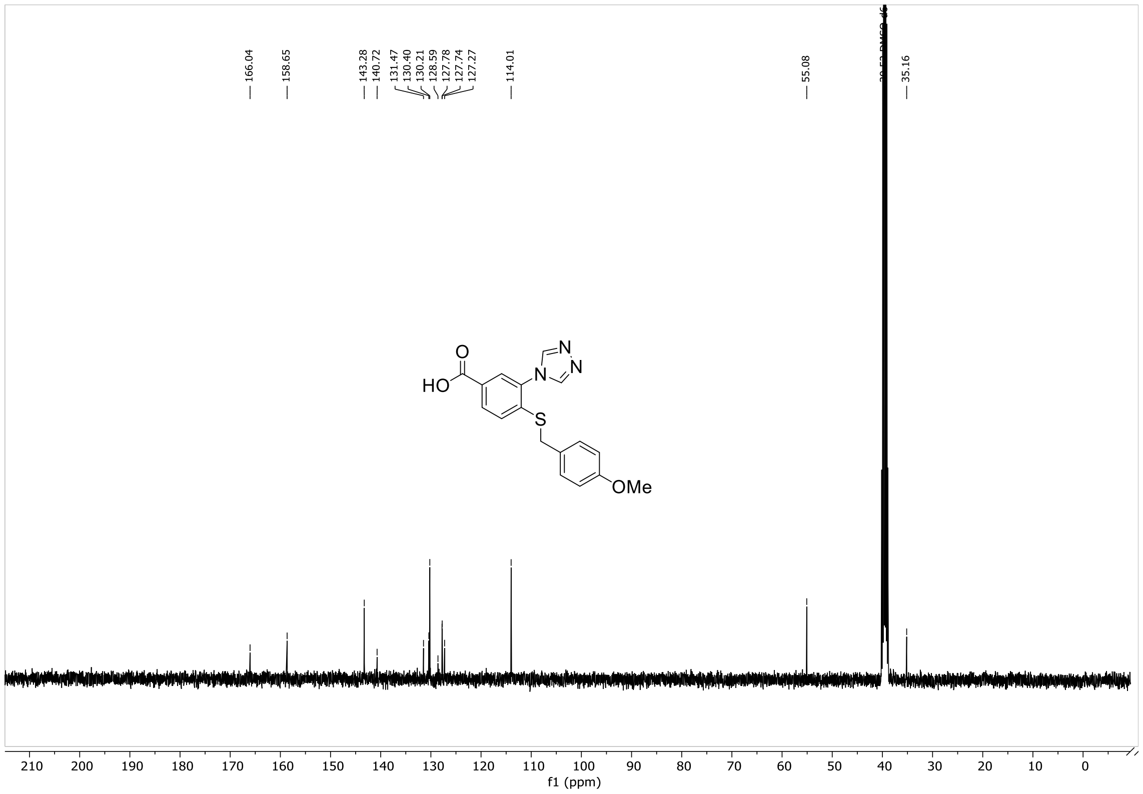


**Figure S113**: $^{13}C\{^1H\}$ NMR spectrum of 4-((4-methoxybenzyl)thio)-3-(4*H*-1,2,4-triazol-4-yl)benzoic acid (**3m**) (100 MHz, DMSO-$d_6$, 298 K).

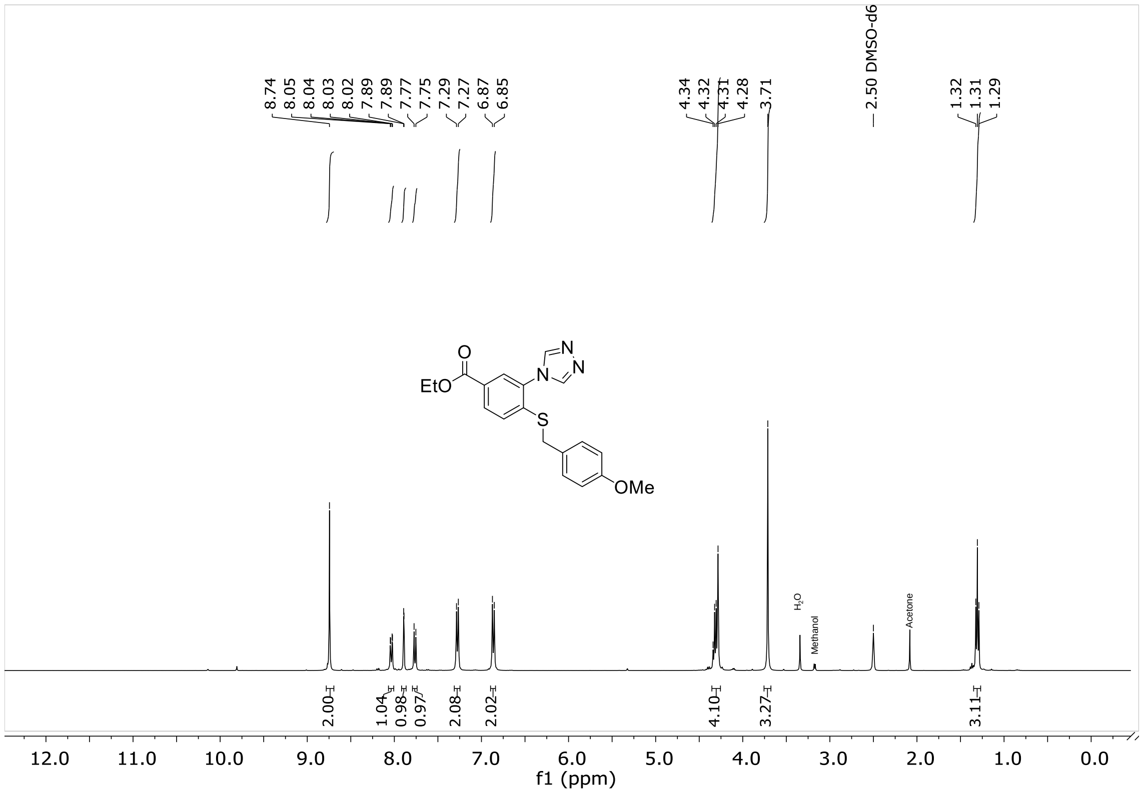


**Figure S114**: $^1H$ NMR spectrum of ethyl 4-((4-methoxybenzyl)thio)-3-(4*H*-1,2,4-triazol-4-yl)benzoate (**3m**) (400 MHz, DMSO-$d_6$, 298 K).

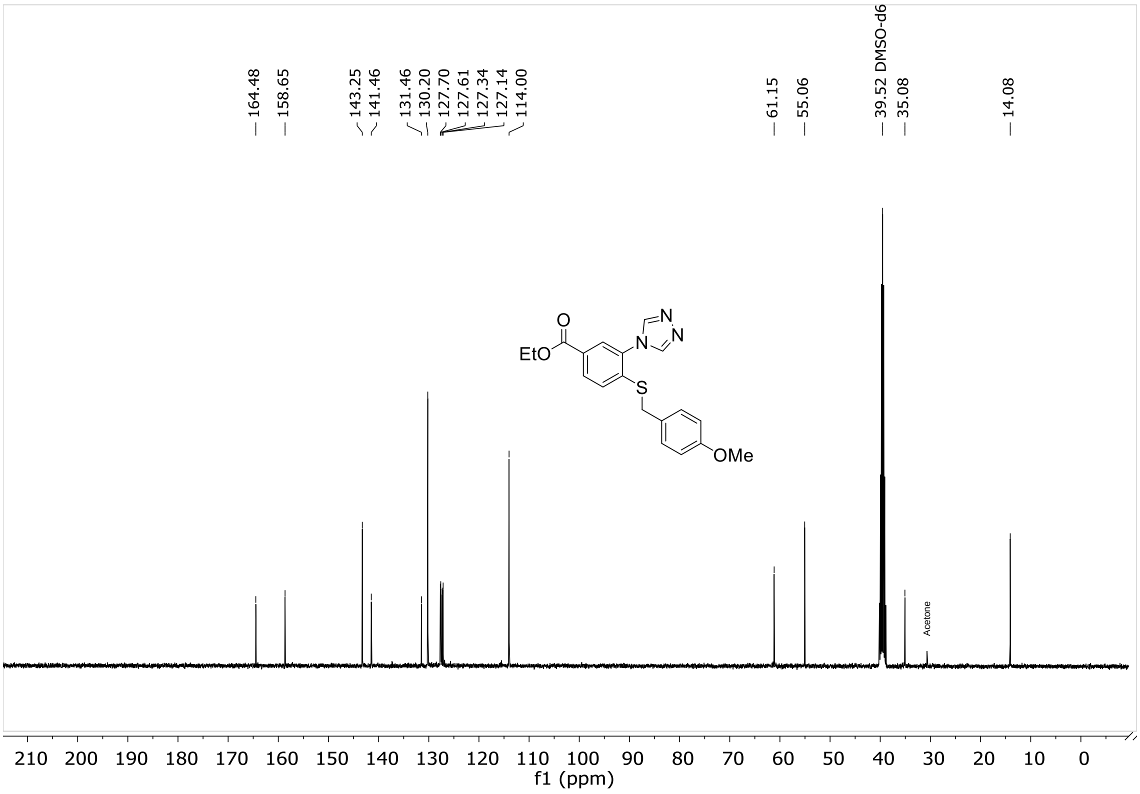


**Figure S115**: $^{13}C\{^{1}H\}$ NMR spectrum of ethyl 4-((4-methoxybenzyl)thio)-3-(4*H*-1,2,4-triazol-4-yl)benzoate (**3m**) (400 MHz, DMSO-$d_6$, 298 K).

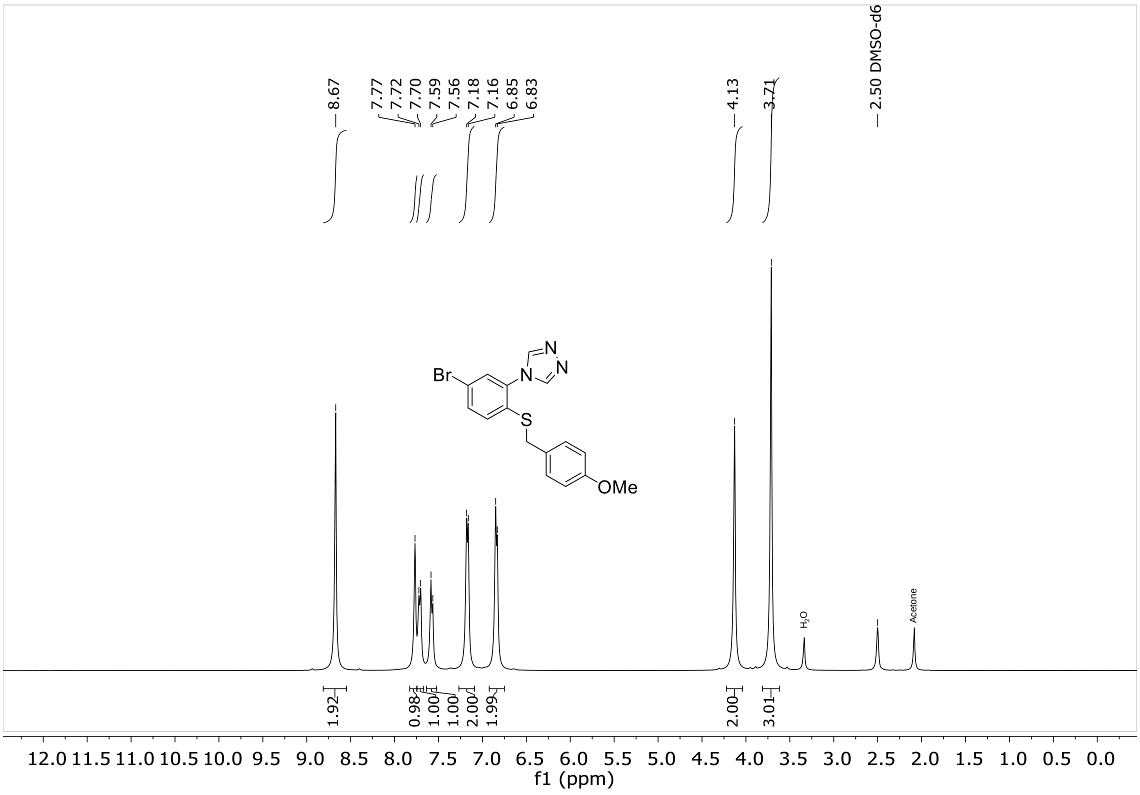


**Figure S116**: $^{1}H$ NMR spectrum of 4-(5-bromo-2-((4-methoxybenzyl)thio)phenyl)-4*H*-1,2,4-triazole (**3n**) (400 MHz, DMSO-$d_6$, 298 K).

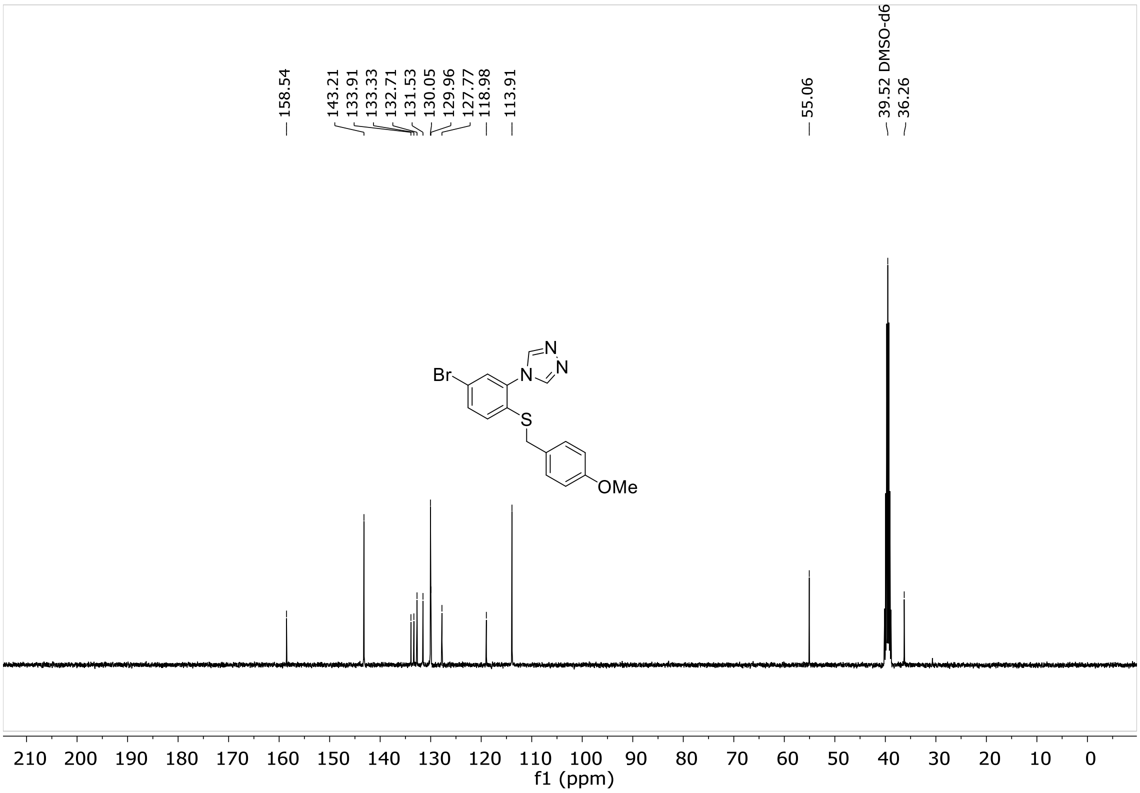


**Figure S117**: $^{13}C\{^{1}H\}$ NMR spectrum of 4-(5-bromo-2-((4-methoxybenzyl)thio)phenyl)-4*H*-1,2,4-triazole (**3n**) (100 MHz, DMSO-$d_6$, 298 K).

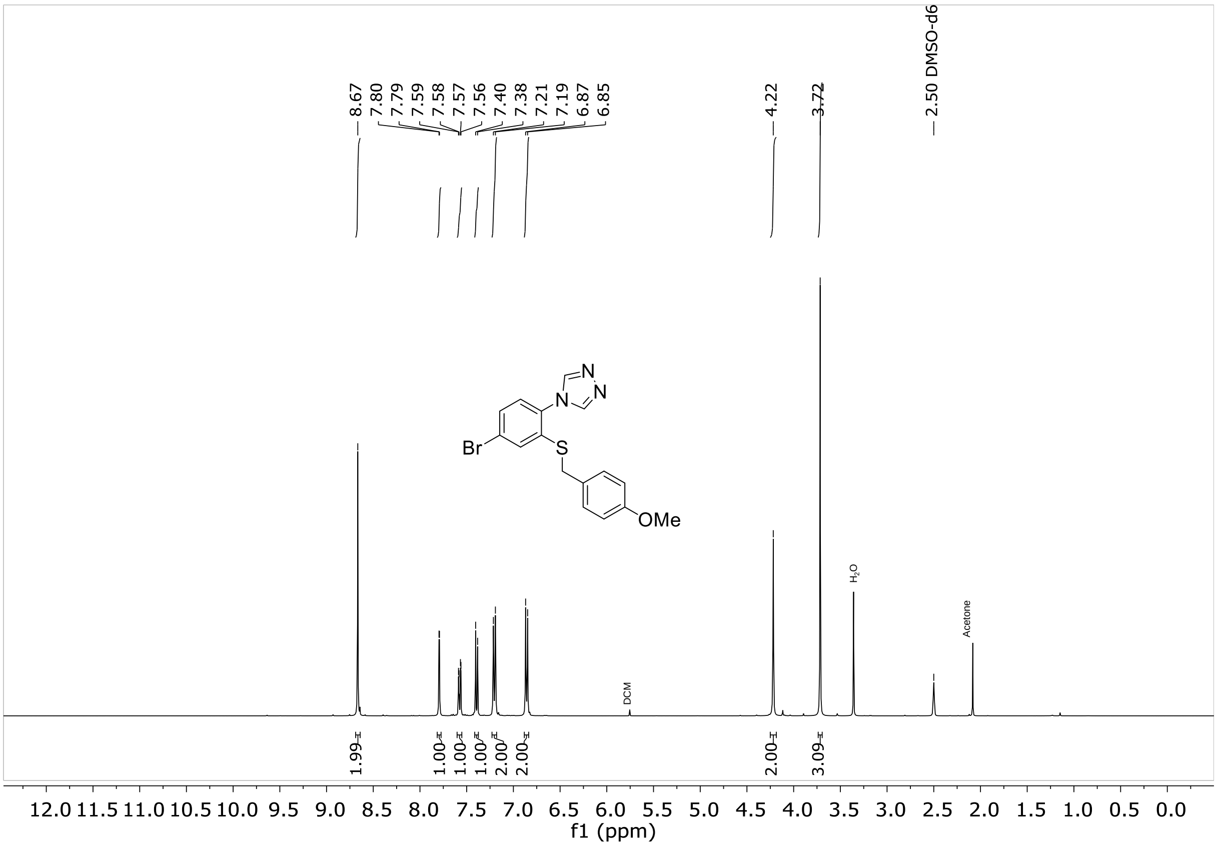


**Figure S118**: $^{1}H$ NMR spectrum of 4-(4-bromo-2-((4-methoxybenzyl)thio)phenyl)-4*H*-1,2,4-triazole (**3o**) (400 MHz, DMSO-$d_6$, 298 K).

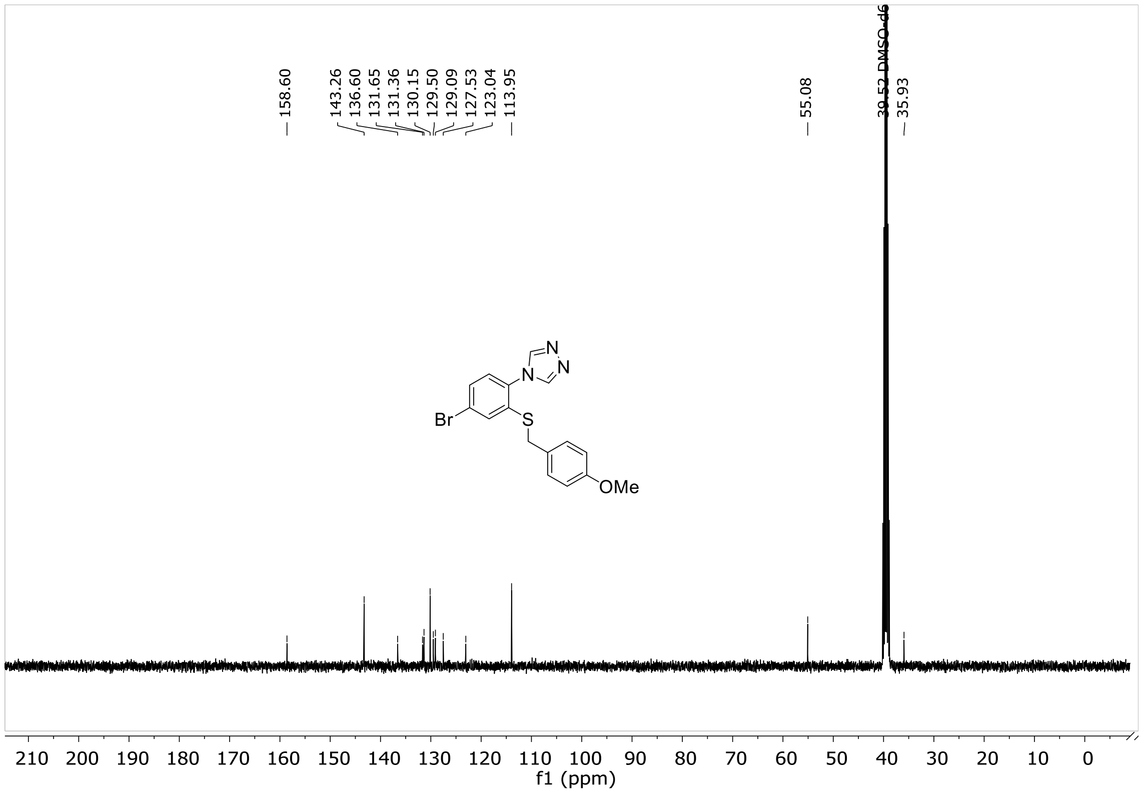

**Figure S119**: $^{13}C\{^{1}H\}$ NMR spectrum of 4-(4-bromo-2-((4-methoxybenzyl)thio)phenyl)-4*H*-1,2,4-triazole (**3o**) (100 MHz, DMSO-$d_6$, 298 K).

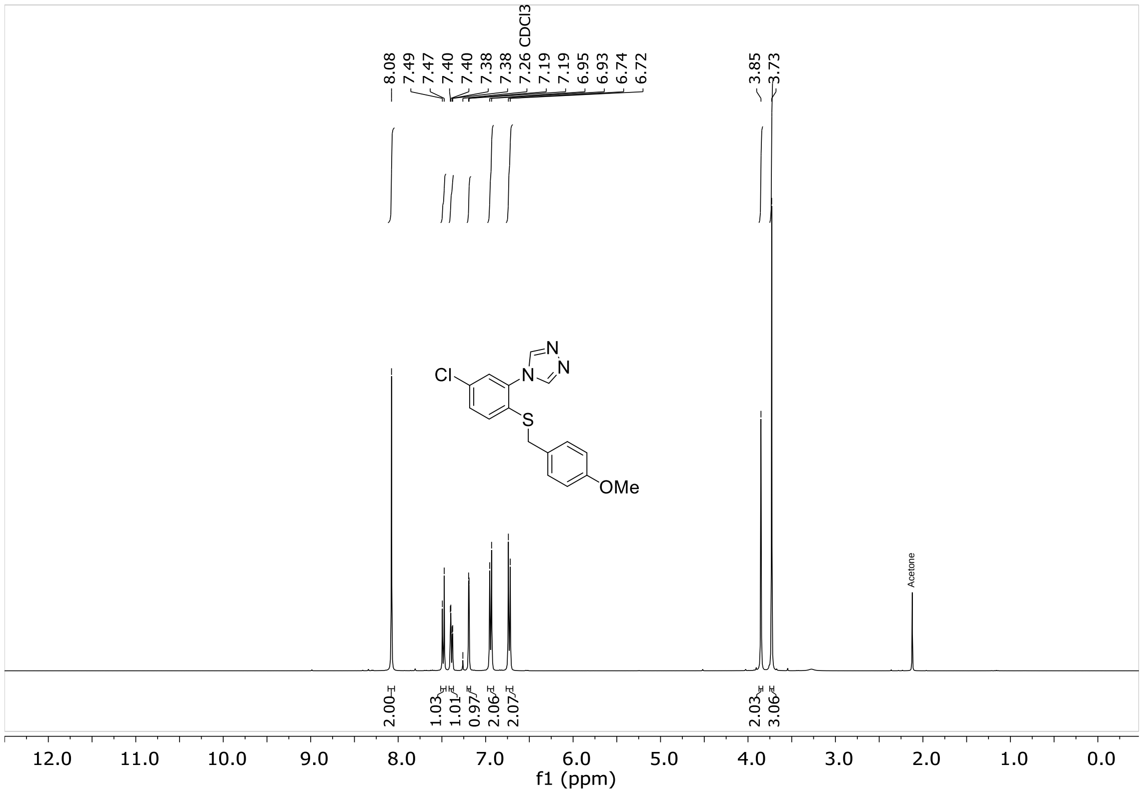

**Figure S120**: $^{1}H$ NMR 4-(5-chloro-2-((4-methoxybenzyl)thio)phenyl)-4*H*-1,2,4-triazole (**3p**) (400 MHz, $CDCl_3$, 298 K).

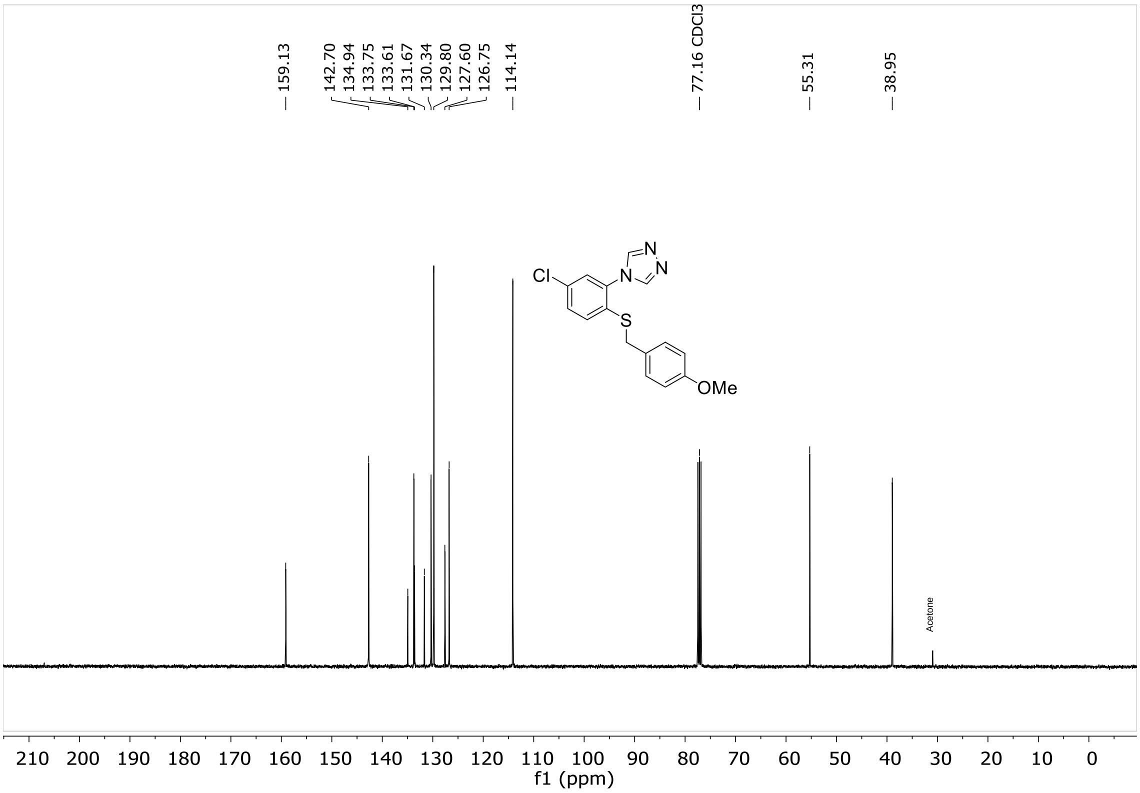

**Figure S121**: $^{13}C\{^{1}H\}$ NMR 4-(5-chloro-2-((4-methoxybenzyl)thio)phenyl)-4*H*-1,2,4-triazole (**3p**) (100 MHz, $CDCl_3$, 298 K).

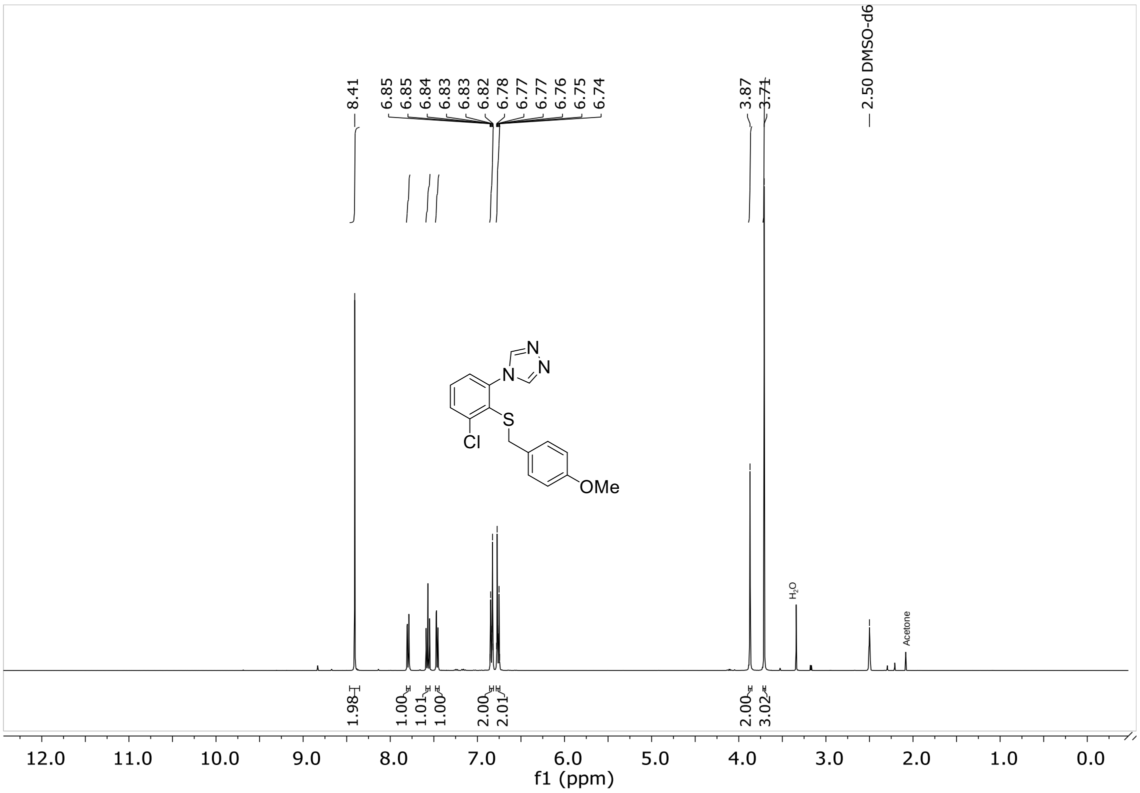

**Figure S122**: $^{1}H$ NMR spectrum of 4-(3-chloro-2-((4-methoxybenzyl)thio)phenyl)-4*H*-1,2,4-triazole (**3q**) (400 MHz, DMSO-$d_6$, 298 K, 298 K).

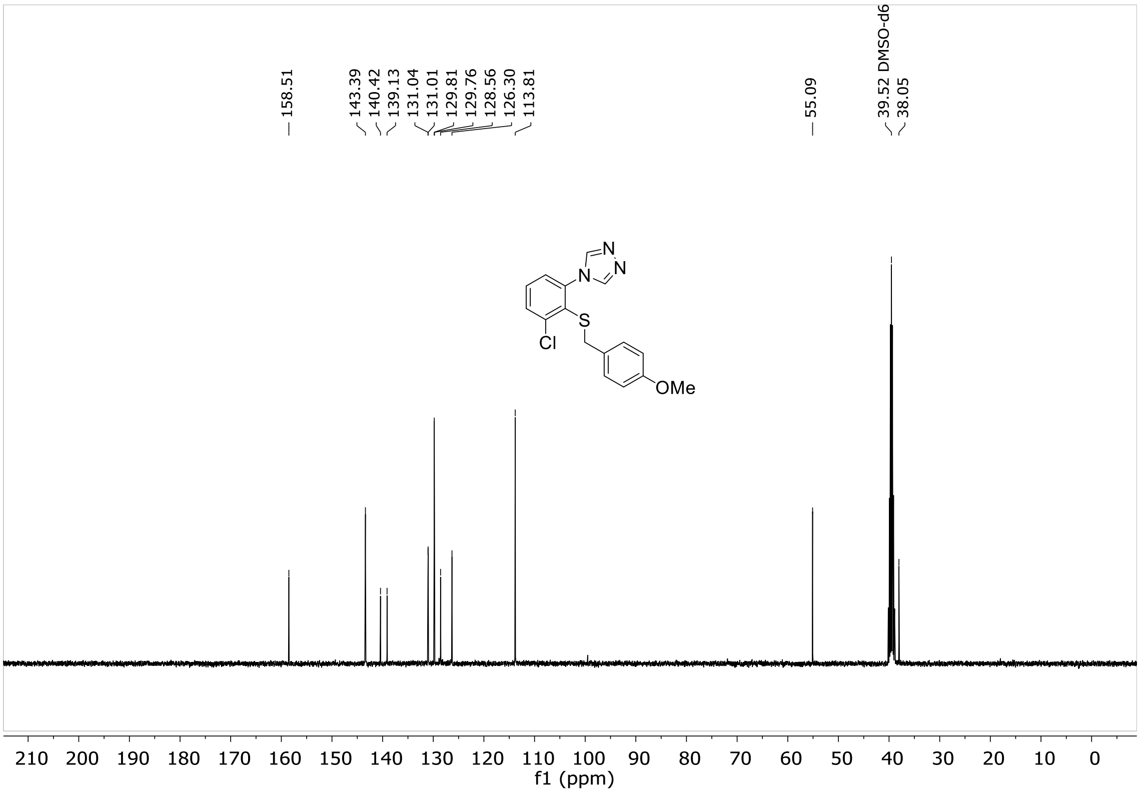


**Figure S123**: $^{13}C\{^1H\}$ NMR spectrum of 4-(3-chloro-2-((4-methoxybenzyl)thio)phenyl)-4*H*-1,2,4-triazole (**3q**) (100 MHz, DMSO-$d_6$, 298 K, 298 K).

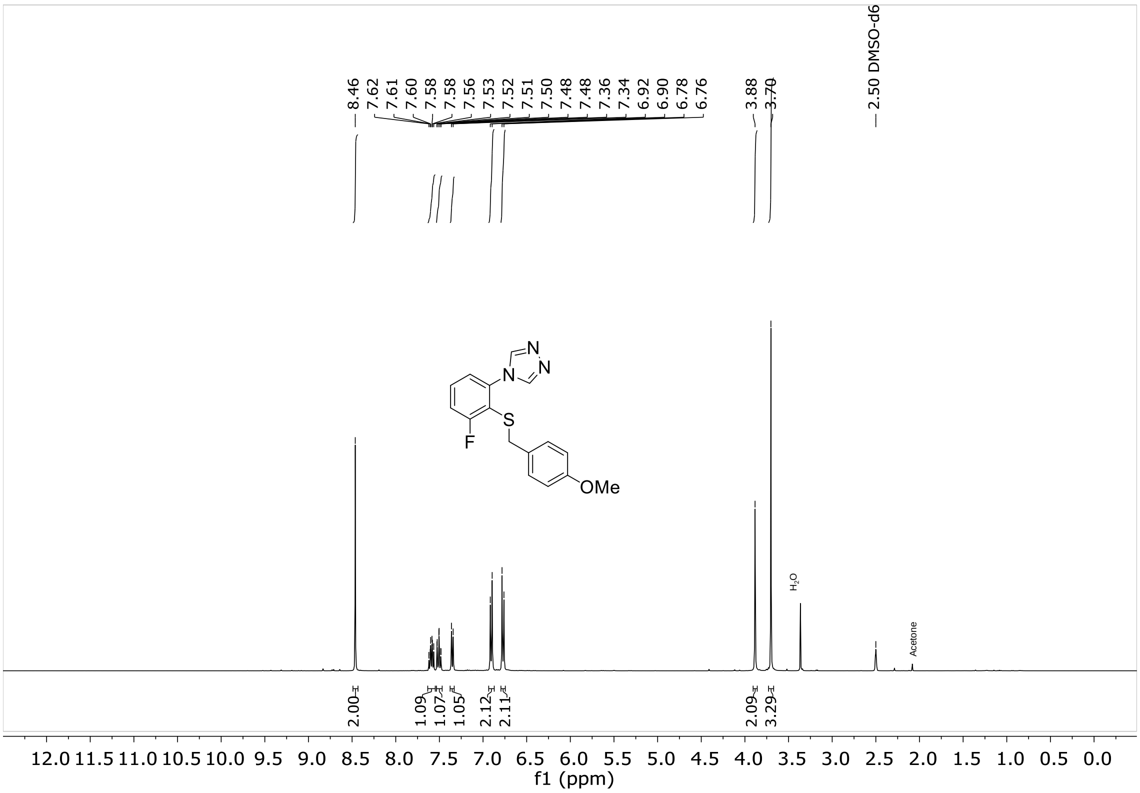


**Figure S124**: $^1H$ NMR spectrum of 4-(3-fluoro-2-((4-methoxybenzyl)thio)phenyl)-4*H*-1,2,4-triazole (**3r**) (400 MHz, DMSO-$d_6$, 298 K).

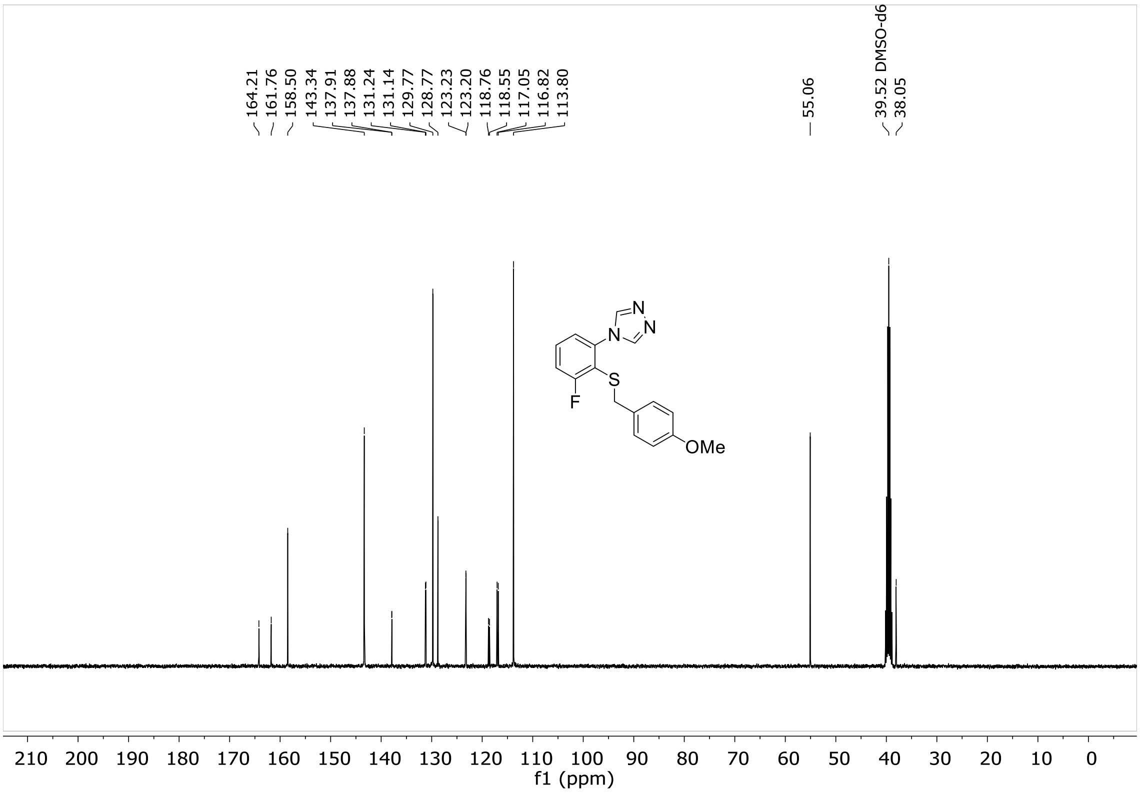


**Figure S125**: $^{13}C\{^{1}H\}$ NMR spectrum of 4-(3-fluoro-2-((4-methoxybenzyl)thio)phenyl)-4*H*-1,2,4-triazole (**3r**) (100 MHz, DMSO-$d_6$, 298 K).

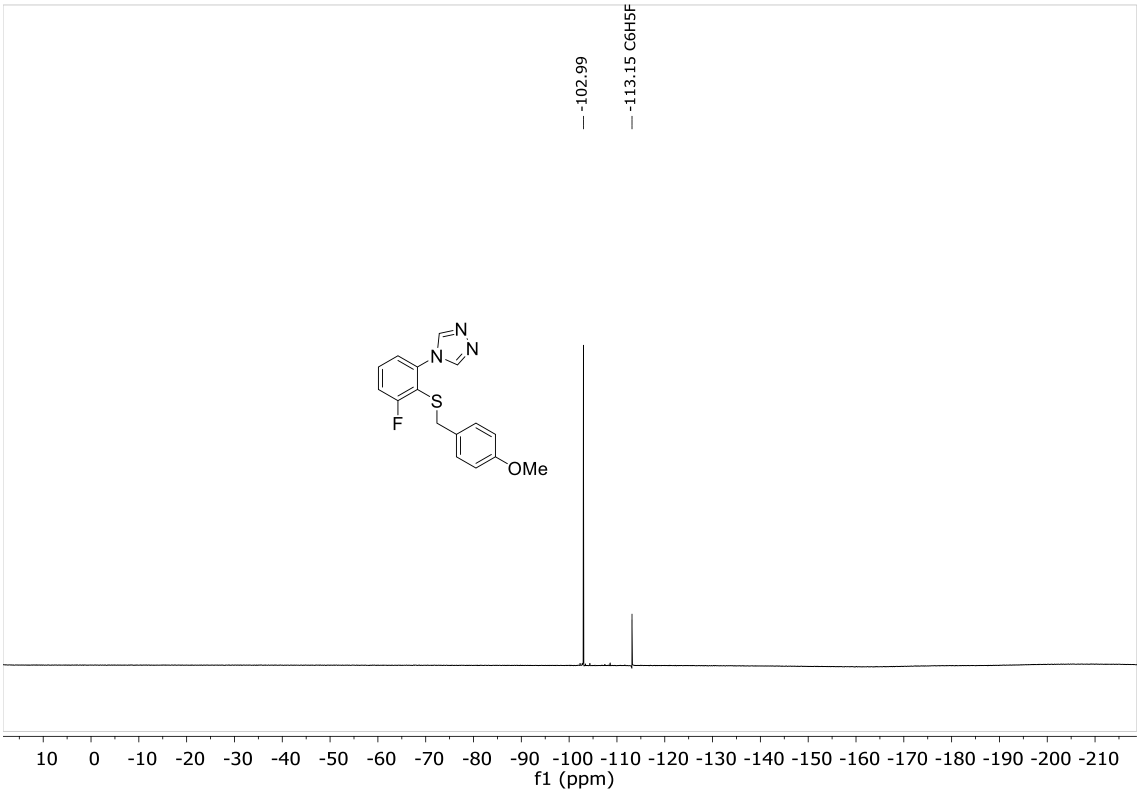


**Figure S126**: $^{19}F\{^{1}H\}$ NMR spectrum of 4-(3-fluoro-2-((4-methoxybenzyl)thio)phenyl)-4*H*-1,2,4-triazole (**3r**) (376 MHz, DMSO-$d_6$, 298 K, referenced to fluorobenzene).

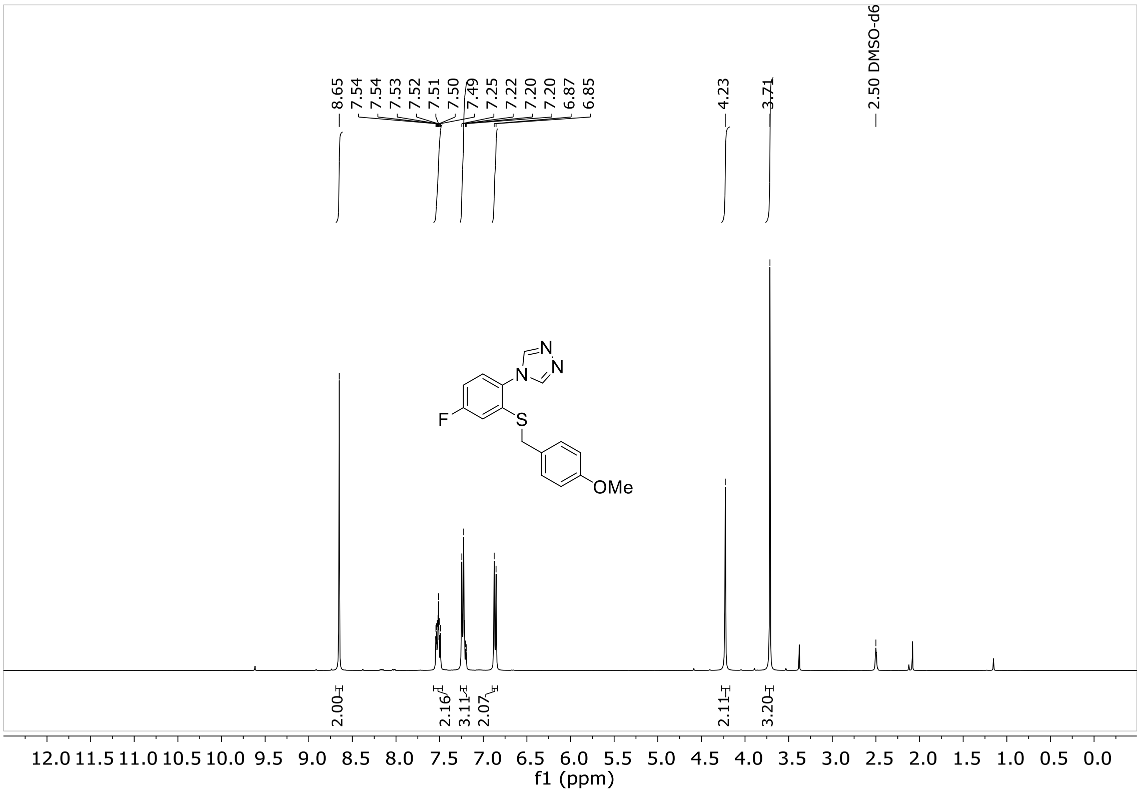

**Figure S127**: $^{1}$H NMR spectrum of 4-(4-fluoro-2-((4-methoxybenzyl)thio)phenyl)-4*H*-1,2,4-triazole (**3s**) (400 MHz, DMSO-$d_6$, 298 K).

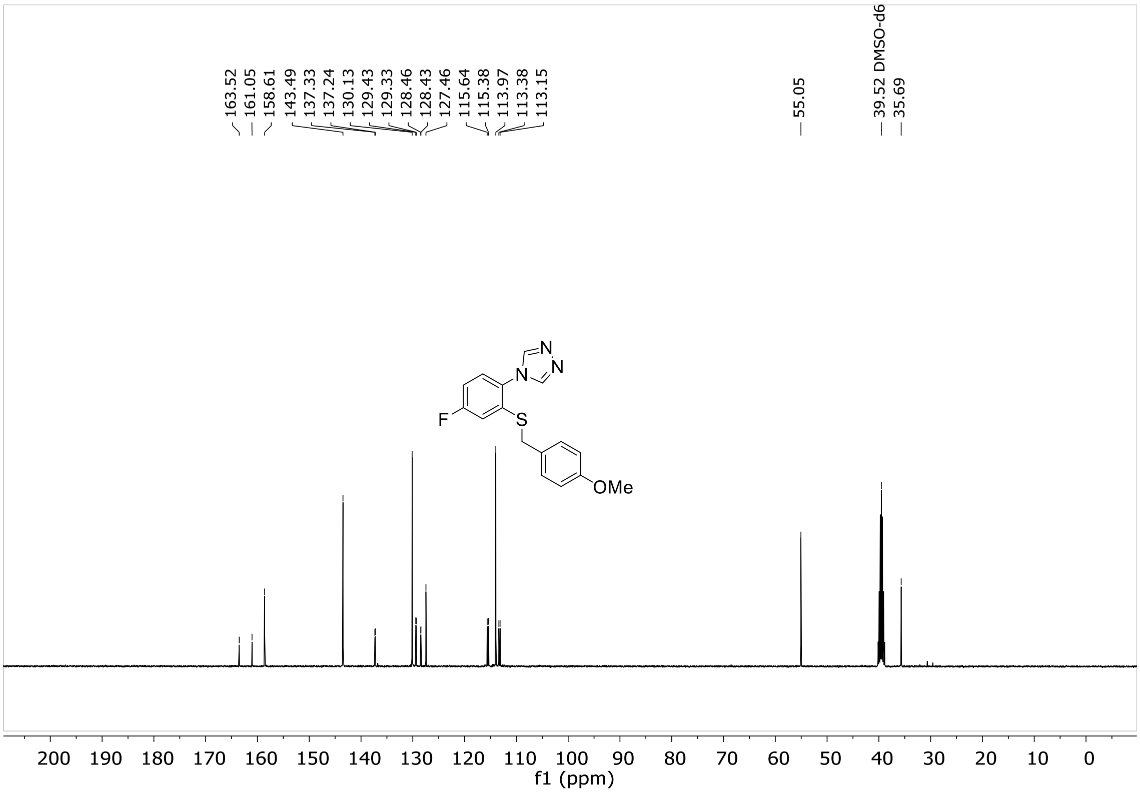

**Figure S128**: $^{13}$C{$^{1}$H} NMR spectrum of 4-(4-fluoro-2-((4-methoxybenzyl)thio)phenyl)-4*H*-1,2,4- (**3s**) (100 MHz, DMSO-$d_6$, 298 K).

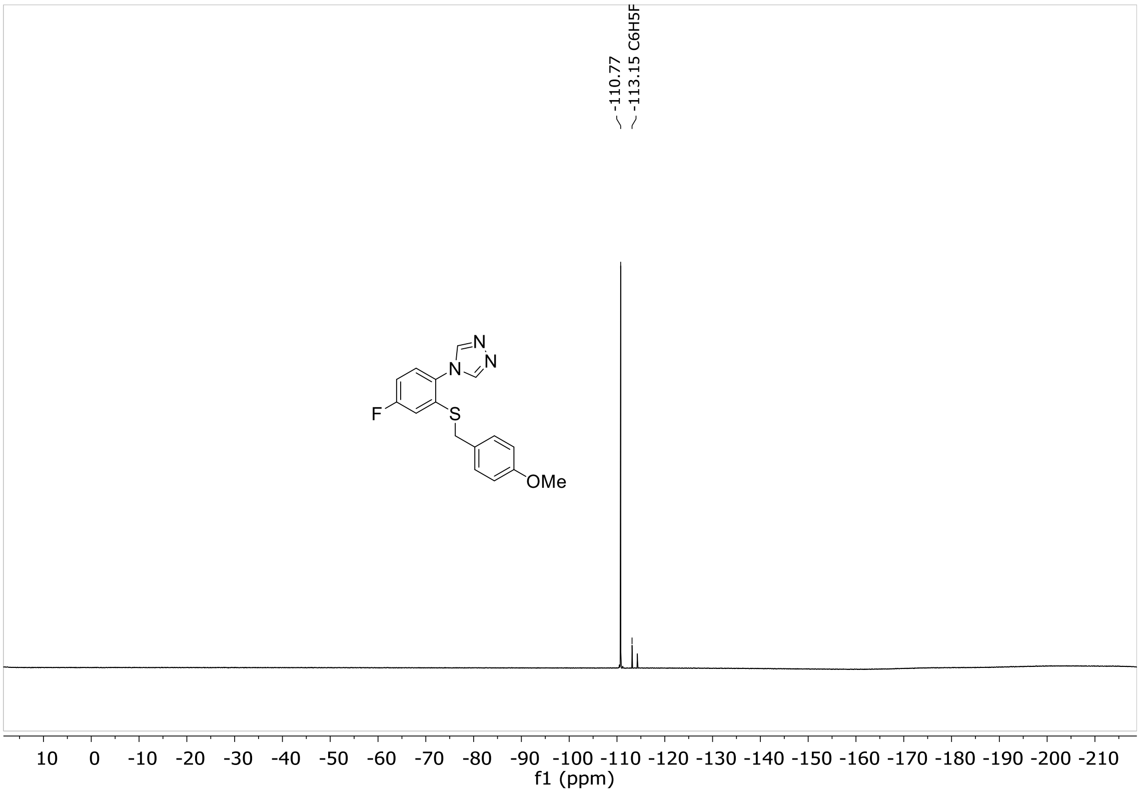


**Figure S129**: $^{19}F\{^{1}H\}$ NMR spectrum of 4-(4-fluoro-2-((4-methoxybenzyl)thio)phenyl)-4*H*-1,2,4-triazole (**3s**) (376 MHz, DMSO-$d_6$, 298 K, referenced to fluorobenzene).

## *1.5 NMR Spectra of Benzo[4,5]thiazolo[2,3-c][1,2,4]triazoles (6a-6s)*

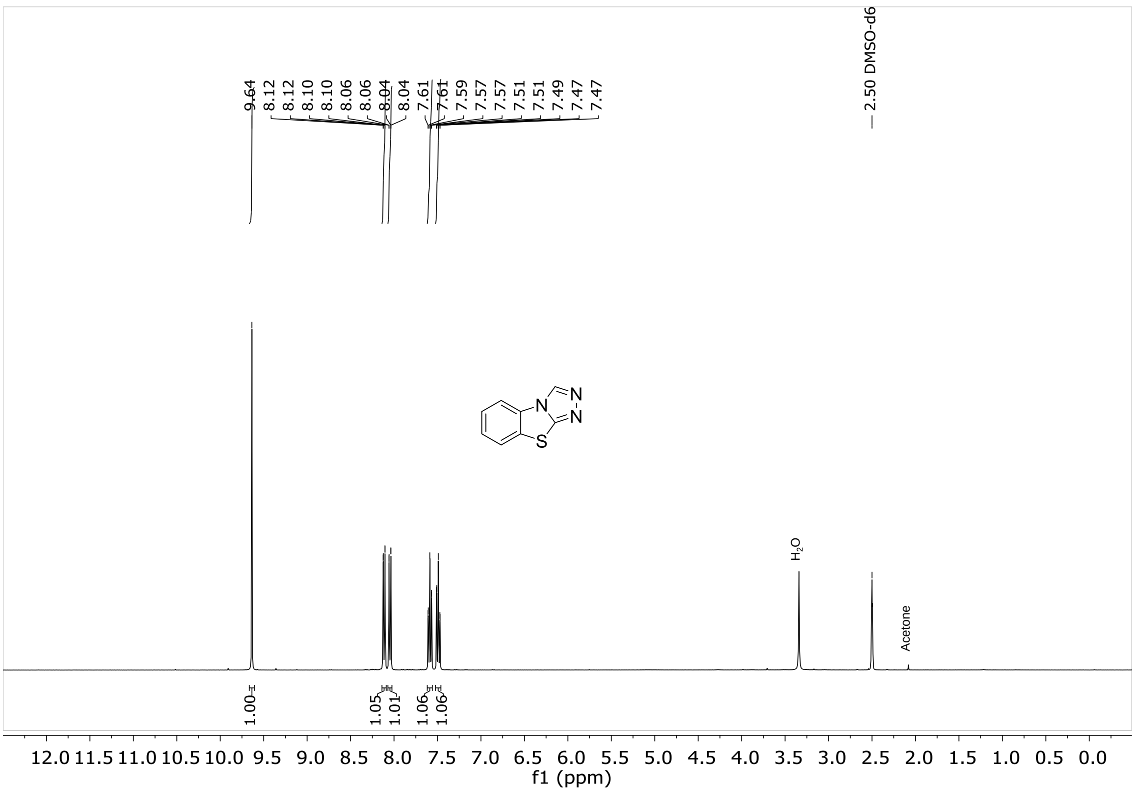


**Figure S130**: $^1$H NMR spectrum of benzo[4,5]thiazolo[2,3-*c*][1,2,4]triazole (**6a**) (400 MHz, DMSO-$d_6$, 298 K).

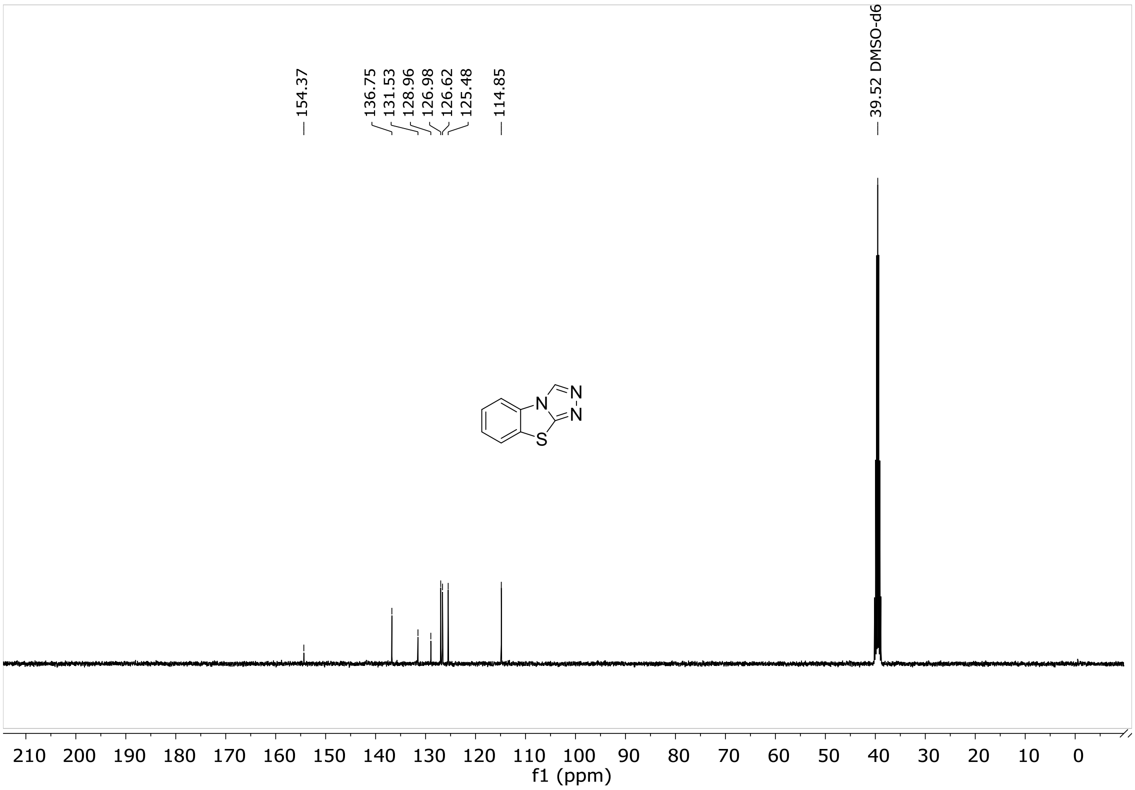


**Figure S131**: $^{13}$C{$^1$H} NMR spectrum of benzo[4,5]thiazolo[2,3-*c*][1,2,4]triazole (**6a**) (100 MHz, DMSO-$d_6$, 298 K).

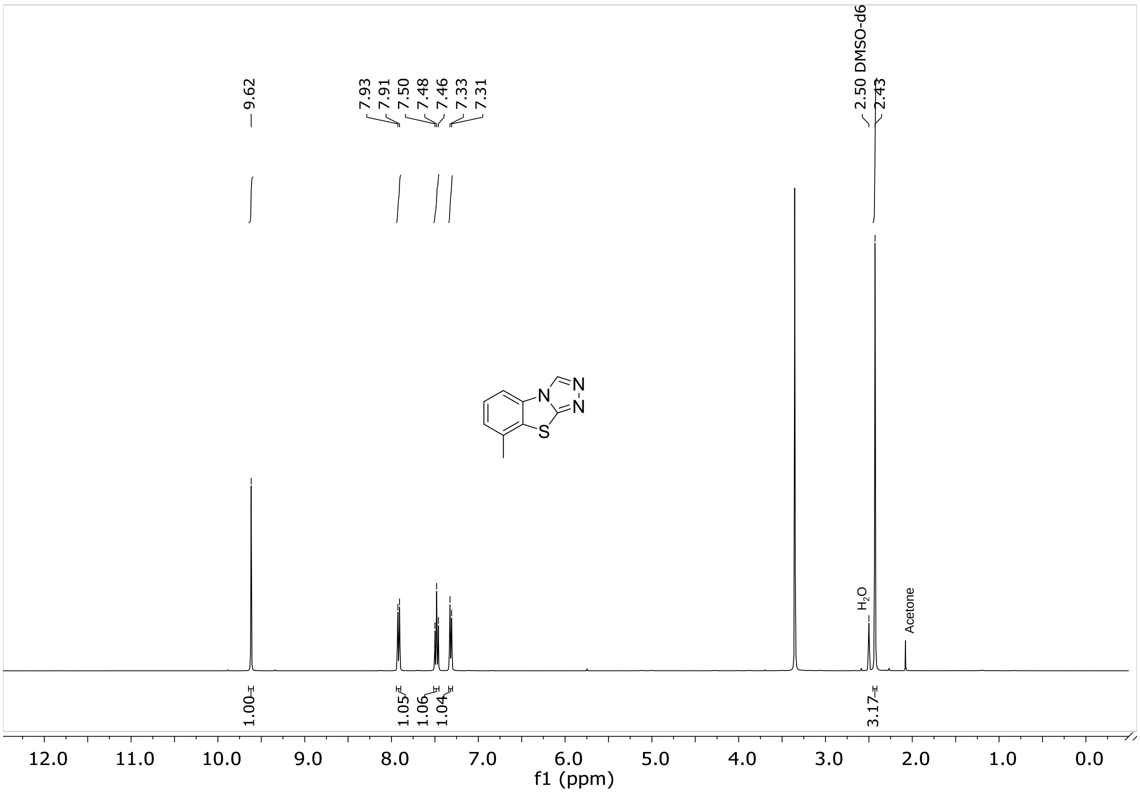


**Figure S132**: $^{1}$H NMR spectrum of 5-methylbenzo[4,5]thiazolo[2,3-*c*][1,2,4]triazole (**6b**) (400 MHz, DMSO-$d_6$, 298 K).

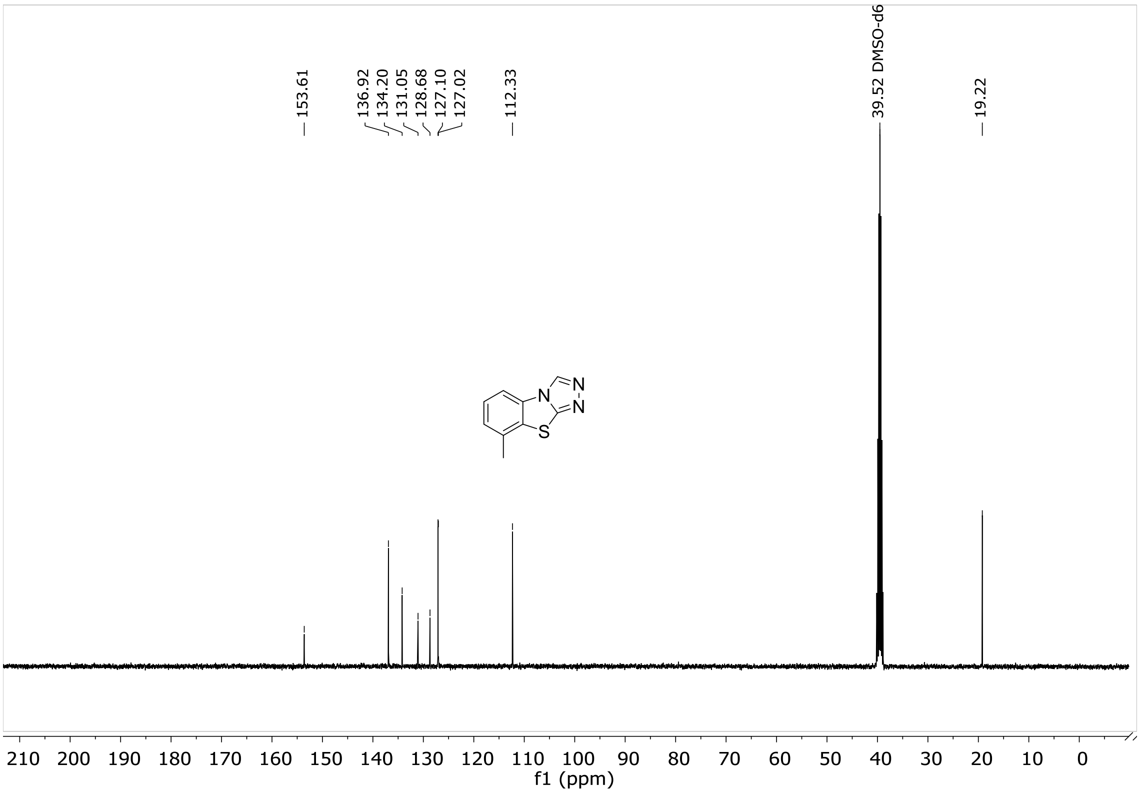


**Figure S133**: $^{13}$C{$^{1}$H} NMR spectrum of 5-methylbenzo[4,5]thiazolo[2,3-*c*][1,2,4]triazole (**6b**) (100 MHz, DMSO-$d_6$, 298 K).

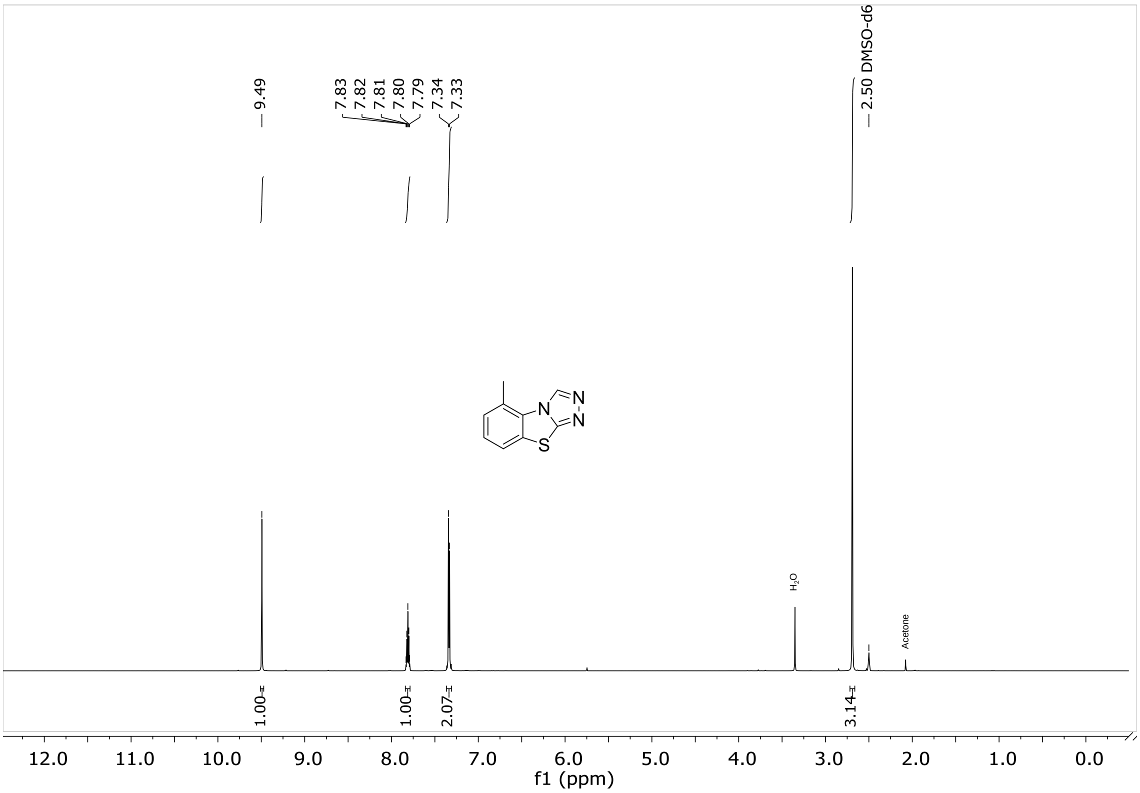


**Figure S134**: $^{1}H$ NMR spectrum of 5-methylbenzo[4,5]thiazolo[2,3-*c*][1,2,4]triazole (**6c**) (400 MHz, DMSO-$d_6$, 298 K).

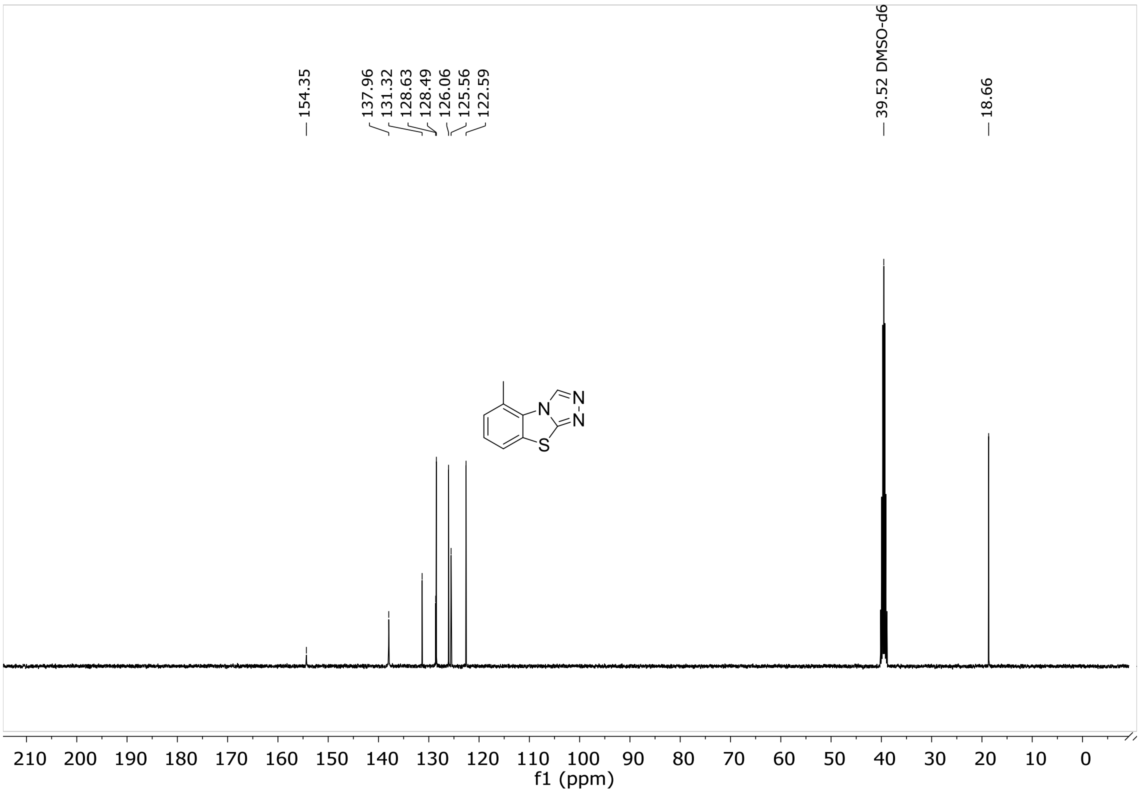


**Figure S135**: $^{13}C\{^{1}H\}$ NMR spectrum of 5-methylbenzo[4,5]thiazolo[2,3-*c*][1,2,4]triazole (**6c**) (100 MHz, DMSO-$d_6$, 298 K).

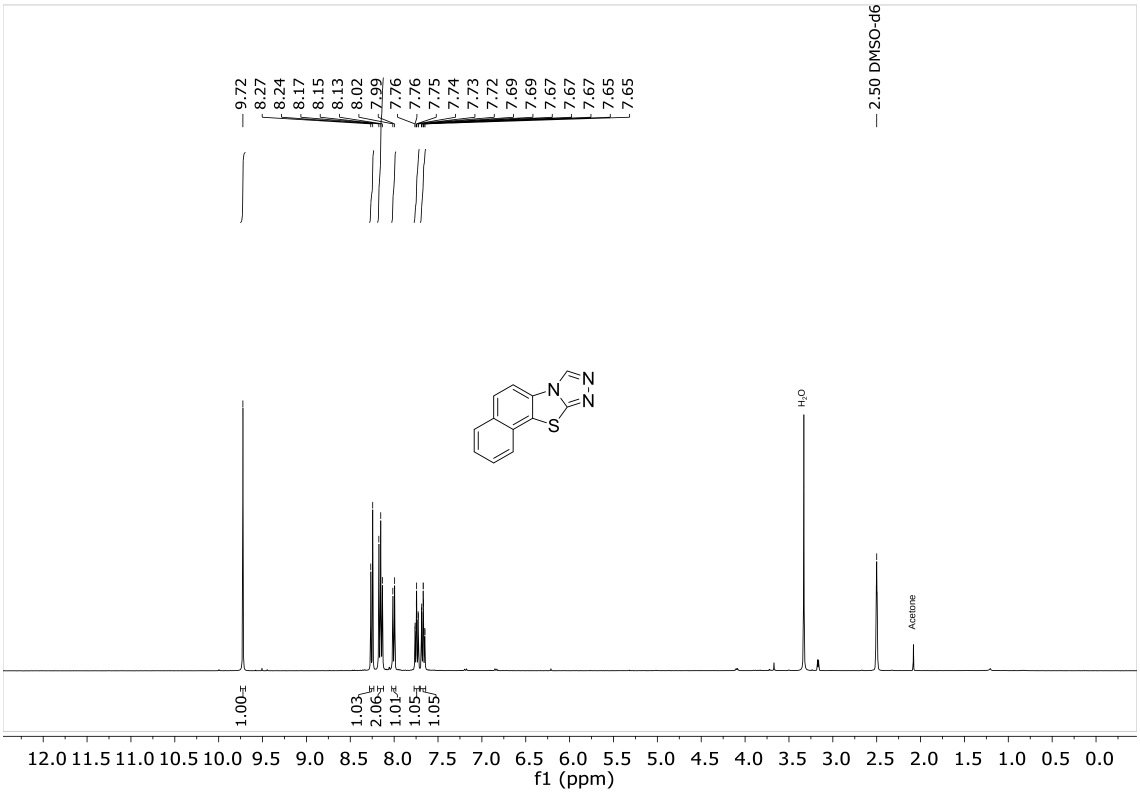


**Figure S136**: $^{1}$H NMR spectrum of naphtho[2',1':4,5]thiazolo[2,3-*c*][1,2,4]triazole (**6d**) (400 MHz, DMSO-$d_6$, 298 K).

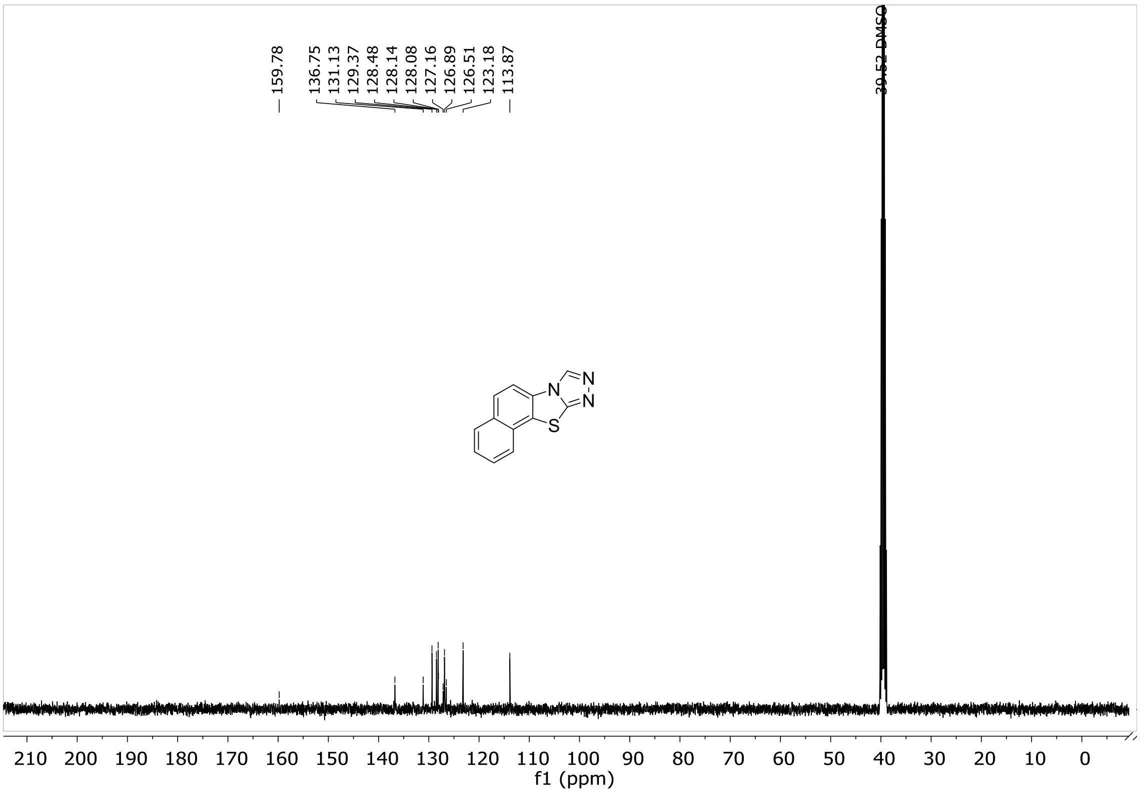


**Figure S137**: $^{13}$C{$^{1}$H} NMR spectrum of naphtho[2',1':4,5]thiazolo[2,3-*c*][1,2,4]triazole (**6d**) (100 MHz, DMSO-$d_6$, 298 K).

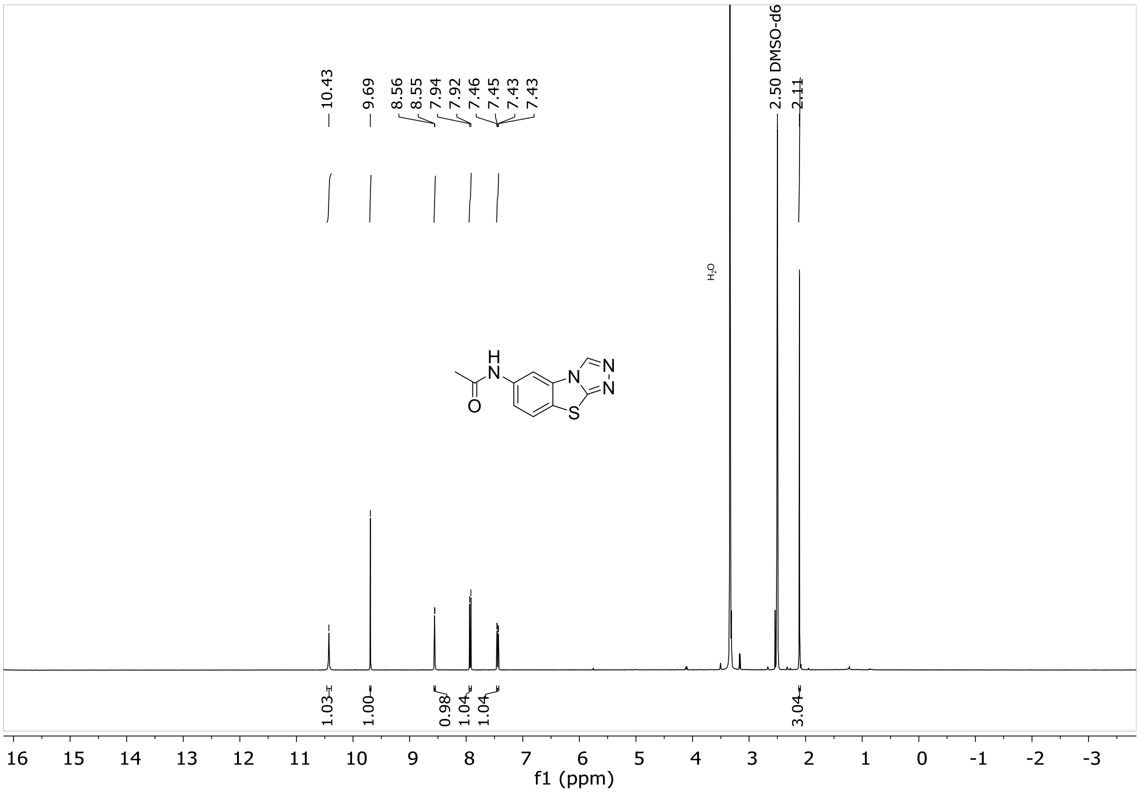

**Figure S138**: $^{1}$H NMR spectrum of *N*-(benzo[4,5]thiazolo[2,3-*c*][1,2,4]triazol-6-yl)acetamide (**6e**) (400 MHz, DMSO-$d_6$, 298 K).

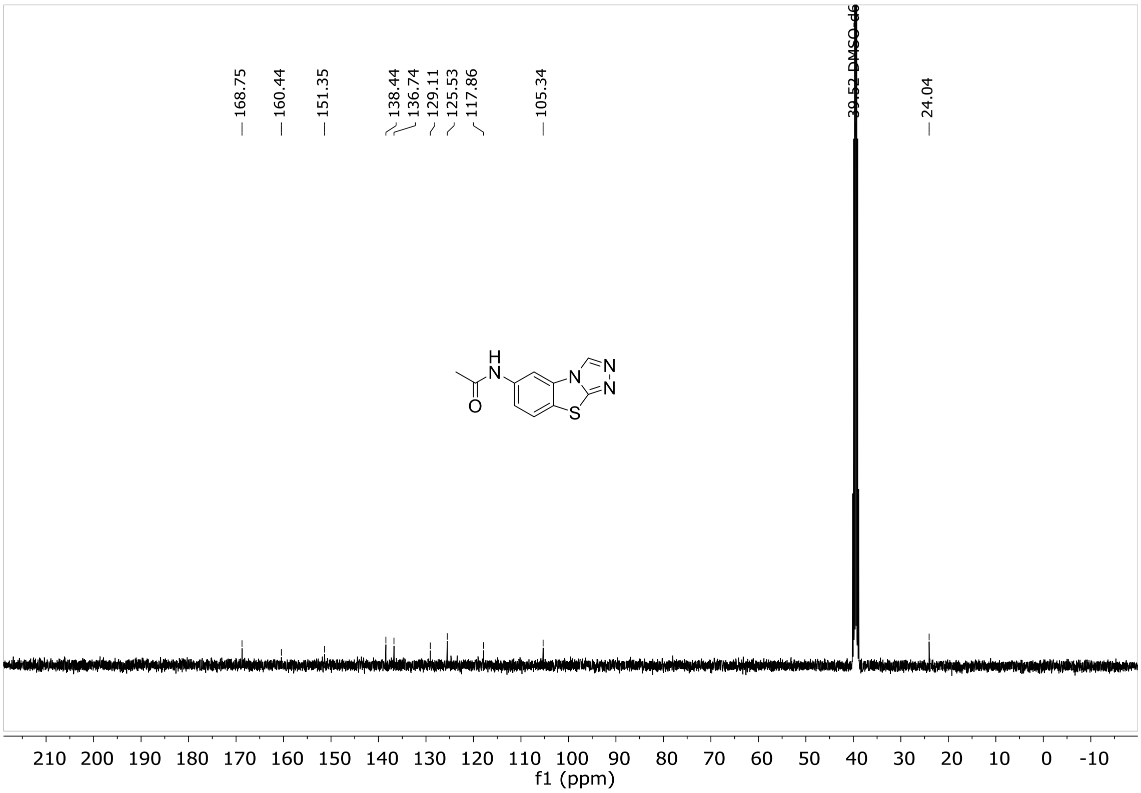

**Figure S139**: $^{13}C\{^1H\}$ NMR spectrum of *N*-(benzo[4,5]thiazolo[2,3-*c*][1,2,4]triazol-6-yl)acetamide (**6e**) (100 MHz, DMSO-$d_6$, 298 K).

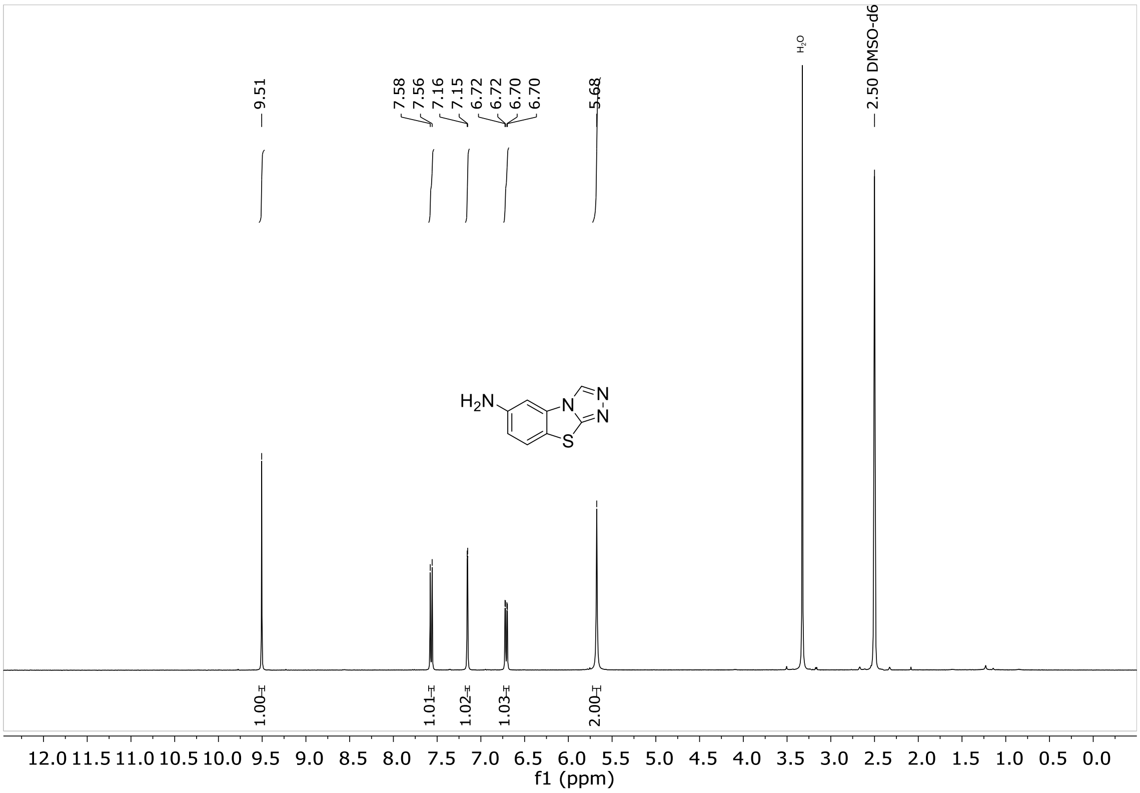


**Figure S140**: $^{1}$H NMR spectrum of benzo[4,5]thiazolo[2,3-*c*][1,2,4]triazol-6-amine (**6f**) (400 MHz, DMSO-$d_6$, 298 K).

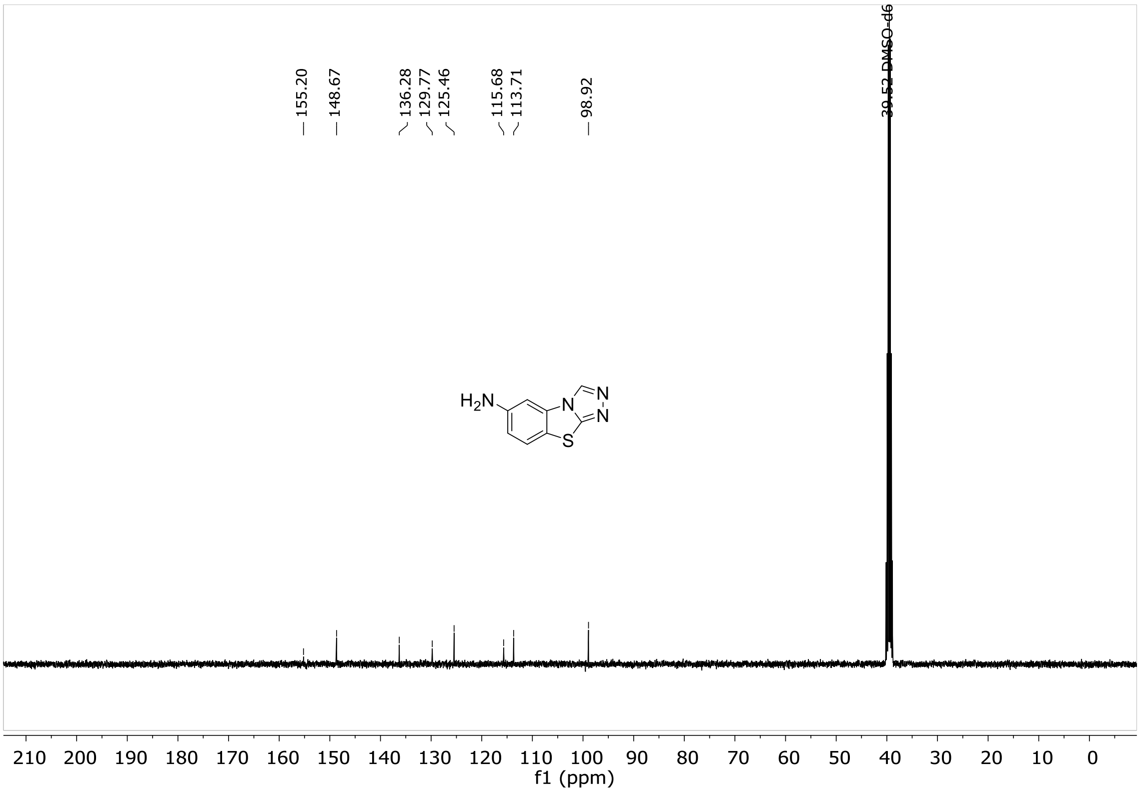


**Figure S141**: $^{13}$C{$^{1}$H} NMR spectrum of benzo[4,5]thiazolo[2,3-*c*][1,2,4]triazol-6-amine (**6f**) (100 MHz, DMSO-$d_6$, 298 K).

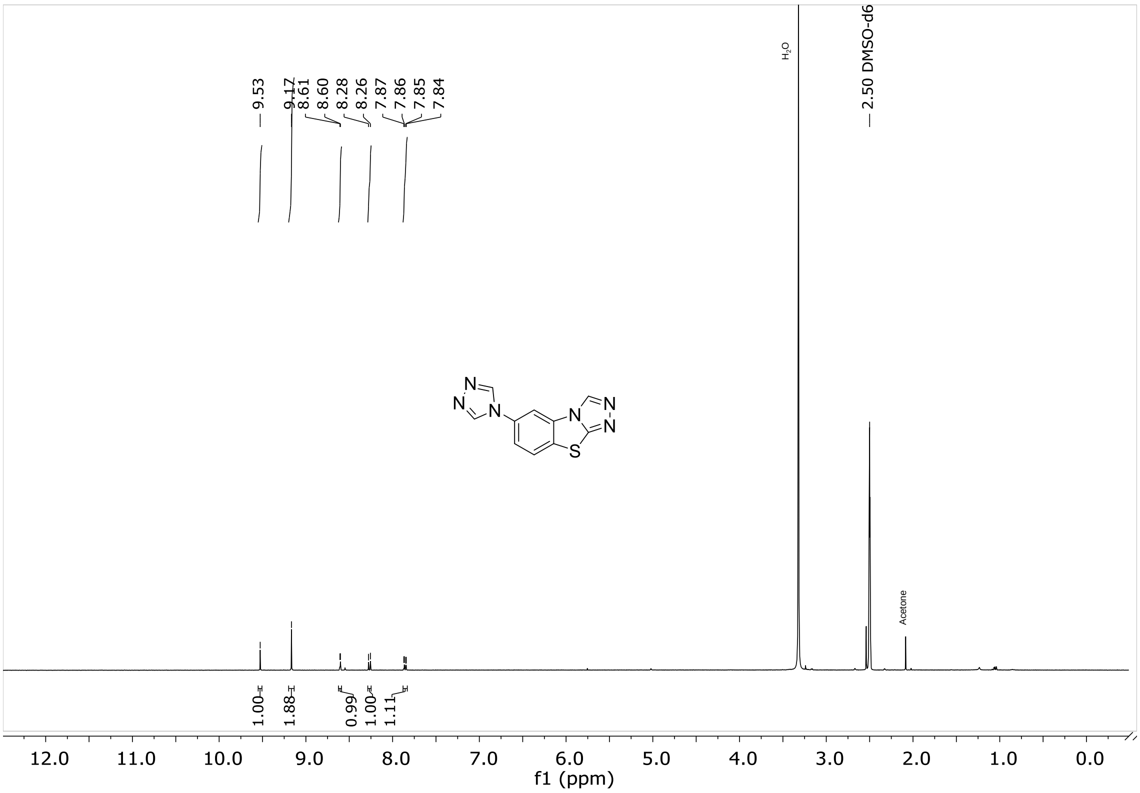


**Figure S142**: $^{1}$H NMR spectrum of 6-(4H-1,2,4-triazol-4-yl)benzo[4,5]thiazolo[2,3-*c*][1,2,4]triazole (**6f'**) (400 MHz, DMSO-$d_6$, 298 K).

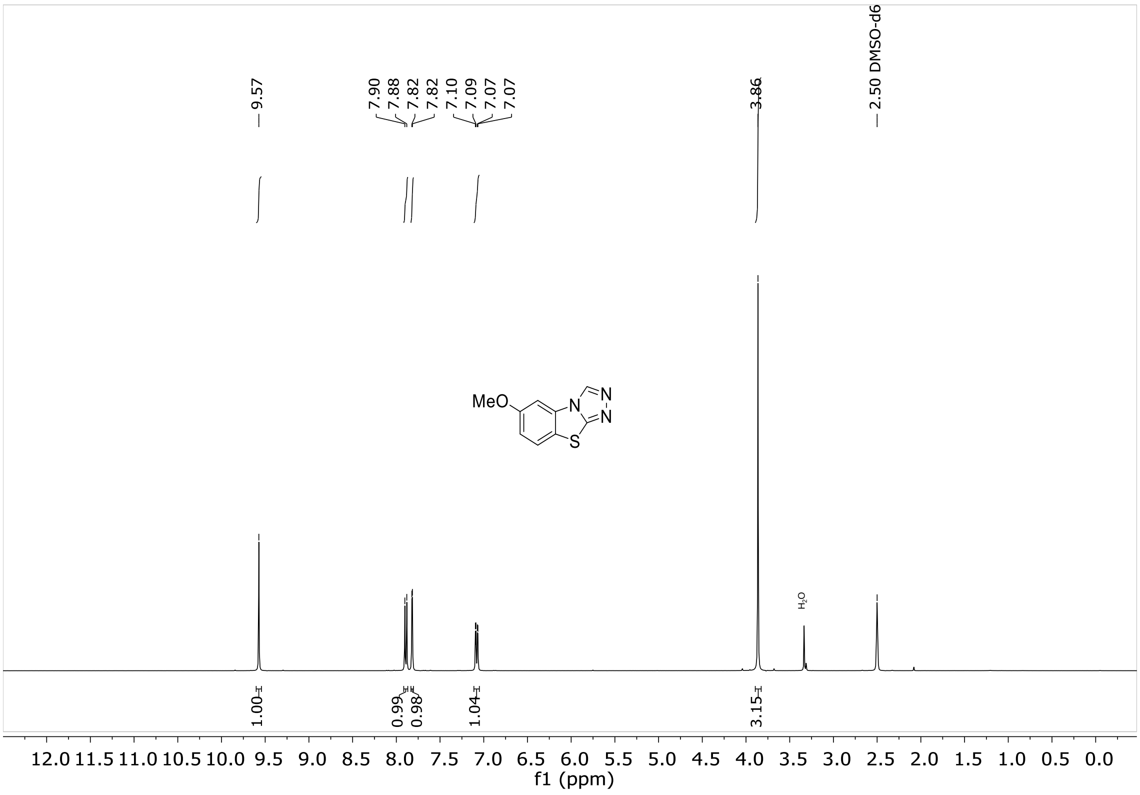


**Figure S143**: $^{1}$H NMR spectrum of 6-methoxybenzo[4,5]thiazolo[2,3-*c*][1,2,4]triazole (**6g**) (400 MHz, DMSO-$d_6$, 298 K).

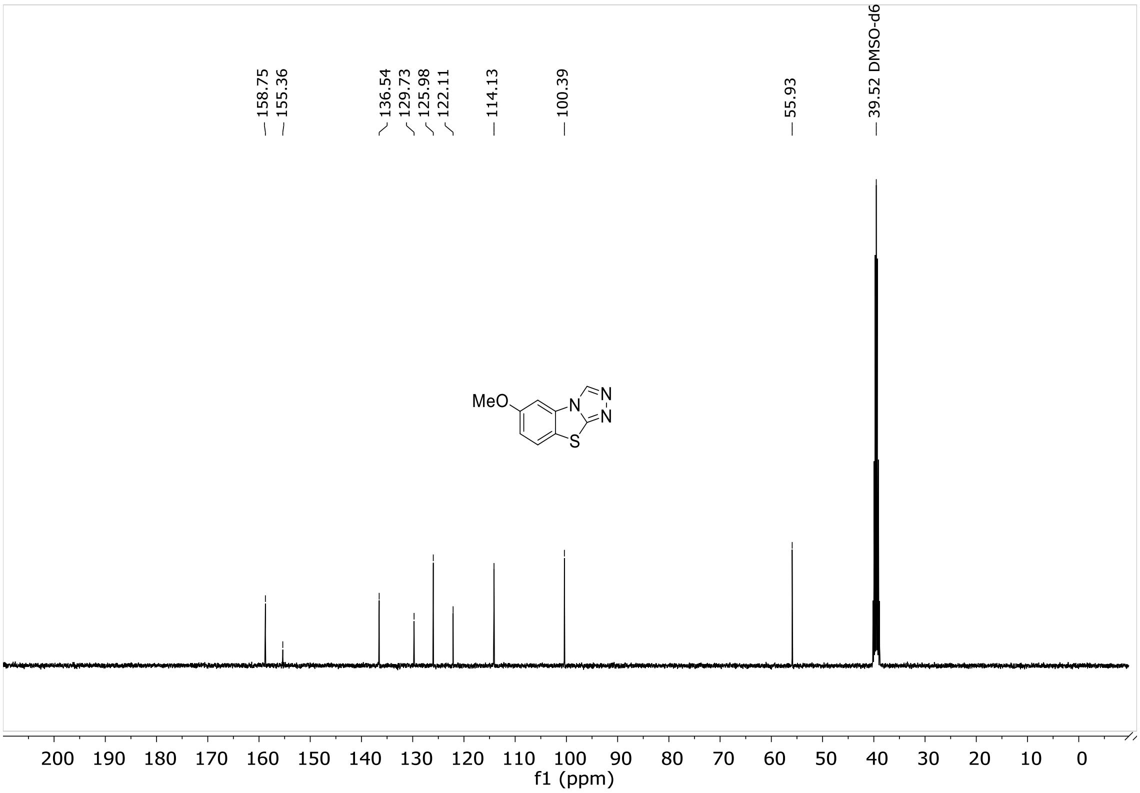


**Figure S144**: $^{13}C\{^{1}H\}$ NMR spectrum of 6-methoxybenzo[4,5]thiazolo[2,3-*c*][1,2,4]triazole (**6g**) (100 MHz, DMSO-$d_6$, 298 K).

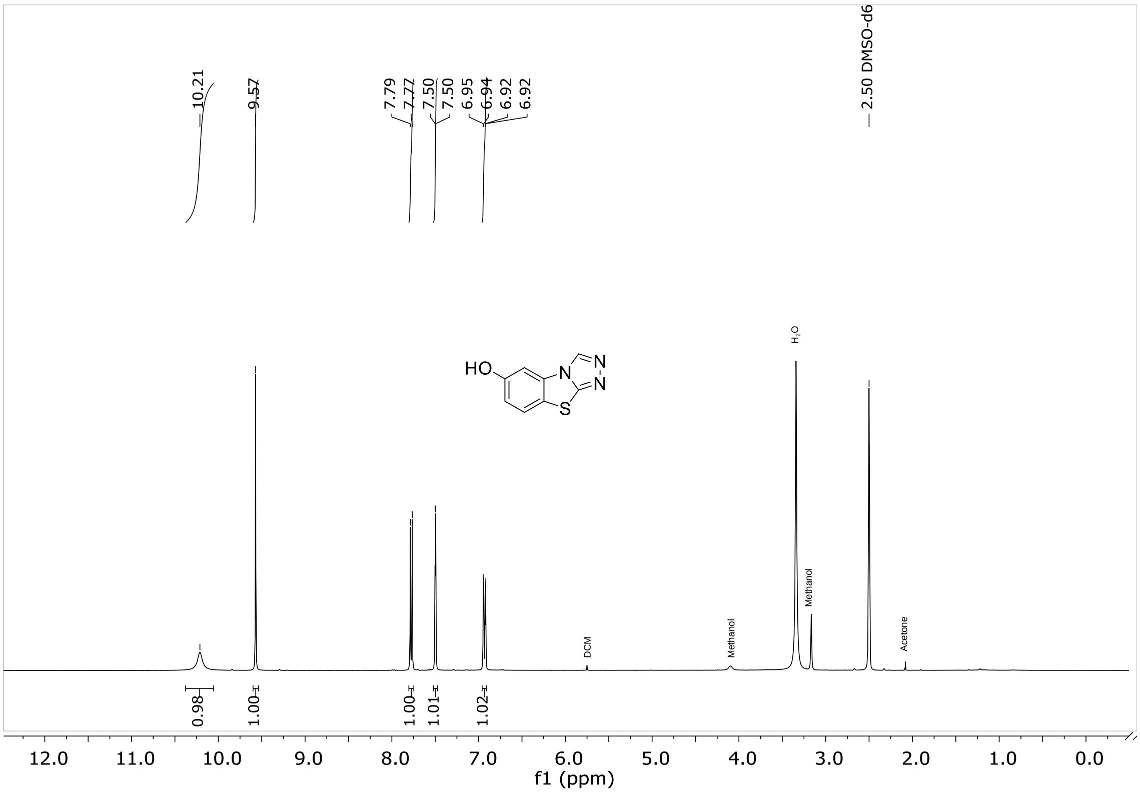


**Figure S145**: $^{1}H$ NMR spectrum of benzo[4,5]thiazolo[2,3-*c*][1,2,4]triazol-6-ol (**6h)** (400 MHz, DMSO-$d_6$, 298 K).

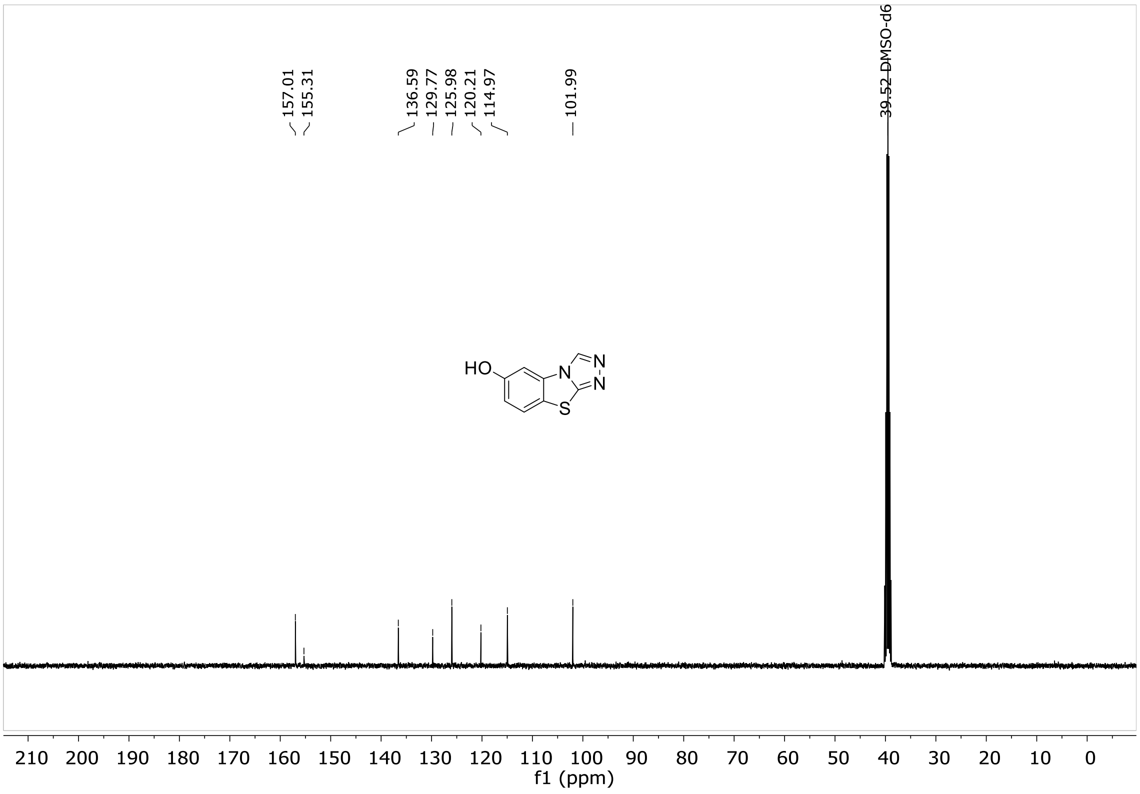


**Figure S146**: $^{13}C\{^{1}H\}$ NMR spectrum of benzo[4,5]thiazolo[2,3-*c*][1,2,4]triazol-6-ol (**6h)** (100 MHz, DMSO-$d_6$, 298 K).

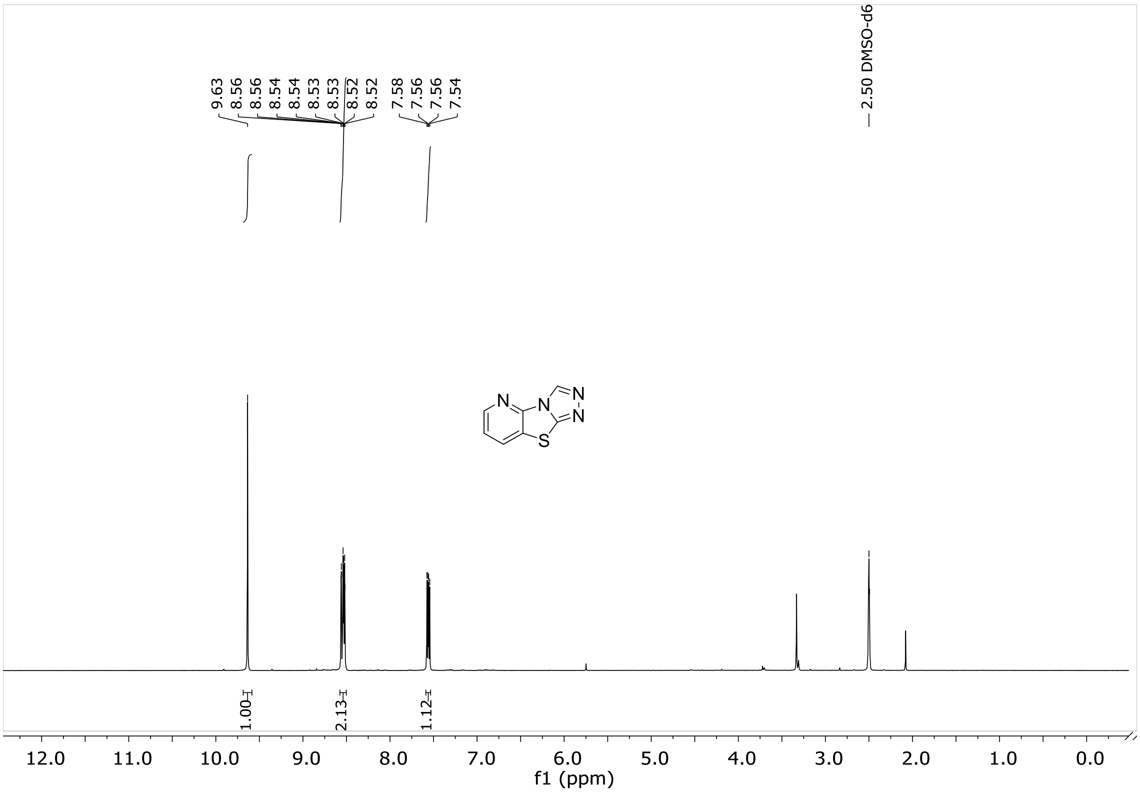


**Figure S147**: $^{1}H$ NMR spectrum of [1,2,4]triazolo[3',4':2,3]thiazolo[4,5-*b*]pyridine (**6i**) (400 MHz, DMSO-$d_6$, 298 K).

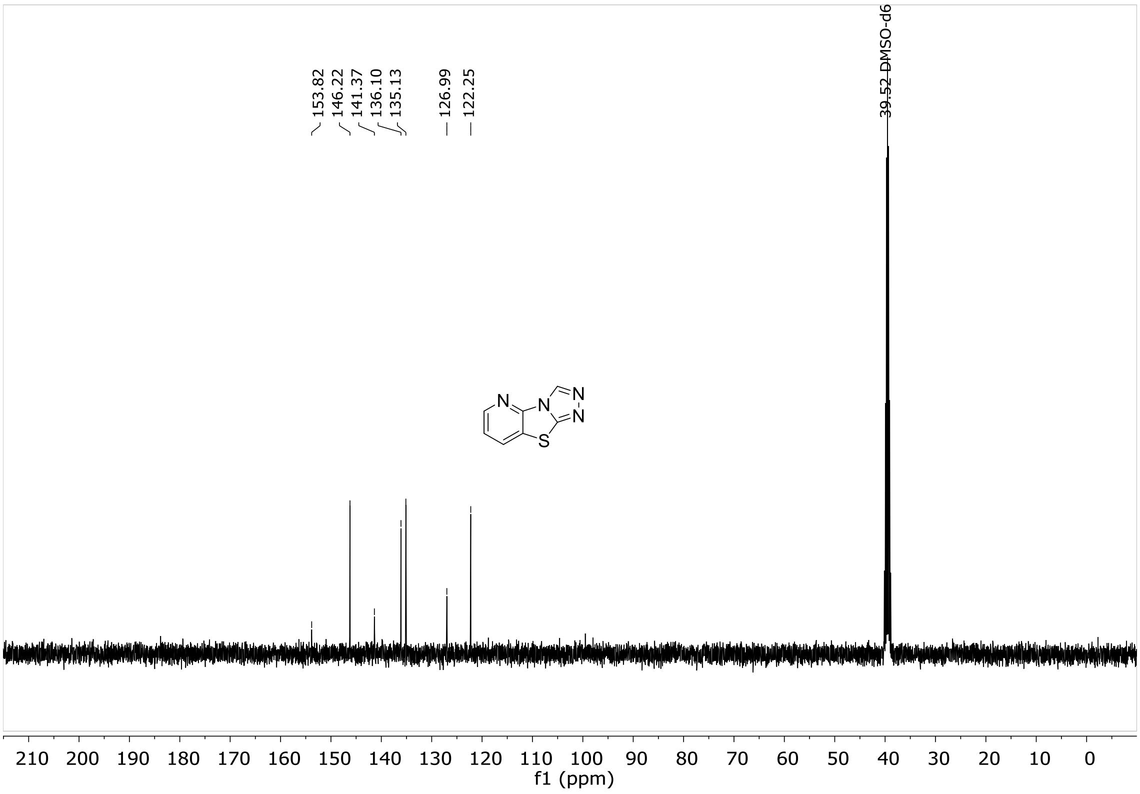


**Figure S148**: $^{13}C\{^{1}H\}$ NMR spectrum of [1,2,4]triazolo[3',4':2,3]thiazolo[4,5-*b*]pyridine (**6i**) (100 MHz, DMSO-$d_6$, 298 K).

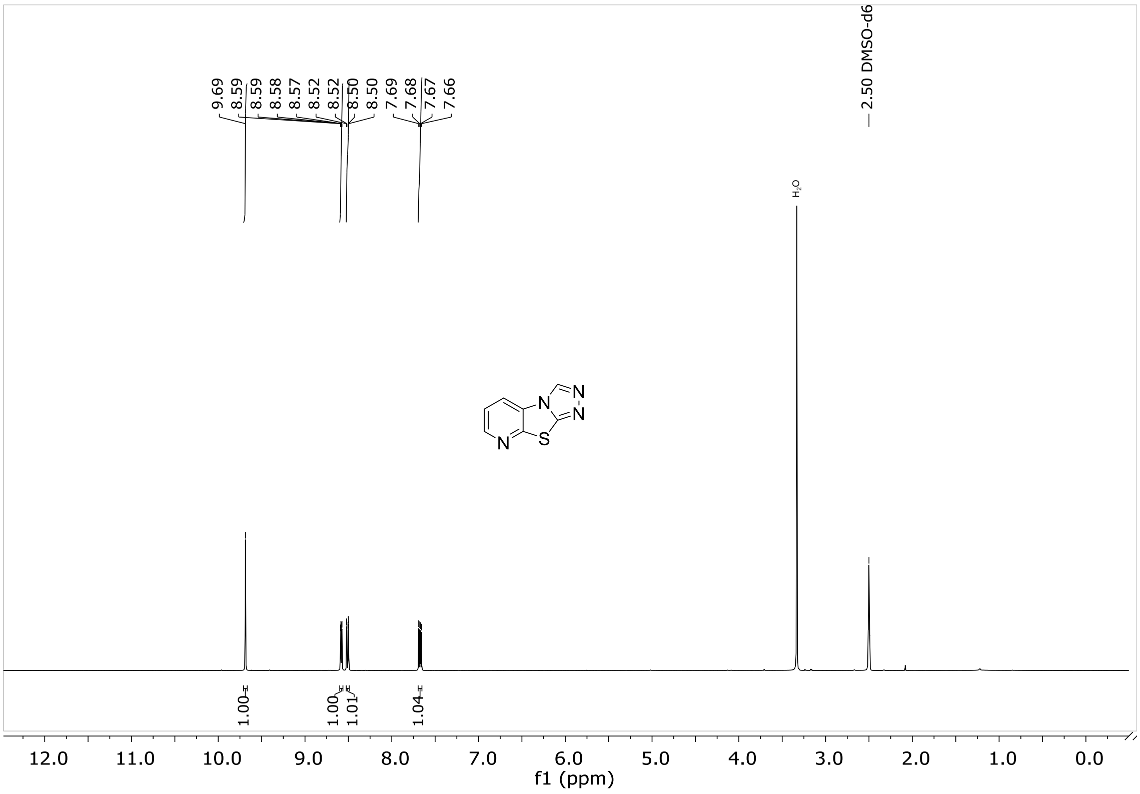


**Figure S149**: $^{1}H$ NMR spectrum of [1,2,4]triazolo[3',4':2,3]thiazolo[5,4-*b*]pyridine (**6j**) (400 MHz, DMSO-$d_6$, 298 K).

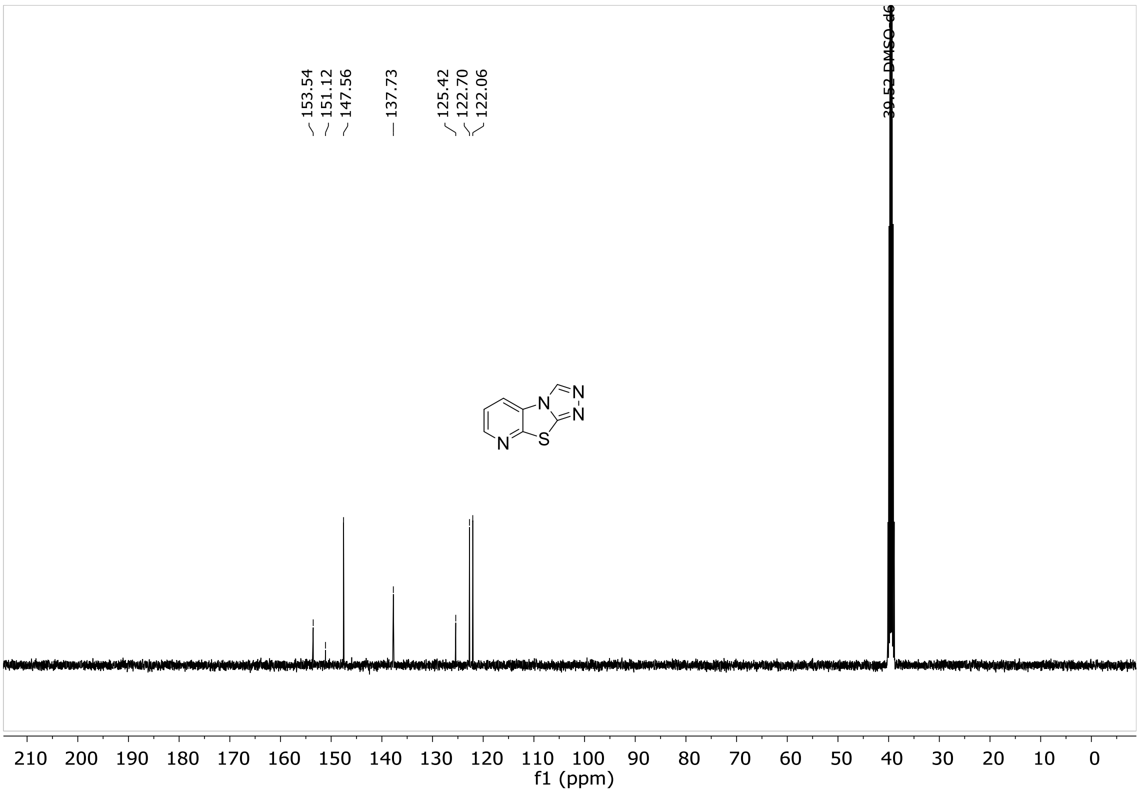


**Figure S150**: $^{13}C\{^{1}H\}$ NMR spectrum of [1,2,4]triazolo[3',4':2,3]thiazolo[5,4-*b*]pyridine (**6j**) (100 MHz, DMSO-$d_6$, 298 K).

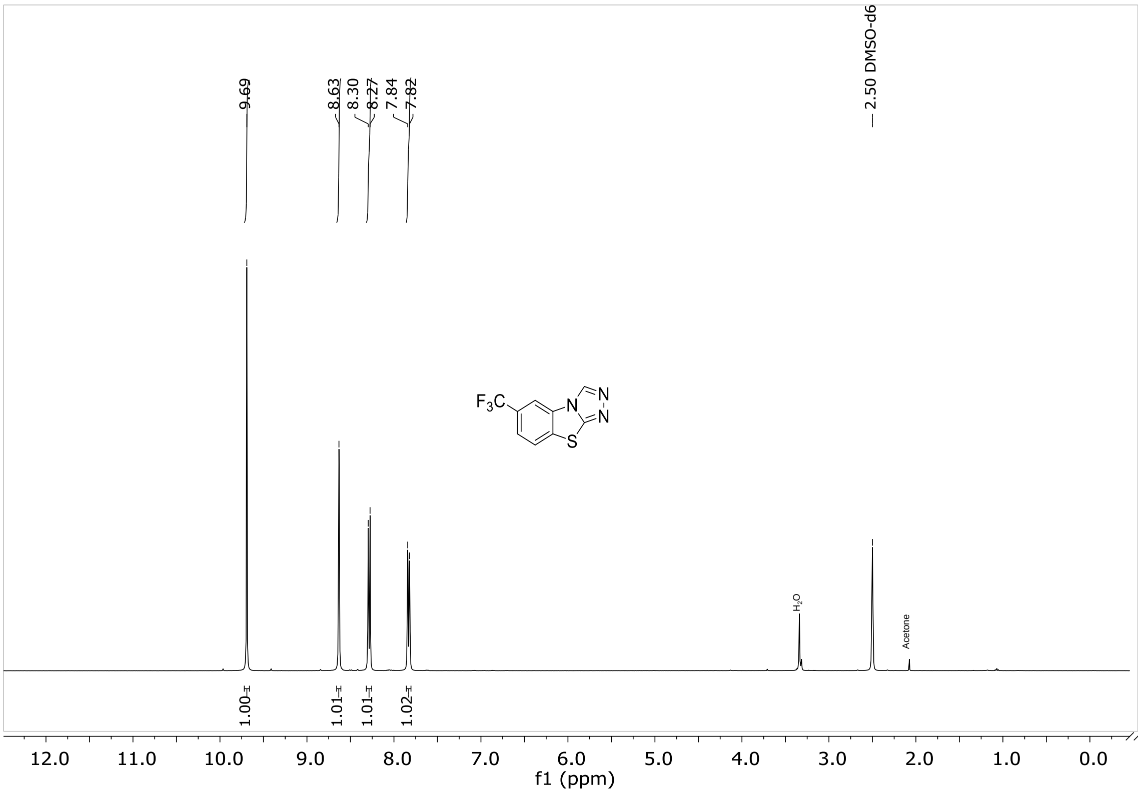


**Figure S151**: $^{1}H$ NMR spectrum of 6-(trifluoromethyl)benzo[4,5]thiazolo[2,3-*c*][1,2,4]triazole (**6k**) (400 MHz, DMSO-$d_6$, 298 K).

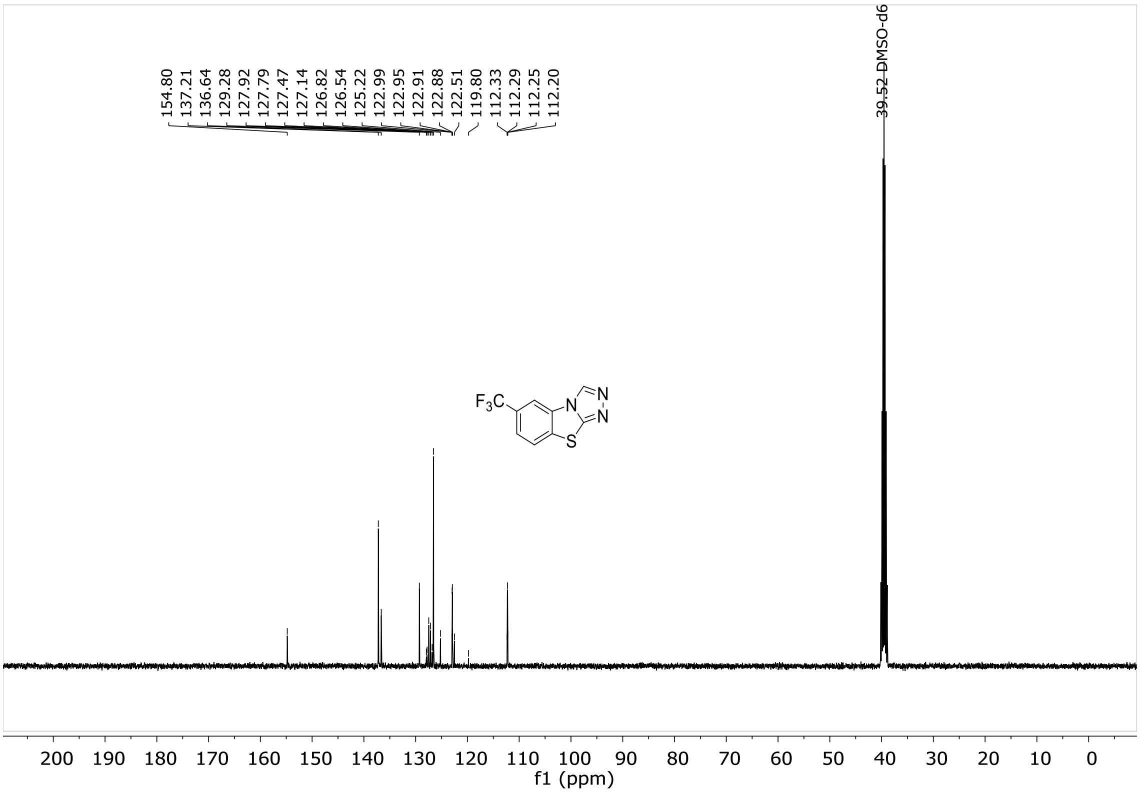


**Figure S152**: $^{13}C\{^{1}H\}$ NMR spectrum of 6-(trifluoromethyl)benzo[4,5]thiazolo[2,3-*c*][1,2,4]triazole (**6k**) (100 MHz, DMSO-$d_6$, 298 K).

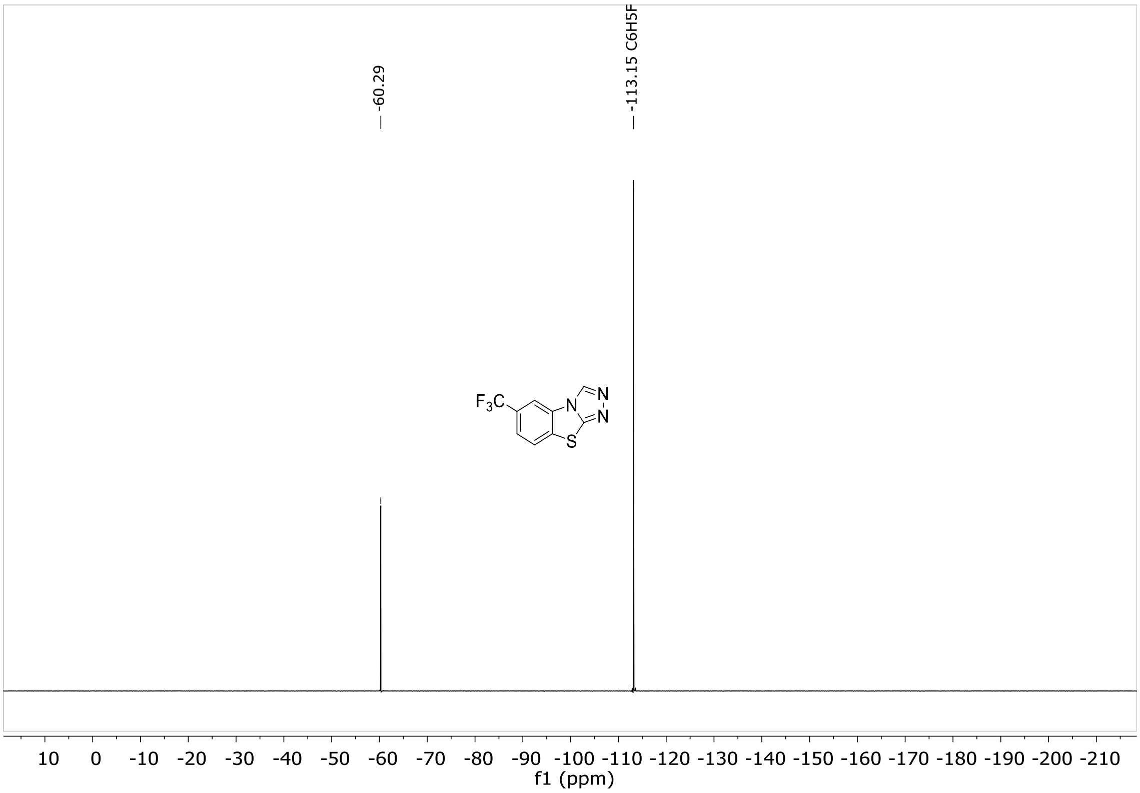


**Figure S153**: $^{19}F\{^{1}H\}$ NMR spectrum of 6-(trifluoromethyl)benzo[4,5]thiazolo[2,3-*c*][1,2,4]triazole (**6k**) (376 MHz, DMSO-$d_6$, 298 K).

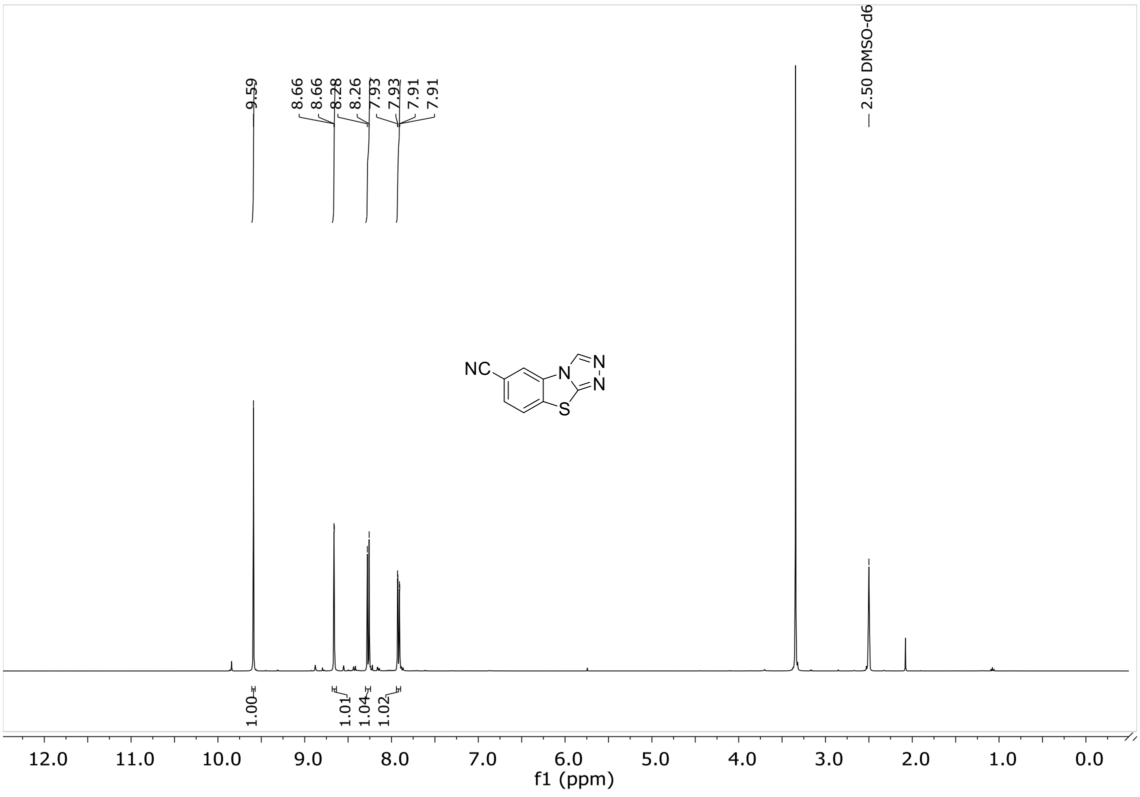


**Figure S154**: $^{1}$H NMR spectrum of benzo[4,5]thiazolo[2,3-*c*][1,2,4]triazole-6-carbonitrile (**6l**) (400 MHz, DMSO-$d_6$, 298 K).

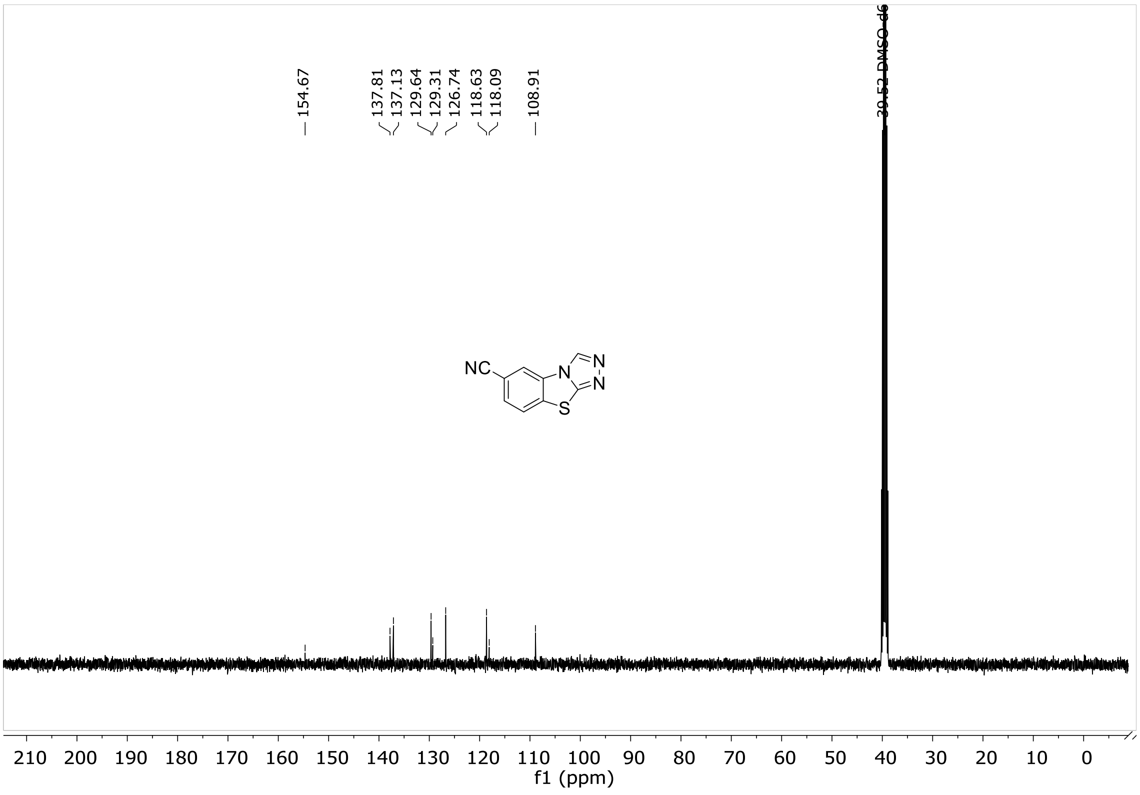


**Figure S155**: $^{13}C\{^1H\}$ NMR spectrum of benzo[4,5]thiazolo[2,3-*c*][1,2,4]triazole-6-carbonitrile (**6l**) (100 MHz, DMSO-$d_6$, 298 K).

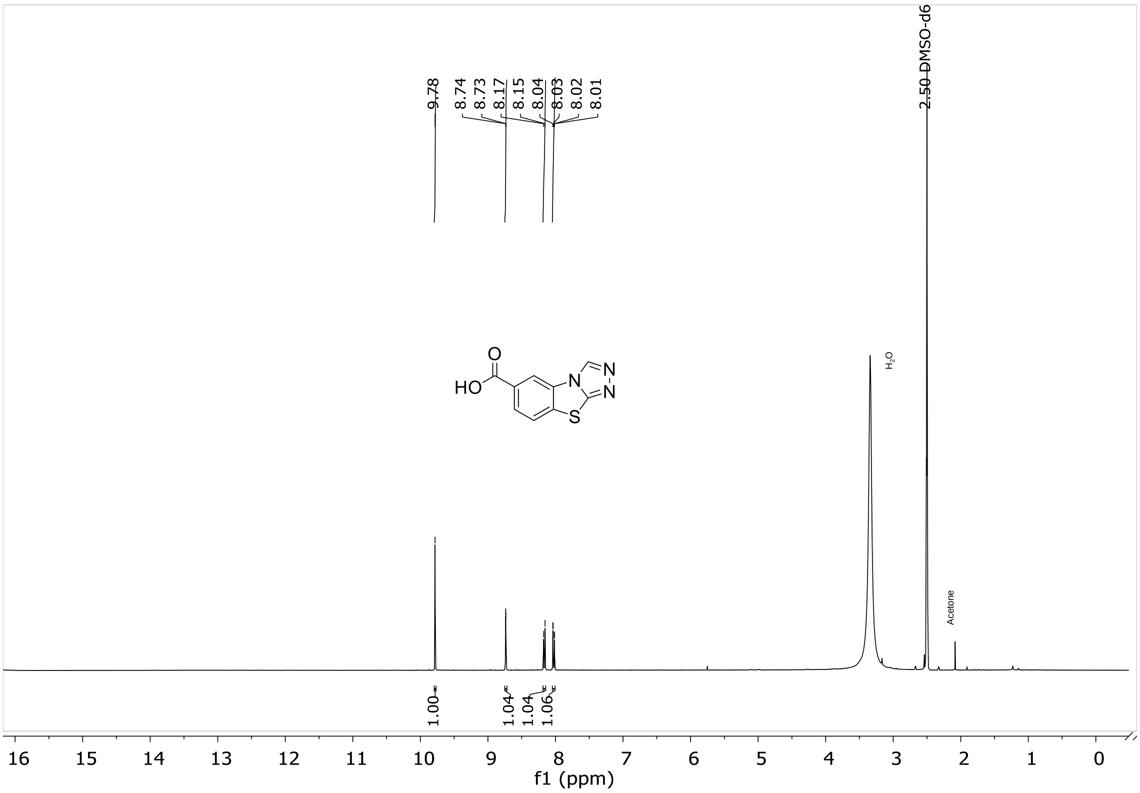


**Figure S156**: $^{1}$H NMR spectrum of benzo[4,5]thiazolo[2,3-*c*][1,2,4]triazole-6-carboxylic acid (**6m**) (400 MHz, DMSO-$d_6$, 298 K).

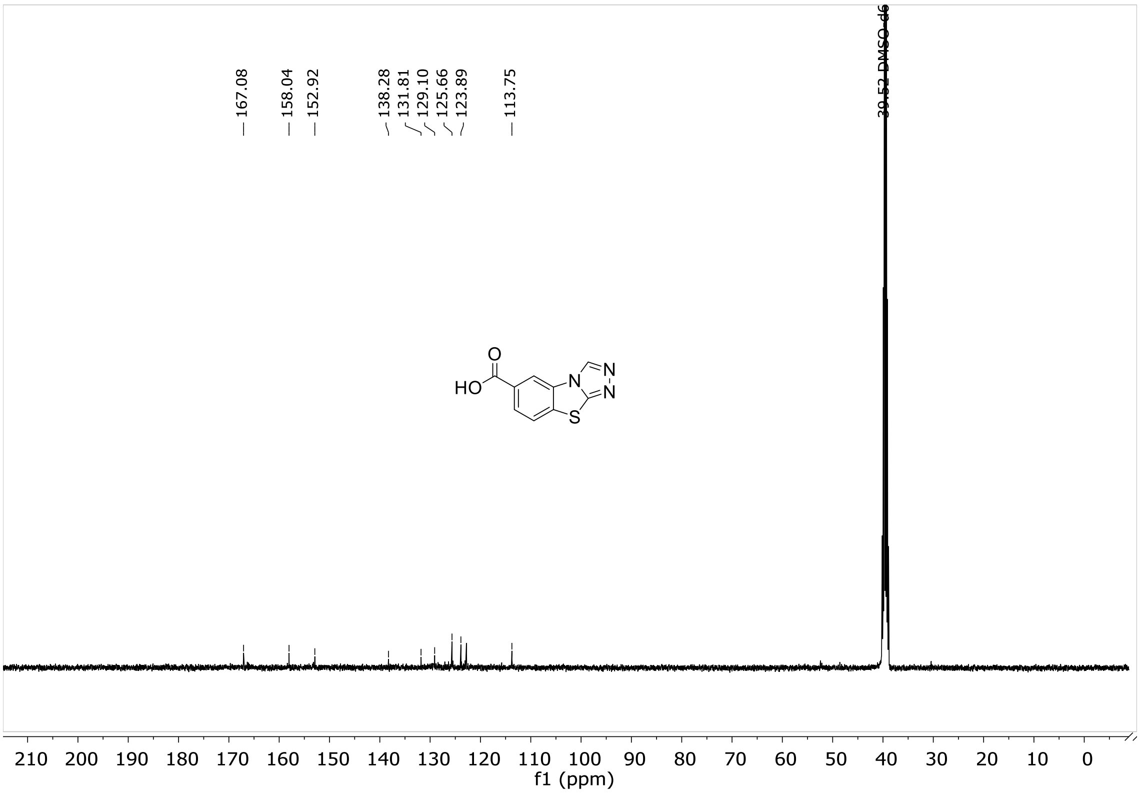


**Figure S157**: $^{13}$C{$^{1}$H} NMR spectrum of benzo[4,5]thiazolo[2,3-*c*][1,2,4]triazole-6-carboxylic acid (**6m**) (100 MHz, DMSO-$d_6$, 298 K).

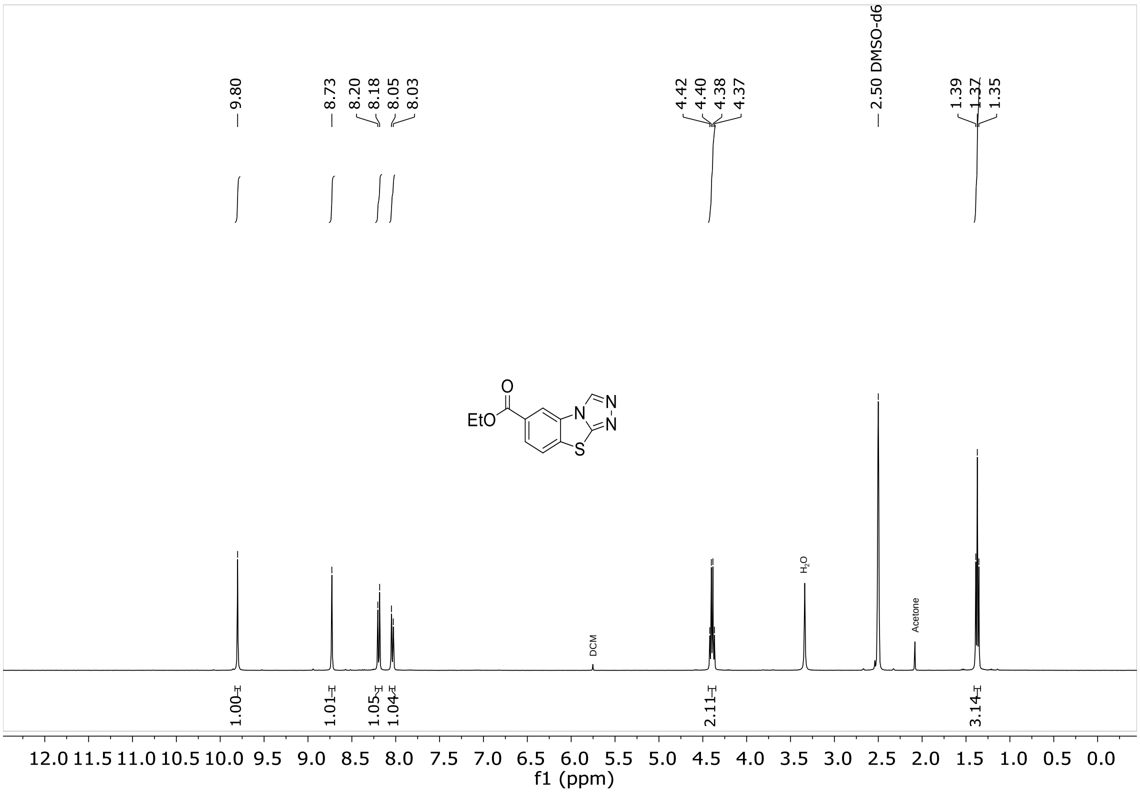


**Figure S158**: $^{1}H$ NMR spectrum of ethyl benzo[4,5]thiazolo[2,3-*c*][1,2,4]triazole-6-carboxylate (**6m'**) (400 MHz, DMSO-$d_6$, 298 K).

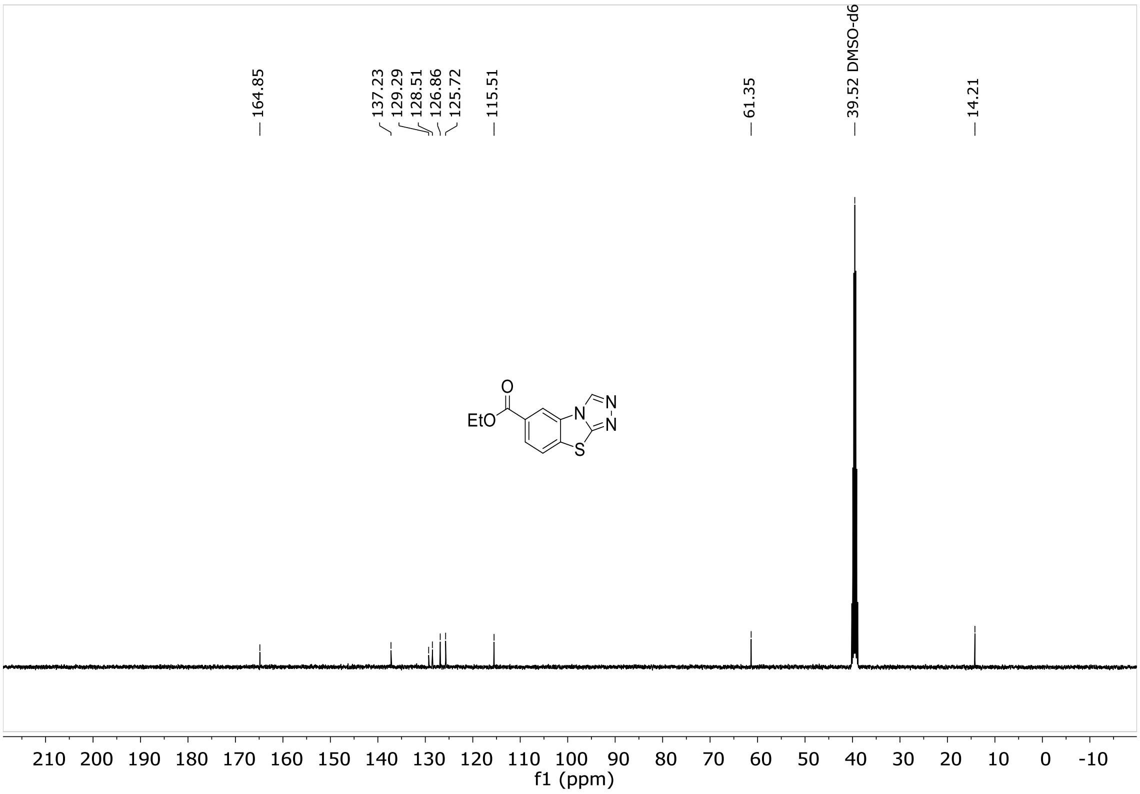


**Figure S159**: $^{13}C\{^{1}H\}$ NMR spectrum of ethyl benzo[4,5]thiazolo[2,3-*c*][1,2,4]triazole-6-carboxylate (**6m**) (100 MHz, DMSO-$d_6$, 298 K).

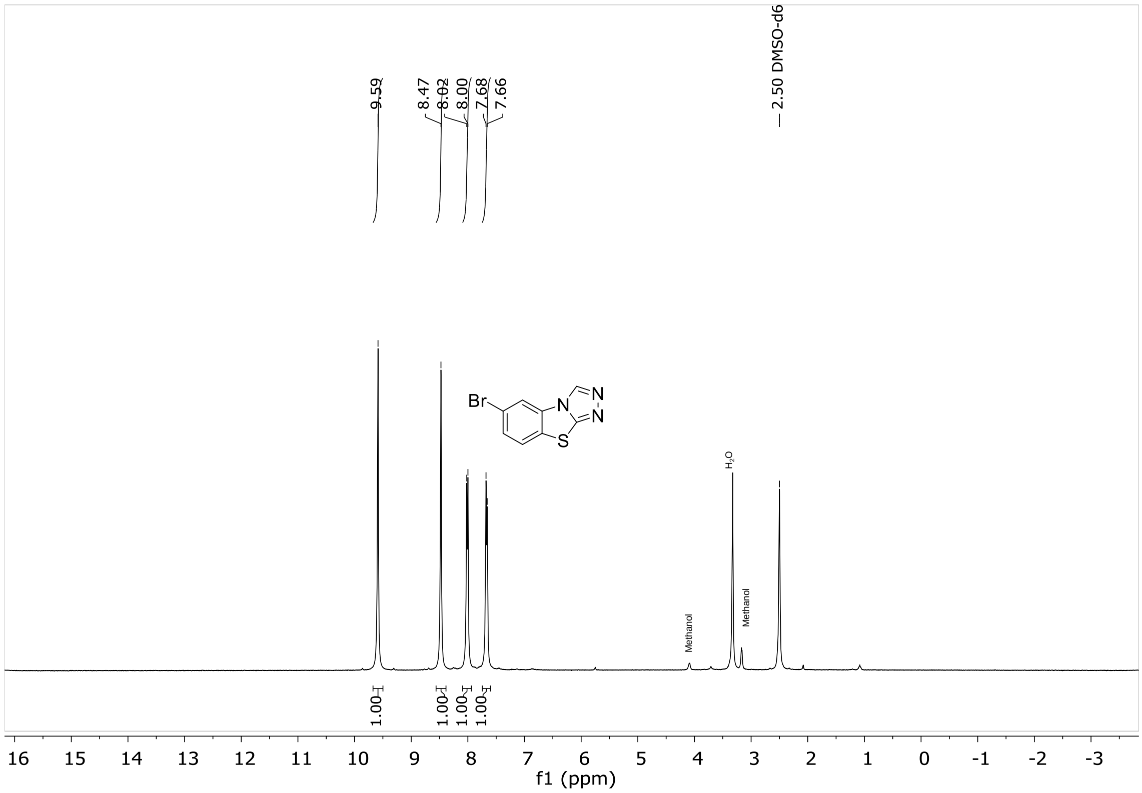


**Figure S160**: $^1$H NMR spectrum of 6-bromobenzo[4,5]thiazolo[2,3-*c*][1,2,4]triazole (**6n**) (400 MHz, DMSO-$d_6$, 298 K).

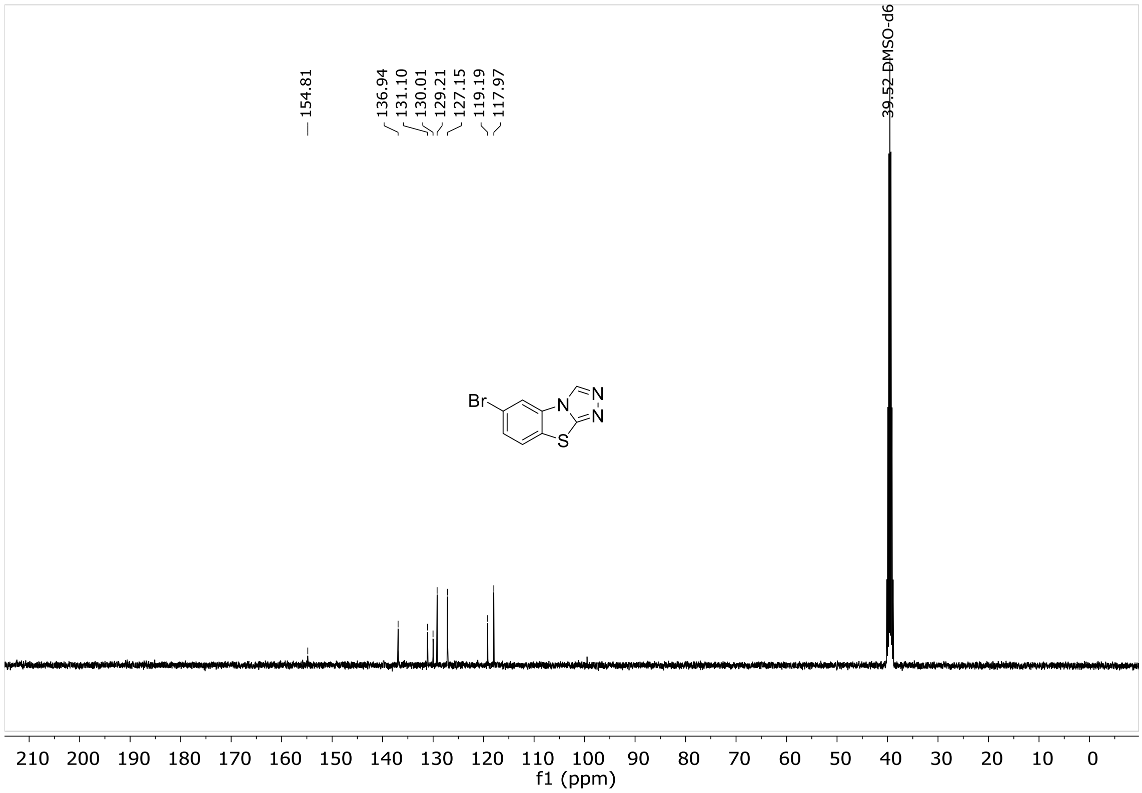


**Figure S161**: $^{13}$C NMR spectrum of 6-bromobenzo[4,5]thiazolo[2,3-*c*][1,2,4]triazole (**6n**) (100 MHz, DMSO-$d_6$, 298 K).

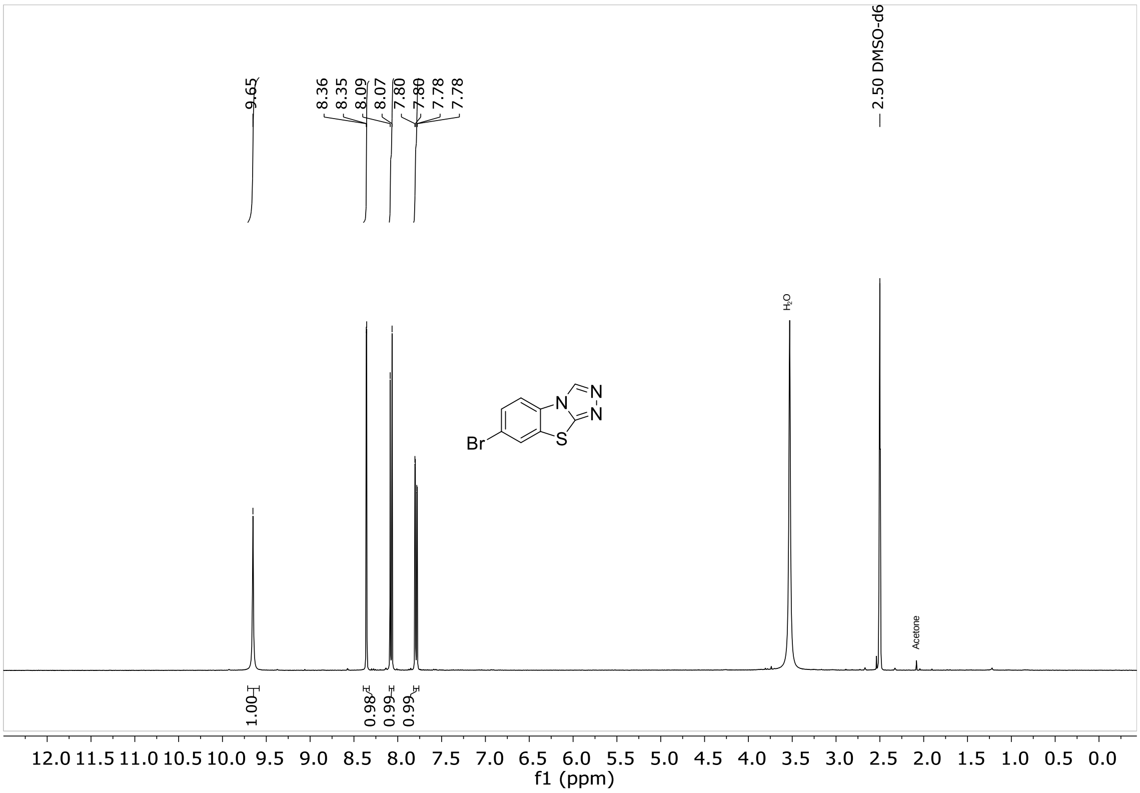


**Figure S162**: $^{1}$H NMR spectrum of 7-bromobenzo[4,5]thiazolo[2,3-*c*][1,2,4]triazole (**6o**) (400 MHz, DMSO-$d_6$, 298 K).

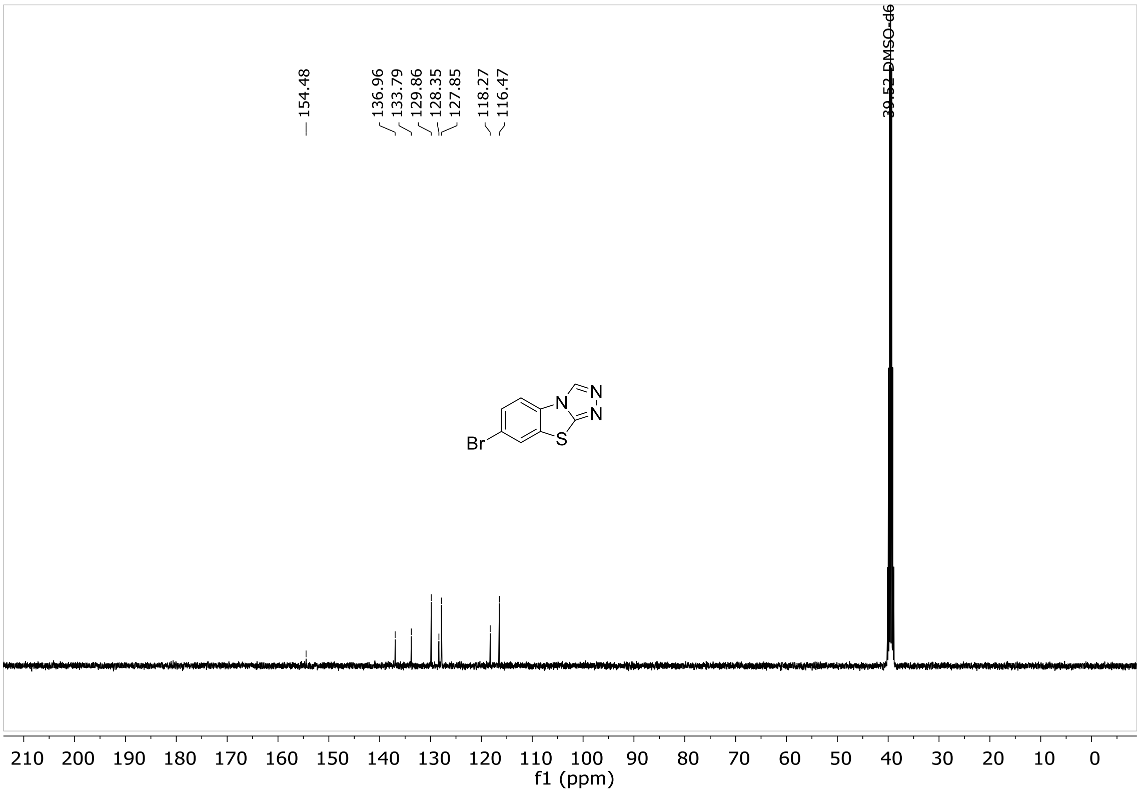


**Figure S163**: $^{13}$C{$^{1}$H} NMR spectrum of 7-bromobenzo[4,5]thiazolo[2,3-*c*][1,2,4]triazole (**6o**) (100 MHz, DMSO-$d_6$, 298 K).

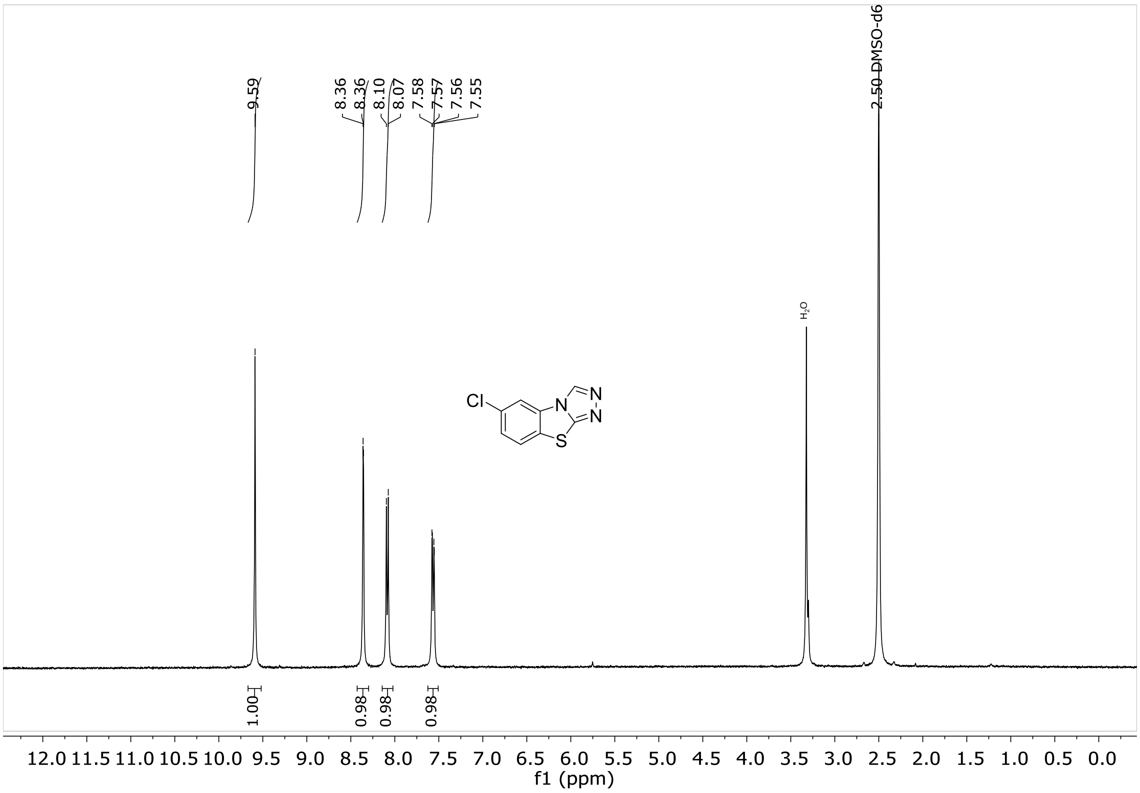


**Figure S164**: $^{1}H$ NMR spectrum of 6-chlorobenzo[4,5]thiazolo[2,3-*c*][1,2,4]triazole (**6p**) (400 MHz, DMSO-$d_6$, 298 K).

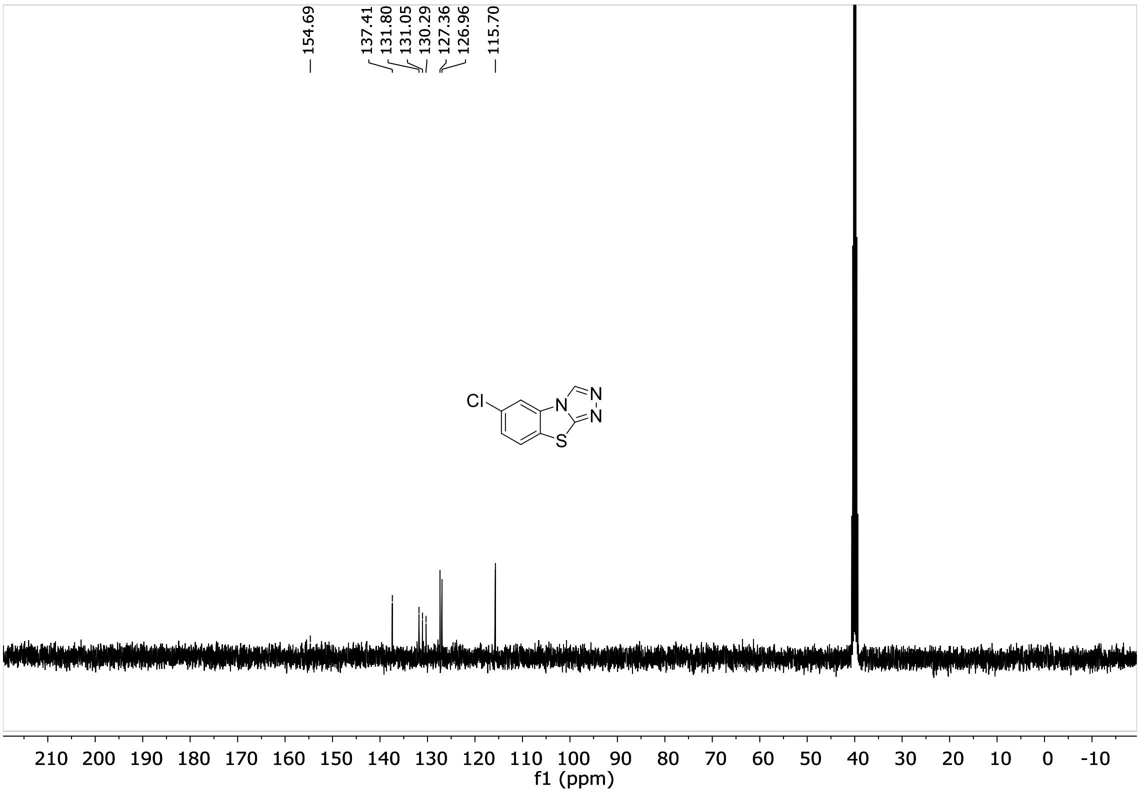


**Figure S165**: $^{13}C\{^{1}H\}$ NMR spectrum of 6-chlorobenzo[4,5]thiazolo[2,3-*c*][1,2,4]triazole (**6p**) (100 MHz, DMSO-$d_6$, 298 K).

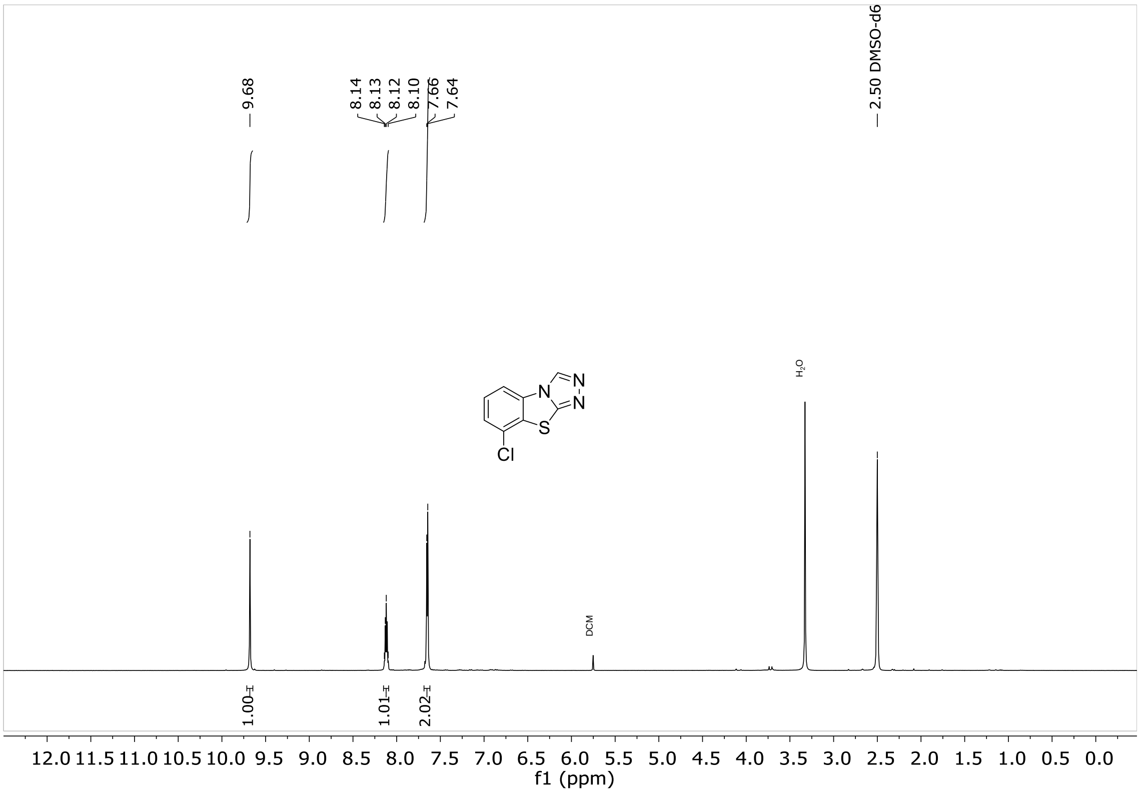


**Figure S166**: $^{1}$H NMR spectrum of 8-chlorobenzo[4,5]thiazolo[2,3-*c*][1,2,4]triazole (**6q**) (400 MHz, DMSO-$d_6$, 298 K).

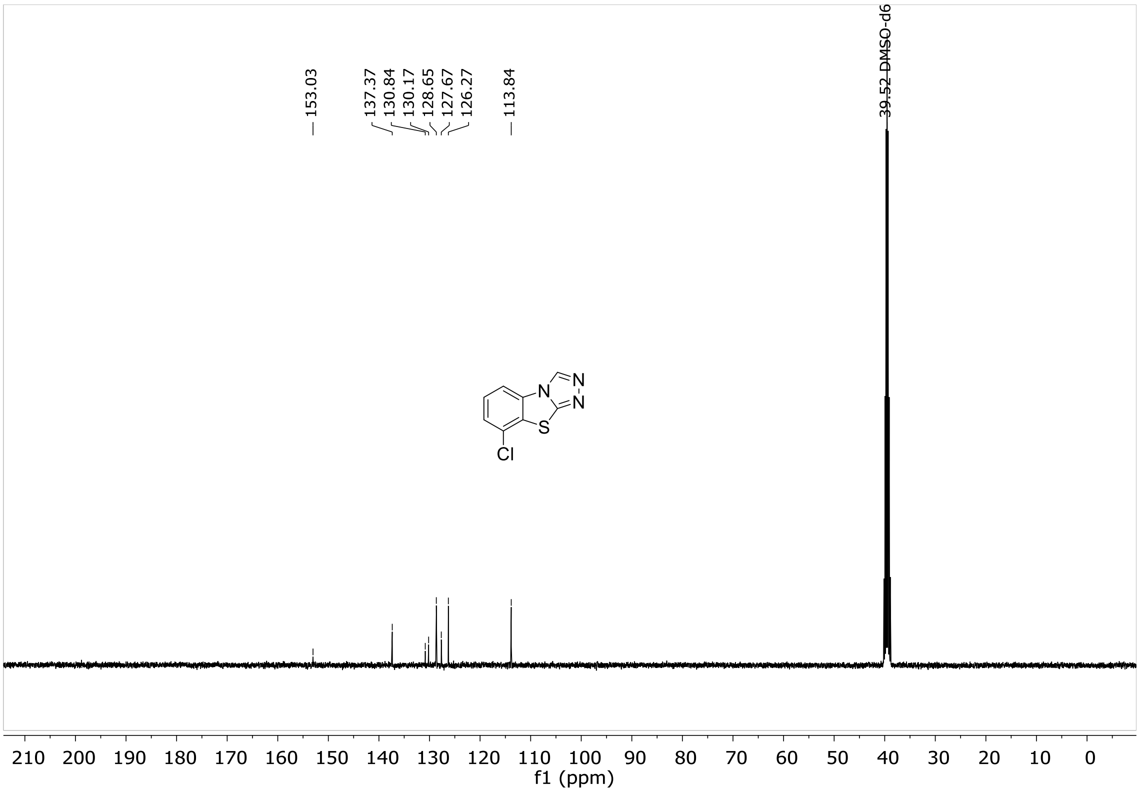


**Figure S167**: $^{13}$C{$^{1}$H} NMR spectrum of 8-chlorobenzo[4,5]thiazolo[2,3-*c*][1,2,4]triazole (**6q**) (100 MHz, DMSO-$d_6$, 298 K).

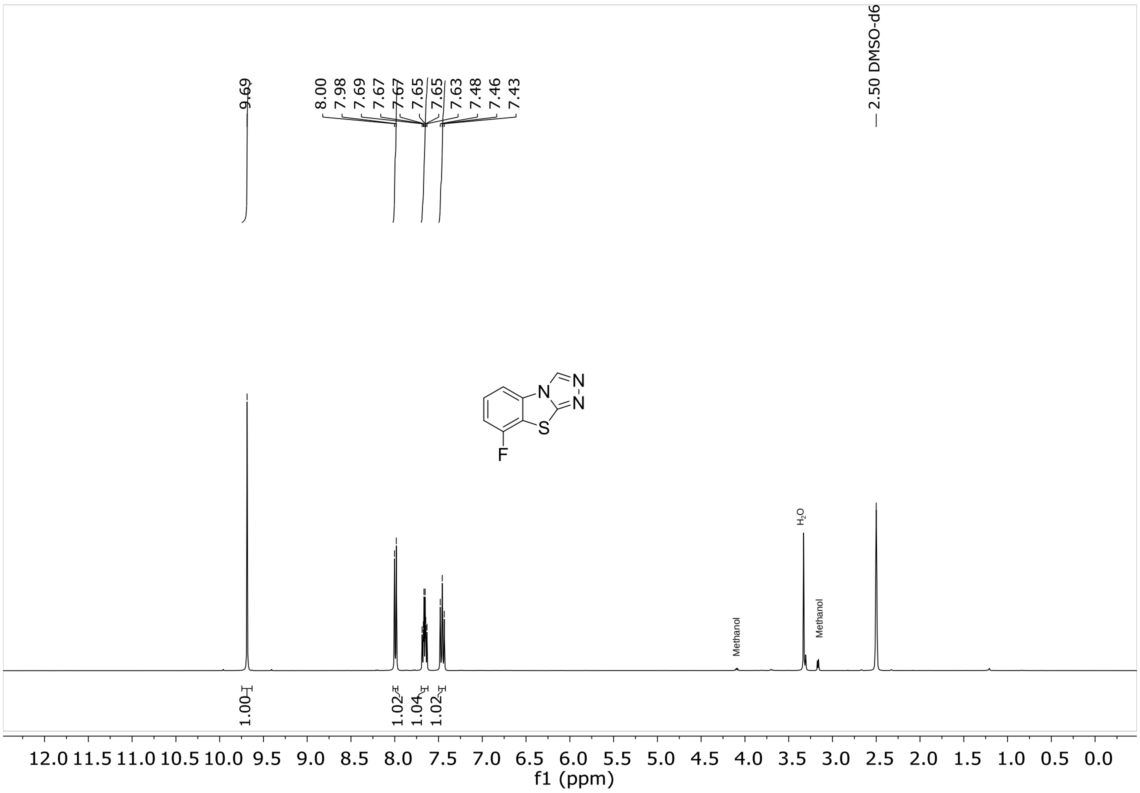


**Figure S168**: $^{1}$H NMR spectrum of 8-fluorobenzo[4,5]thiazolo[2,3-*c*][1,2,4]triazole (**6r**) (400 MHz, DMSO-$d_6$ 298 K).

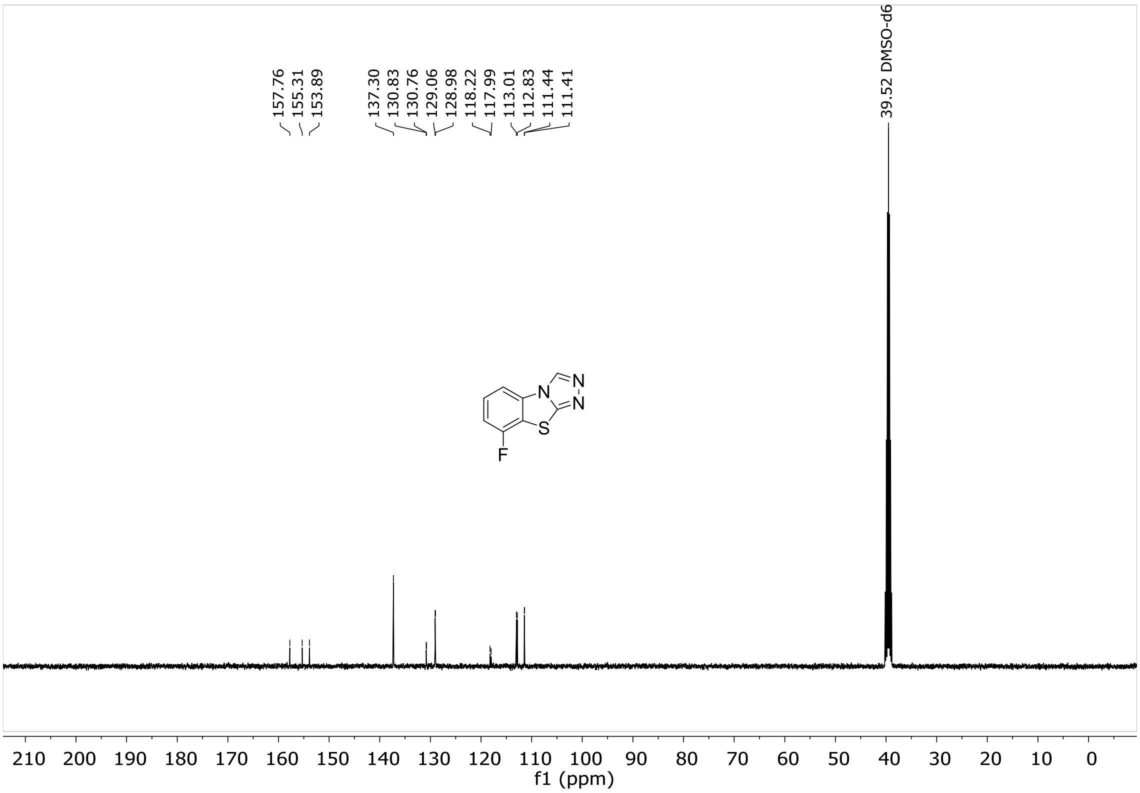


**Figure S169**: $^{13}$C{$^{1}$H} NMR spectrum of 8-fluorobenzo[4,5]thiazolo[2,3-*c*][1,2,4]triazole (**6r**) (100 MHz, DMSO-$d_6$, 298 K).

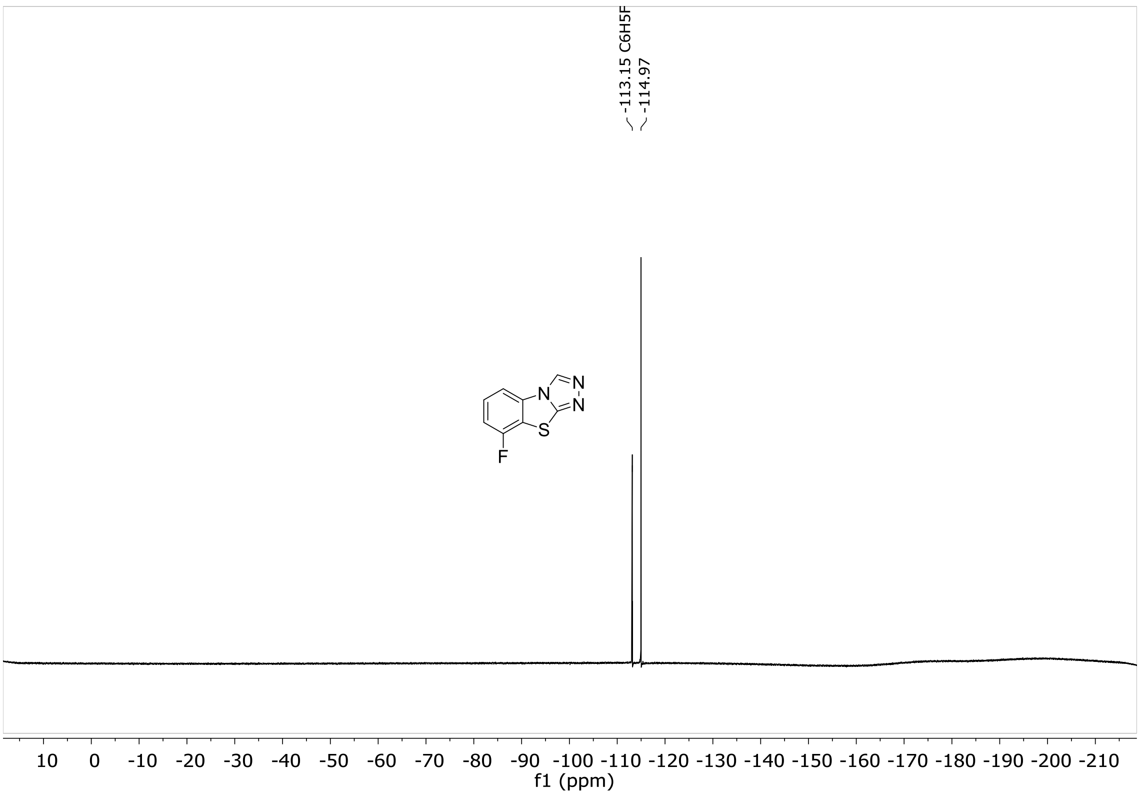


**Figure S170**: $^{19}F\{^1H\}$ NMR spectrum of 8-fluorobenzo[4,5]thiazolo[2,3-*c*][1,2,4]triazole (**6r**) (376 MHz, DMSO-$d_6$ 298 K, referenced to fluorobenzene).

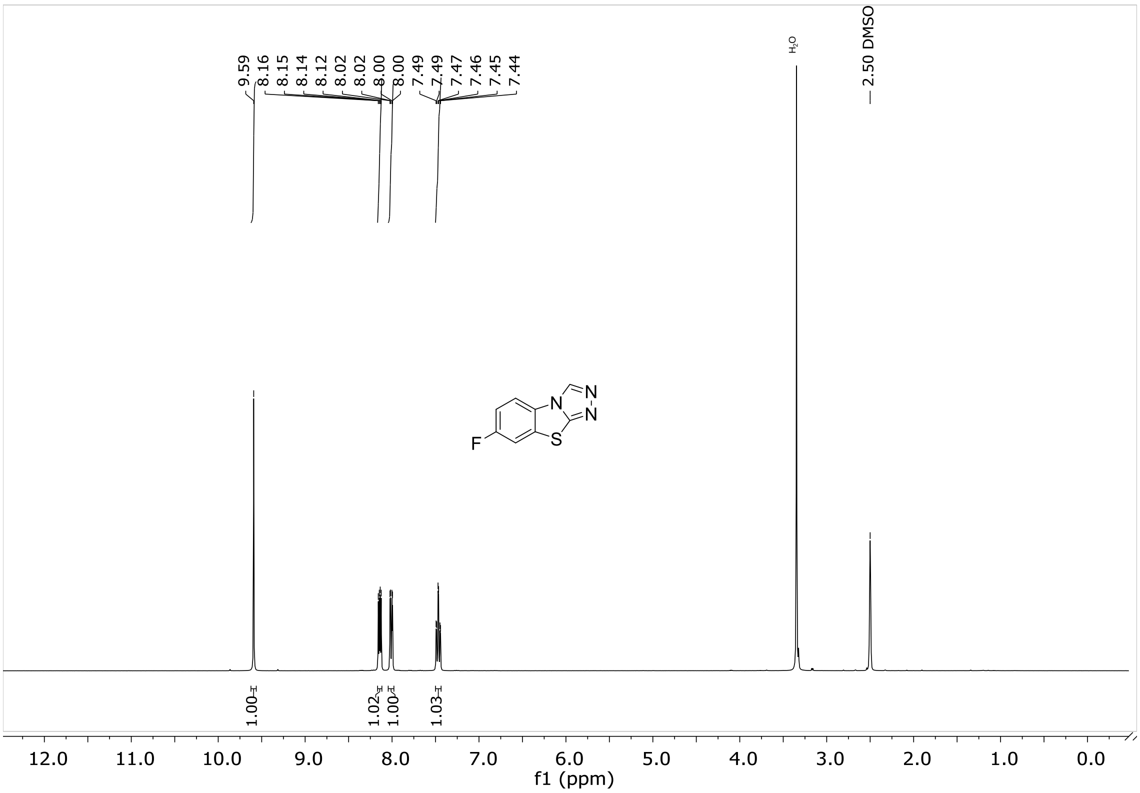


**Figure S171**: $^1H$ NMR spectrum of 7-fluorobenzo[4,5]thiazolo[2,3-*c*][1,2,4]triazole (**6s**) (400 MHz, DMSO-$d_6$, 298 K).

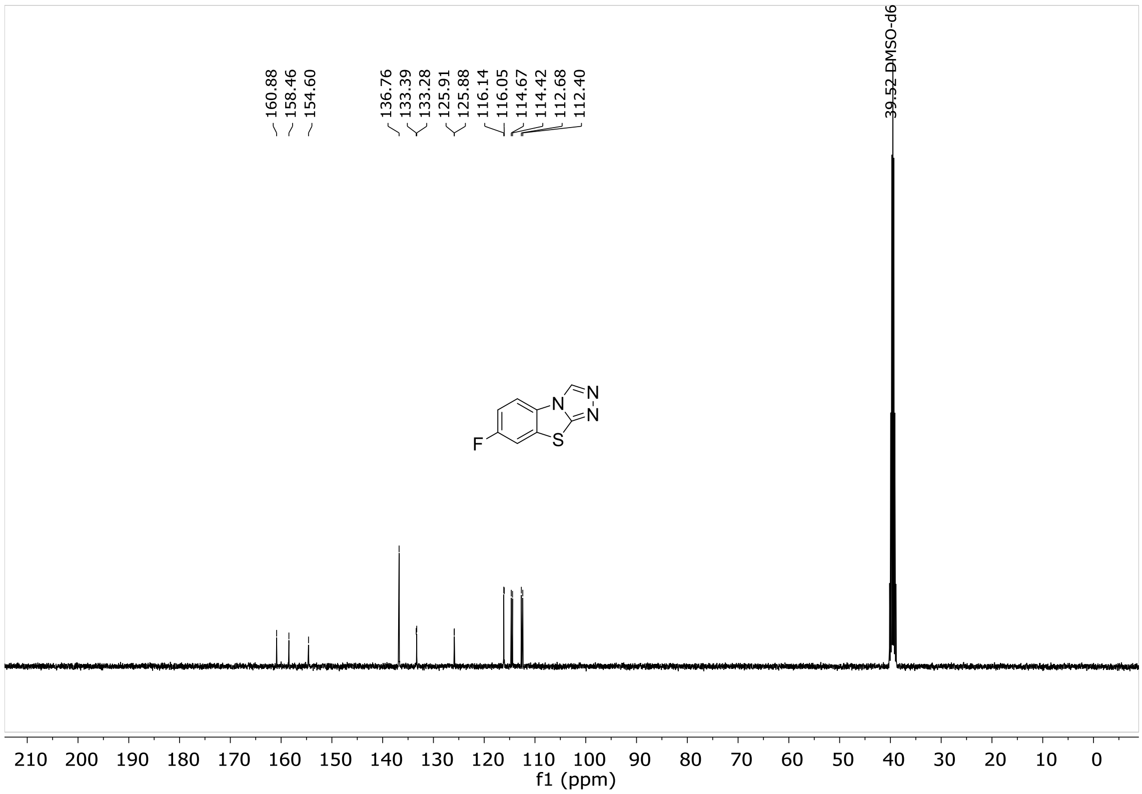


**Figure S172**: $^{13}C\{^{1}H\}$ NMR spectrum of 7-fluorobenzo[4,5]thiazolo[2,3-*c*][1,2,4]triazole (**6s**) (100 MHz, DMSO-$d_6$, 298 K).

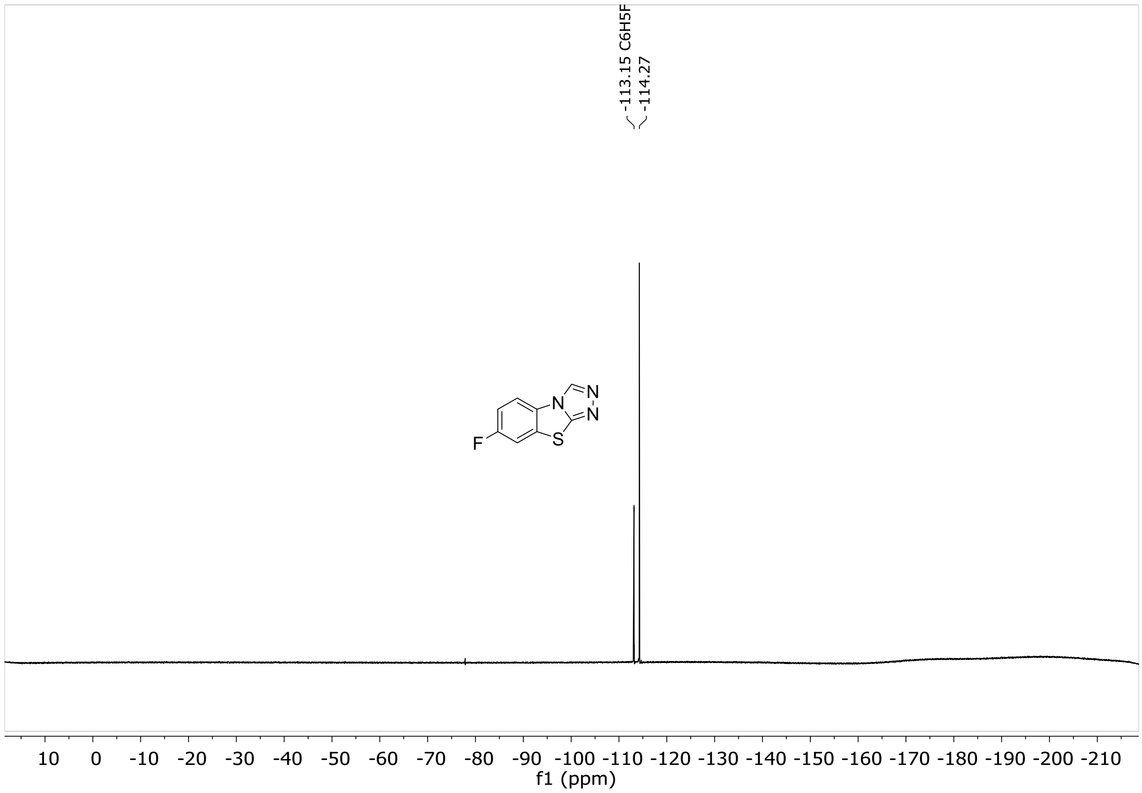


**Figure S173**: $^{19}F\{^{1}H\}$ NMR spectrum of 7-fluorobenzo[4,5]thiazolo[2,3-*c*][1,2,4]triazole (**6s**) (376 MHz, DMSO-$d_6$, 298 K).
